# Supplementary material for: Superstructure Detection in Nucleosome Distribution Shows Common Pattern within a Chromosome and within the Genome
Source: Life (Basel). 2022 Apr 6;12(4):541. doi: 10.3390/life12040541 (PMC9026121; doi:10.3390/life12040541)
Supplement: Supplementary file 1 [file life-12-00541-s001.zip › life-1592845-supplementary/supplementary.pdf]

Supporting information

1.1 Coarse-Graining

Coarse-graining is a procedure that has successfully been developed and applied to critical phenomena in physics. The basic idea is that each system has a fundamental length scale on which the physical interactions play out. While there are interactions such as excluded volume interaction or Van-der-Waals interactions on a short scale, these all add up to the relevant scale given by the typical correlation length of the system. If the correlations are small, such as in a gas where the constituents particles almost never interact then the fundamental interactions determine the physical scale. For more dense system, there is a scale, the correlation length, on which the system needs to be described.

The coarse-graining procedure is demonstrated in Figure. Panel A shows a noisy signal based on the data shown in panel E. For panels B to D we increase the coarse-graining length L from 10 to 50 and to 250. The first coarse-graining step shown in panel B already recovers some aspects of the underlying data. The second coarse-graining length $L = 50$ essentially has recovered the underlying structure while for $L = 250$ the signal is too much washed out.

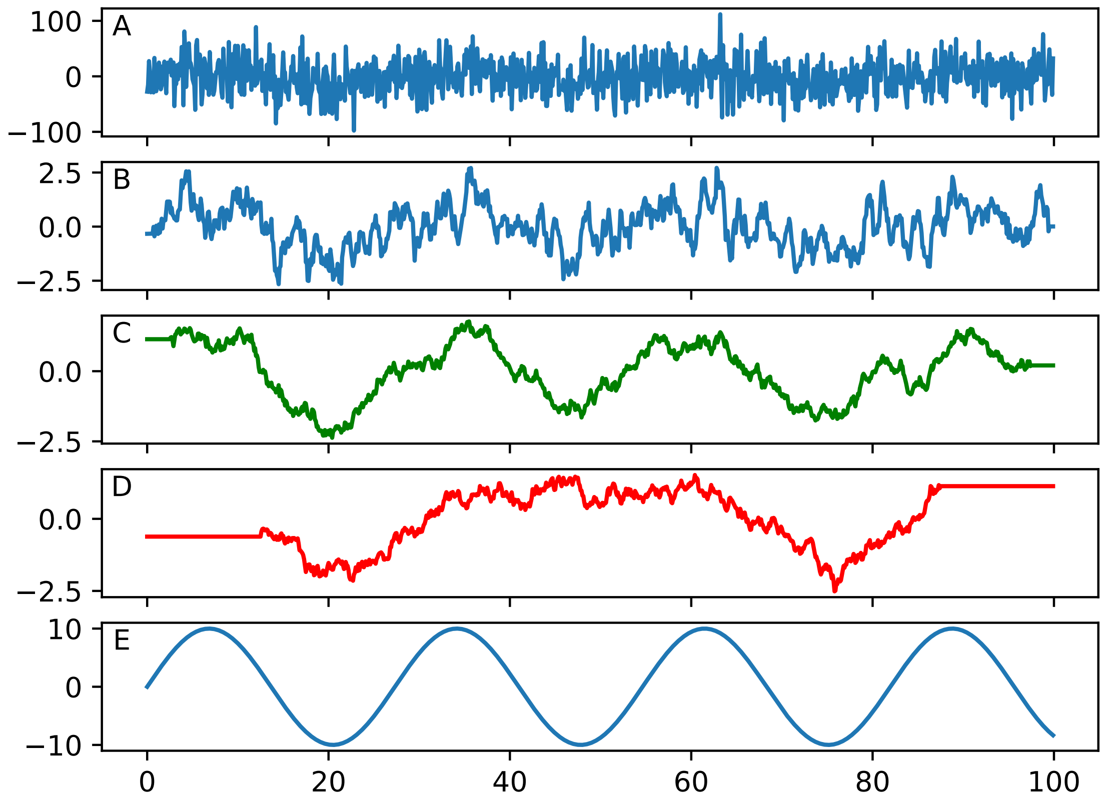

1.2 Nucleosome Density

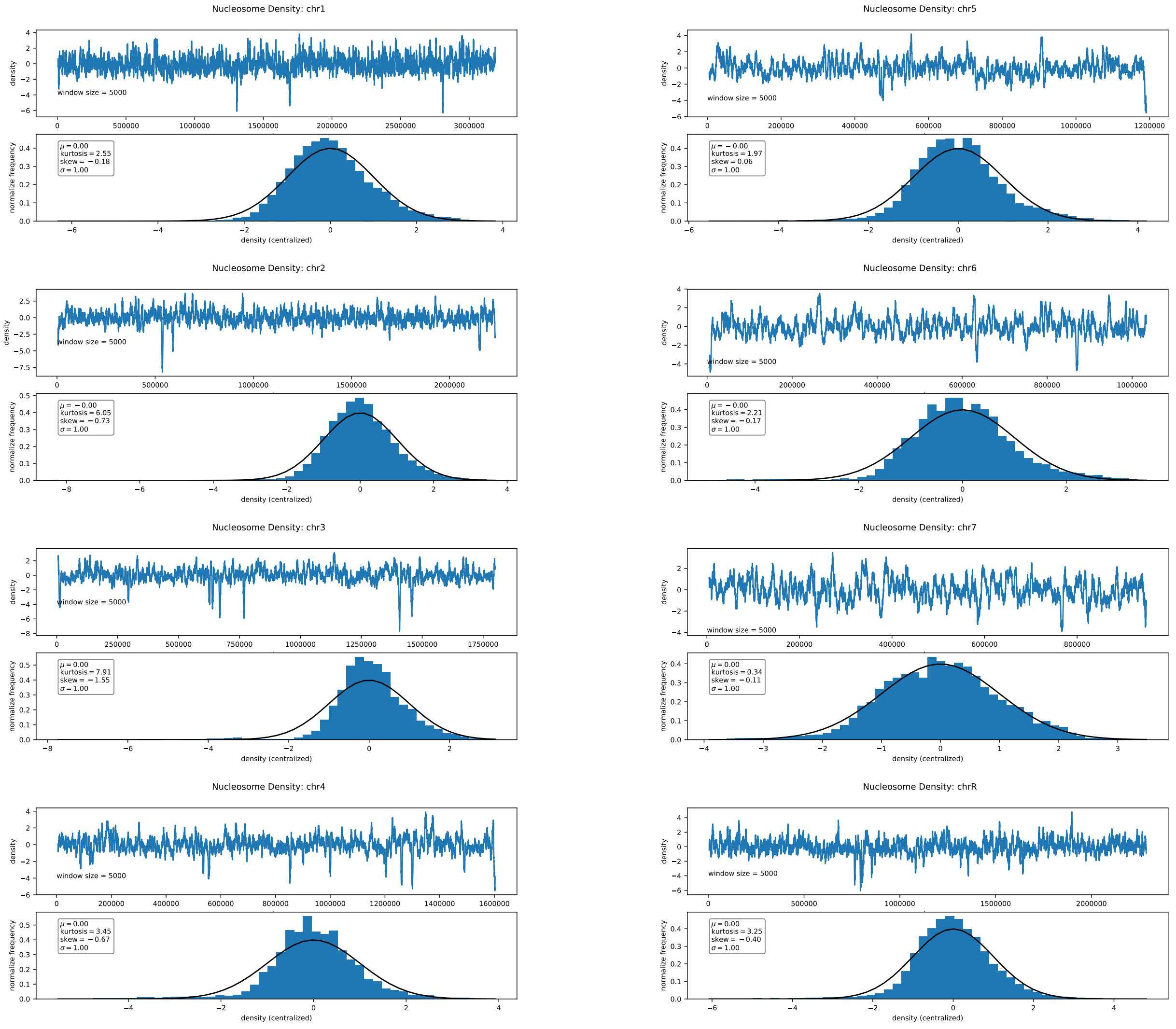

Figure S1: Shown is the nucleosomal density after applying a rolling average with a window size of 5000 of all of the chromosomes (upper panels). The lower panels show the corresponding histogram of the densities with a bin size of 50. The black line is the fit with a gaussian distribution.

1.2.1 Nucleosome Density at $b = 2500$

Chromosome	Distribution	chi_square	D_statistic
chr1	fisk	1.675740e+05	0.026701
chr1	norm	4.029705e+05	0.047346
chr2	fisk	2.215488e+05	0.034197
chr2	norm	4.888579e+05	0.049294
chr3	fisk	2.315703e+05	0.038608
chr3	norm	1.174085e+06	0.083966
chr4	fisk	1.530916e+05	0.034538
chr4	norm	6.824904e+05	0.070372
chr5	fisk	9.322028e+04	0.030918
chr5	norm	2.783759e+05	0.056306
chr6	fisk	1.280710e+05	0.037654
chr6	norm	2.753396e+05	0.052656
chr7	fisk	5.100021e+04	0.032512
chr7	norm	7.679109e+04	0.031660
chrR	fisk	2.258400e+05	0.033580
chrR	norm	6.023527e+05	0.054608

Table S1: The Fisk distribution, also known as the log-logistic distribution gives the best consistent fit. The fit was done for the bin size of 50 and the rolling average of size 5000. Statistical Kolmogorov-Smirnov test for goodness of fit was done using SciPy.org scipy.stats.kstest function **?**. The D statistic is the absolute max distance (supremum) between the CDFs of the two samples. All results show small values D values corresponding to p-values close to 1, the log-logistic distribution may explain the data.

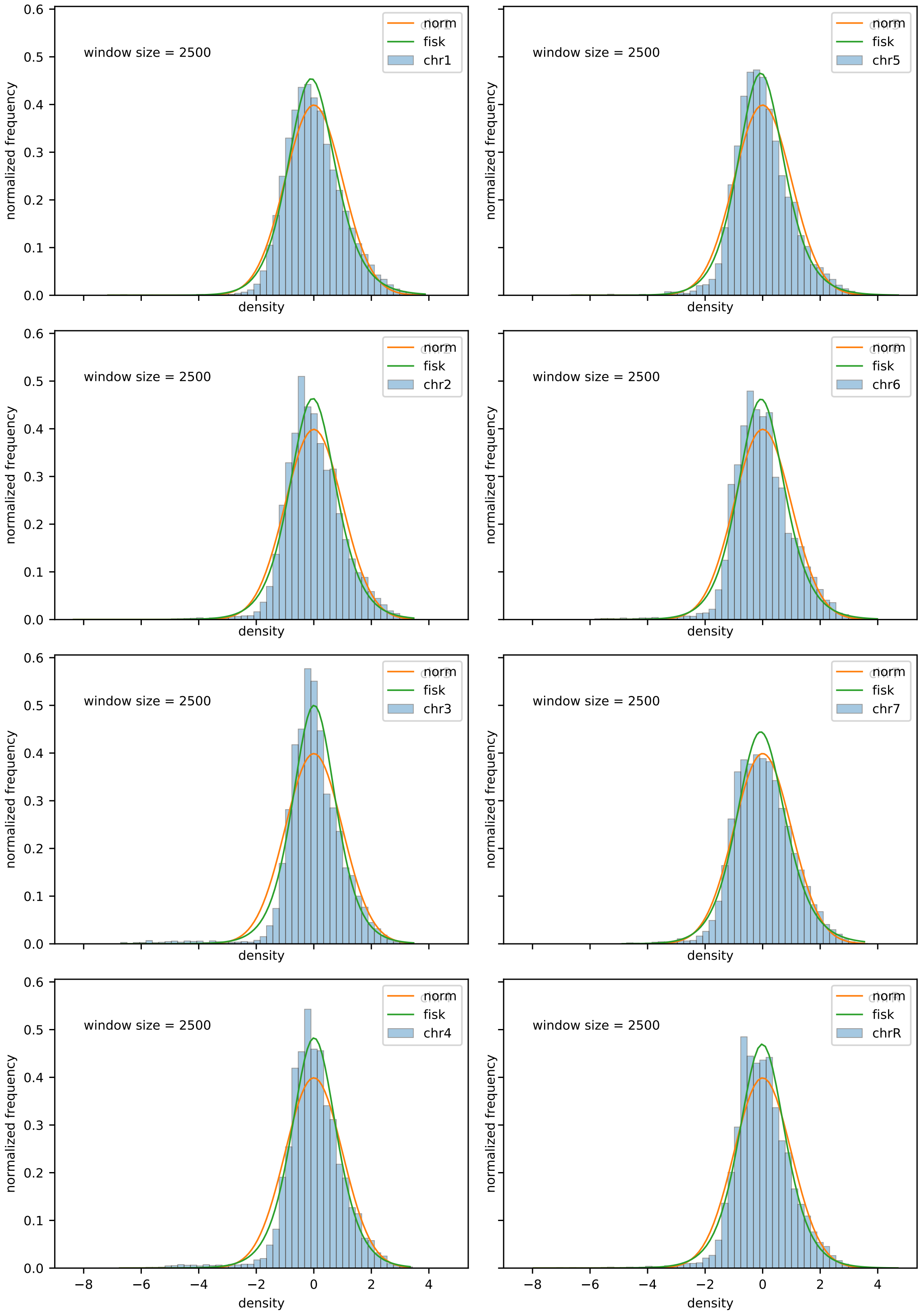

Figure S2: Normalized nucleosome density distributions for all of the chromosomes. The data shows the non-gaussian behavior (red line). For comparison a fit to a Log-logistic distribution is shown yielding a much better consistent fit. The bin size was 50 and the rolling average of size 2500 was used.

1.2.2 Nucleosome Density at $b = 5000$

Chromosome	Distribution	chi_square	D_statistic
chr1	fisk	1.678610e+05	0.021809
chr1	norm	4.310806e+05	0.040372
chr2	fisk	1.011539e+05	0.024922
chr2	norm	4.873082e+05	0.048215
chr3	fisk	2.078474e+05	0.038179
chr3	norm	1.362080e+06	0.094966
chr4	fisk	9.270418e+04	0.027728
chr4	norm	6.014198e+05	0.069622
chr5	fisk	4.004712e+04	0.020815
chr5	norm	1.715085e+05	0.048451
chr6	fisk	1.347119e+04	0.020806
chr6	norm	1.609603e+05	0.038205
chr7	fisk	1.682594e+04	0.022172
chr7	norm	1.636277e+04	0.016584
chrR	fisk	9.955245e+04	0.025810
chrR	norm	4.967464e+05	0.052443

Table S2: The Fisk distribution, also known as the log-logistic distribution gives the best consistent fit. The fit was done for the bin size of 50 and the rolling average of size 5000. Statistical Kolmogorov-Smirnov test for goodness of fit was done using SciPy.org scipy.stats.kstest function **?**. The D statistic is the absolute max distance (supremum) between the CDFs of the two samples. As the all the results show small values D values corresponding to p-values close to 1, the log-logistic distribution may explain the data.

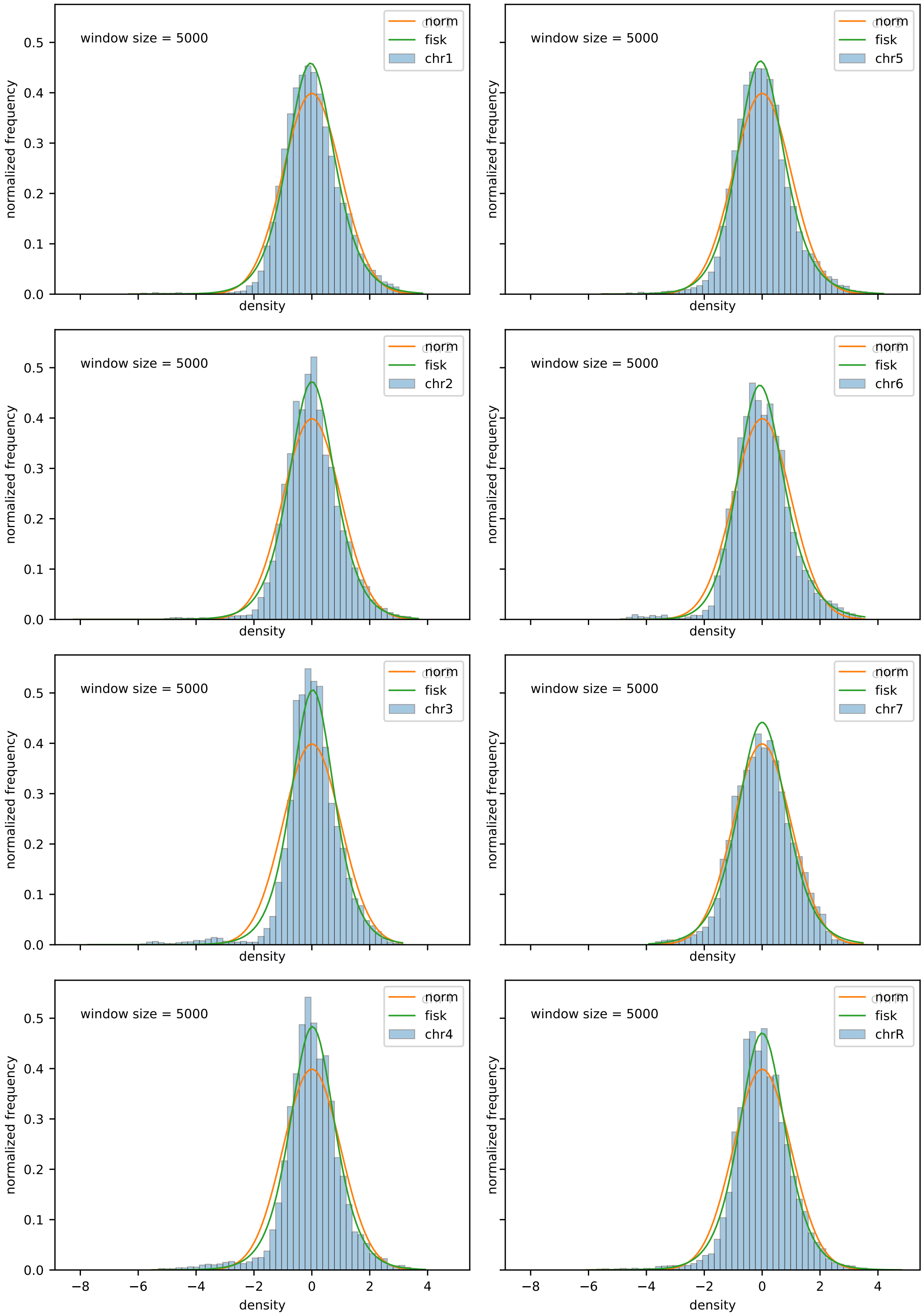

Figure S3: Normalized nucleosome density distributions for all of the chromosomes. The data shows the non-gaussian behavior (red line). For comparison a fit to a Log-logistic distribution is shown yielding a much better consistent fit. The bin size was 50 and the rolling average of size 5000 was used.

1.3 Distance Matrix for Individual Chromosomes

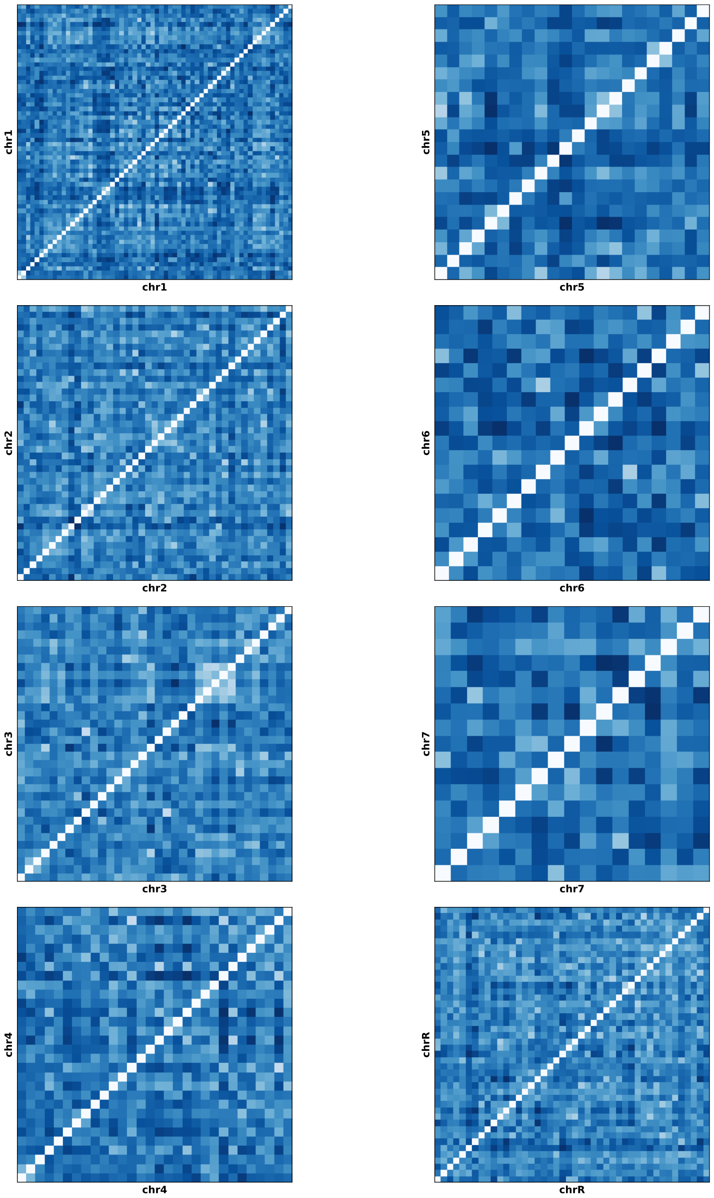

Figure S4: Shown are the distance matrices for all chromosomes. Distance refers to the distance between two correlation functions as measured by the euclidean distance (np.linalg.norm(x-y,ord=norm), with norm = 2. The ordering along the axes corresponds to the coarse-grained sections. The rolling average was of size 5000.

1.4 Clustering for Individual Chromosomes

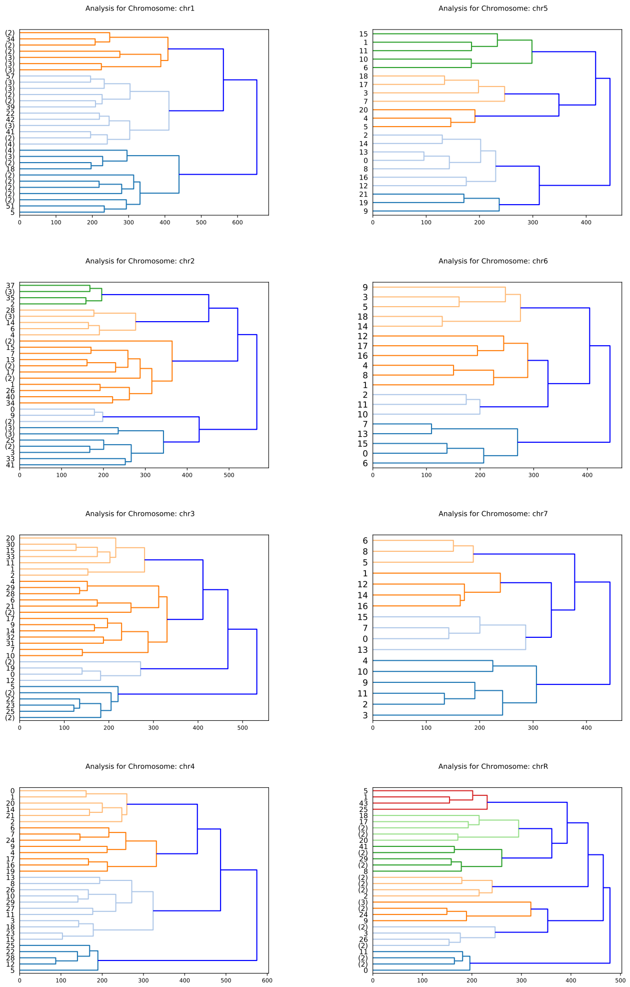

Figure S5: Shown are the dendrograms resulting from the distance matrices for all chromosomes. Results are for the hierarchical clustering on the individual chromosome. The Ward distance was used for the variance minimization algorithm used by SciPy **?**. The labels correspond to the distance matrix entries. Labels in parentheses give the number of labels corresponding to the leave. The rolling average was of size 5000. Labels in parentheses give the number of labels corresponding to the leaf.

1.5 Distance Matrix and Clustering for Individual Chromosomes

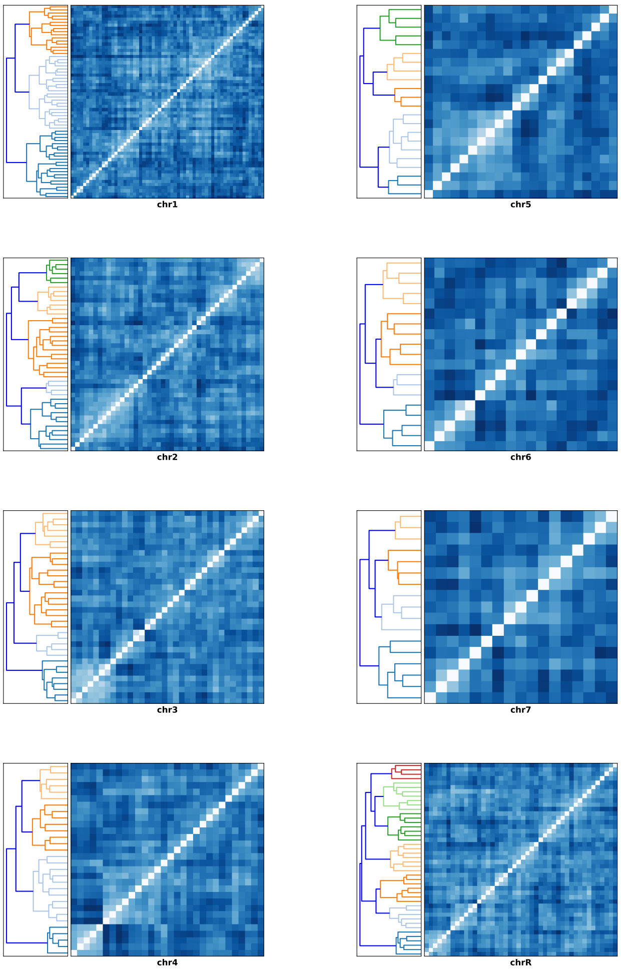

Figure S6: Shown are the distance matrices and corresponding dendrograms for all chromosomes. The matrix entries are sorted to correspond to the identified clusters. The rolling average was of size 5000.

1.6 Cluster Pattern in Chromosomes

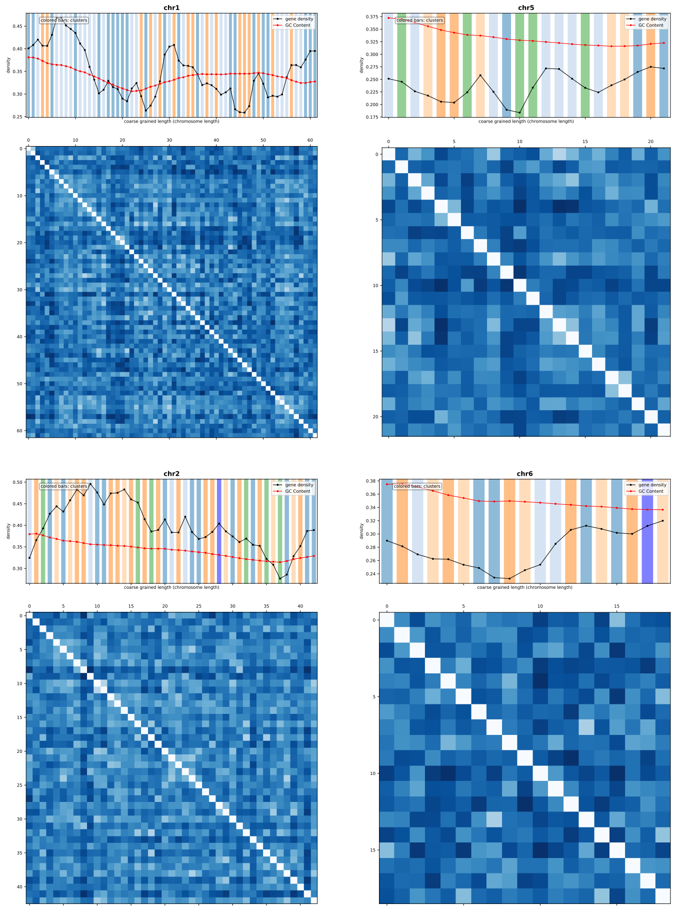

Figure S7: Part 1: Shown are the distance matrices and corresponding mapping of the pattern on the chromosomes. The matrix entries correspond to the positions on the chromosome. The rolling average was of size 5000.

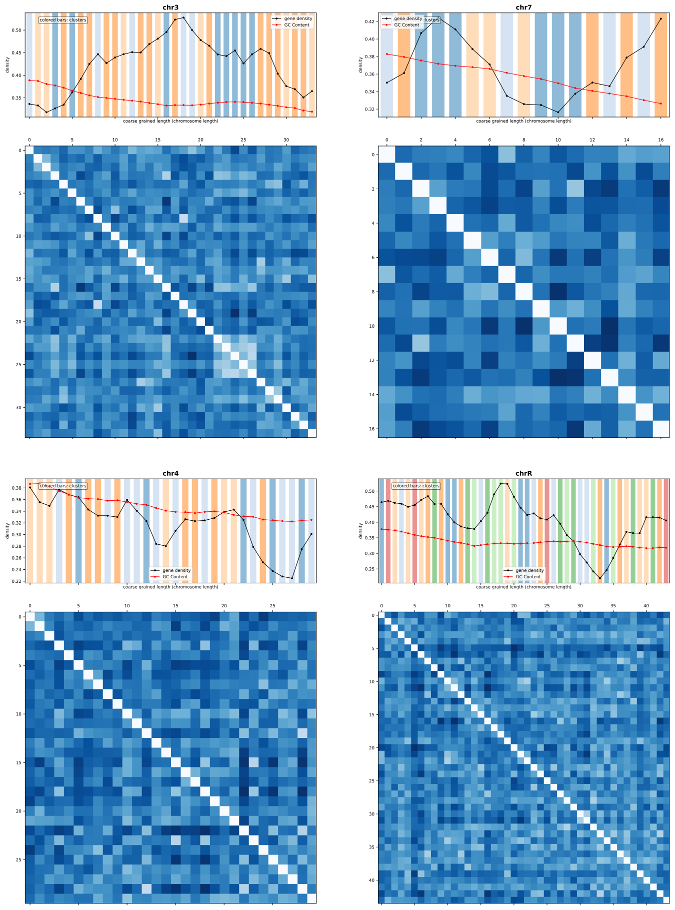

Figure S8: Part 2: Shown are the distance matrices and corresponding mapping of the pattern on the chromosomes. The matrix entries correspond to the positions on the chromosome. The rolling average was of size 5000.

1.7 Correspondence between Pattern and Correlation Function within individual Chromosome

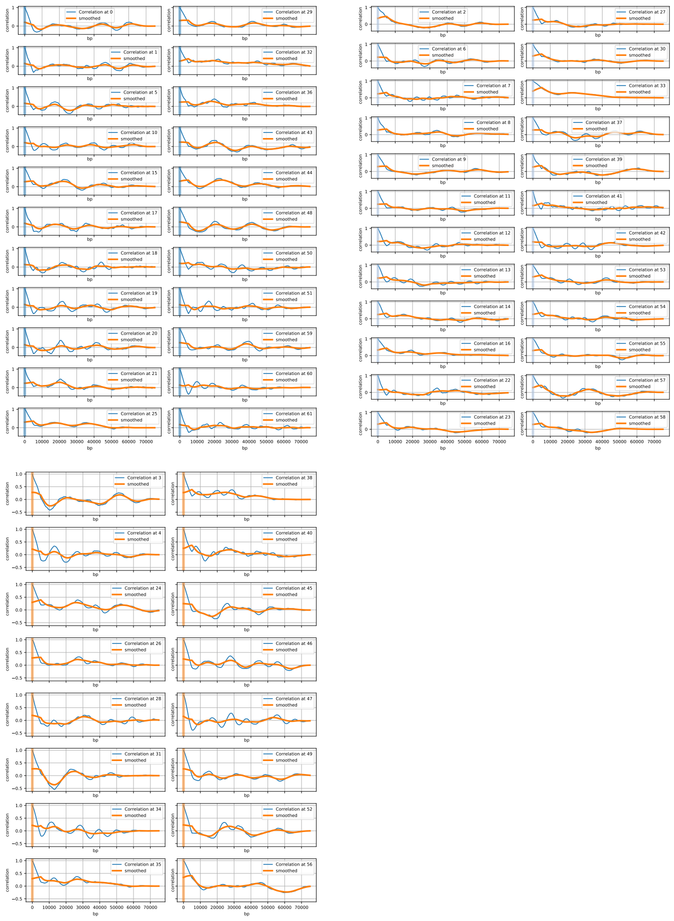

Figure S9: Shown are the correlation functions and the corresponding mapping of the pattern on the chromosome 1. The rolling average was of size 5000. The orange line marked "smoothed" is a smoothed representation of the correlation function (rolling average of size 10000 to highlight the feature commonality between the clustered correlation functions.

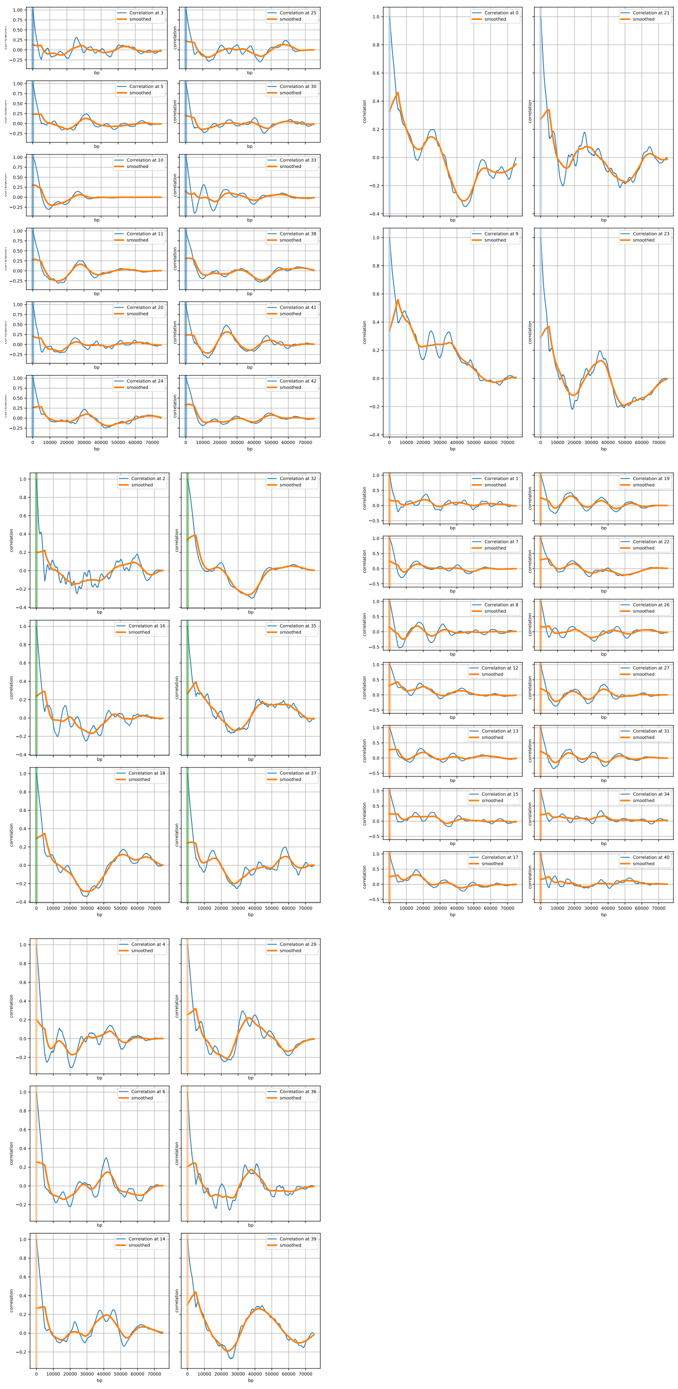

Figure S10: Shown are the correlation functions corresponding mapping of the pattern on the chromosome 2. The rolling average was of size 5000. The orange line marked "smoothed" is a smoothed representation of the correlation function (rolling average of size 10000 to highlight the feature commonality between the clustered correlation functions.

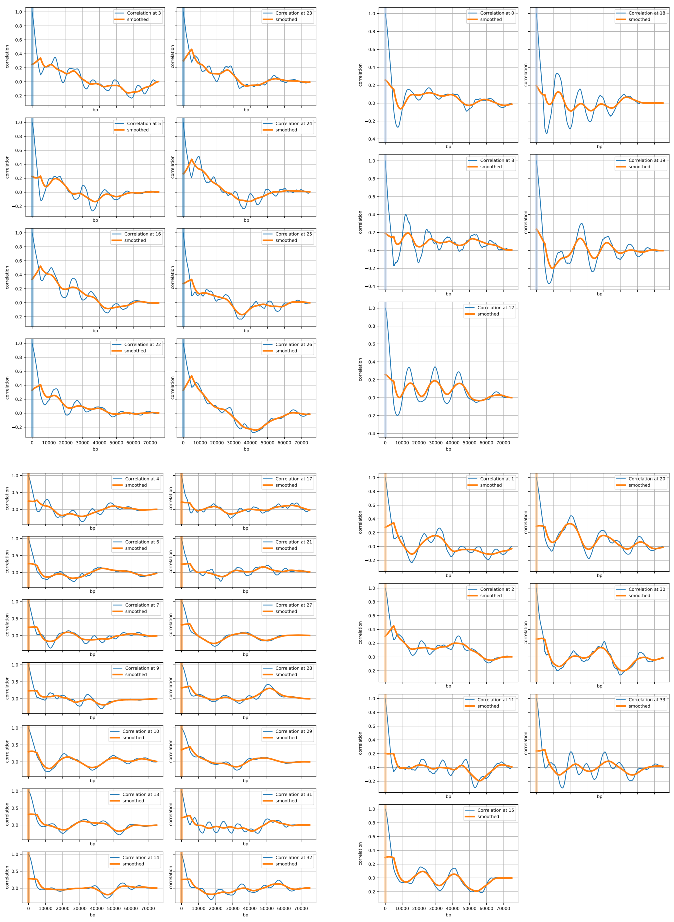

Figure S11: Shown are the correlation functions corresponding mapping of the pattern on the chromosome 3. The rolling average was of size 5000. The orange line marked "smoothed" is a smoothed representation of the correlation function (rolling average of size 10000 to highlight the feature commonality between the clustered correlation functions.

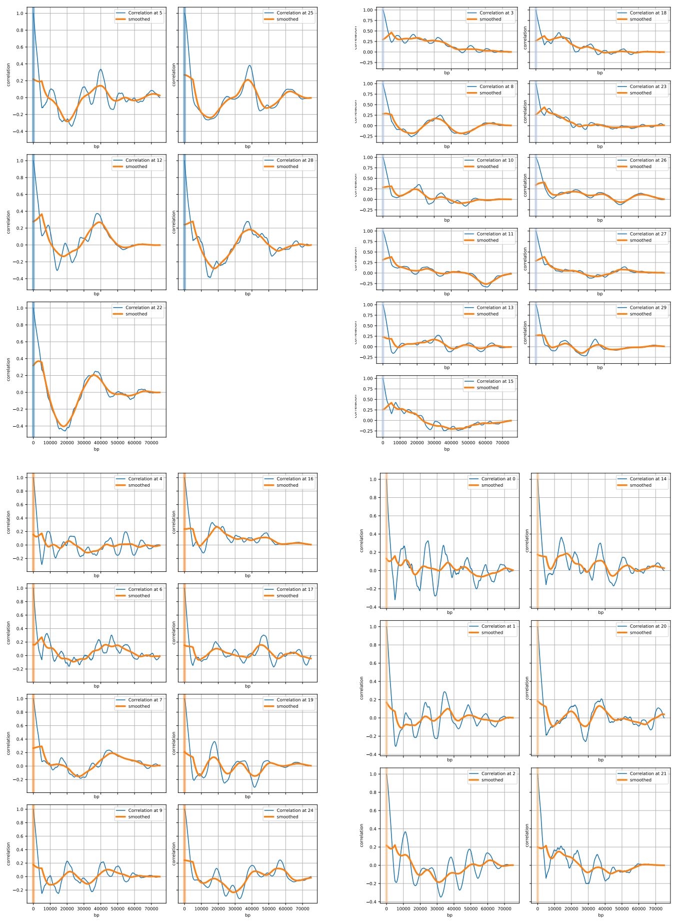

Figure S12: Shown are the correlation functions corresponding mapping of the pattern on the chromosome 4. The rolling average was of size 5000. The orange line marked "smoothed" is a smoothed representation of the correlation function (rolling average of size 10000 to highlight the feature commonality between the clustered correlation functions.

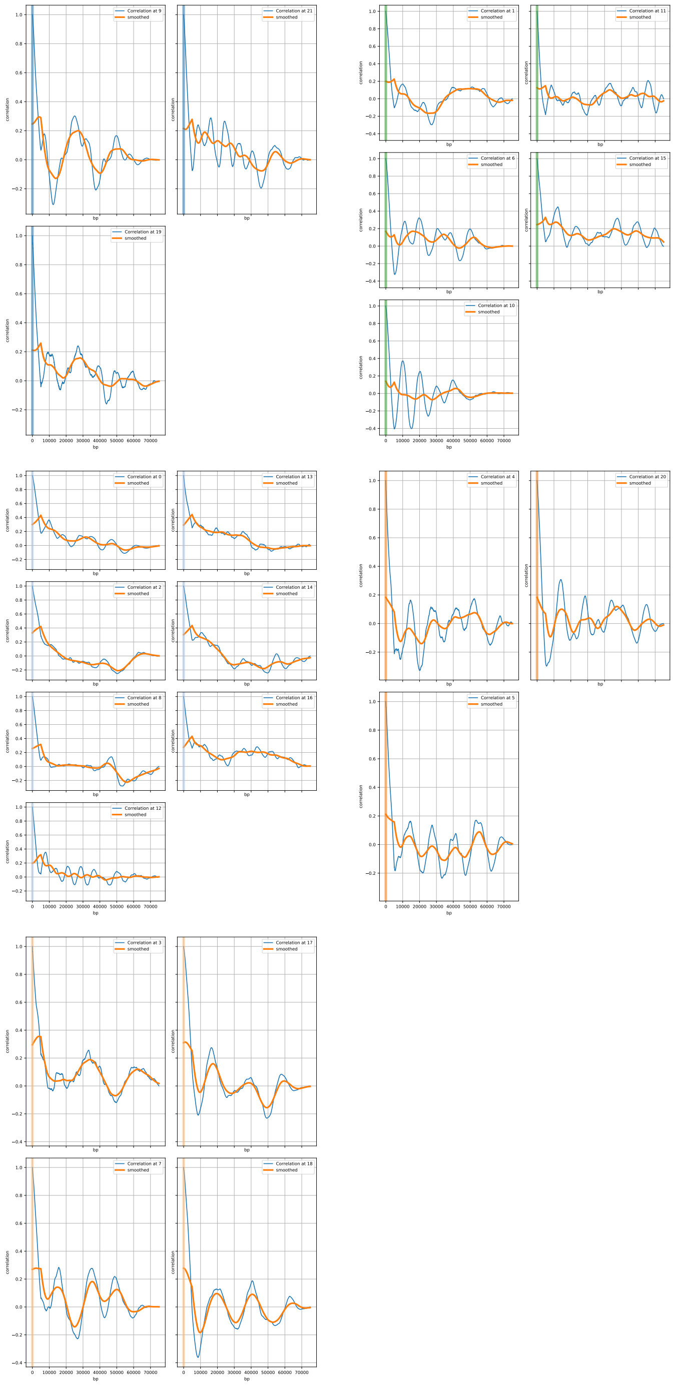

Figure S13: Shown are the correlation functions corresponding mapping of the pattern on the chromosome 5. The rolling average was of size 5000. The orange line marked "smoothed" is a smoothed representation of the correlation function (rolling average of size 10000 to highlight the feature commonality between the clustered correlation functions.

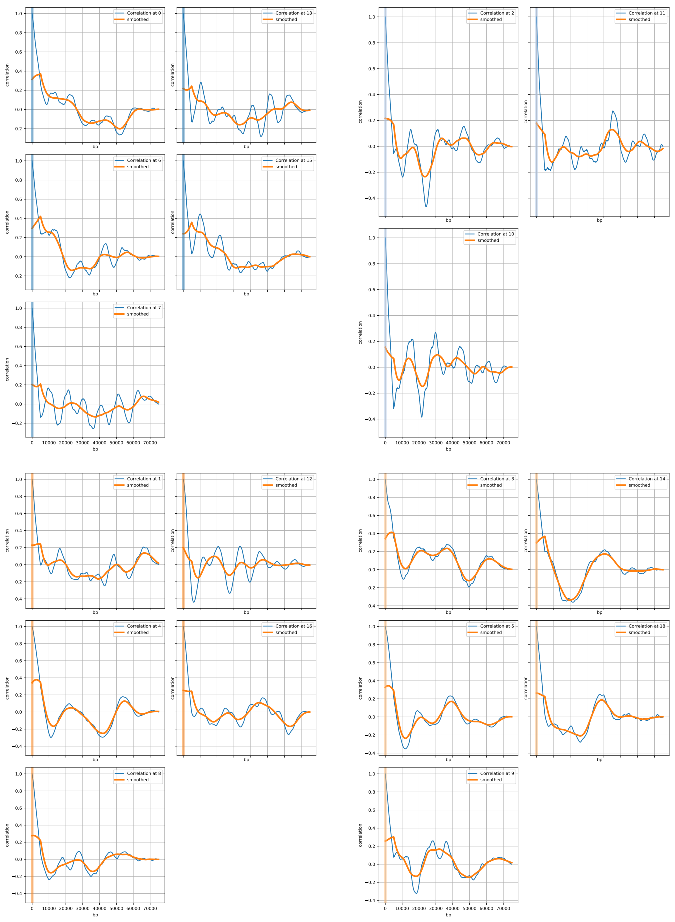

Figure S14: Shown are the correlation functions corresponding mapping of the pattern on the chromosome 6. The rolling average was of size 5000. The orange line marked "smoothed" is a smoothed representation of the correlation function (rolling average of size 10000 to highlight the feature commonality between the clustered correlation functions.

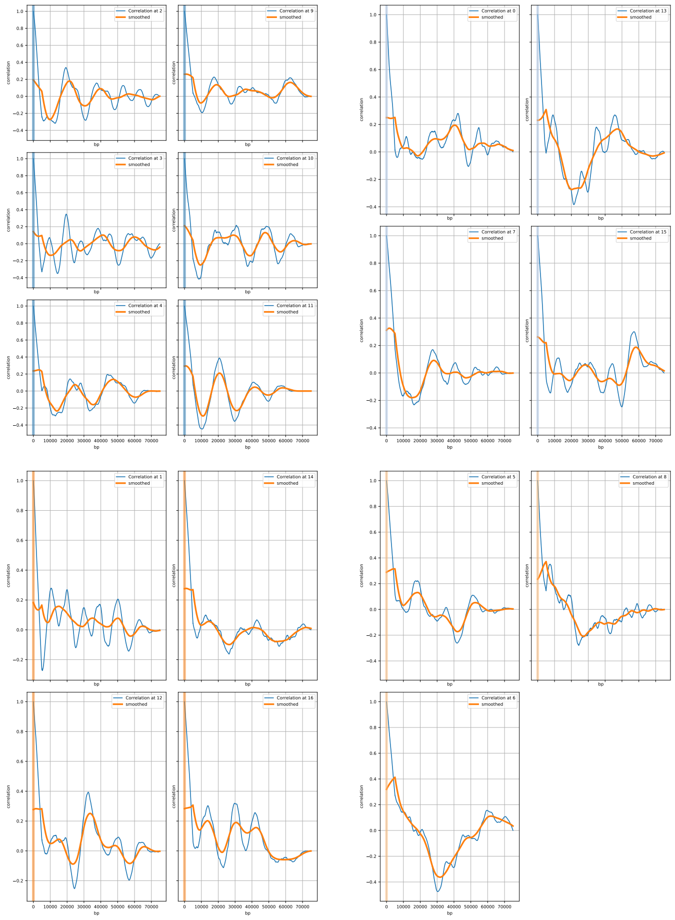

Figure S15: Shown are the correlation functions corresponding mapping of the pattern on the chromosome 7. The rolling average was of size 5000. The orange line marked "smoothed" is a smoothed representation of the correlation function (rolling average of size 10000 to highlight the feature commonality between the clustered correlation functions.

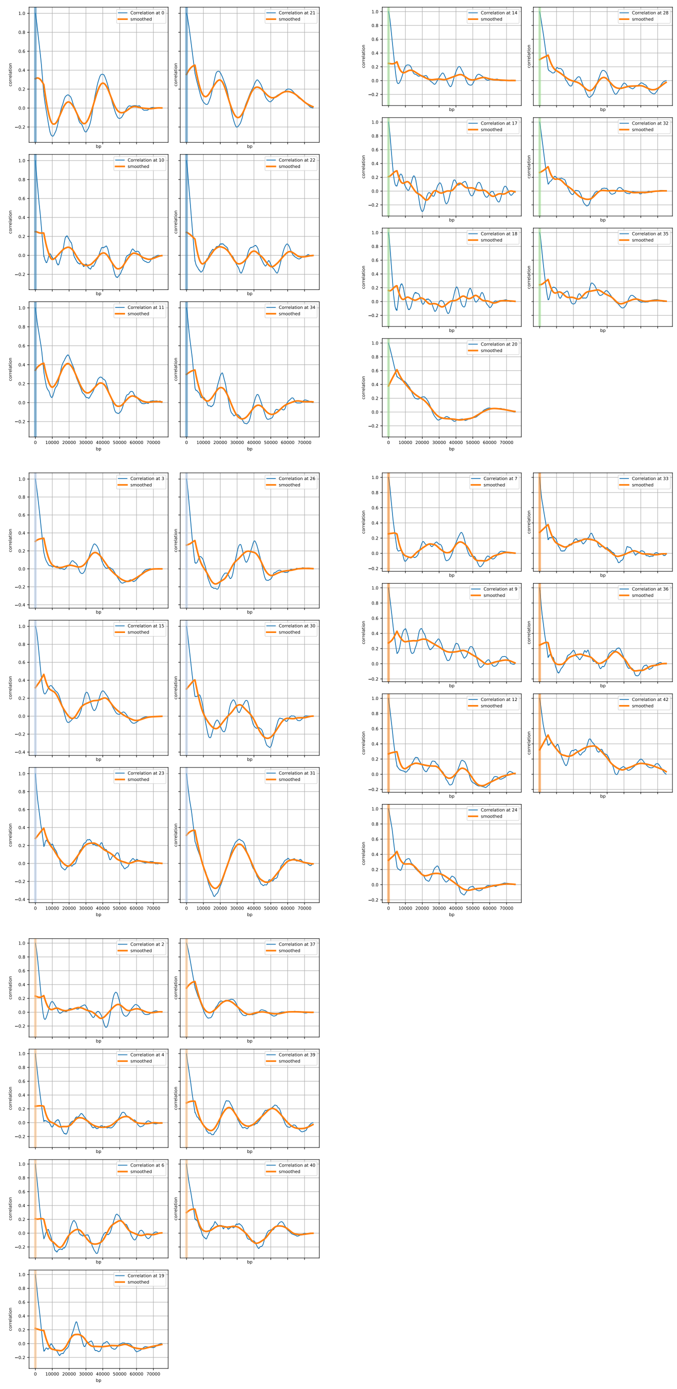

Figure S16: Shown are the correlation functions corresponding mapping of the pattern on the chromosome R. The rolling average was of size 5000. The orange line marked "smoothed" is a smoothed representation of the correlation function (rolling average of size 10000 to highlight the feature commonality between the clustered correlation functions.

1.8 Genome-Wide Distance Matrix

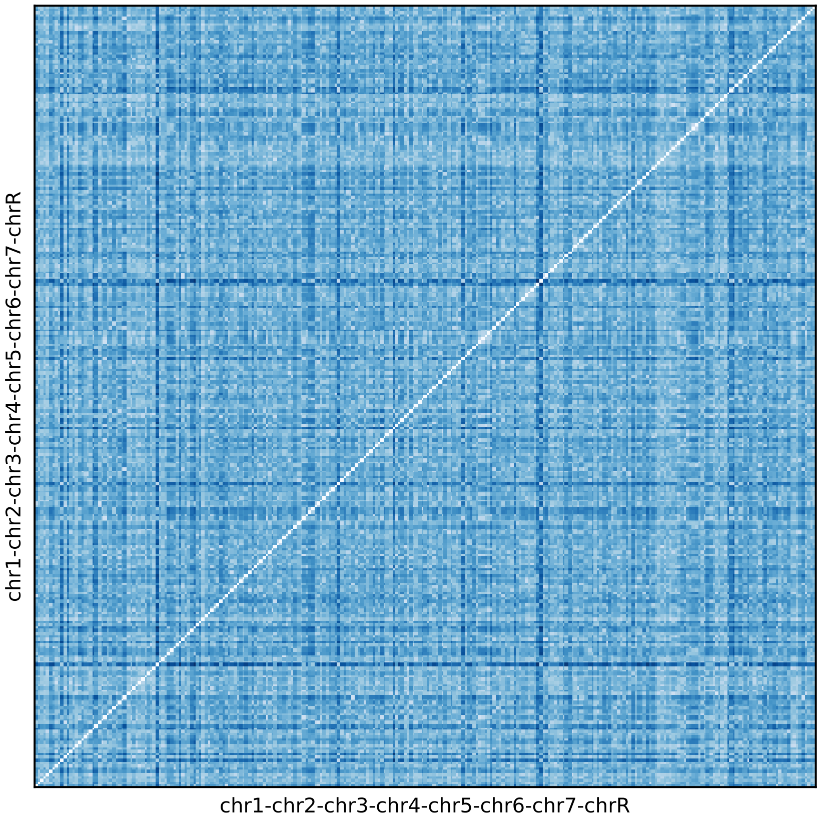

Figure S17: Shown is the genome-wide distance matrice. Distance refers to the distance between two correlation functions as measured by the euclidean distance (np.linalg.norm(x-y,ord=norm), with norm = 2. The ordering along the axes corresponds to the coarse-grained sections. The rolling average was of size 5000.

1.9 Genome-Wide Clustering

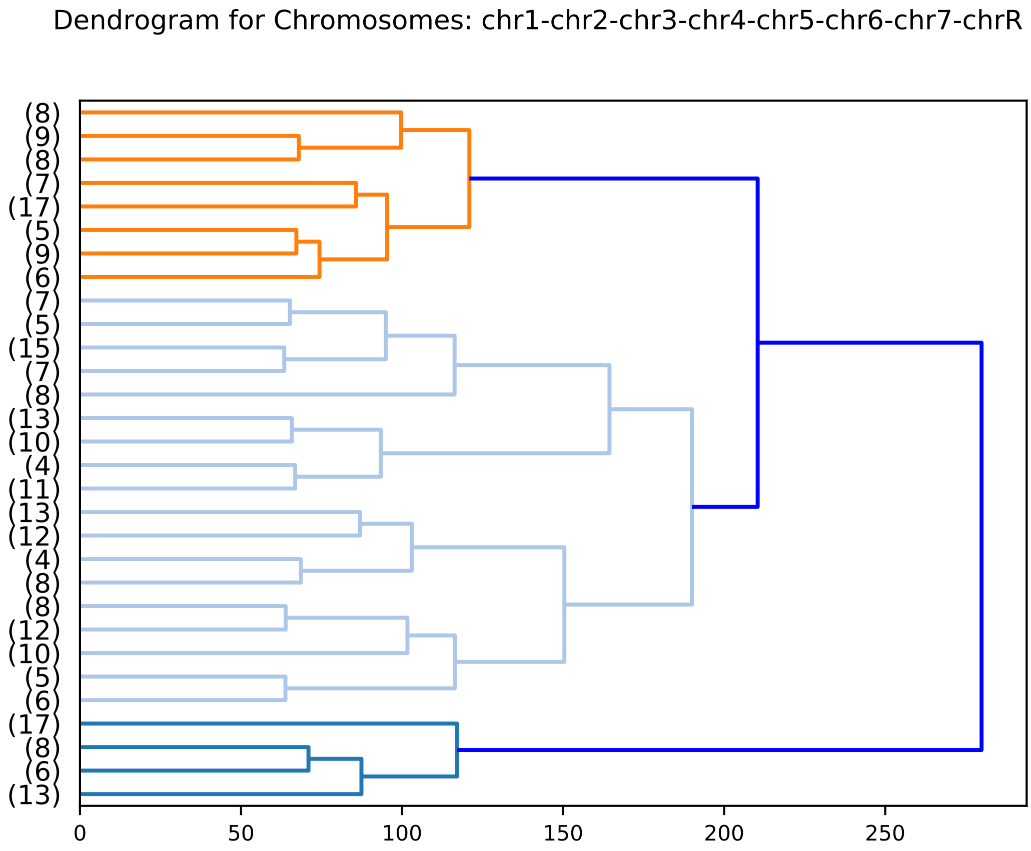

Figure S18: Shown is the dendrograms resulting from the genome-wide distance matrix. Results are for the hierarchical clustering on the individual chromosome. The Ward distance was used for the variance minimization algorithm used by SciPy **?**. The labels correspond to the distance matrix entries. Labels in parentheses give the number of labels corresponding to the leave. The rolling average was of size 5000.

1.10 Genome-Wide Distance Matrix and Clustering

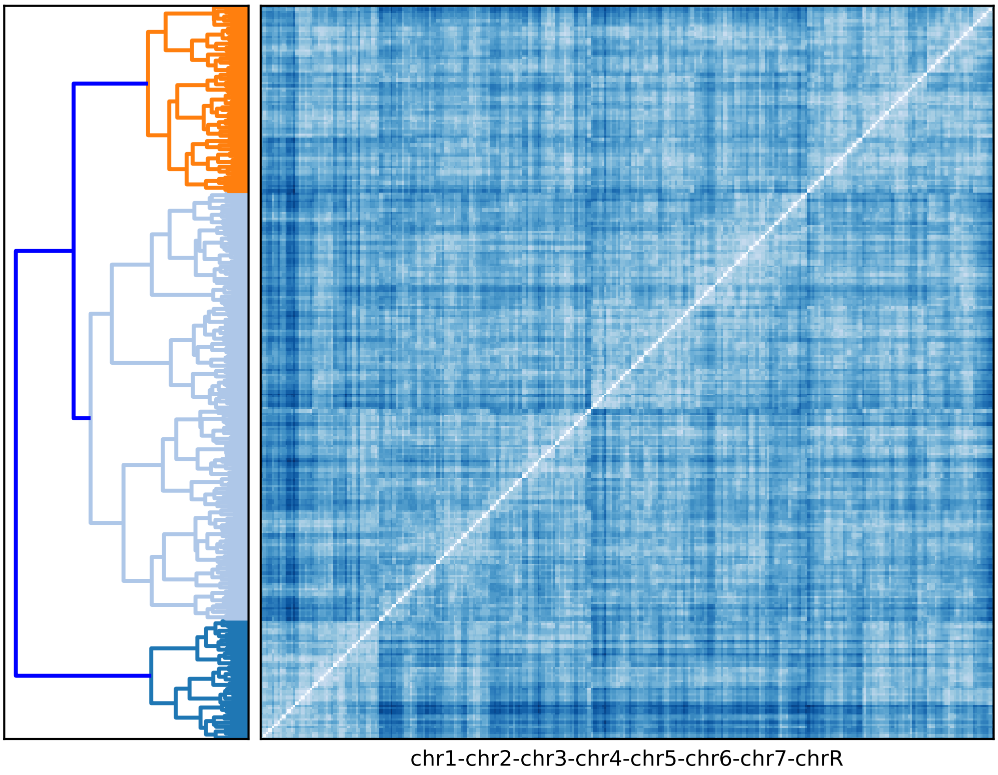

Figure S19: Shown is the genome-wide distance matrix and the corresponding dendrogram. The matrix entries are sorted to correspond to the identified clusters. The rolling average was of size 5000.

1.11 Correspondence between Pattern and Correlation Function Genome-Wide

Correlation Function corresponding to the Pattern Genome-Wide Pattern No. 1

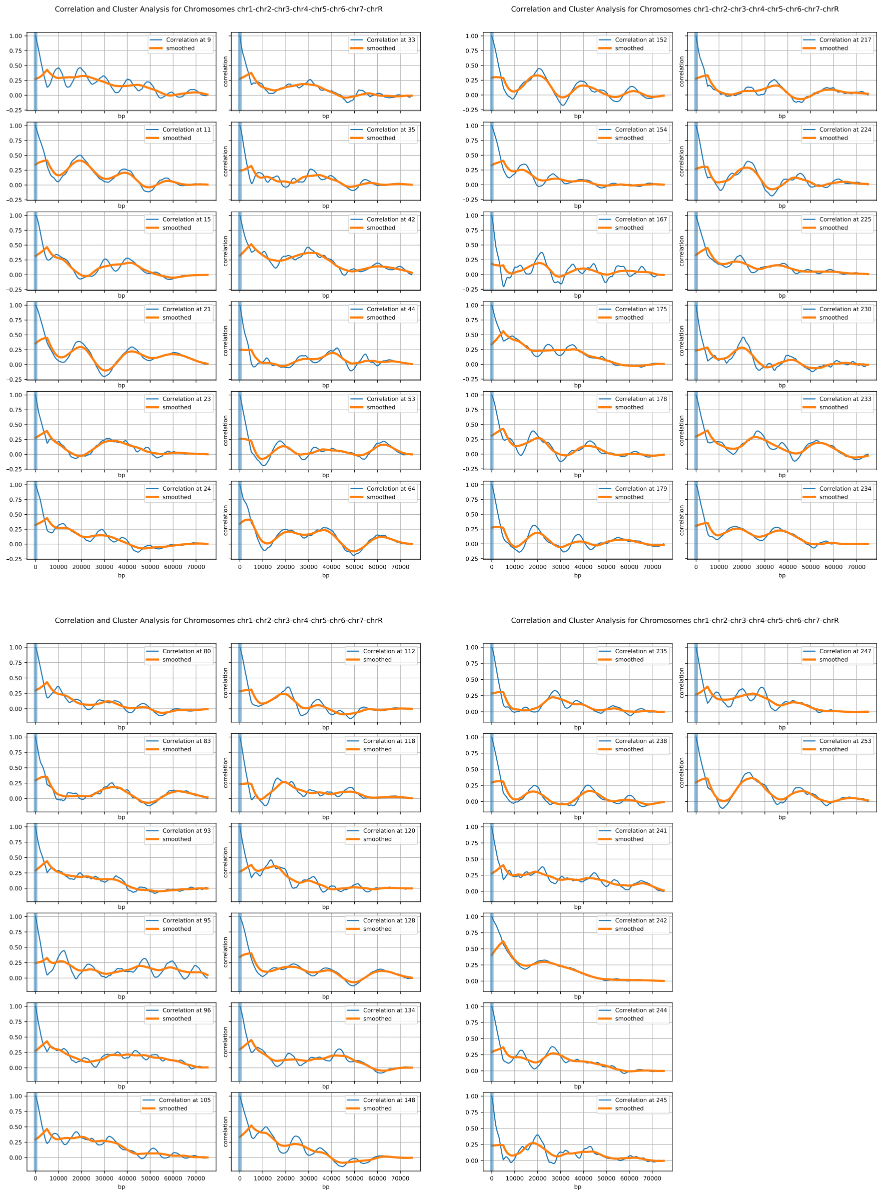

Figure S20: Shown are the correlation functions corresponding mapping of the pattern on the chromosomes. The rolling average was of size 5000. The orange line marked "smoothed" is a smoothed representation of the correlation function (rolling average of size 10000 to highlight the feature commonality between the clustered correlation functions.

Correlation Function corresponding to the Pattern Genome-Wide Pattern No. 2 (1)

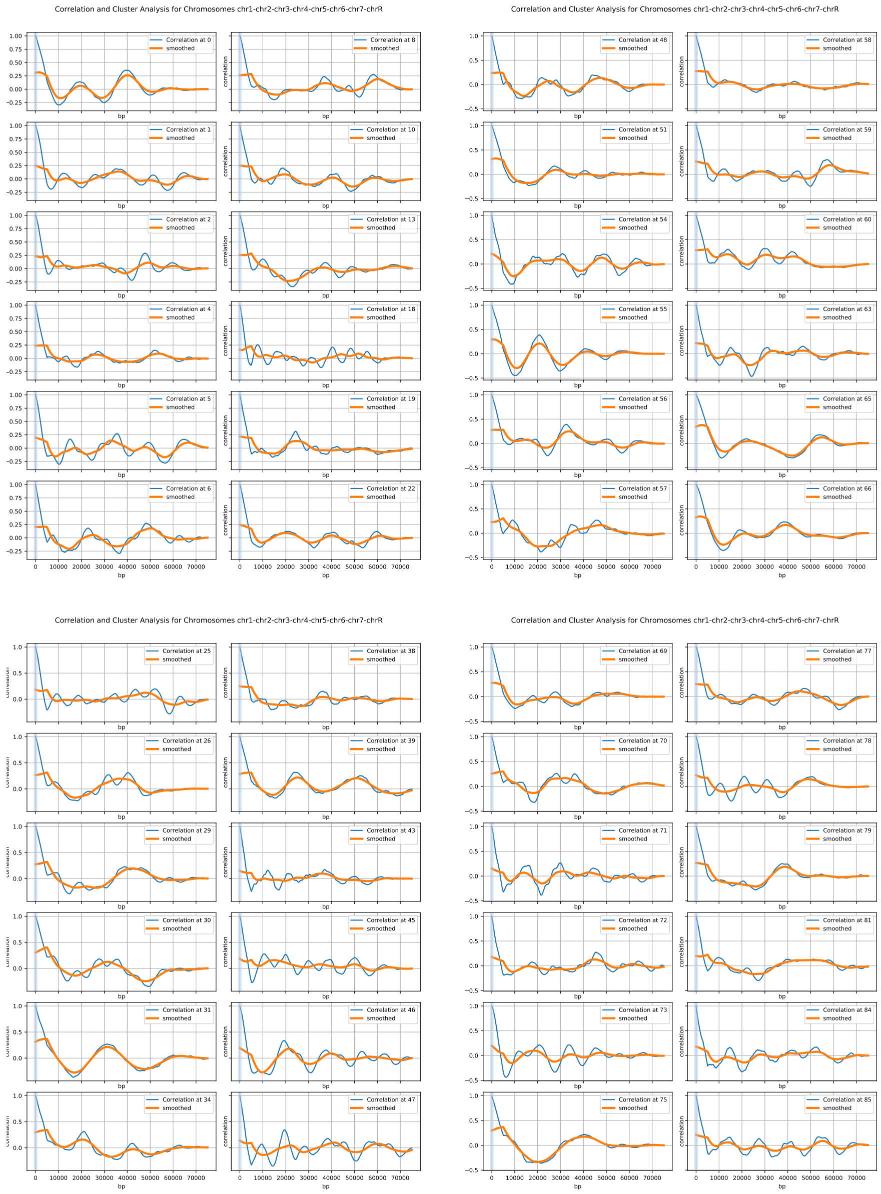

Figure S21: Shown are the correlation functions corresponding mapping of the pattern on the chromosomes. The rolling average was of size 5000. The orange line marked "smoothed" is a smoothed representation of the correlation function (rolling average of size 10000 to highlight the feature commonality between the clustered correlation functions.

Correlation Function corresponding to the Pattern Genome-Wide Pattern No. 2 (2)

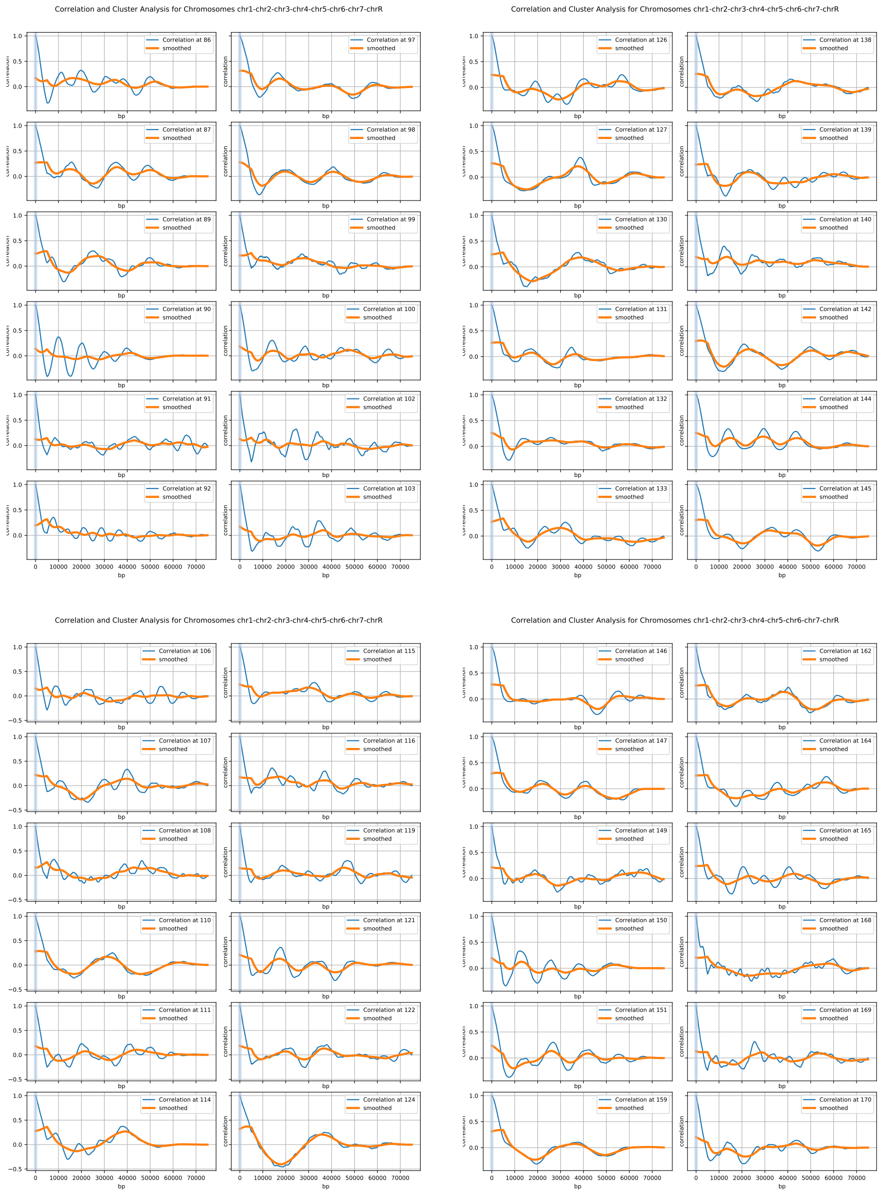

Figure S22: Shown are the correlation functions corresponding mapping of the pattern on the chromosomes. The rolling average was of size 5000. The orange line marked "smoothed" is a smoothed representation of the correlation function (rolling average of size 10000 to highlight the feature commonality between the clustered correlation functions.

Correlation Function corresponding to the Pattern Genome-Wide Pattern No. 2 (3)

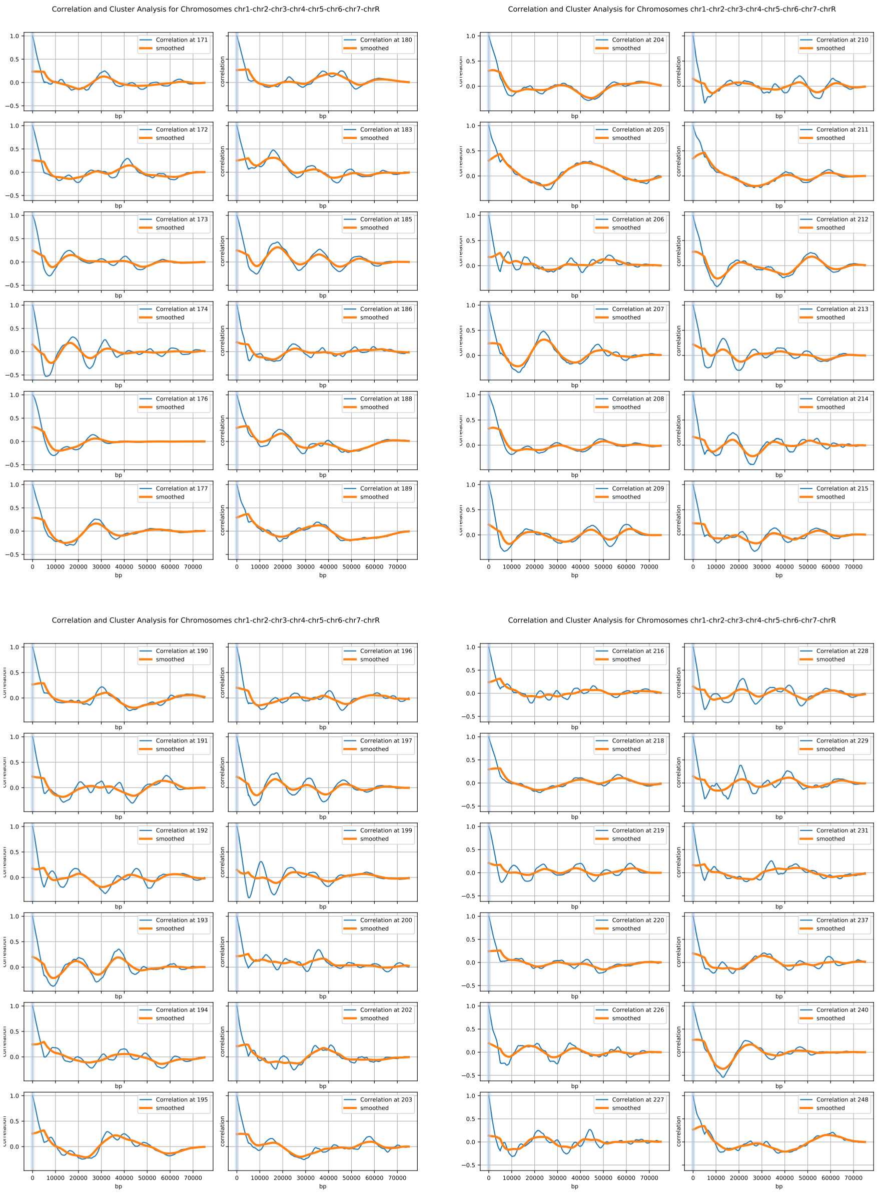

Figure S23: Shown are the correlation functions corresponding mapping of the pattern on the chromosomes. The rolling average was of size 5000. The orange line marked "smoothed" is a smoothed representation of the correlation function (rolling average of size 10000 to highlight the feature commonality between the clustered correlation functions.

Correlation Function corresponding to the Pattern Genome-Wide Pattern No. 2 (4)

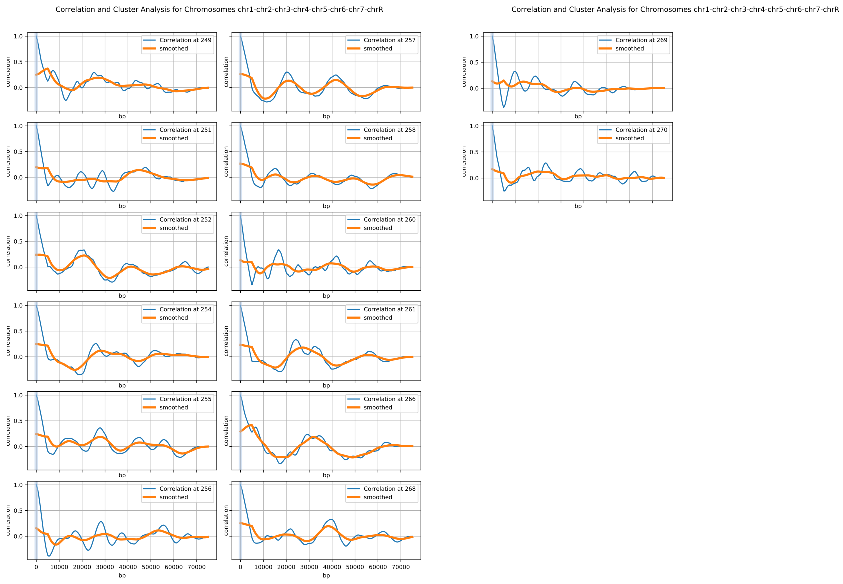

Figure S24: Shown are the correlation functions corresponding mapping of the pattern on the chromosomes. The rolling average was of size 5000. The orange line marked "smoothed" is a smoothed representation of the correlation function (rolling average of size 10000 to highlight the feature commonality between the clustered correlation functions.

Correlation Function corresponding to the Pattern Genome-Wide Pattern No. 3 (1)

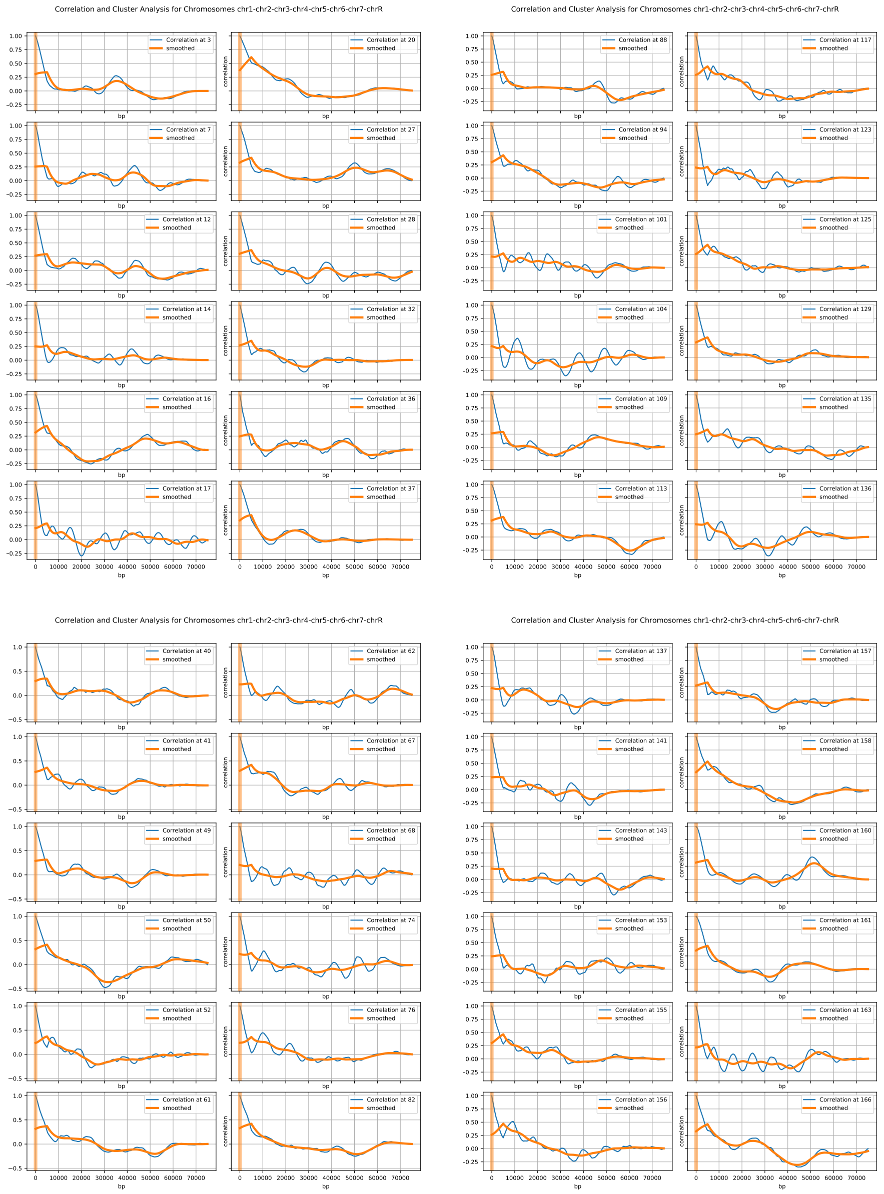

Figure S25: Shown are the correlation functions corresponding mapping of the pattern on the chromosomes. The rolling average was of size 5000. The orange line marked "smoothed" is a smoothed representation of the correlation function (rolling average of size 10000 to highlight the feature commonality between the clustered correlation functions.

Correlation Function corresponding to the Pattern Genome-Wide Pattern No. 3 (2)

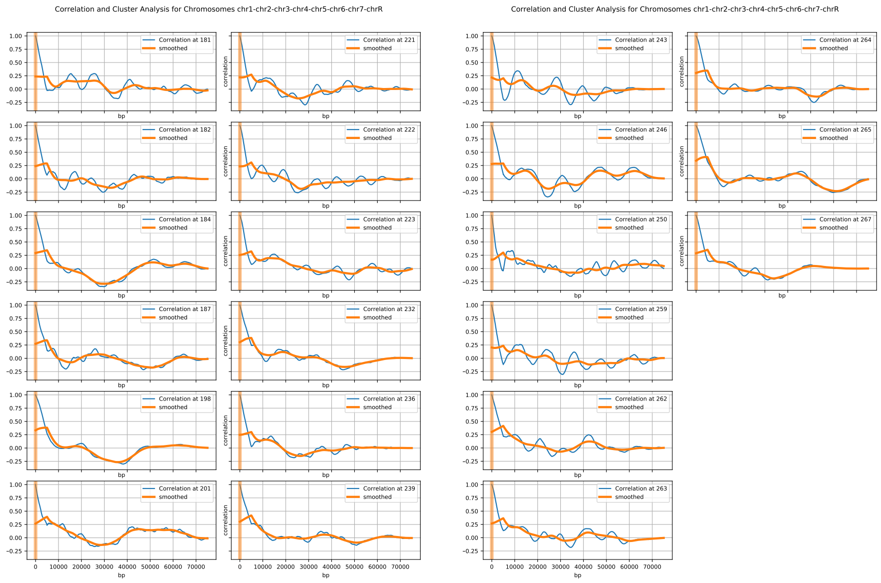

Figure S26: Shown are the correlation functions corresponding mapping of the pattern on the chromosomes. The rolling average was of size 5000. The orange line marked "smoothed" is a smoothed representation of the correlation function (rolling average of size 10000 to highlight the feature commonality between the clustered correlation functions.

1.12 Comparison of Different Metrics

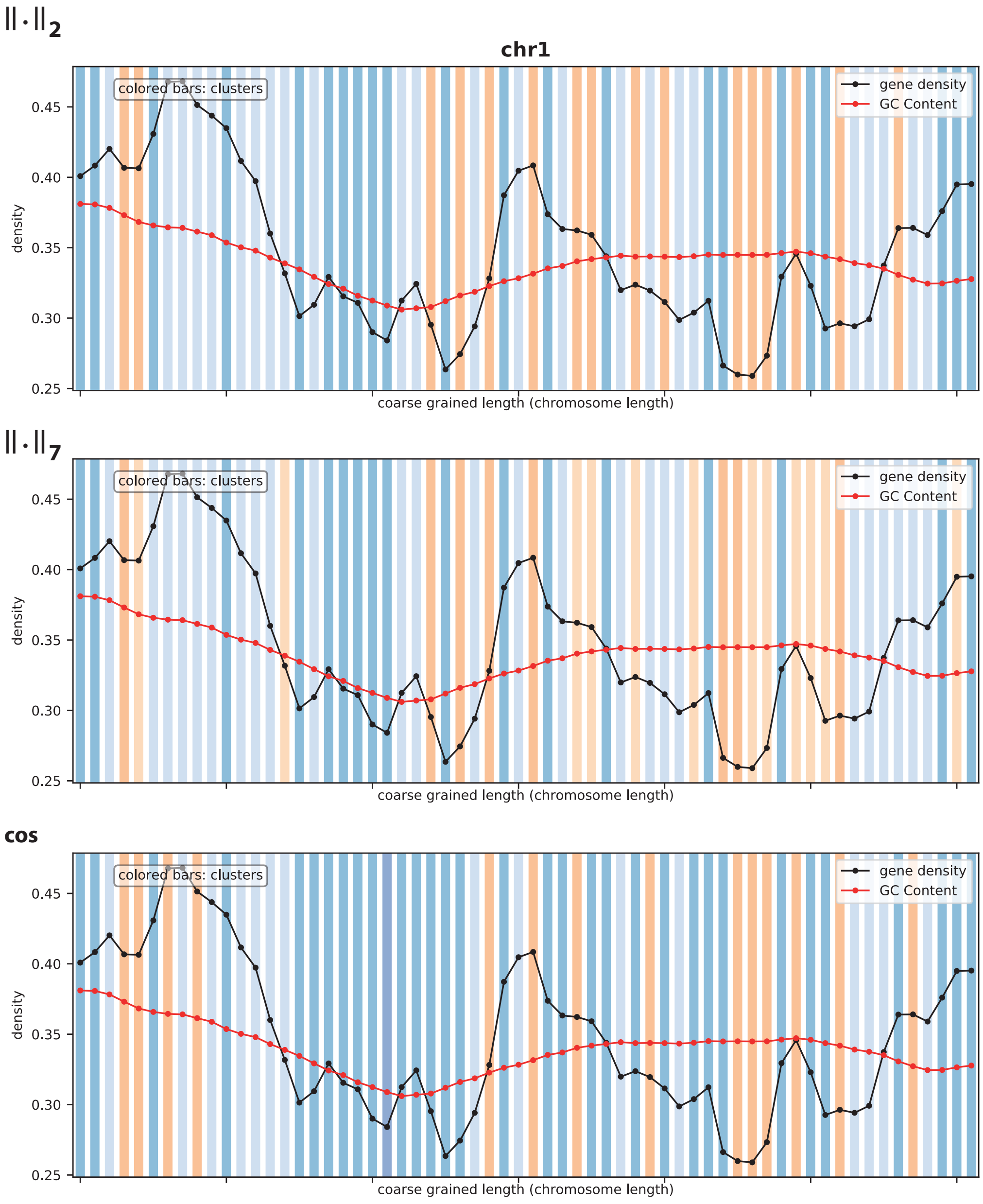

Figure S27: The upper panel shows the classification of the structures with respect to the euclidean distance $\|.\|_2$ while the middle one shows the result for $\|.\|_7$. Note that $\|.\|_7$ shows a further subdivision of the orange colored regions. Otherwise, the structure is stable against the two metrics for the distance between two correlation functions. The black line shows the gene density and the red line the GC content. The lower panel shows the application of the cosine similarity measure. While there are differences between the different metric, overall, a stable pattern is observed. What is remarkable is that the similarity measure shows less variation within certain domains than the other measures.
