# Supplementary figures and images for: Superstructure Detection in Nucleosome Distribution Shows Common Pattern within a Chromosome and within the Genome (part 2 of 2)
Source: Life (Basel). 2022 Apr 6;12(4):541. doi: 10.3390/life12040541 (PMC9026121; doi:10.3390/life12040541)

**chrR**

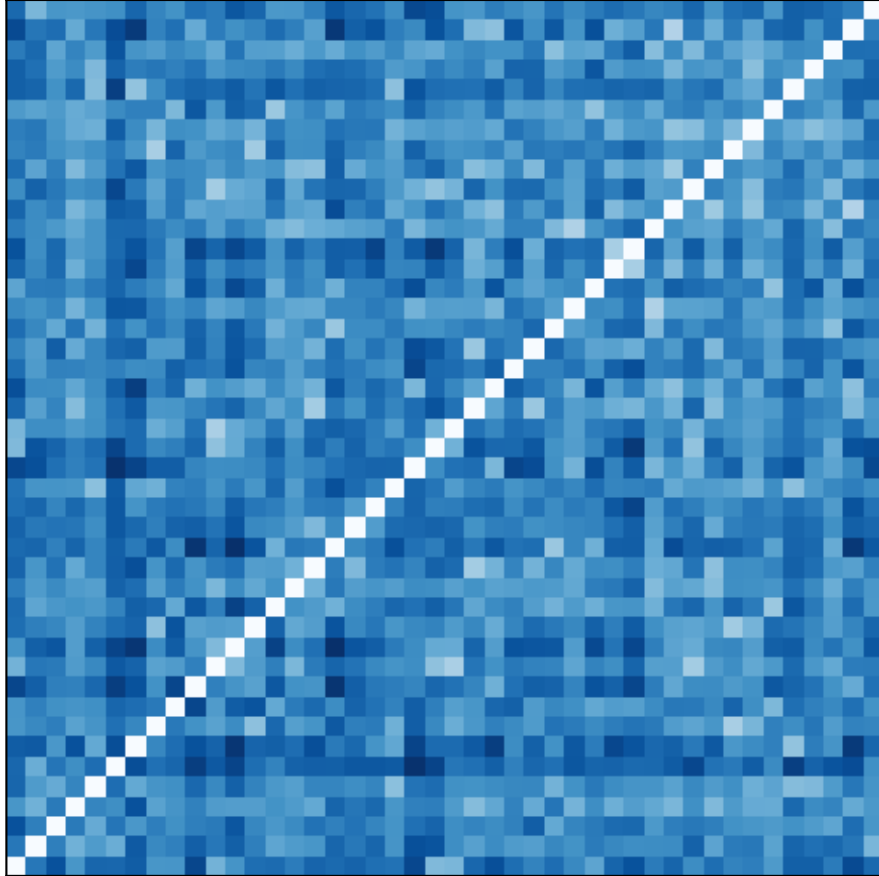

**chrR**

Supplement: Supplementary file 1 [file life-12-00541-s001.zip › life-1592845-supplementary/Heermann-distance-matrix-chrR.pdf]

# Gene Density Analysis: Ca22chr1A

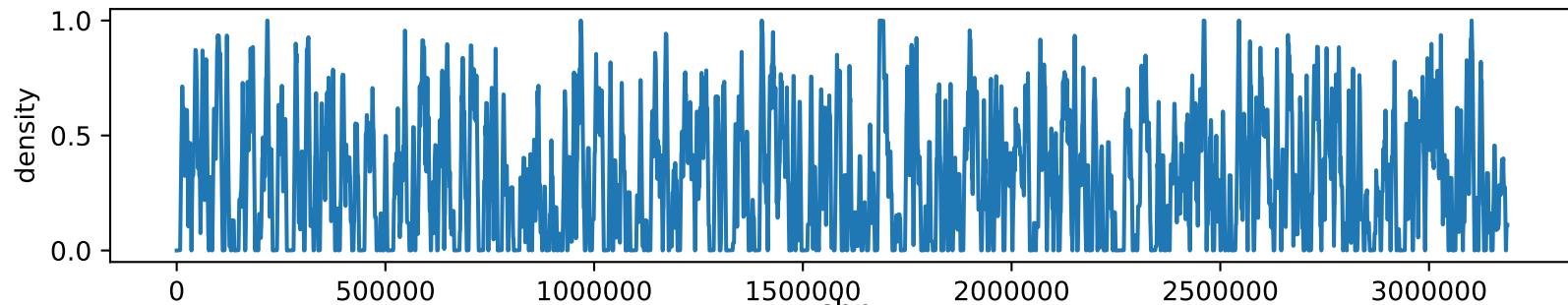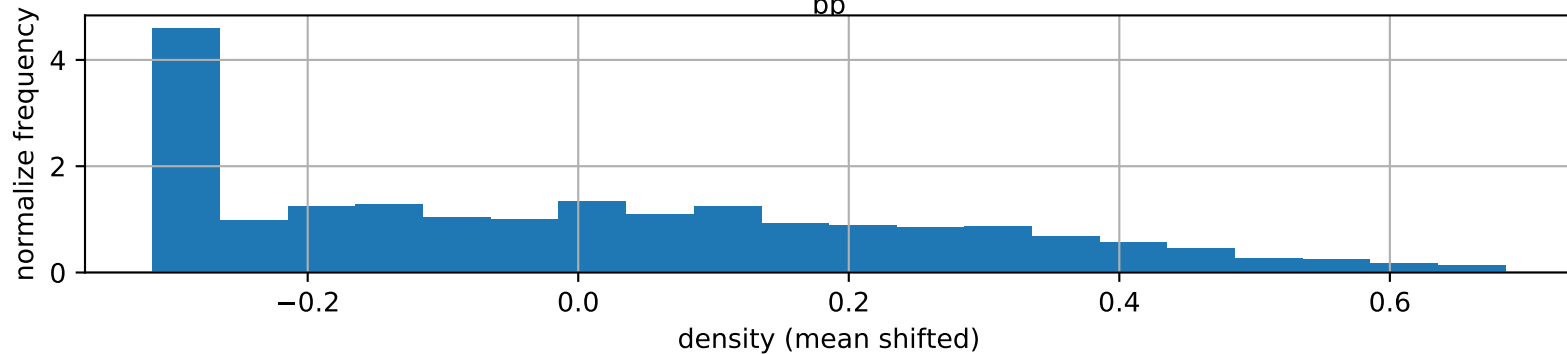

Supplement: Supplementary file 1 [file life-12-00541-s001.zip › life-1592845-supplementary/Heermann-gene-density.pdf]

$\|\cdot\|_2$ 

chr1

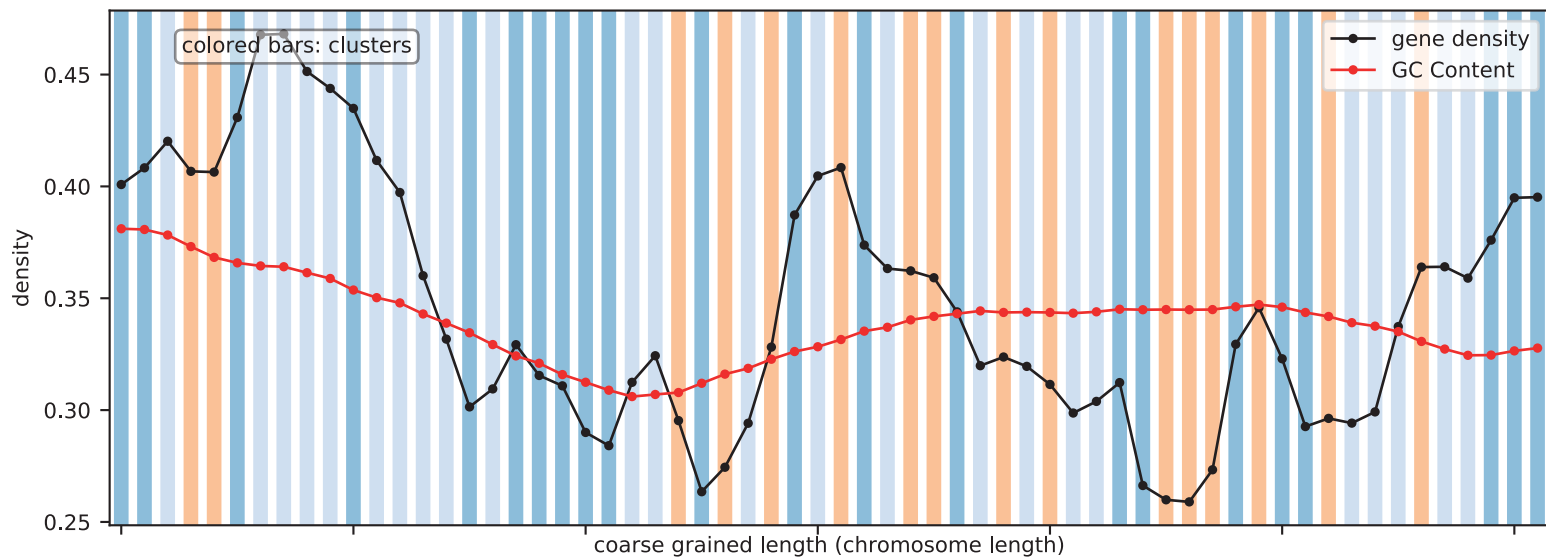 $\|\cdot\|_7$ 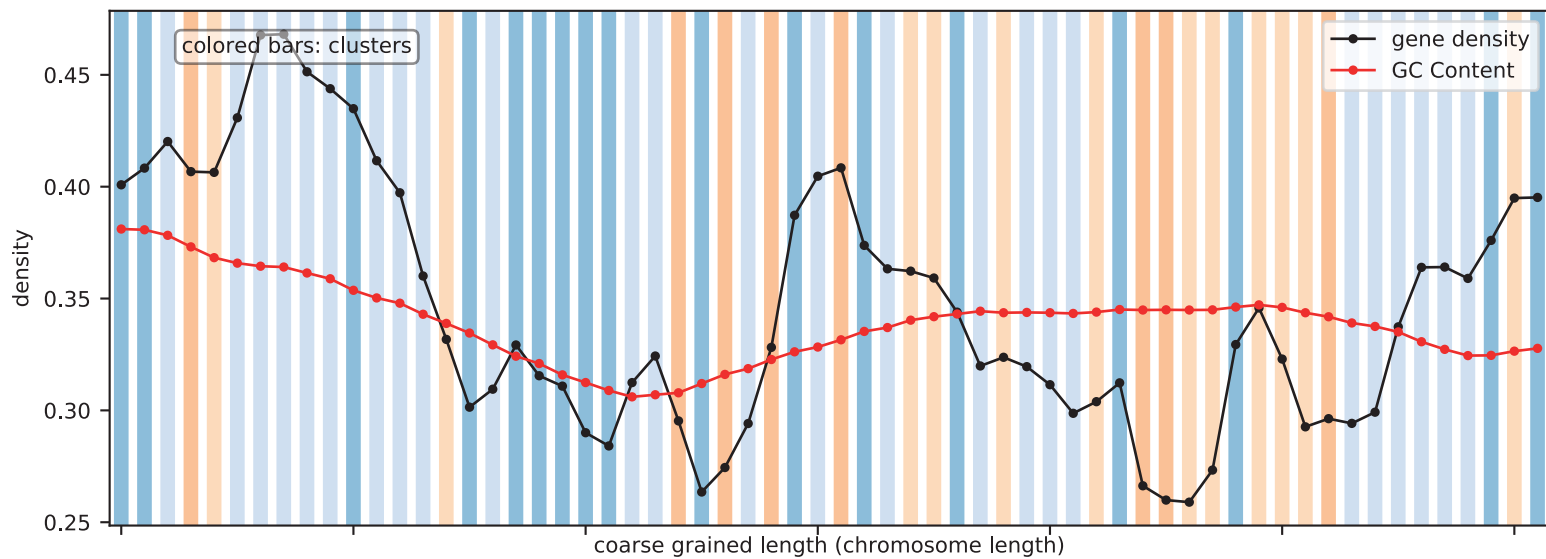

Supplement: Supplementary file 1 [file life-12-00541-s001.zip › life-1592845-supplementary/Heermann-Norm-Comparison.pdf]

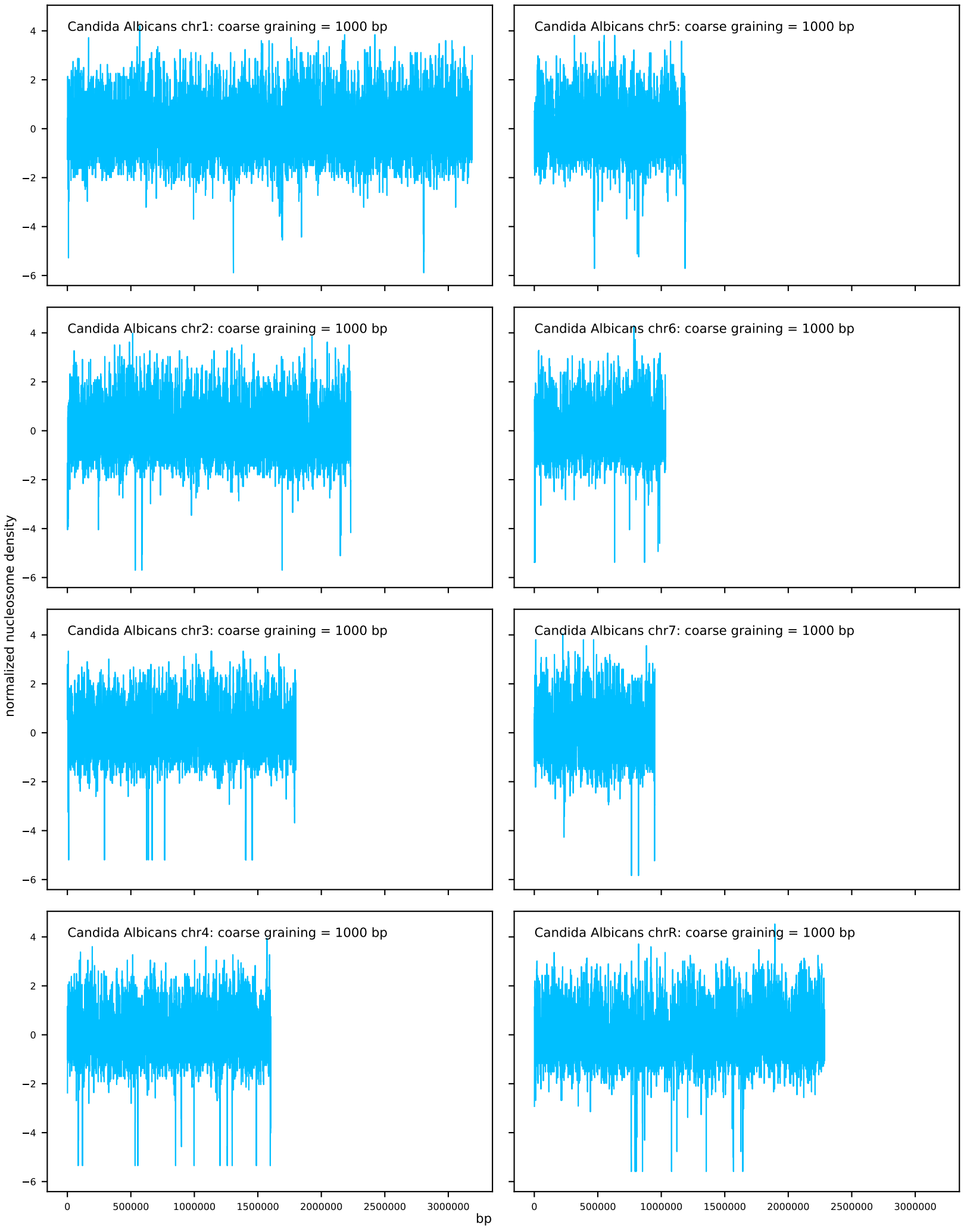

Supplement: Supplementary file 1 [file life-12-00541-s001.zip › life-1592845-supplementary/Heermann-nuc-density-1000-all.pdf]

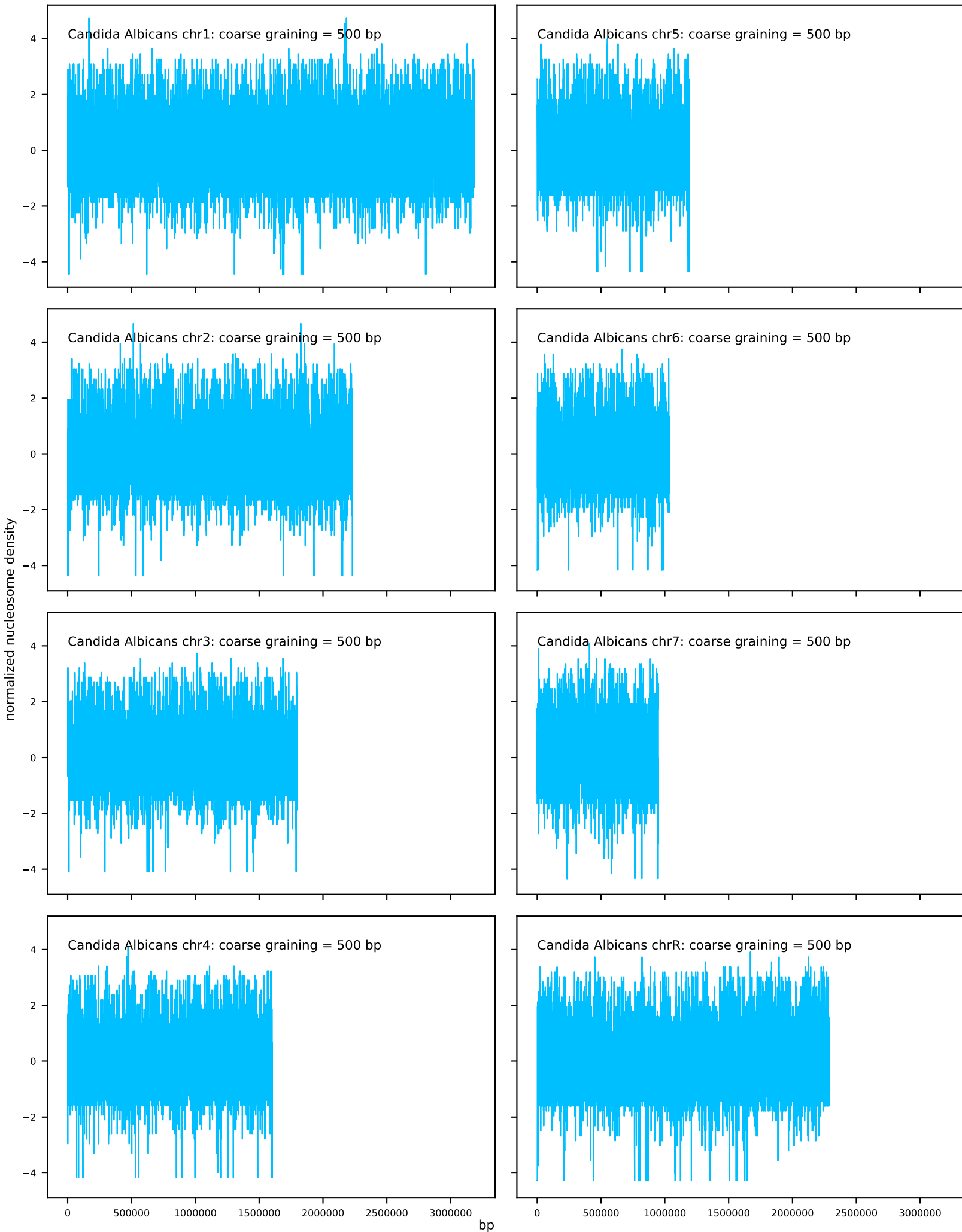

Supplement: Supplementary file 1 [file life-12-00541-s001.zip › life-1592845-supplementary/Heermann-nuc-density-500-all.pdf]

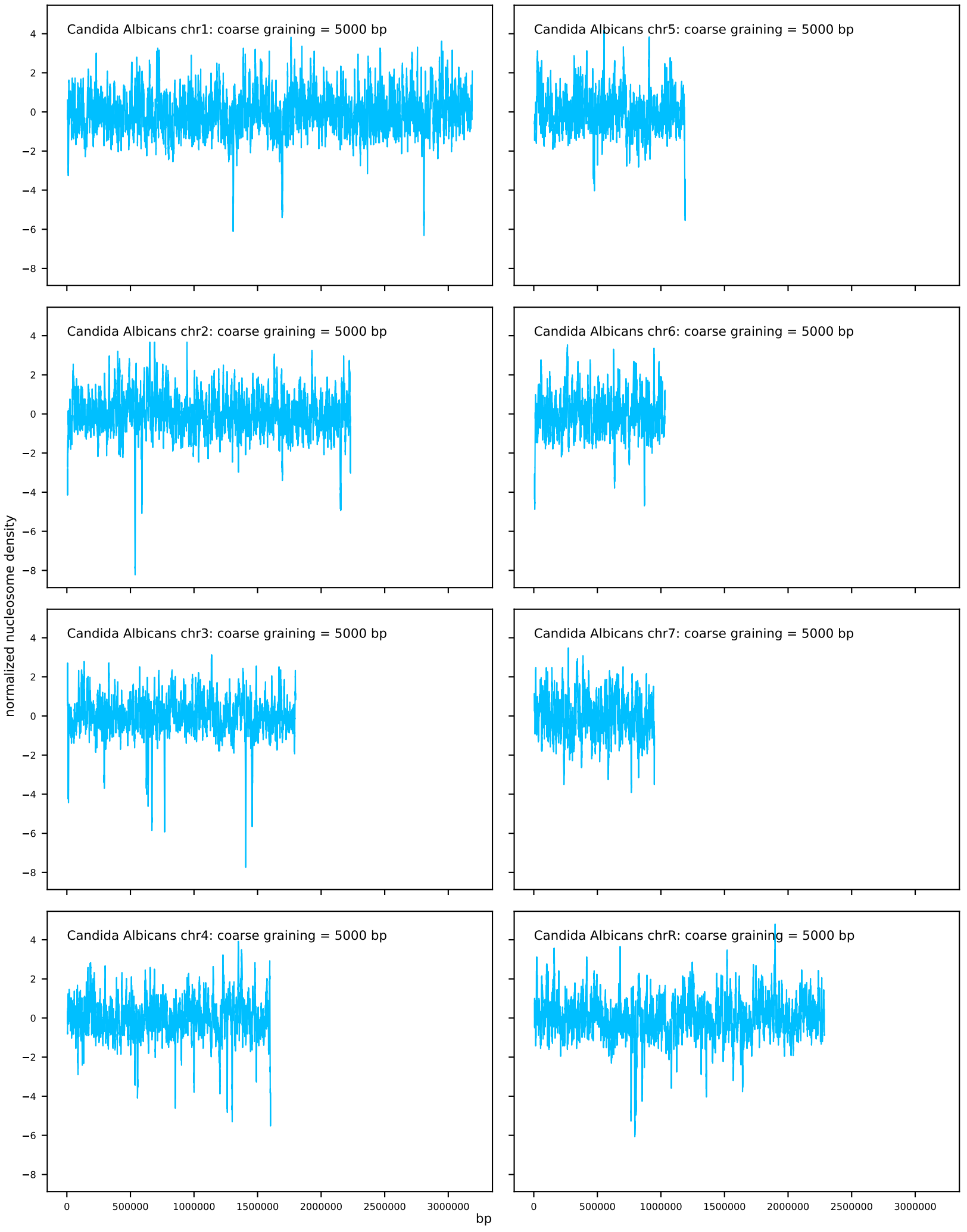

Supplement: Supplementary file 1 [file life-12-00541-s001.zip › life-1592845-supplementary/Heermann-nuc-density-5000-all.pdf]

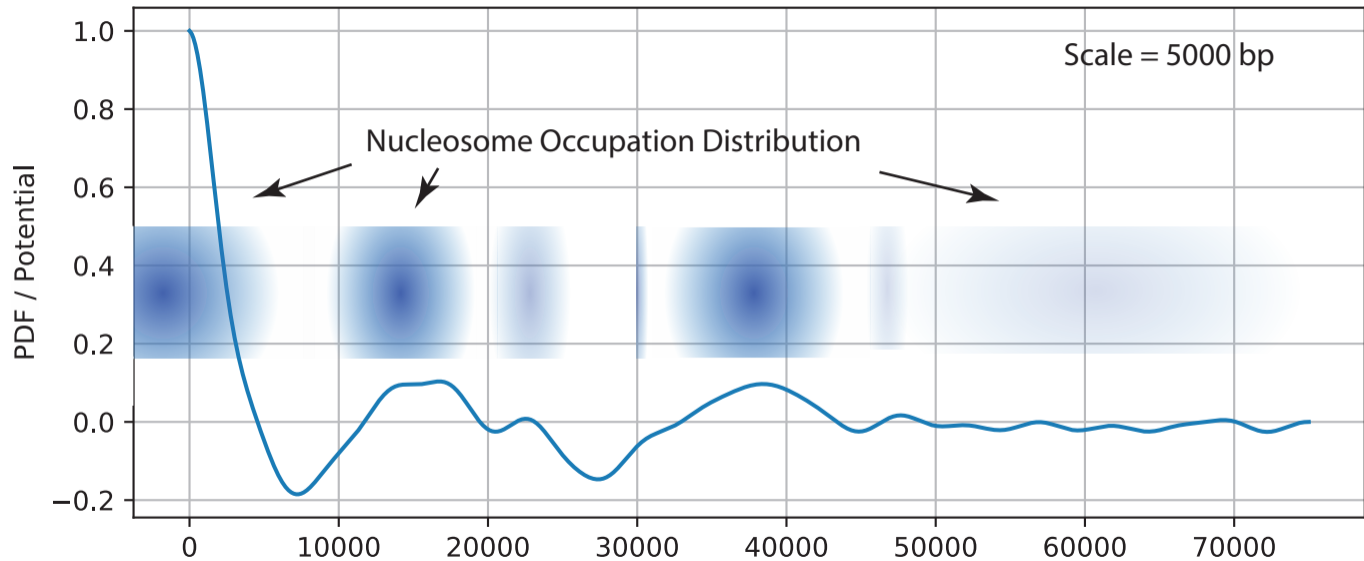

Supplement: Supplementary file 1 [file life-12-00541-s001.zip › life-1592845-supplementary/Heermann-Nuc-Dist.pdf]

Nucleosome Density: chr1

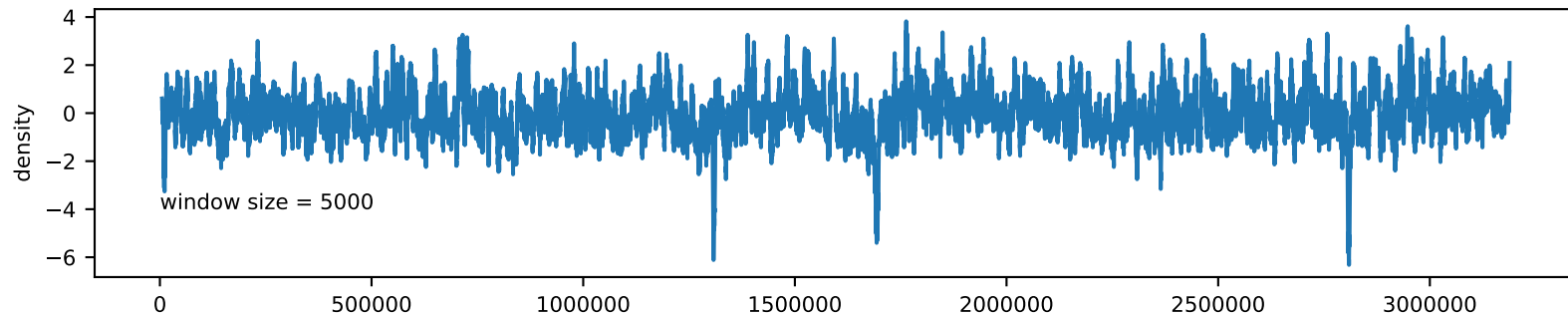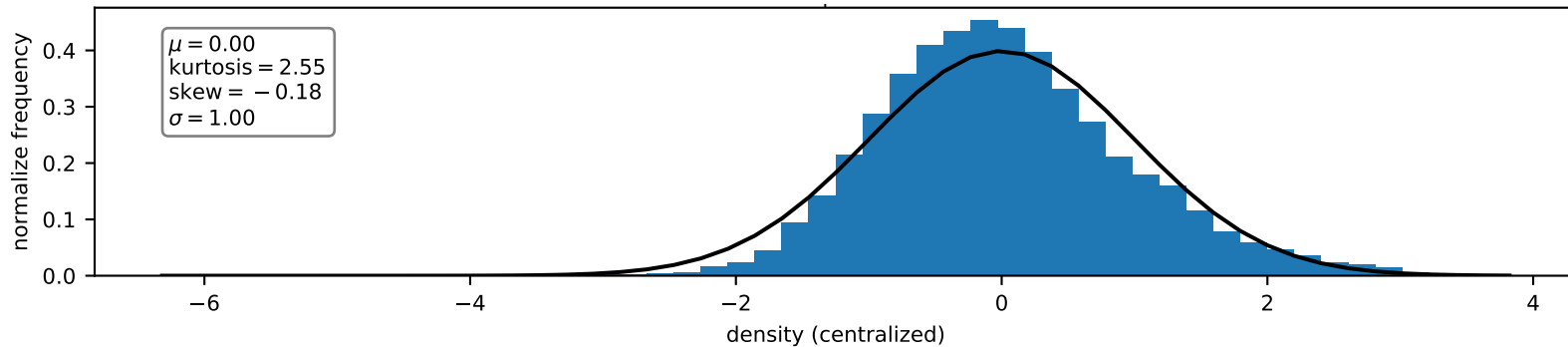

Supplement: Supplementary file 1 [file life-12-00541-s001.zip › life-1592845-supplementary/Heermann-nucleosome-density-chr1.pdf]

Nucleosome Density: chr2

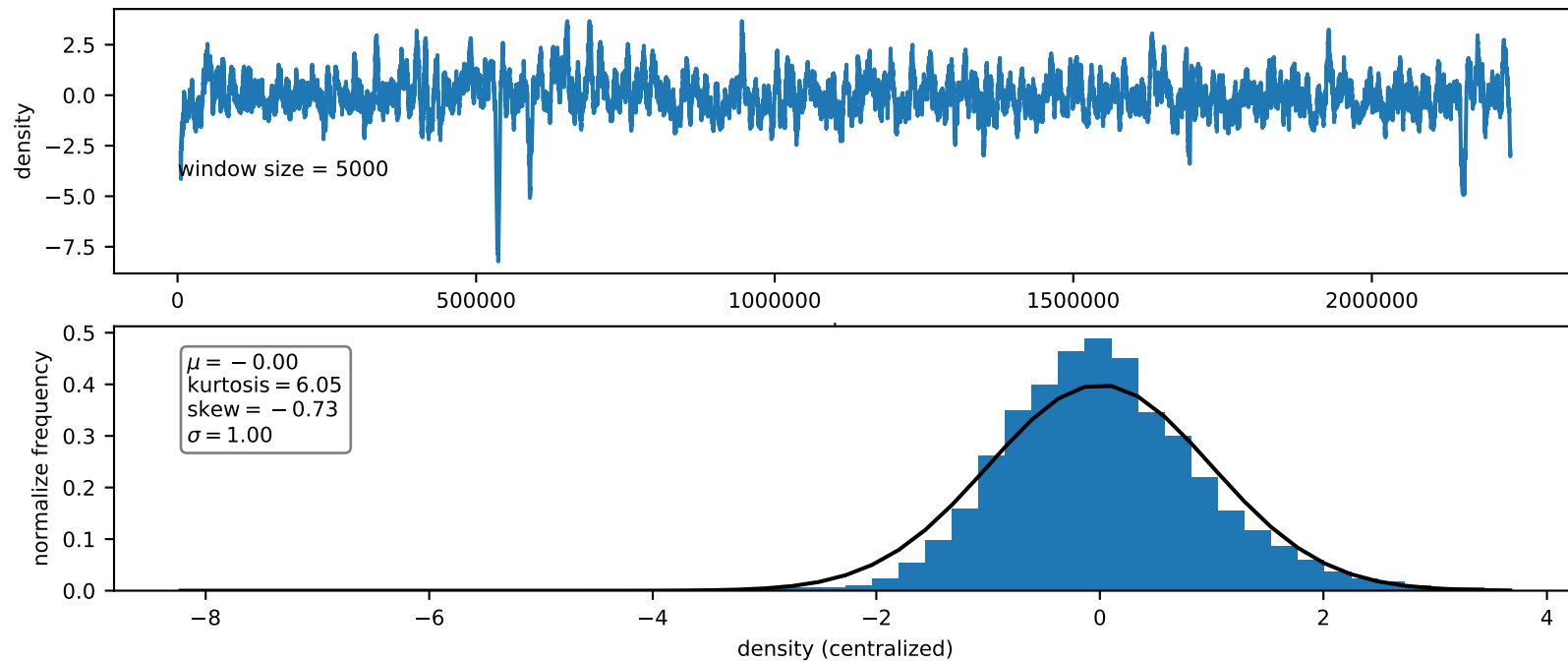

Supplement: Supplementary file 1 [file life-12-00541-s001.zip › life-1592845-supplementary/Heermann-nucleosome-density-chr2.pdf]

Nucleosome Density: chr3

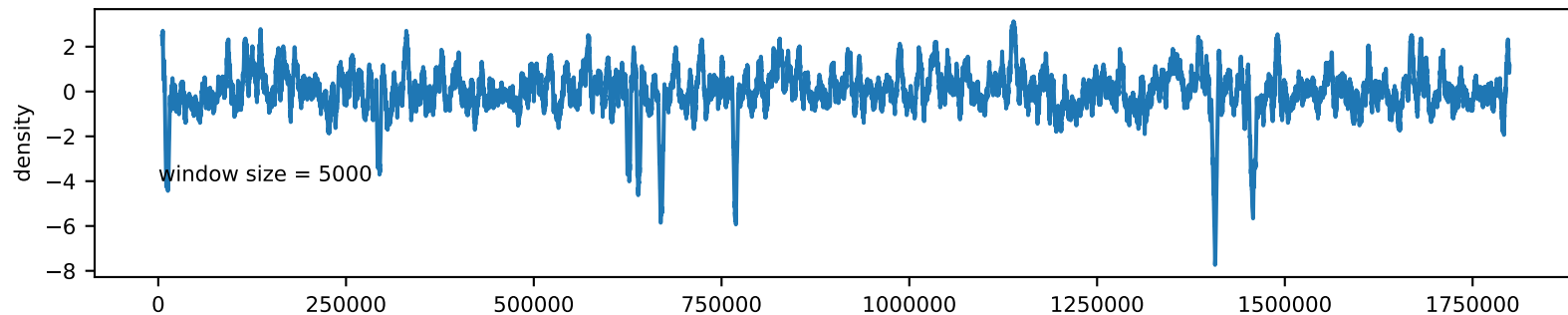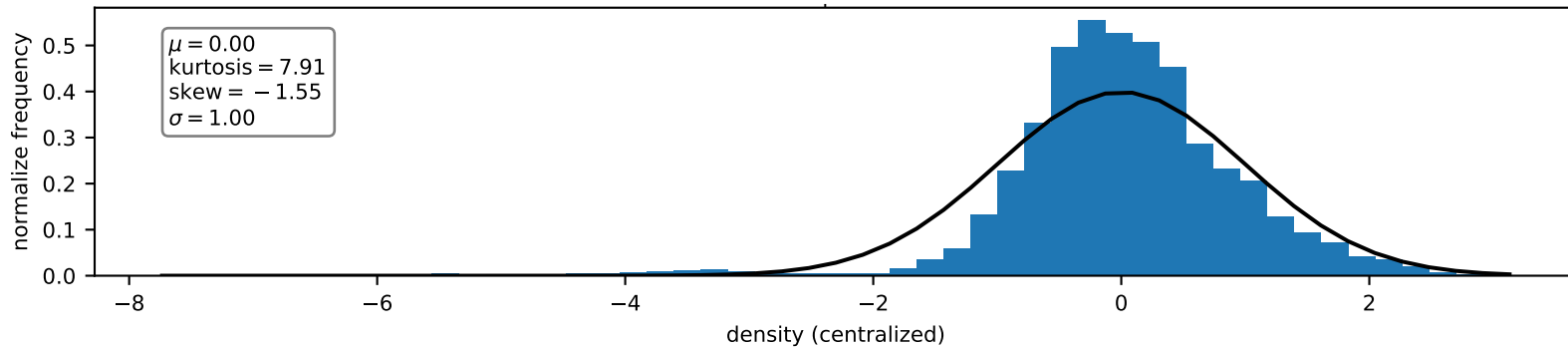

Supplement: Supplementary file 1 [file life-12-00541-s001.zip › life-1592845-supplementary/Heermann-nucleosome-density-chr3.pdf]

Nucleosome Density: chr4

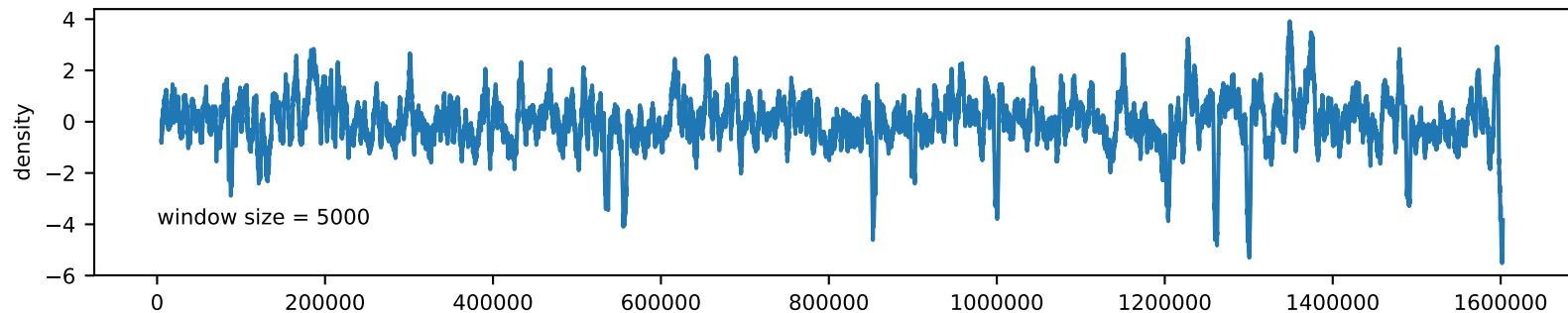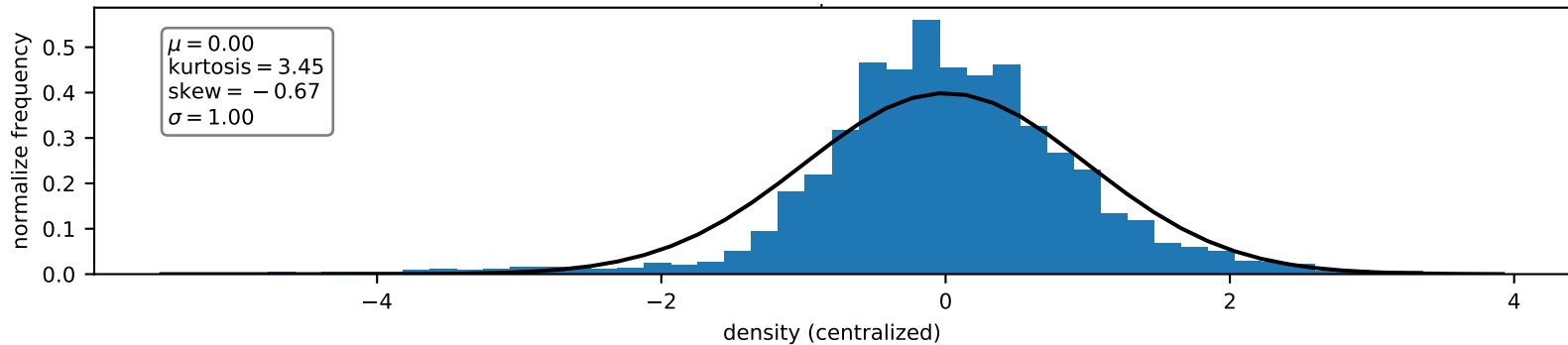

Supplement: Supplementary file 1 [file life-12-00541-s001.zip › life-1592845-supplementary/Heermann-nucleosome-density-chr4.pdf]

Nucleosome Density: chr5

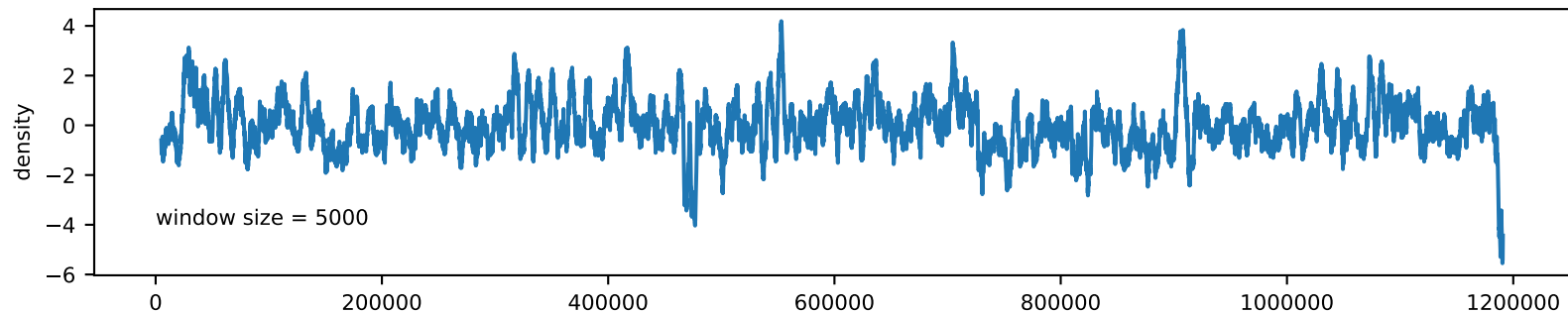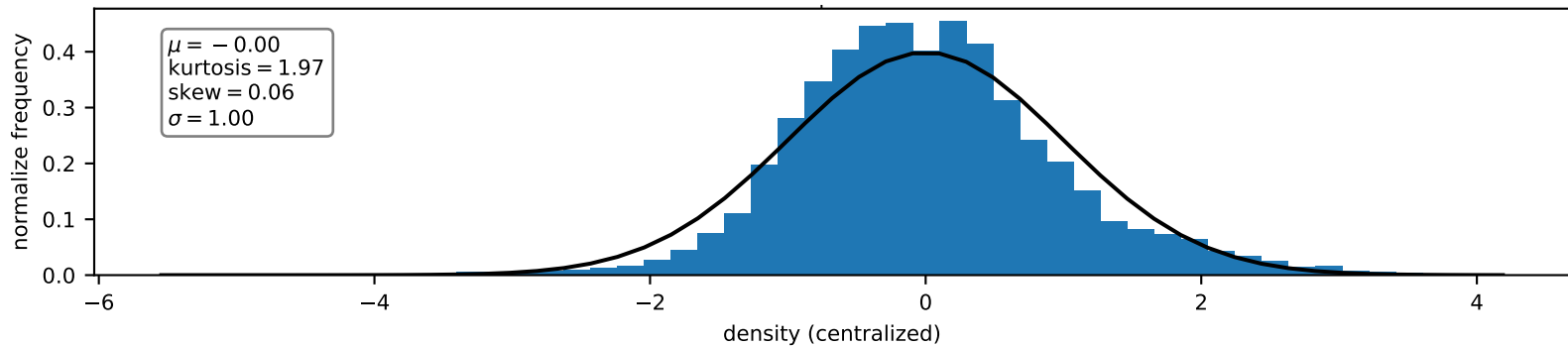

Supplement: Supplementary file 1 [file life-12-00541-s001.zip › life-1592845-supplementary/Heermann-nucleosome-density-chr5.pdf]

Nucleosome Density: chr6

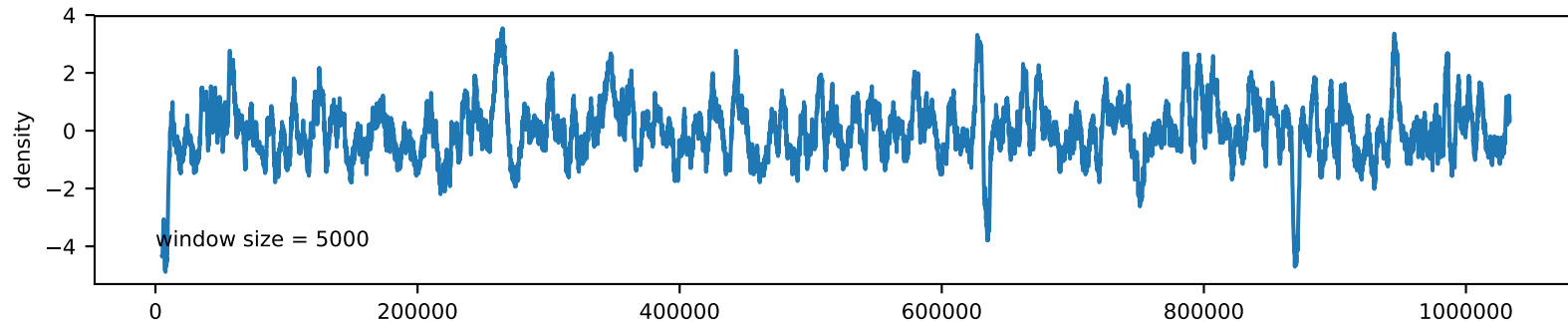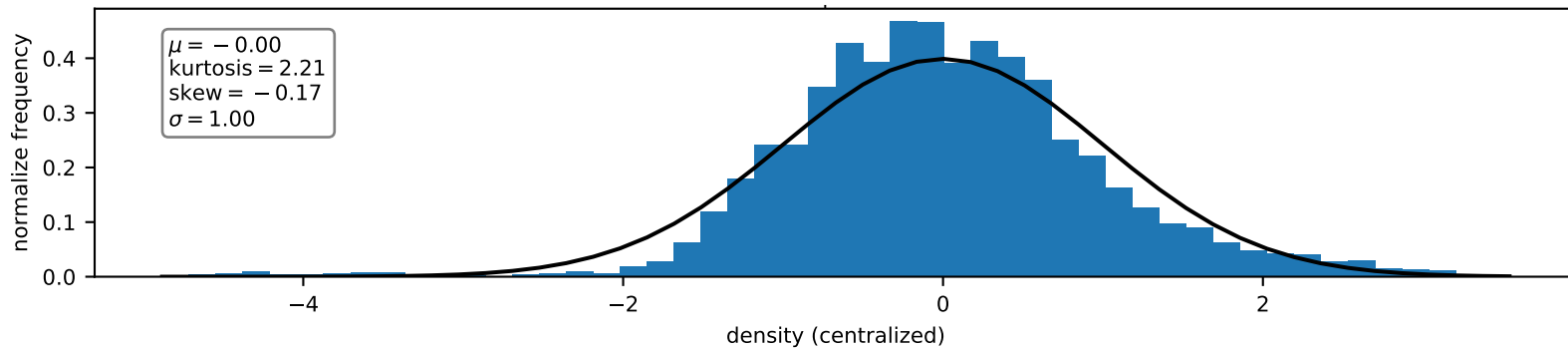

Supplement: Supplementary file 1 [file life-12-00541-s001.zip › life-1592845-supplementary/Heermann-nucleosome-density-chr6.pdf]

Nucleosome Density: chr7

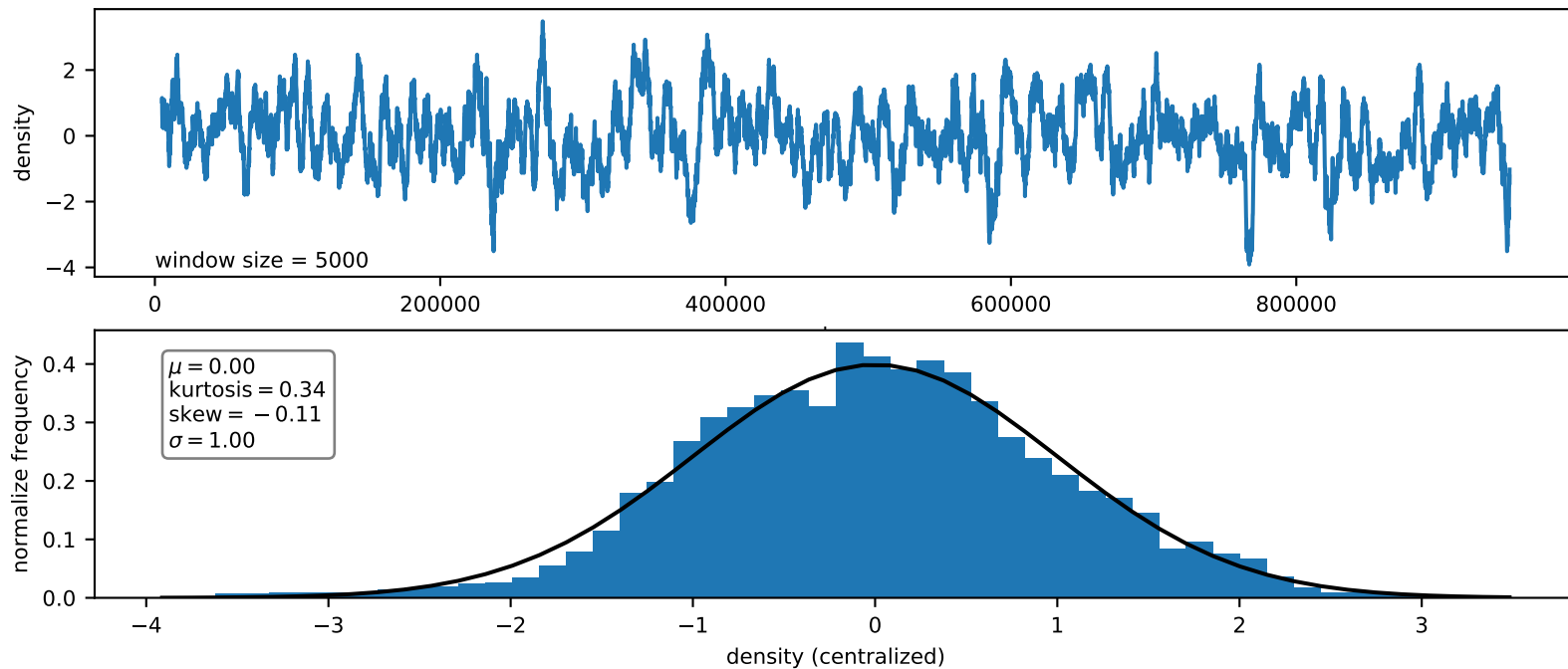

Supplement: Supplementary file 1 [file life-12-00541-s001.zip › life-1592845-supplementary/Heermann-nucleosome-density-chr7.pdf]

Nucleosome Density: chrR

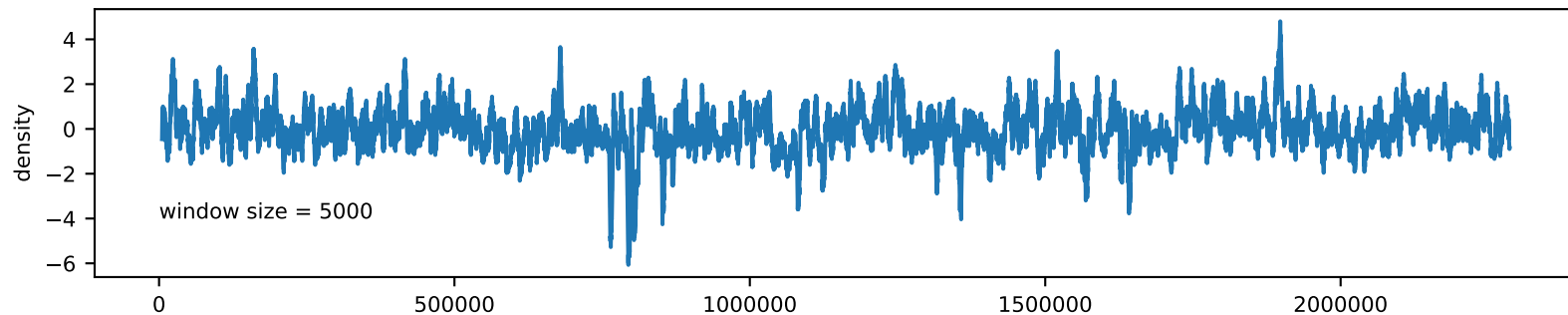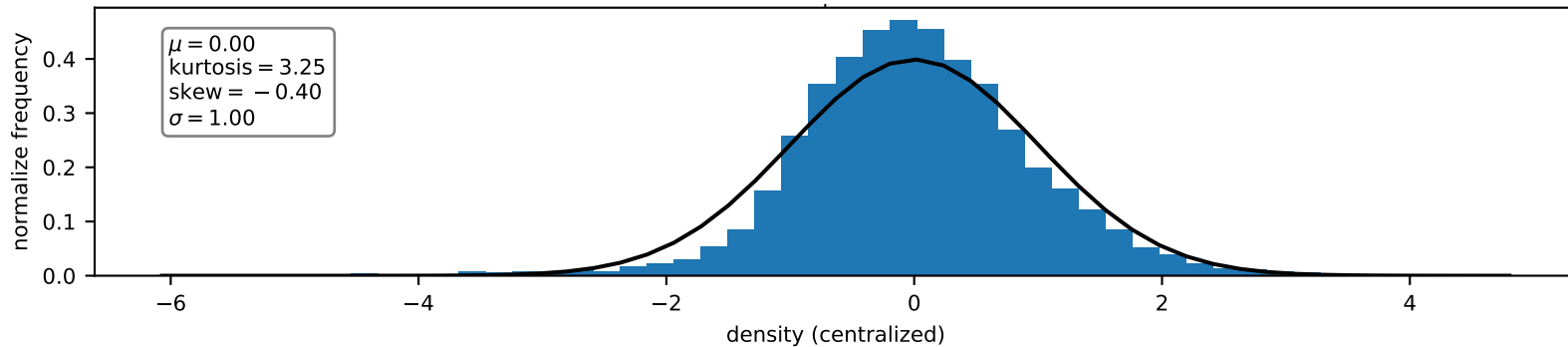

Supplement: Supplementary file 1 [file life-12-00541-s001.zip › life-1592845-supplementary/Heermann-nucleosome-density-chrR.pdf]

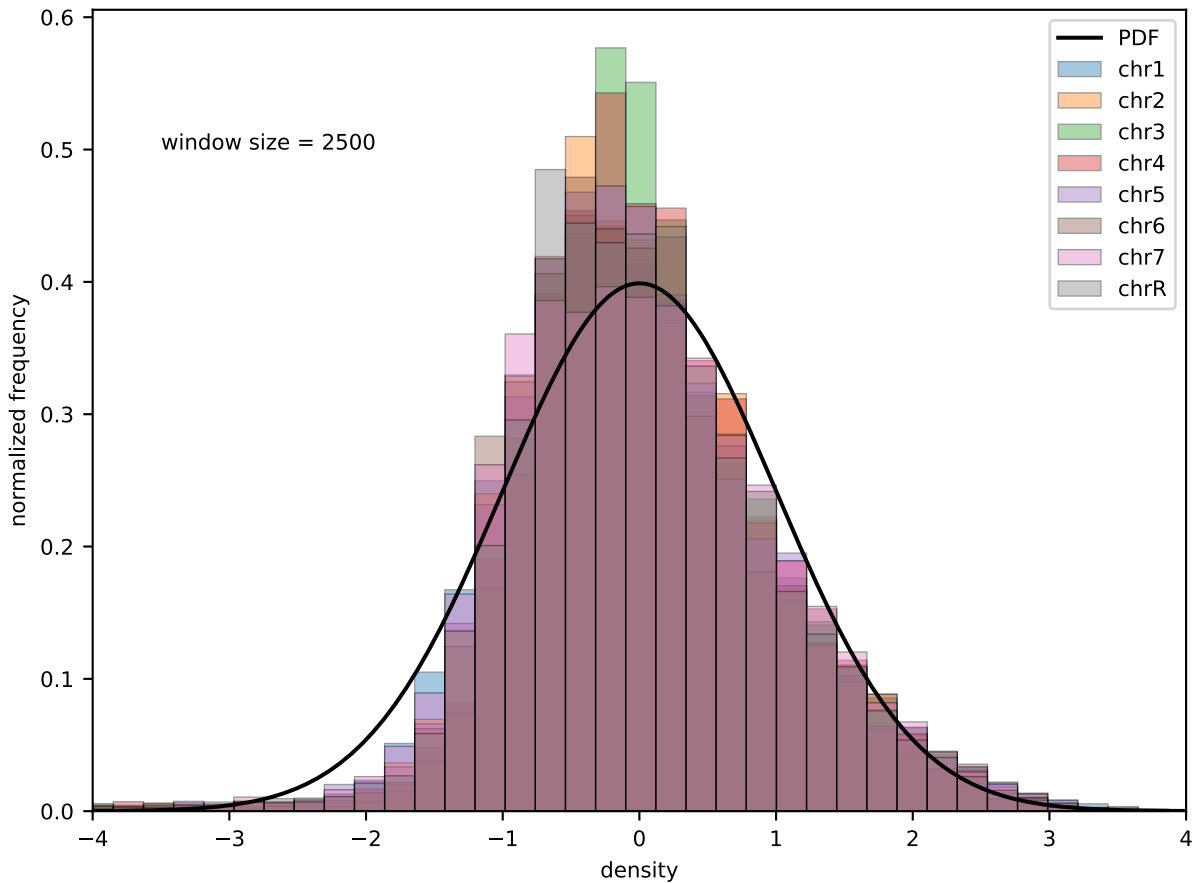

Supplement: Supplementary file 1 [file life-12-00541-s001.zip › life-1592845-supplementary/Heermann-nucleosome-density-scaling-2500.pdf]

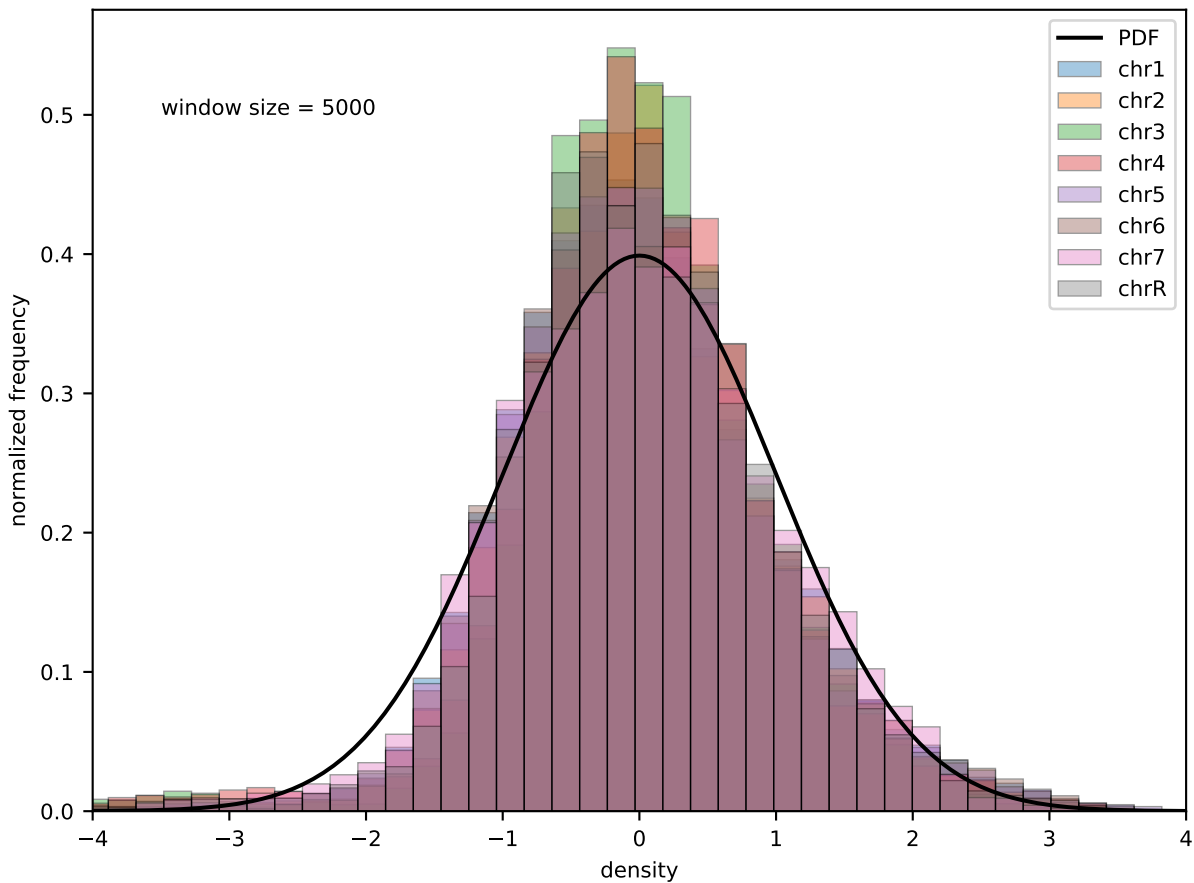

Supplement: Supplementary file 1 [file life-12-00541-s001.zip › life-1592845-supplementary/Heermann-nucleosome-density-scaling-5000.pdf]

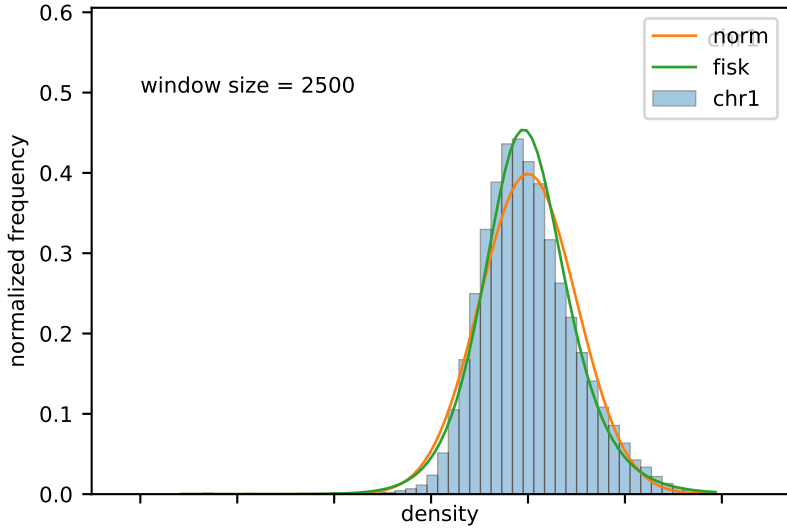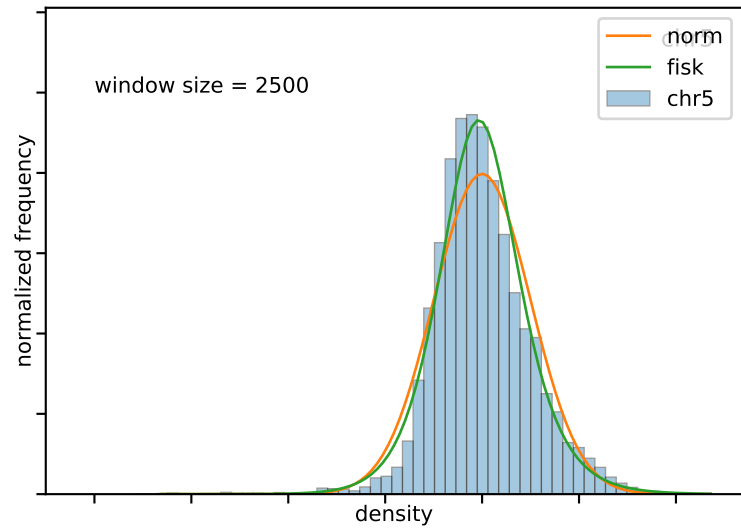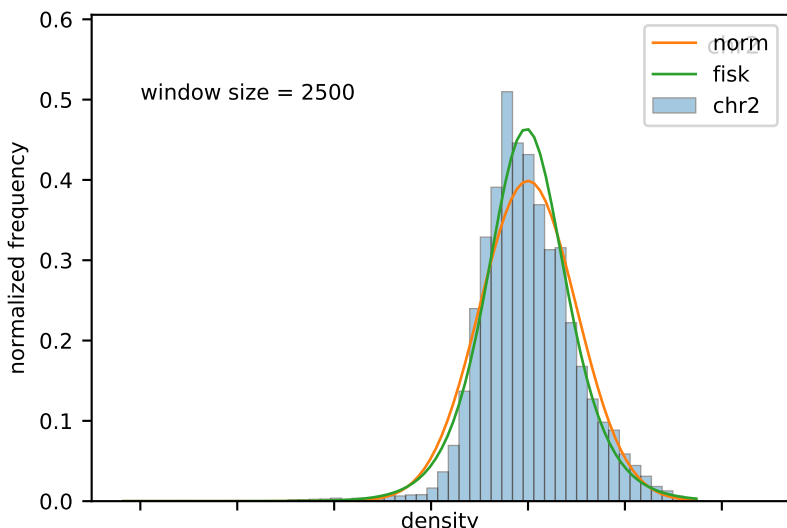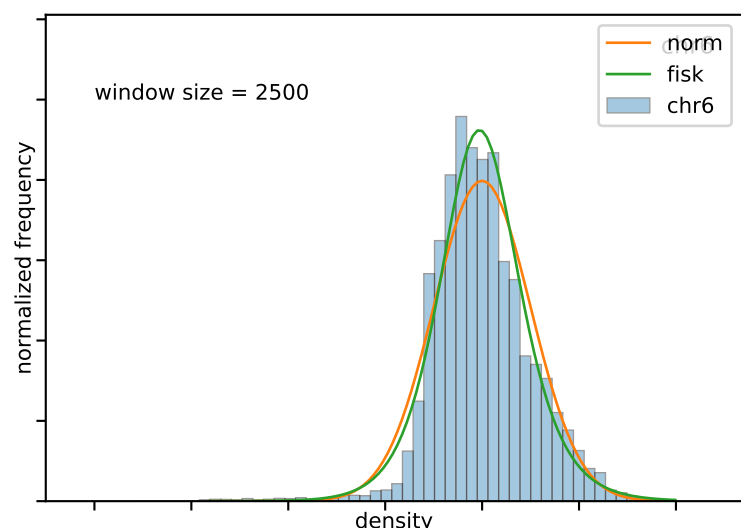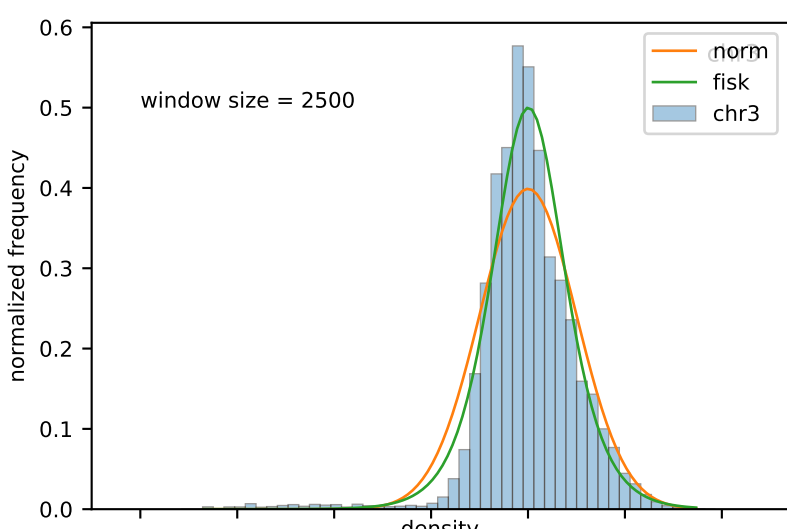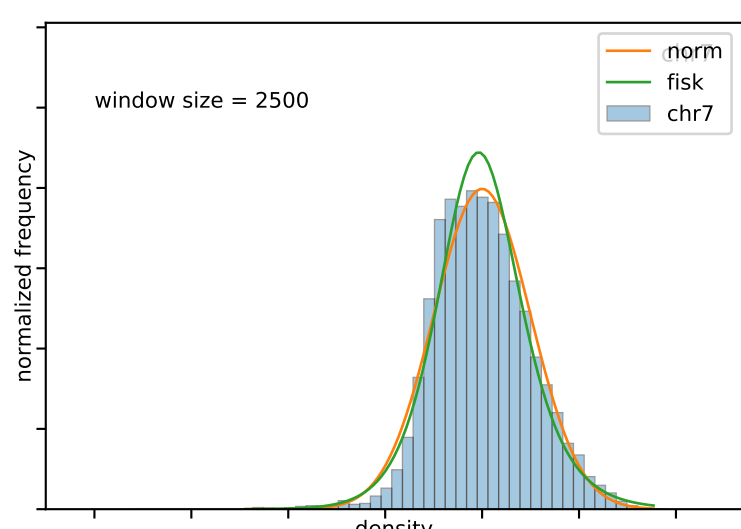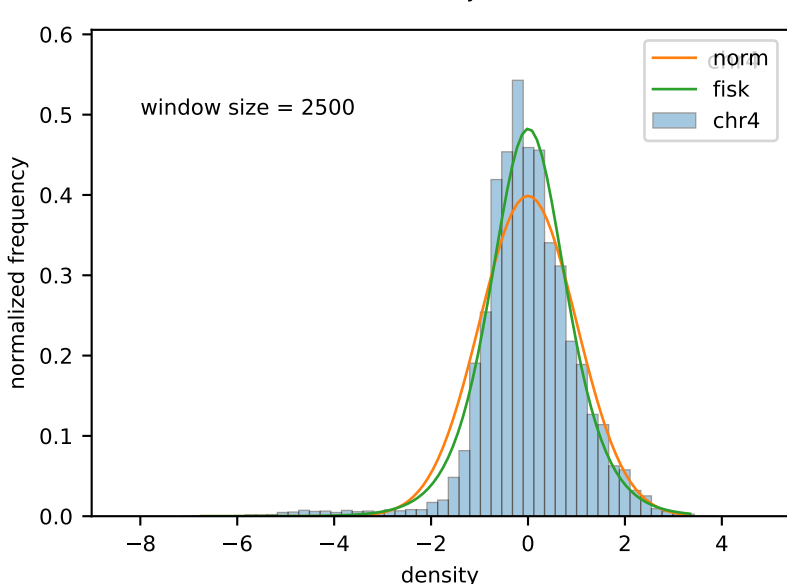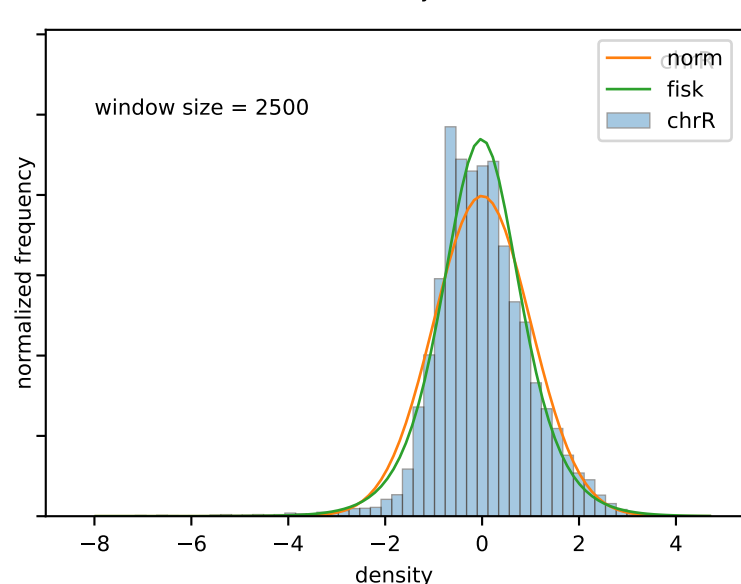

Supplement: Supplementary file 1 [file life-12-00541-s001.zip › life-1592845-supplementary/Heermann-nucleosome-density-scaling-indi-2500.pdf]

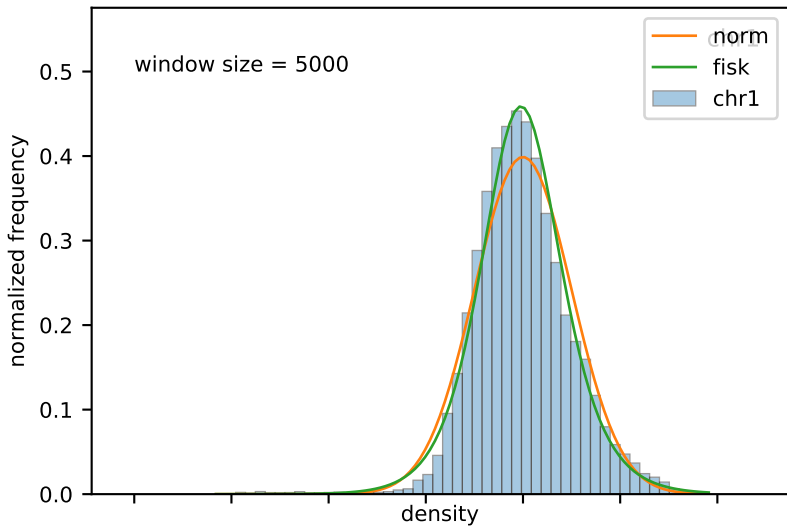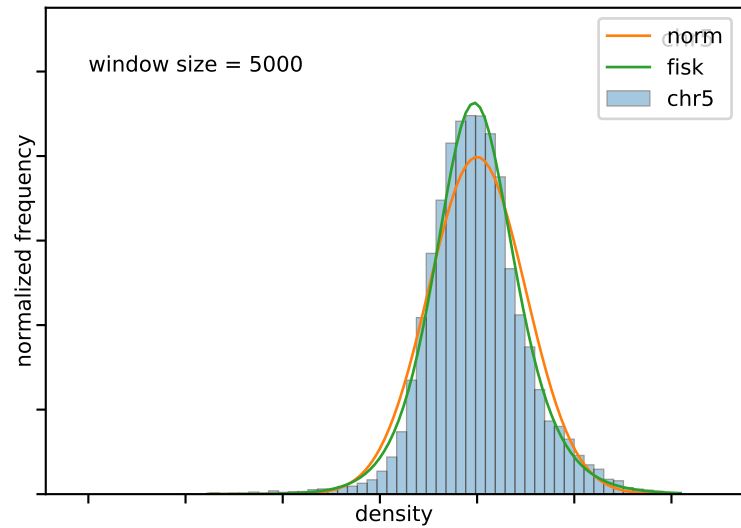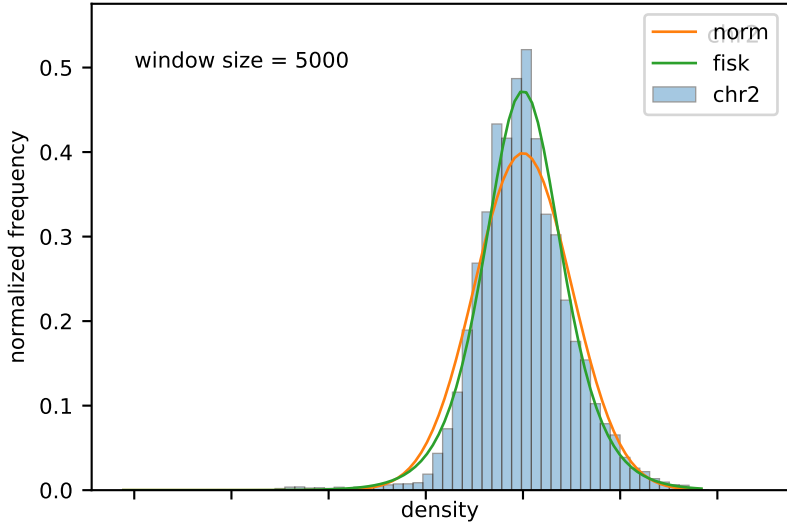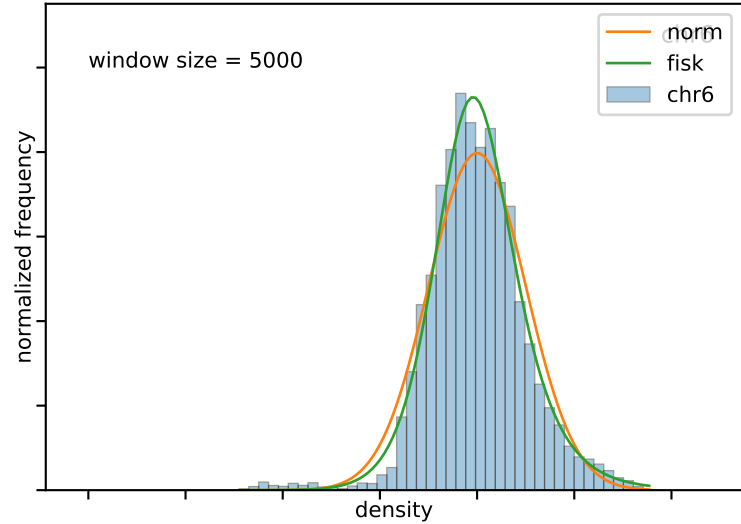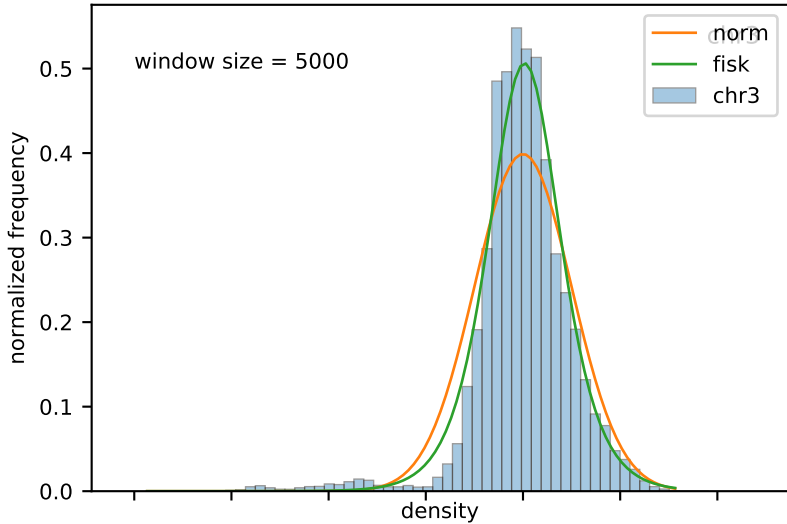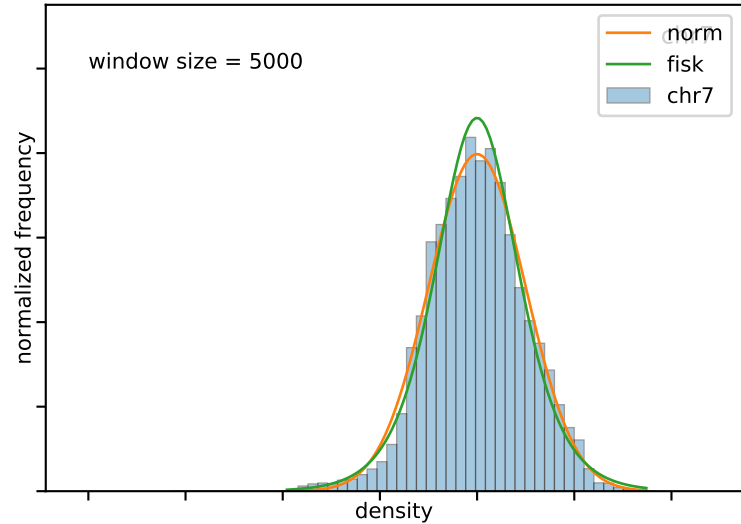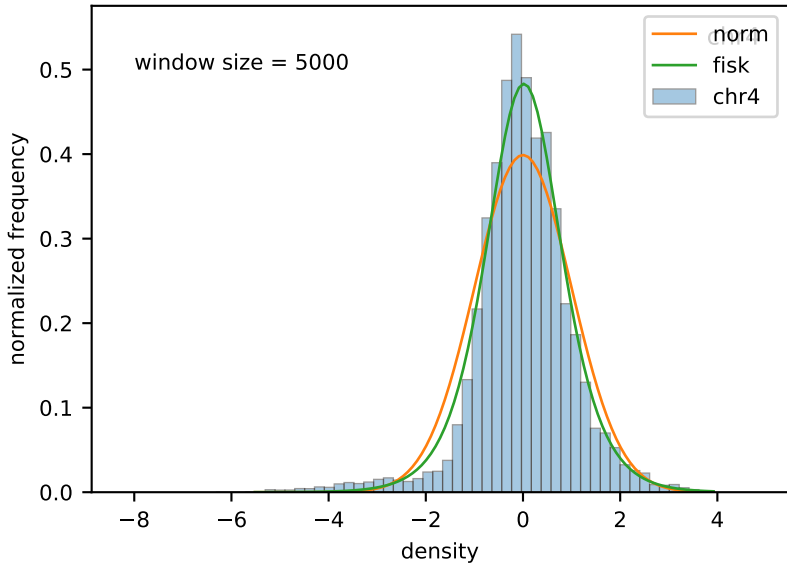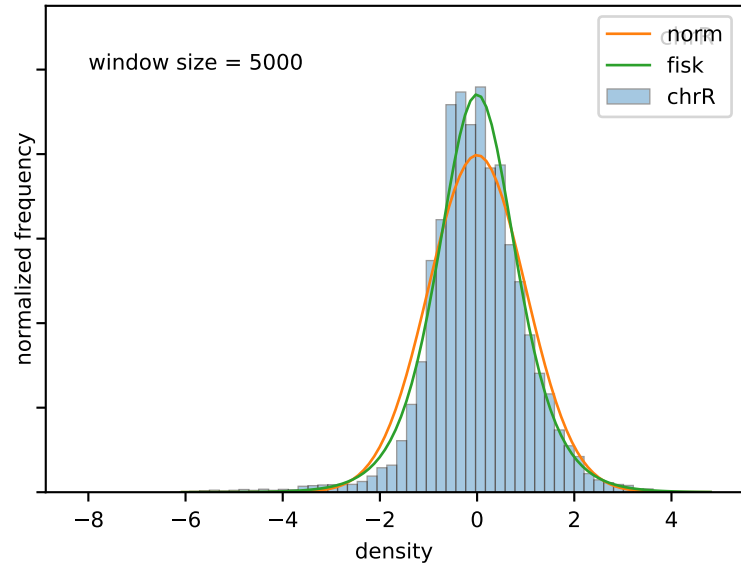

Supplement: Supplementary file 1 [file life-12-00541-s001.zip › life-1592845-supplementary/Heermann-nucleosome-density-scaling-indi-5000.pdf]

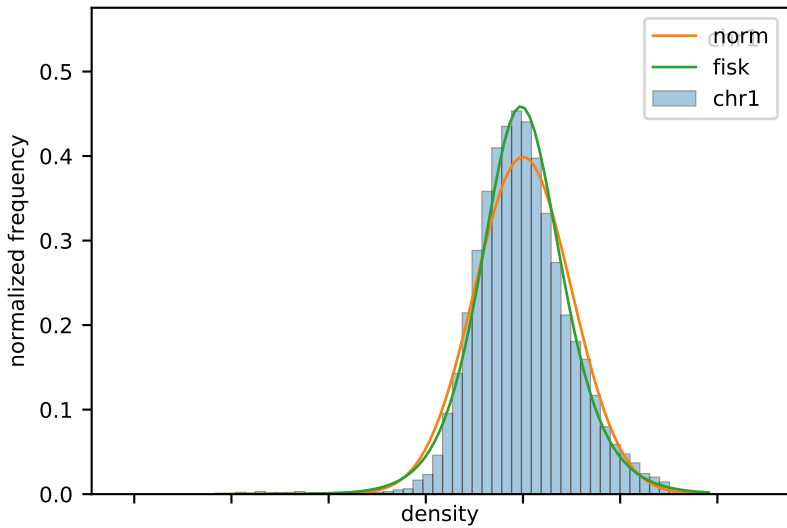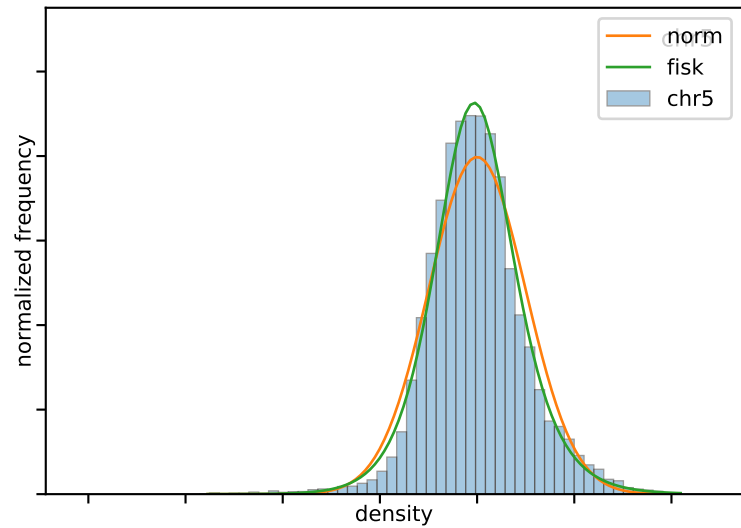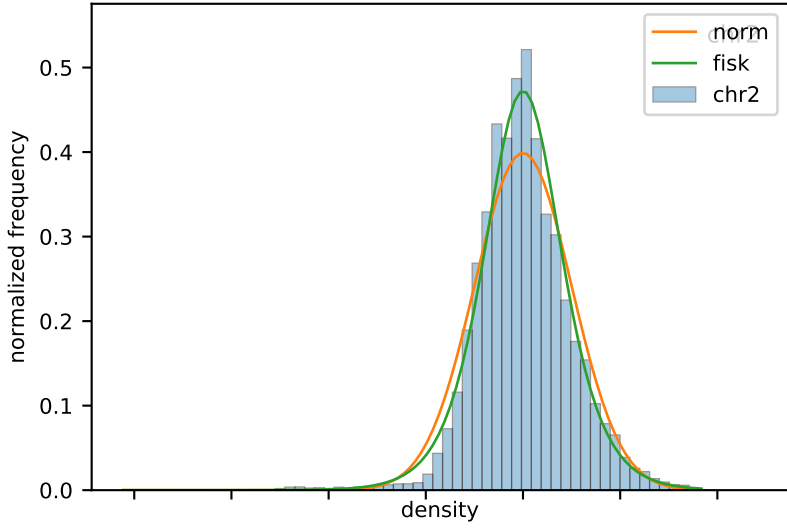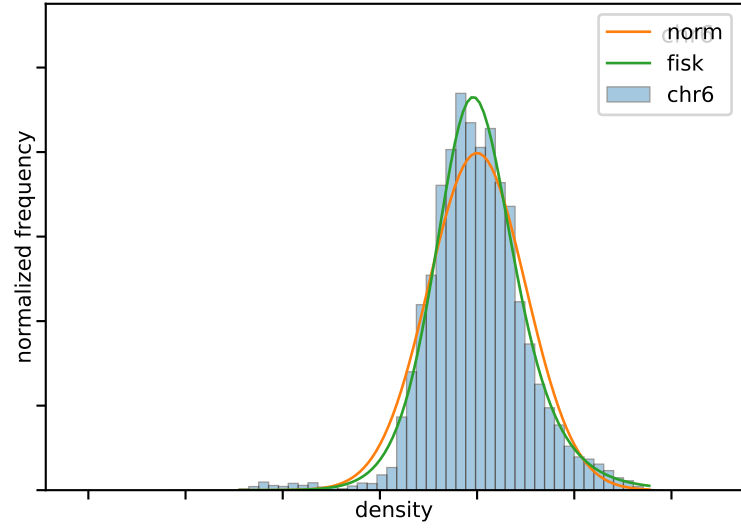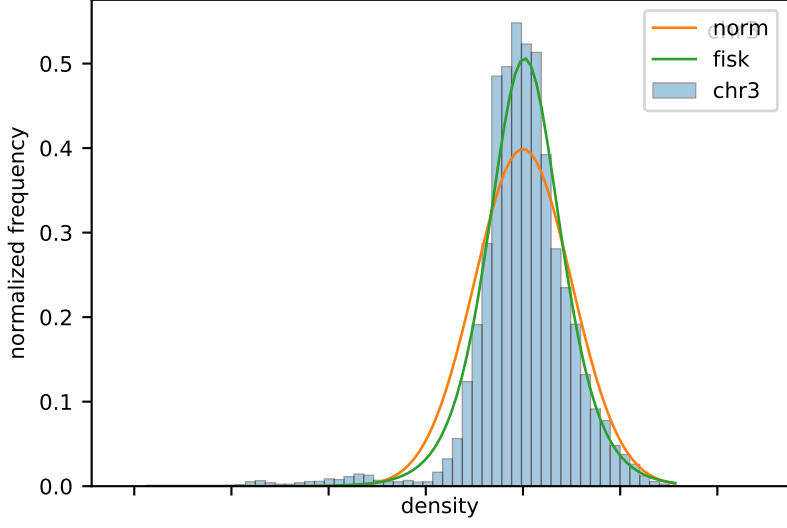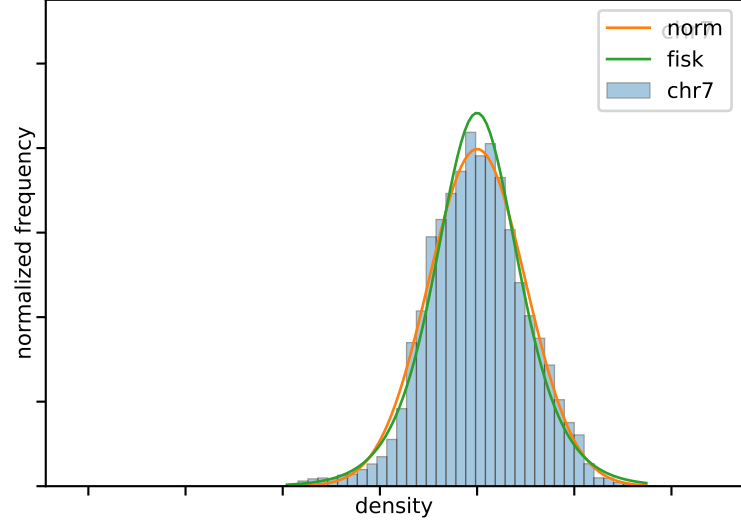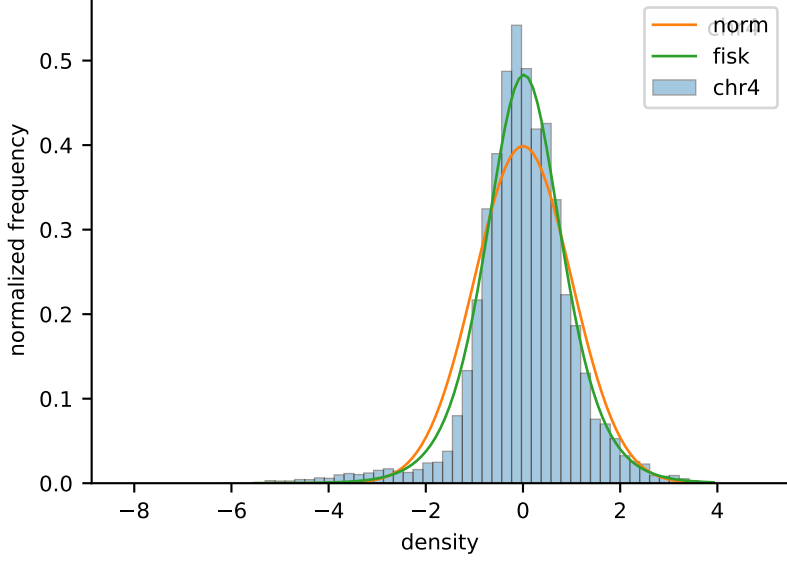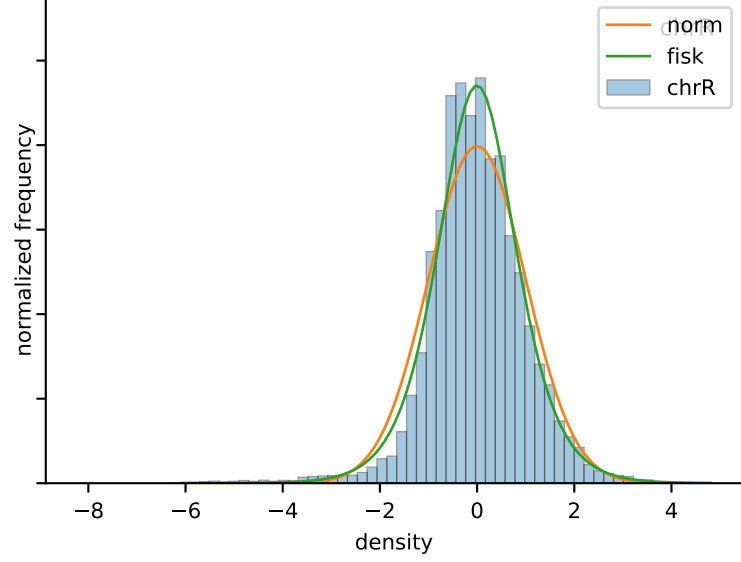

Supplement: Supplementary file 1 [file life-12-00541-s001.zip › life-1592845-supplementary/Heermann-nucleosome-density-scaling-indi.pdf]

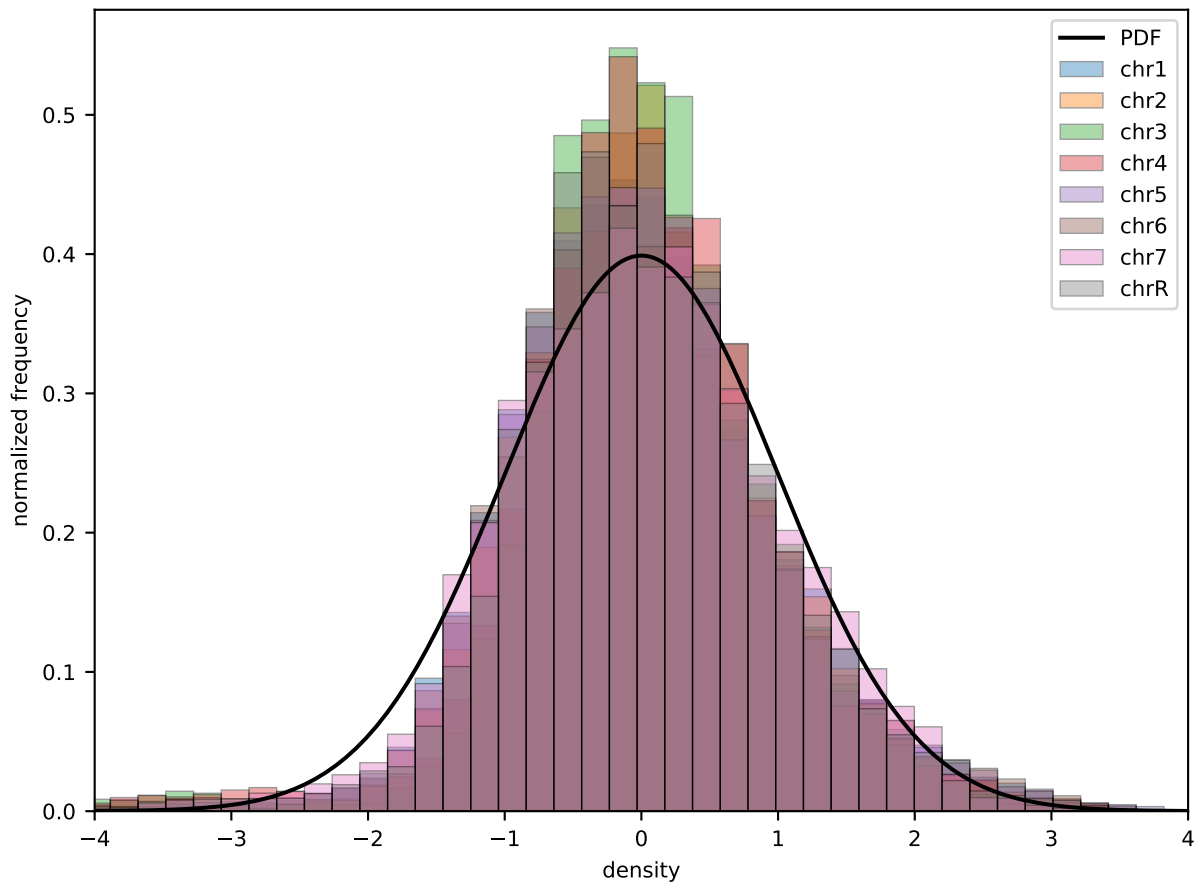

Supplement: Supplementary file 1 [file life-12-00541-s001.zip › life-1592845-supplementary/Heermann-nucleosome-density-scaling.pdf]

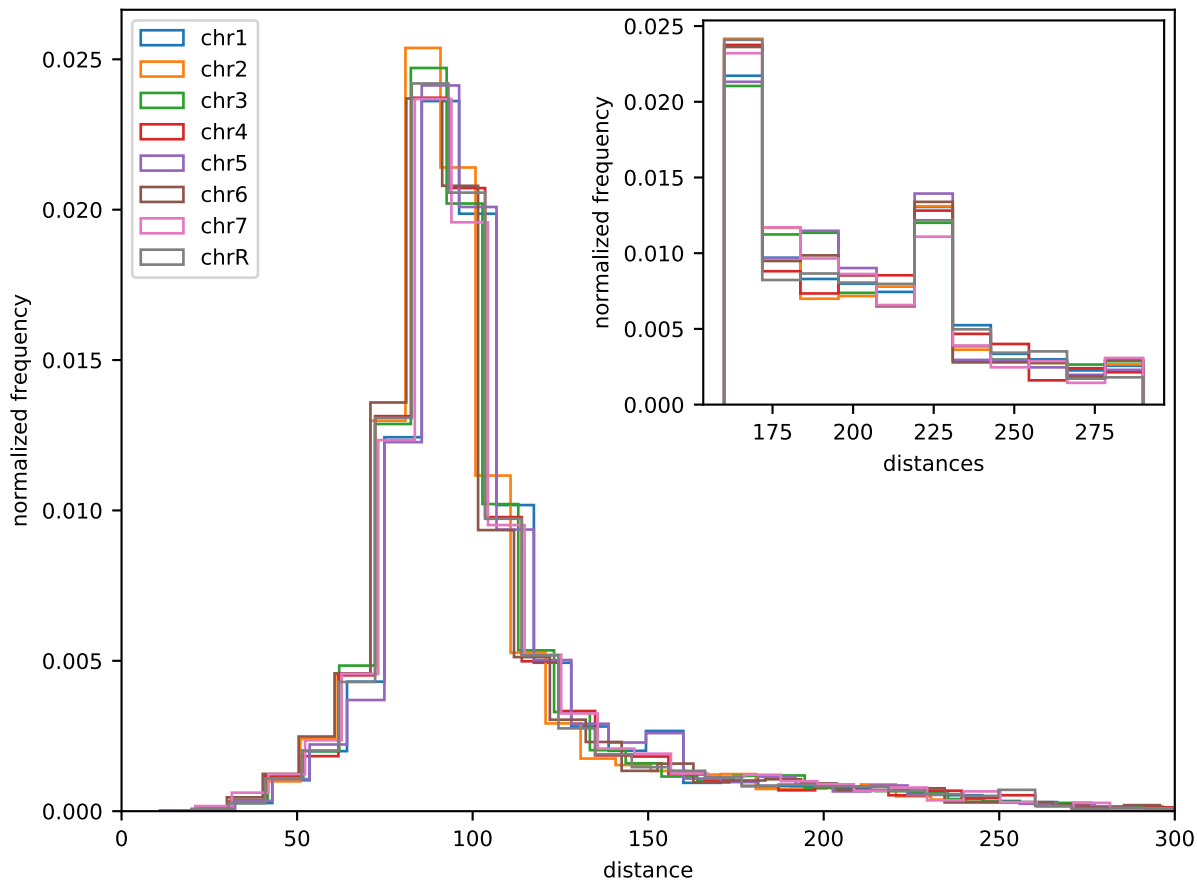

Supplement: Supplementary file 1 [file life-12-00541-s001.zip › life-1592845-supplementary/Heermann-nucleosome-distance-1.pdf]

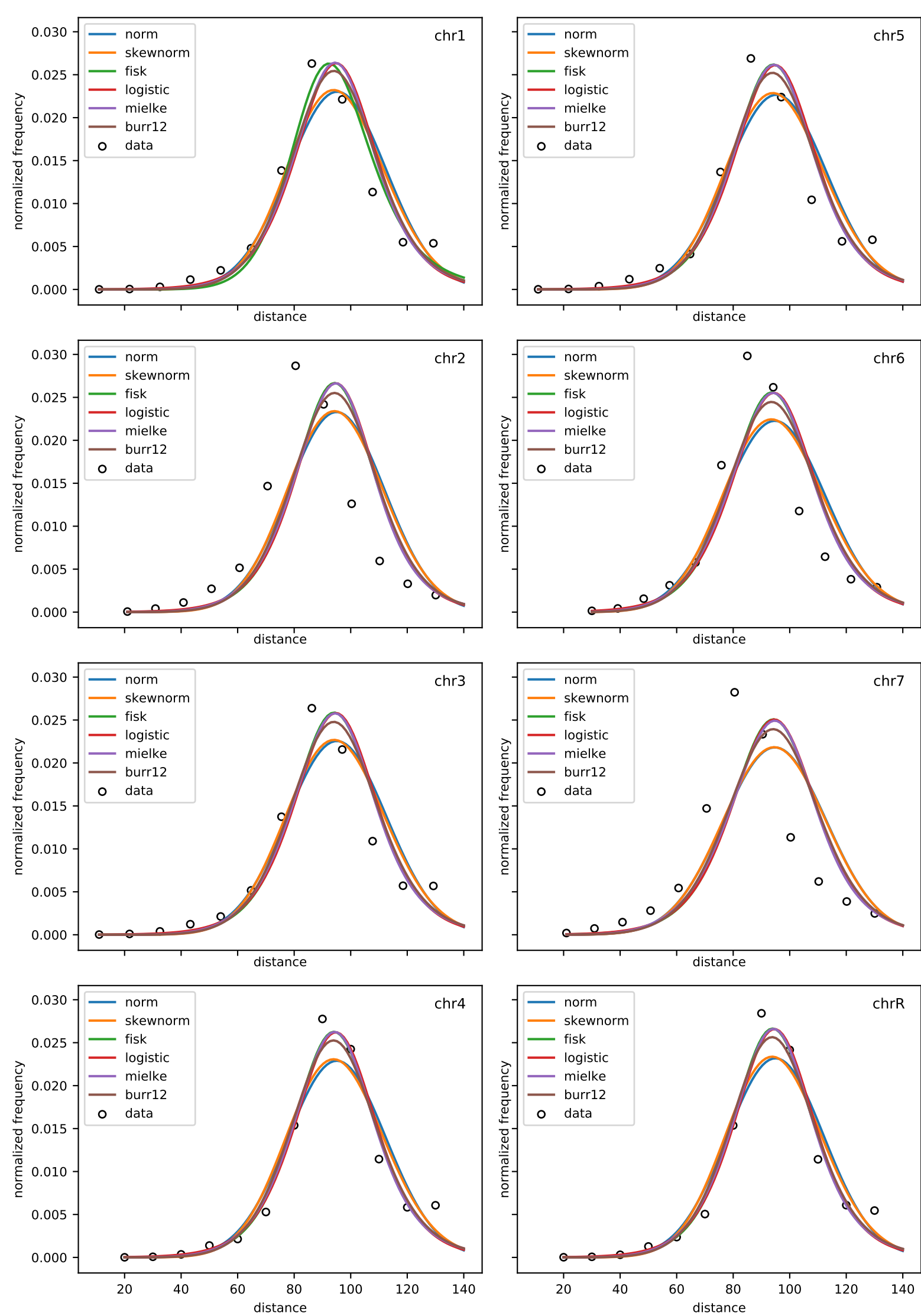

Supplement: Supplementary file 1 [file life-12-00541-s001.zip › life-1592845-supplementary/Heermann-nucleosome-distance-2.pdf]

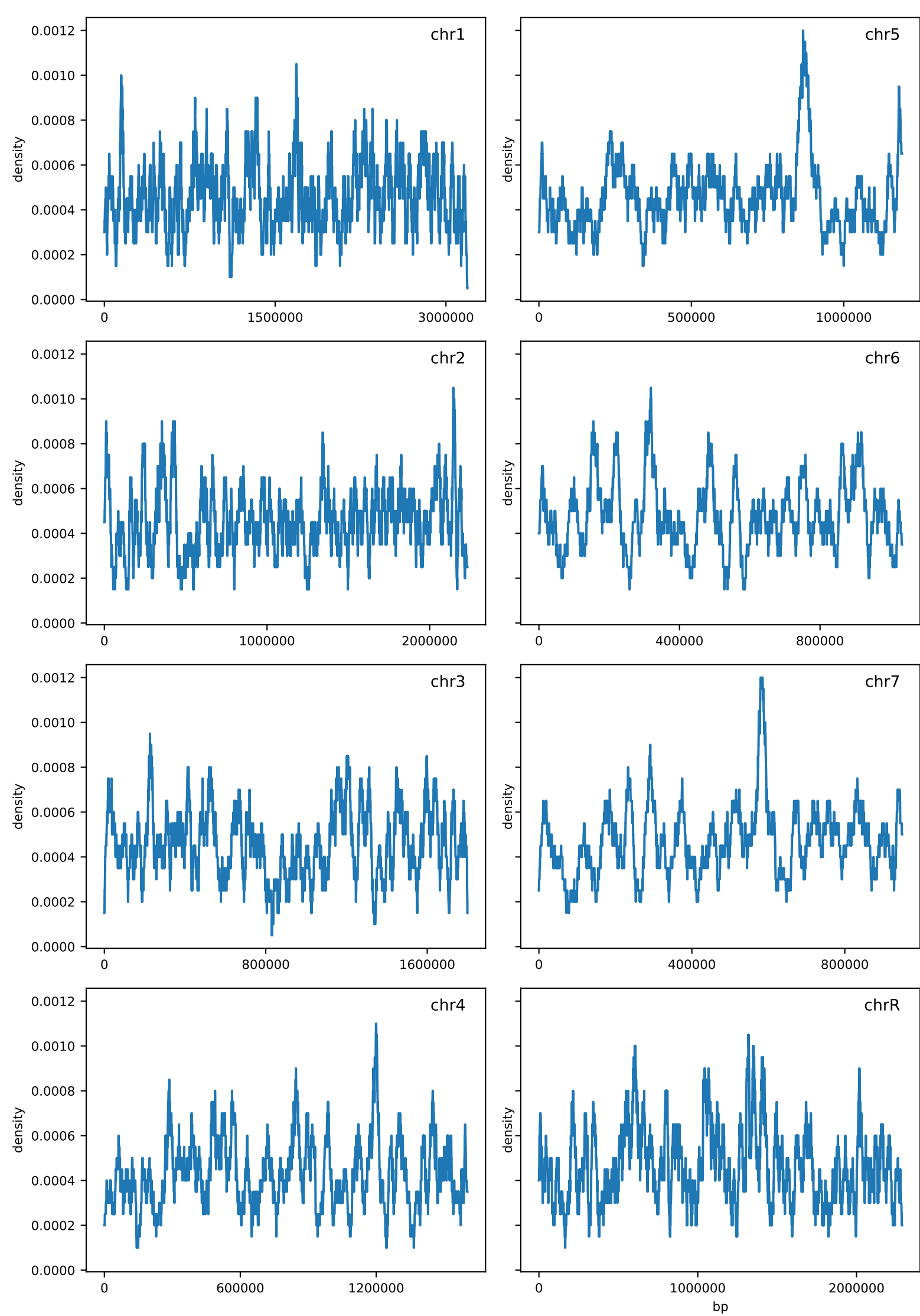

Supplement: Supplementary file 1 [file life-12-00541-s001.zip › life-1592845-supplementary/Heermann-nucleosome-distance-large.pdf]

**A**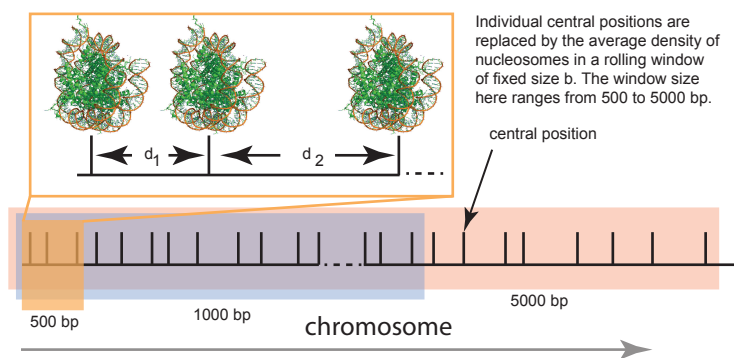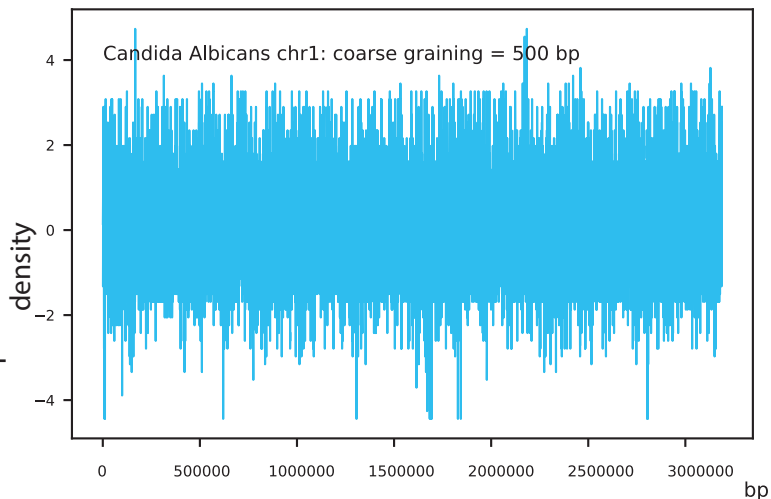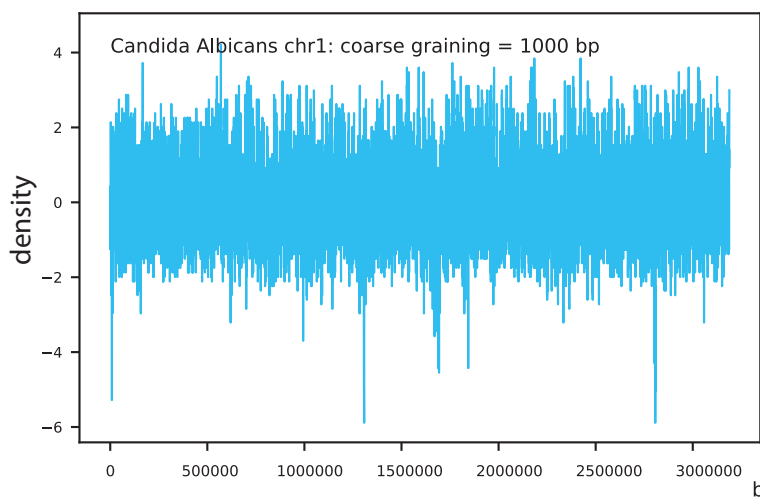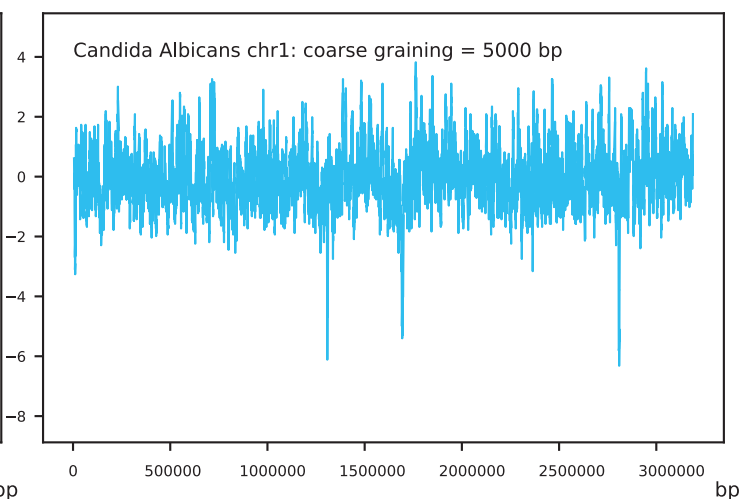**B**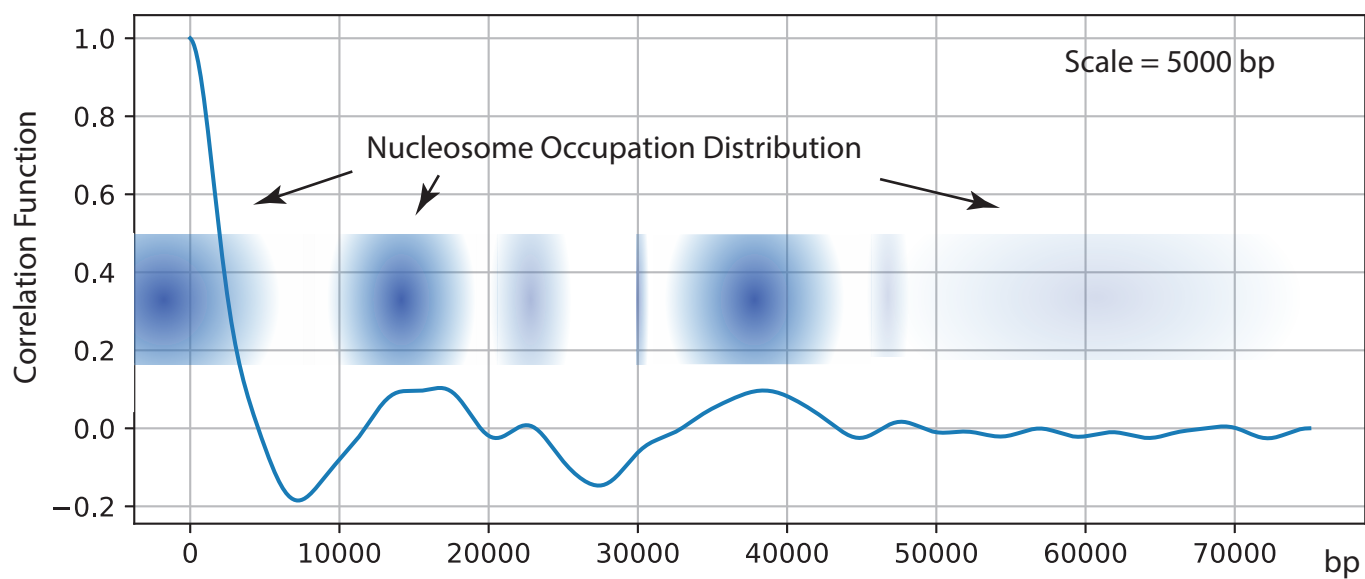**C**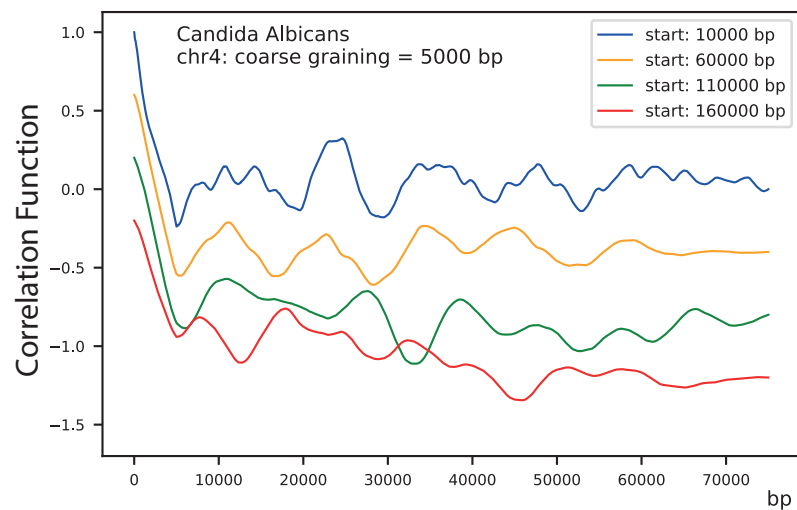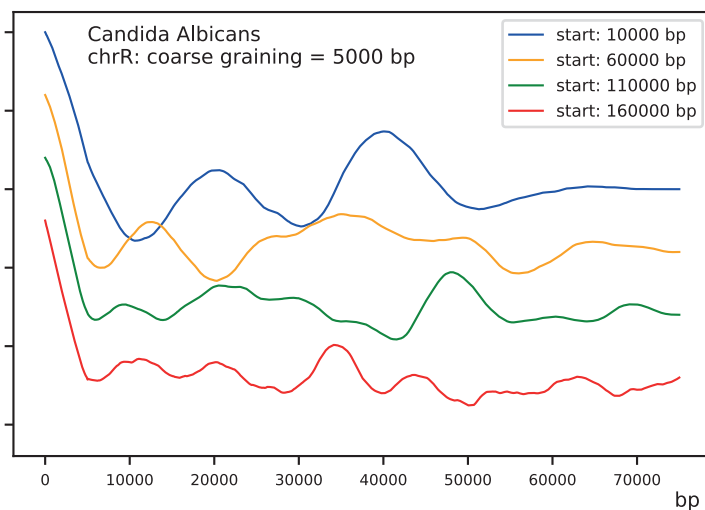

Supplement: Supplementary file 1 [file life-12-00541-s001.zip › life-1592845-supplementary/Heermann-overview.pdf]

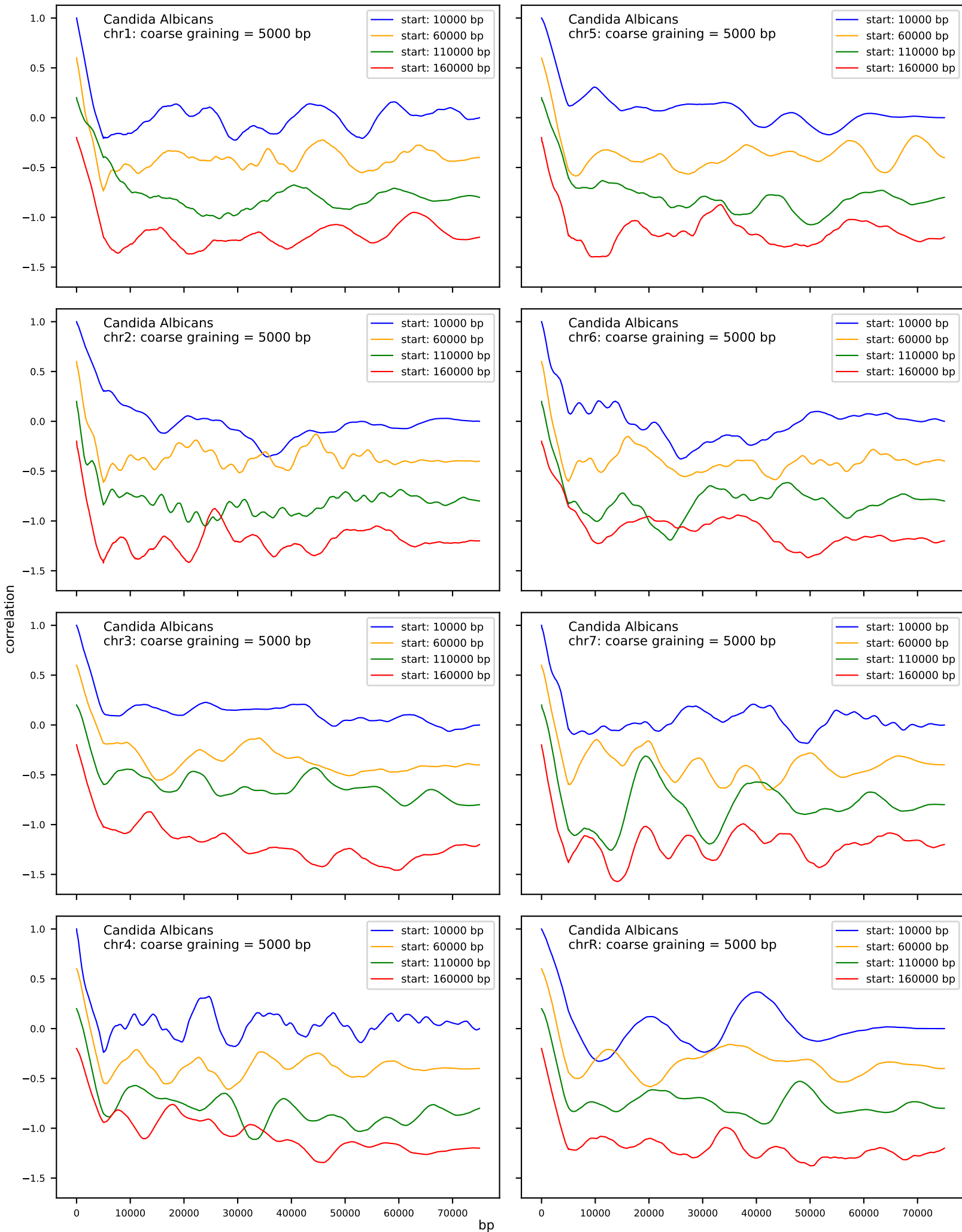

Supplement: Supplementary file 1 [file life-12-00541-s001.zip › life-1592845-supplementary/Heermann-pdf-5000-all.pdf]

# First Peak Analysis of the Pair Correlation Function: CHR1

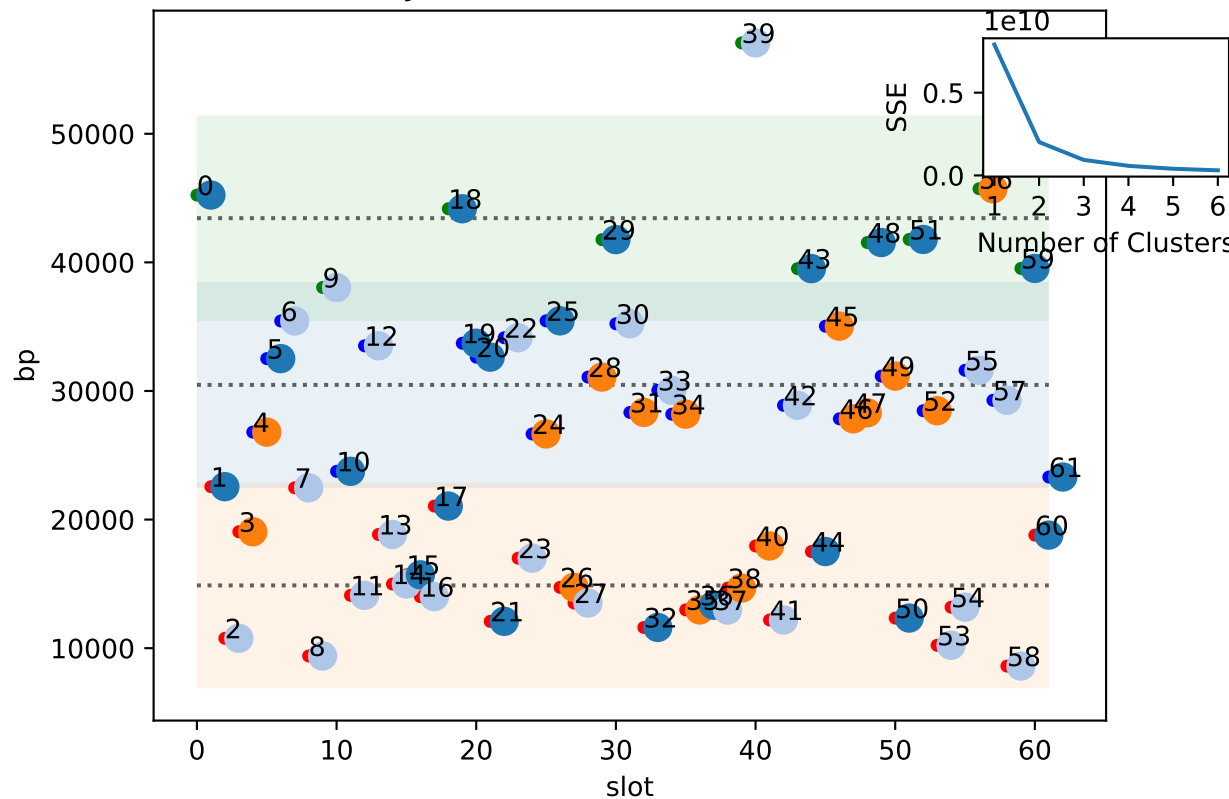

Supplement: Supplementary file 1 [file life-12-00541-s001.zip › life-1592845-supplementary/Heermann-peaks-pdf-chr1.pdf]

**A**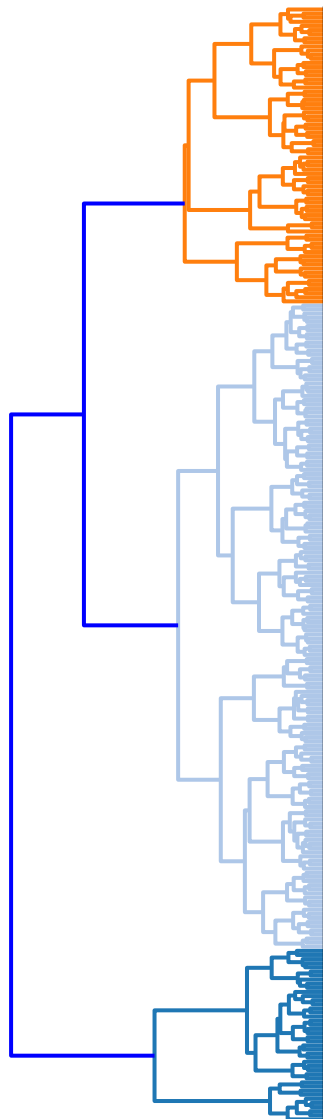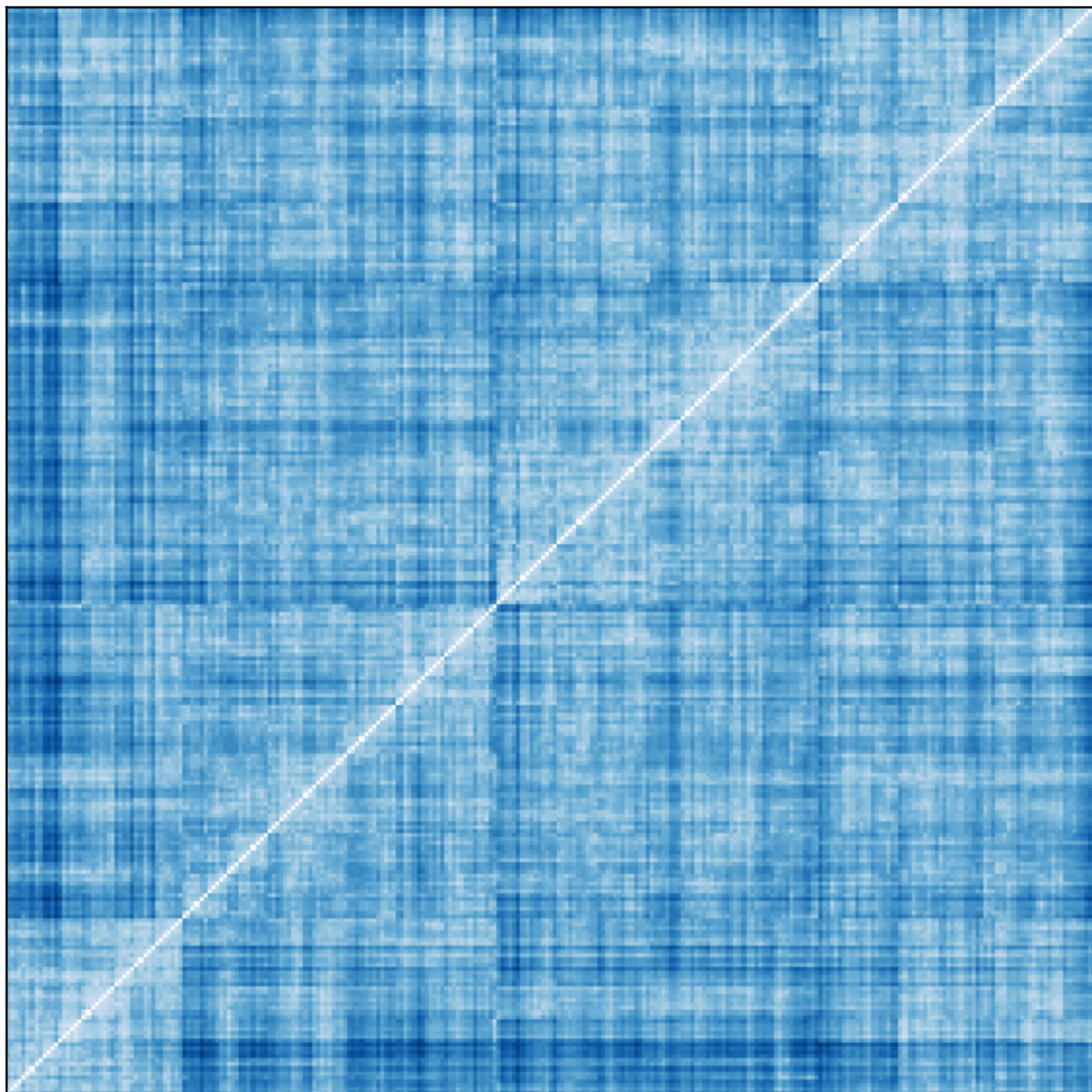**B**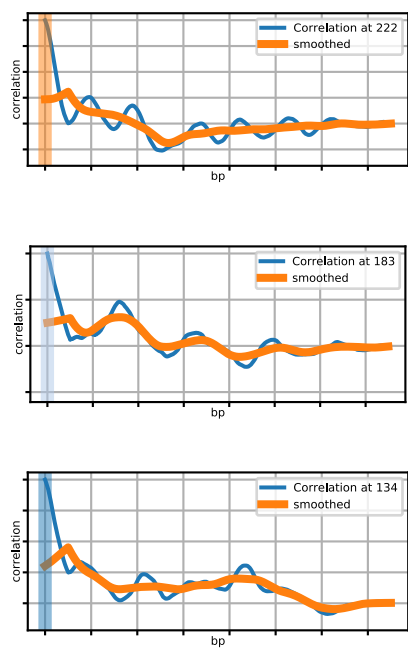**C**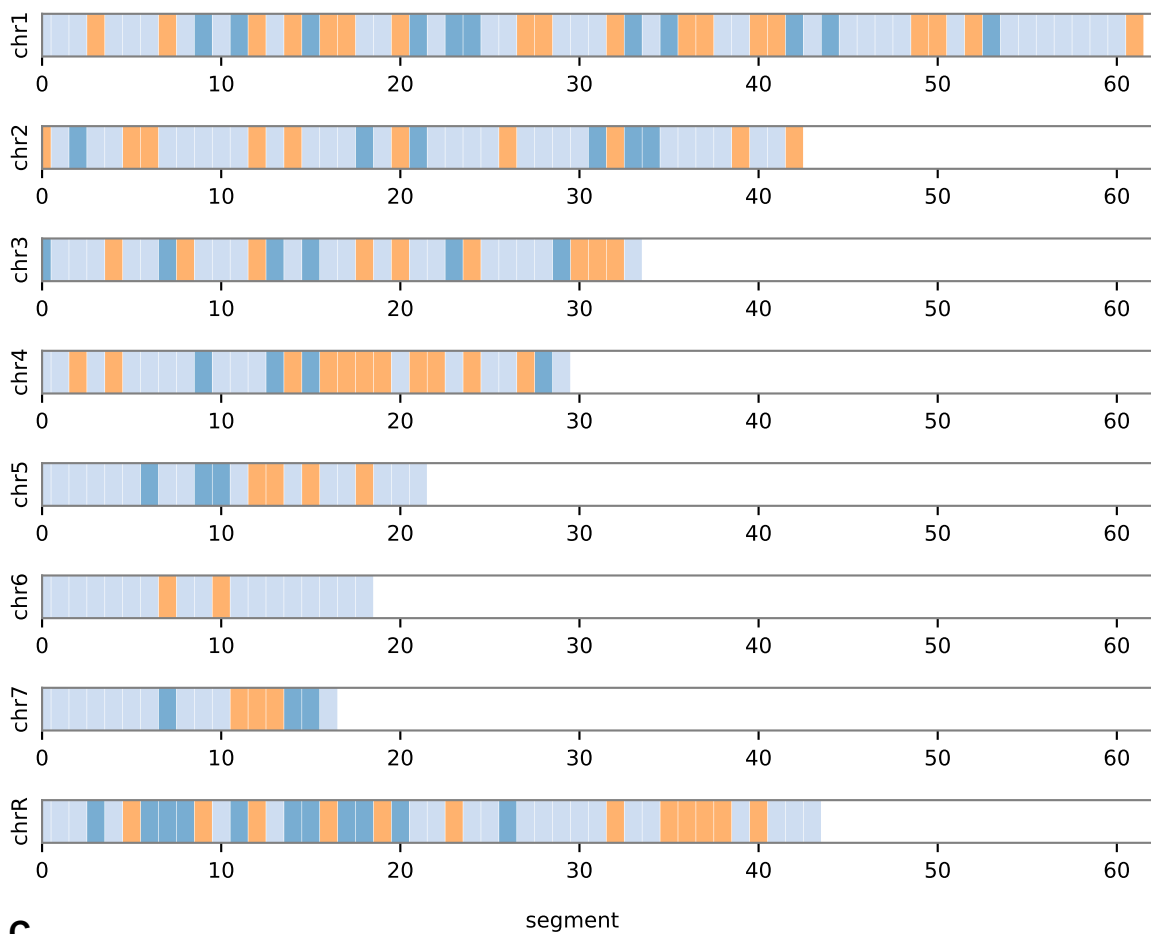

Supplement: Supplementary file 1 [file life-12-00541-s001.zip › life-1592845-supplementary/Heermann-results.pdf]

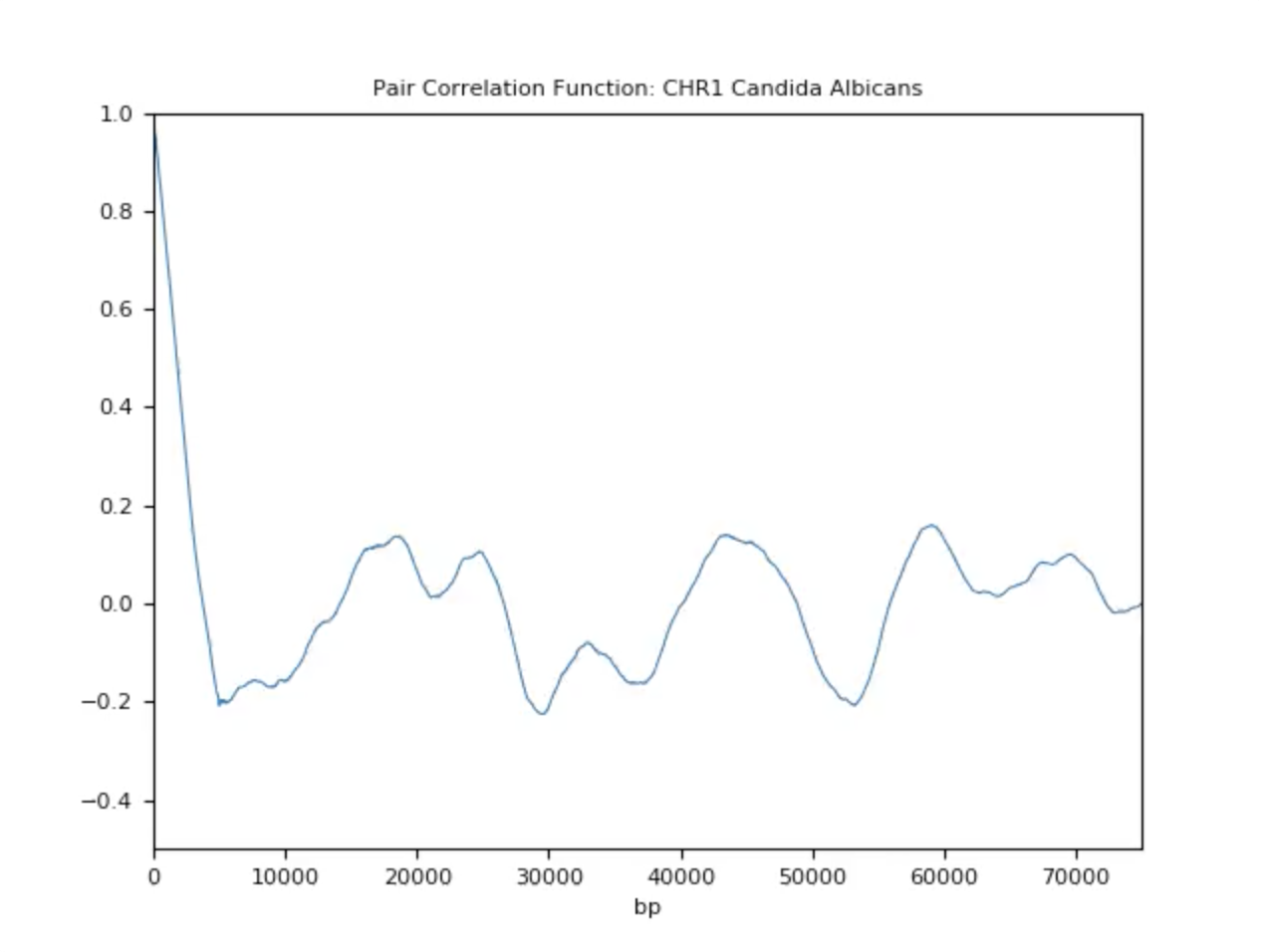

Supplement: Supplementary file 1 [file life-12-00541-s001.zip › life-1592845-supplementary/Heermann_basic_animation.png]

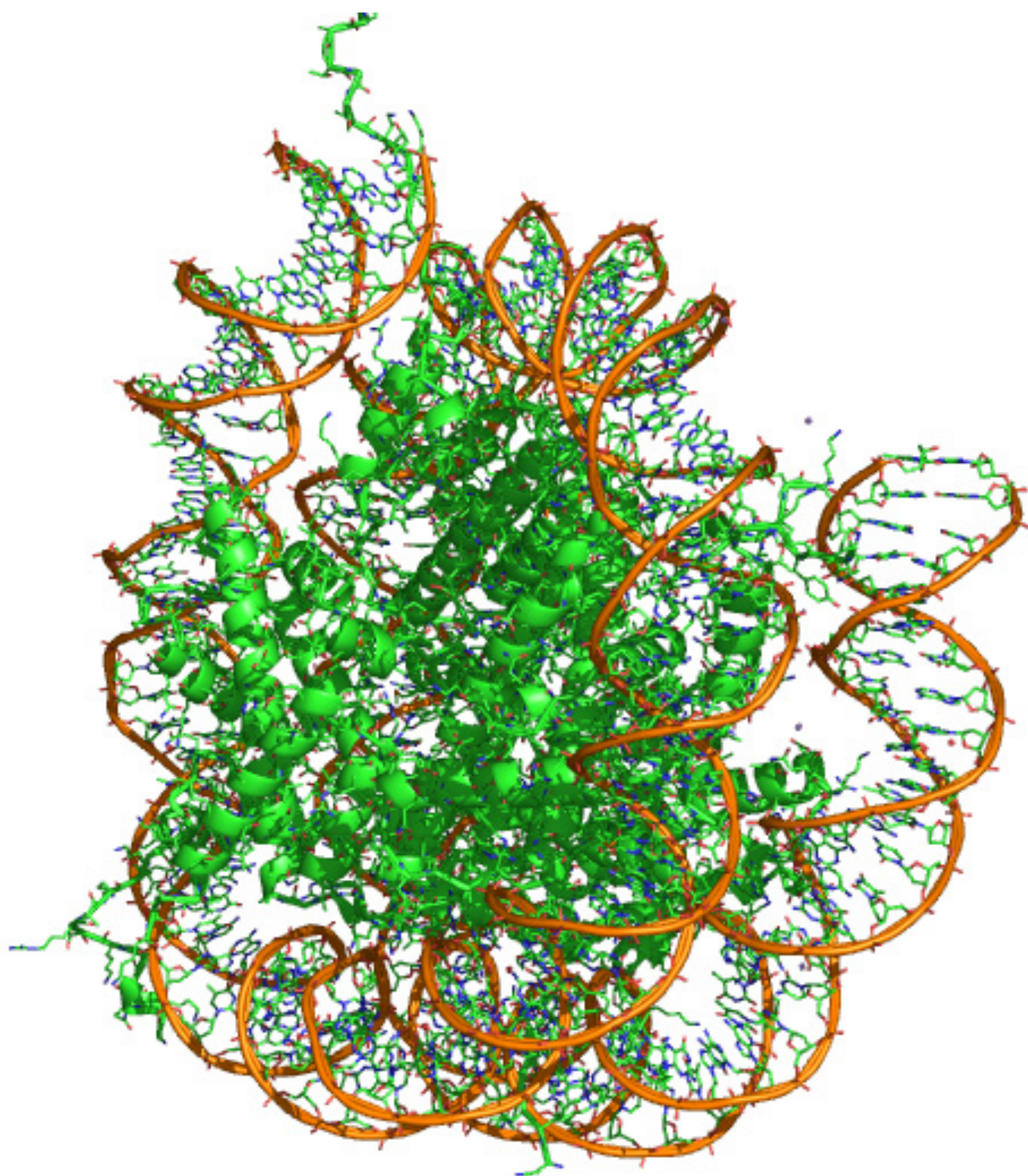

Supplement: Supplementary file 1 [file life-12-00541-s001.zip › life-1592845-supplementary/nucleosome-atomistic.pdf.pdf]

$\|\cdot\|_2$

chr1

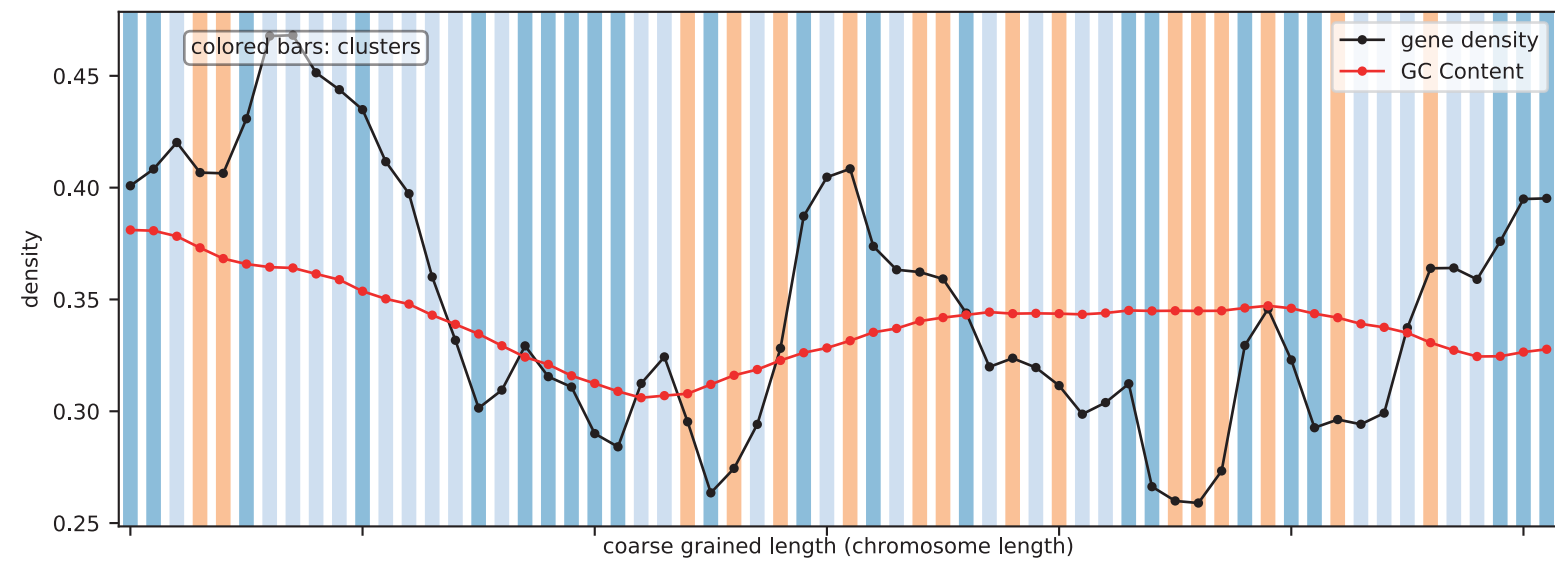

$\|\cdot\|_7$

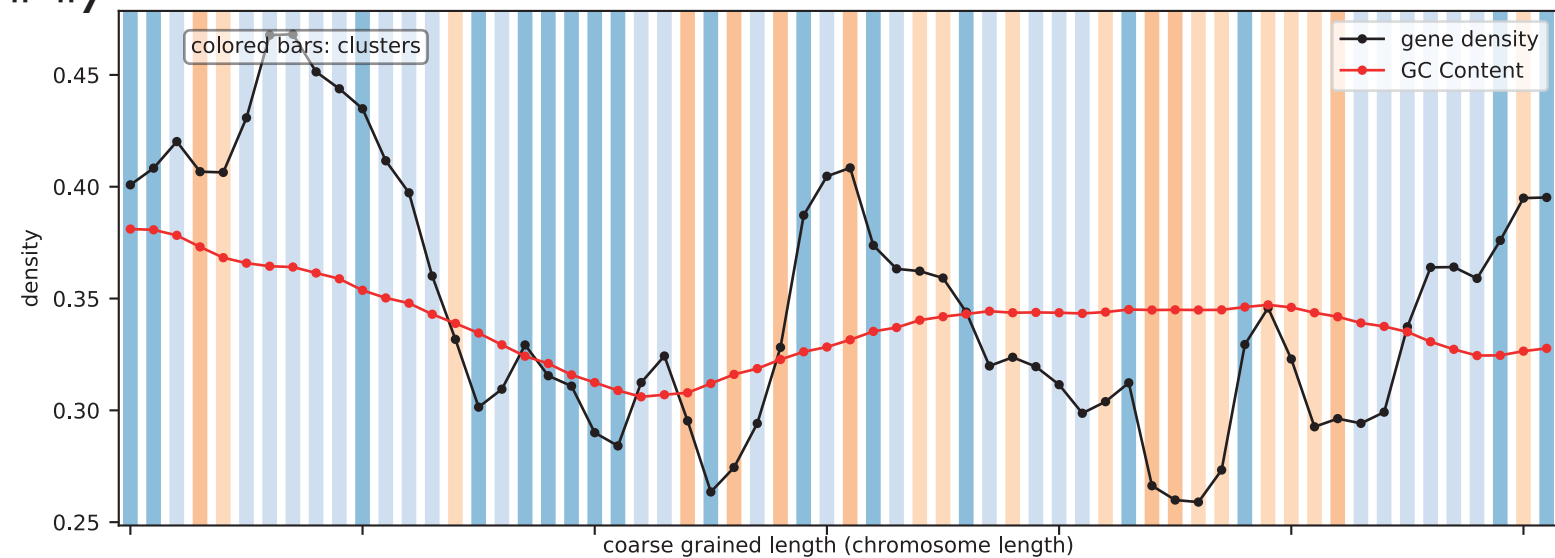

$\cos$

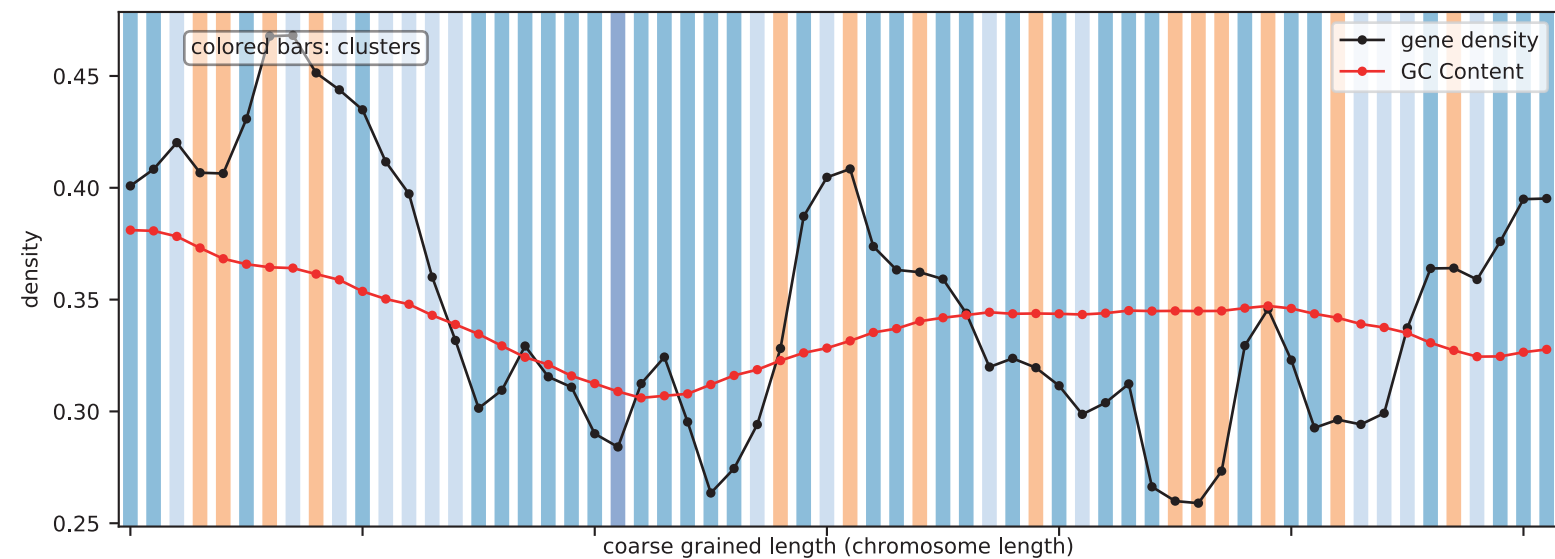

Supplement: Supplementary file 1 [file life-12-00541-s001.zip › life-1592845-supplementary/S1_Heermann-comparison-metrics.pdf]
