# Supplementary figures and images for: Superstructure Detection in Nucleosome Distribution Shows Common Pattern within a Chromosome and within the Genome (part 1 of 2)
Source: Life (Basel). 2022 Apr 6;12(4):541. doi: 10.3390/life12040541 (PMC9026121; doi:10.3390/life12040541)

Correlation and Cluster Analysis for Chromosomes chr1-chr2-chr3-chr4-chr5-chr6-chr7-chrR

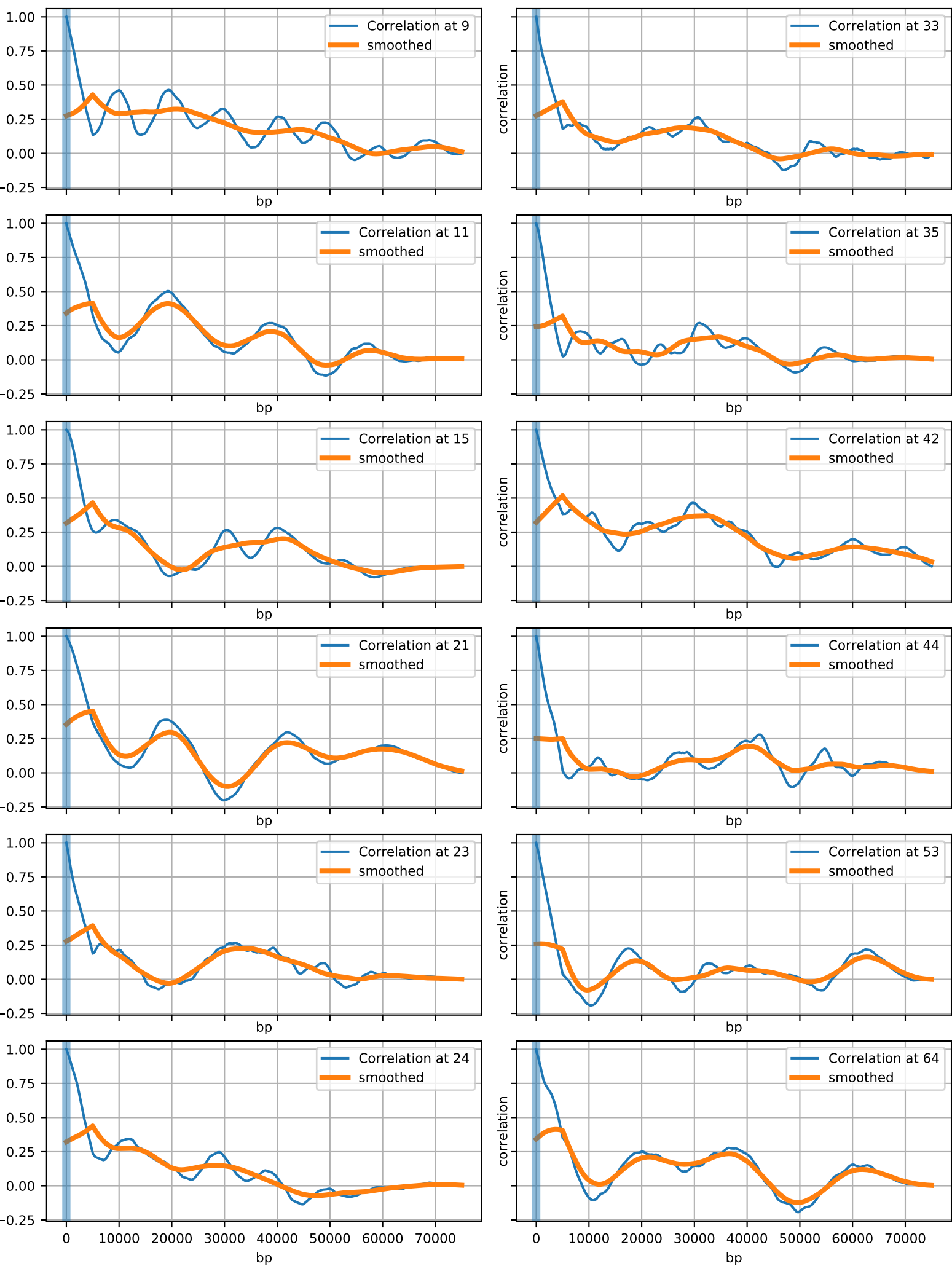

Supplement: Supplementary file 1 [file life-12-00541-s001.zip › life-1592845-supplementary/Heermann-cluster-correlation-function-1f77b4-chr1-chr2-chr3-chr4-chr5-chr6-chr7-chrR-1.pdf]

Correlation and Cluster Analysis for Chromosomes chr1-chr2-chr3-chr4-chr5-chr6-chr7-chrR

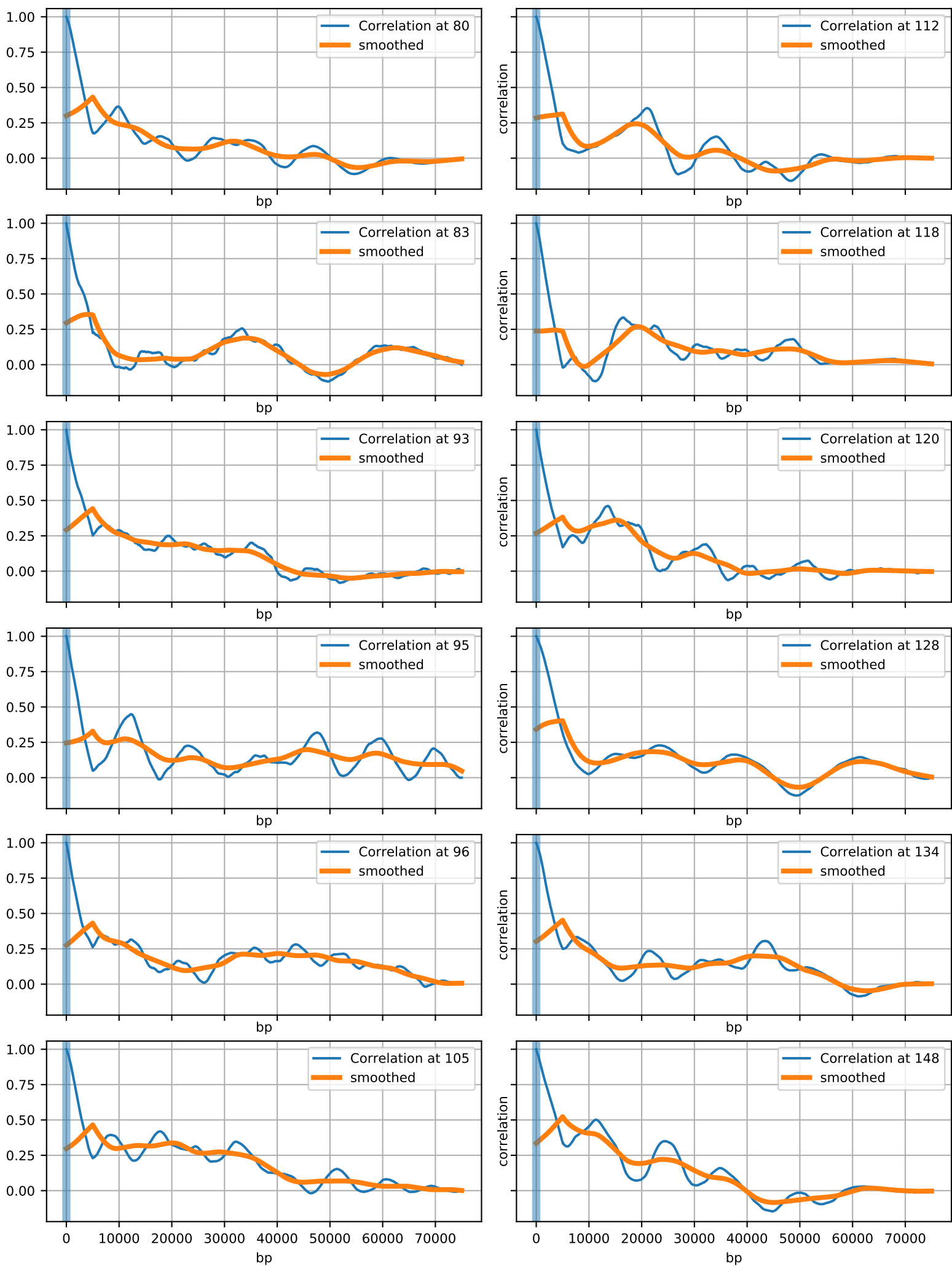

Supplement: Supplementary file 1 [file life-12-00541-s001.zip › life-1592845-supplementary/Heermann-cluster-correlation-function-1f77b4-chr1-chr2-chr3-chr4-chr5-chr6-chr7-chrR-2.pdf]

Correlation and Cluster Analysis for Chromosomes chr1-chr2-chr3-chr4-chr5-chr6-chr7-chrR

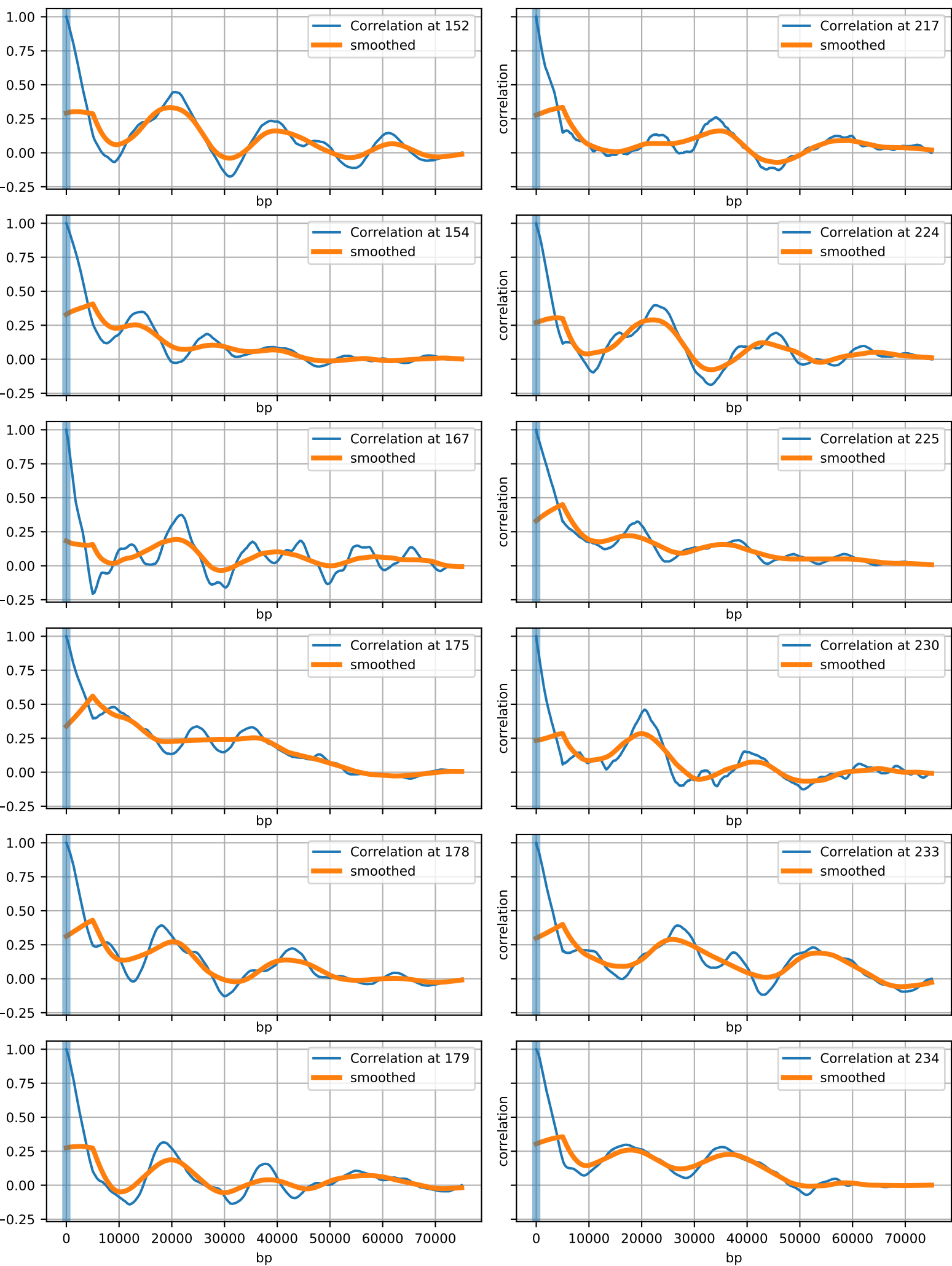

Supplement: Supplementary file 1 [file life-12-00541-s001.zip › life-1592845-supplementary/Heermann-cluster-correlation-function-1f77b4-chr1-chr2-chr3-chr4-chr5-chr6-chr7-chrR-3.pdf]

Correlation and Cluster Analysis for Chromosomes chr1-chr2-chr3-chr4-chr5-chr6-chr7-chrR

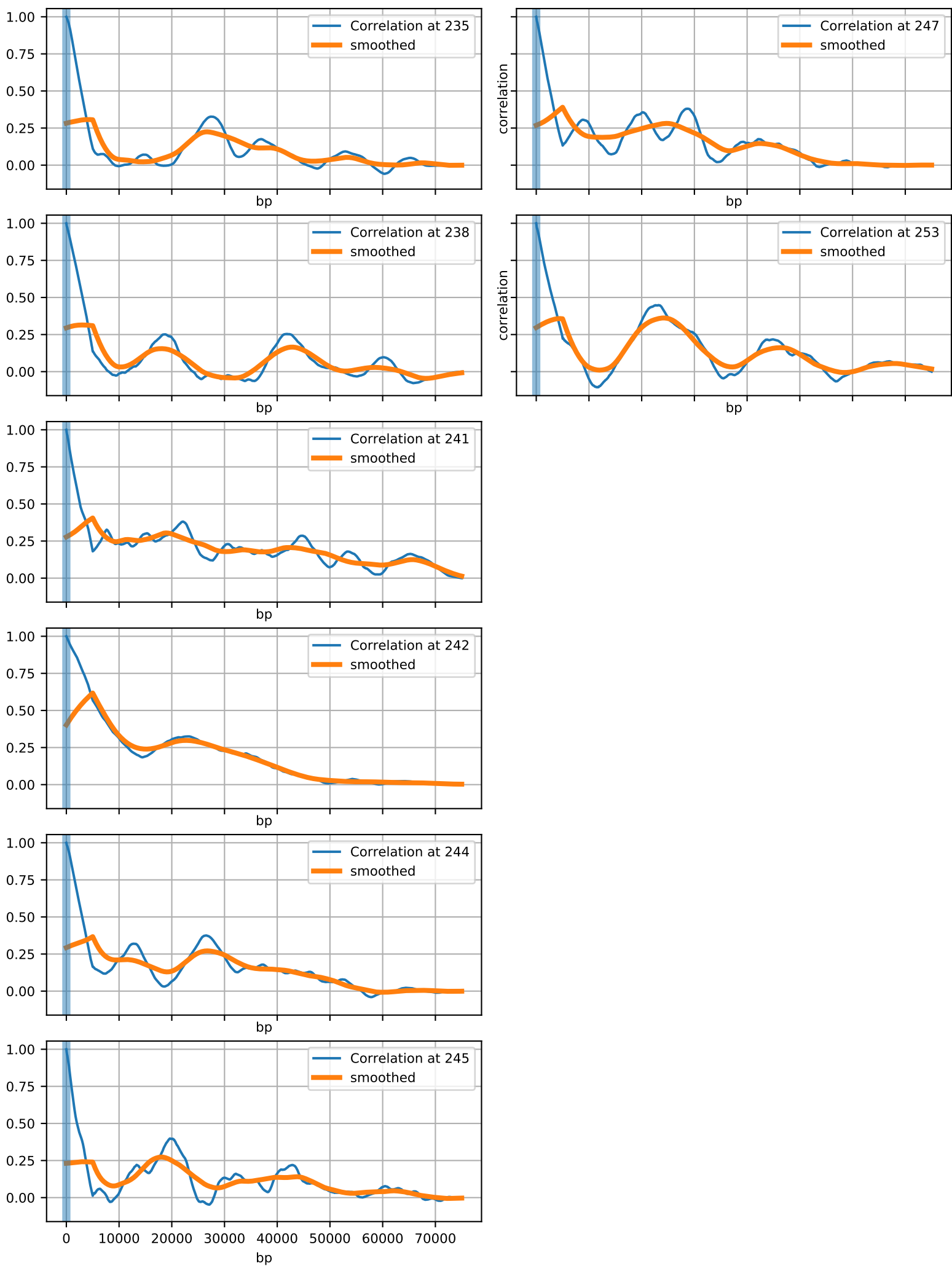

Supplement: Supplementary file 1 [file life-12-00541-s001.zip › life-1592845-supplementary/Heermann-cluster-correlation-function-1f77b4-chr1-chr2-chr3-chr4-chr5-chr6-chr7-chrR-4.pdf]

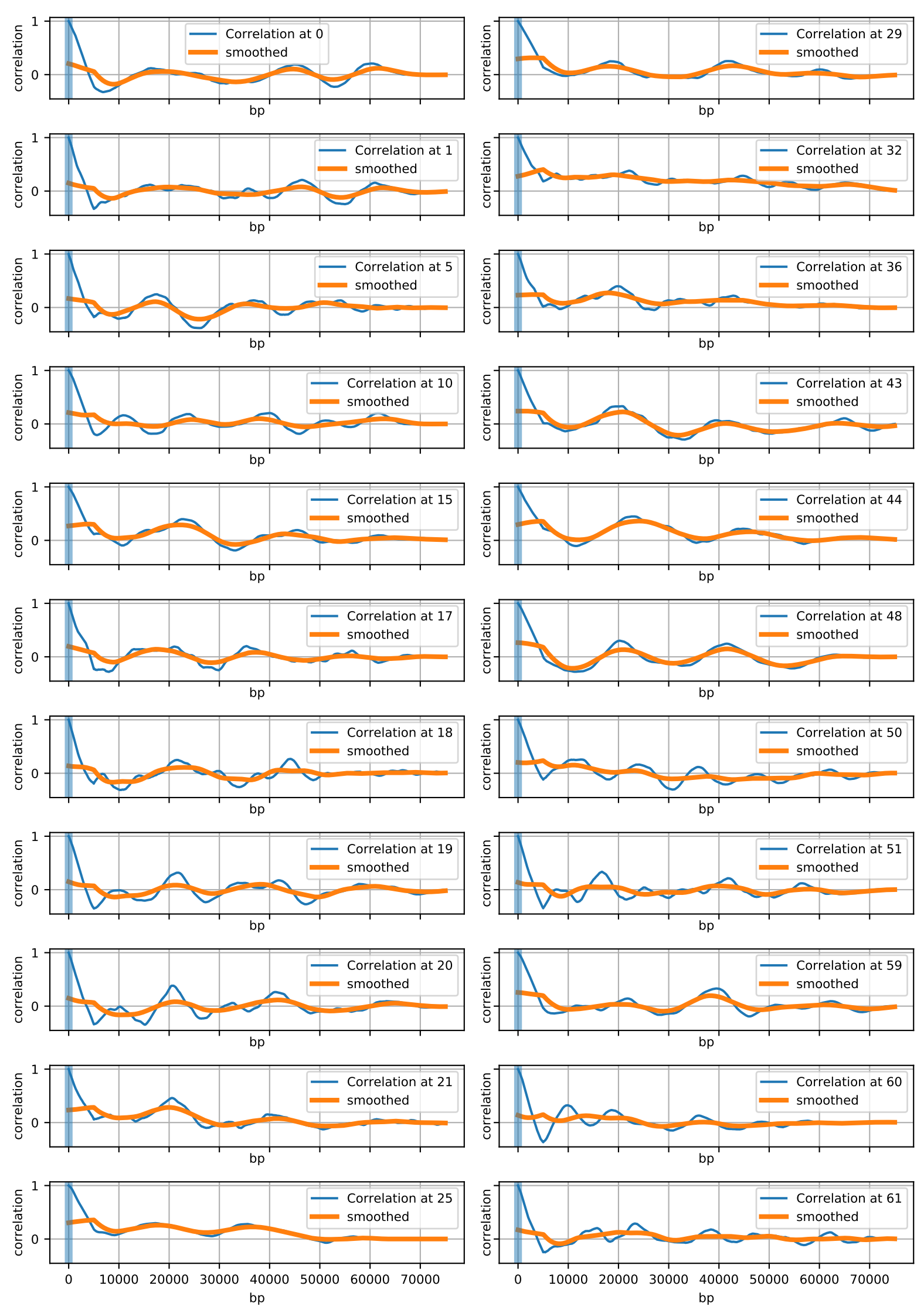

Supplement: Supplementary file 1 [file life-12-00541-s001.zip › life-1592845-supplementary/Heermann-cluster-correlation-function-1f77b4-chr1.pdf]

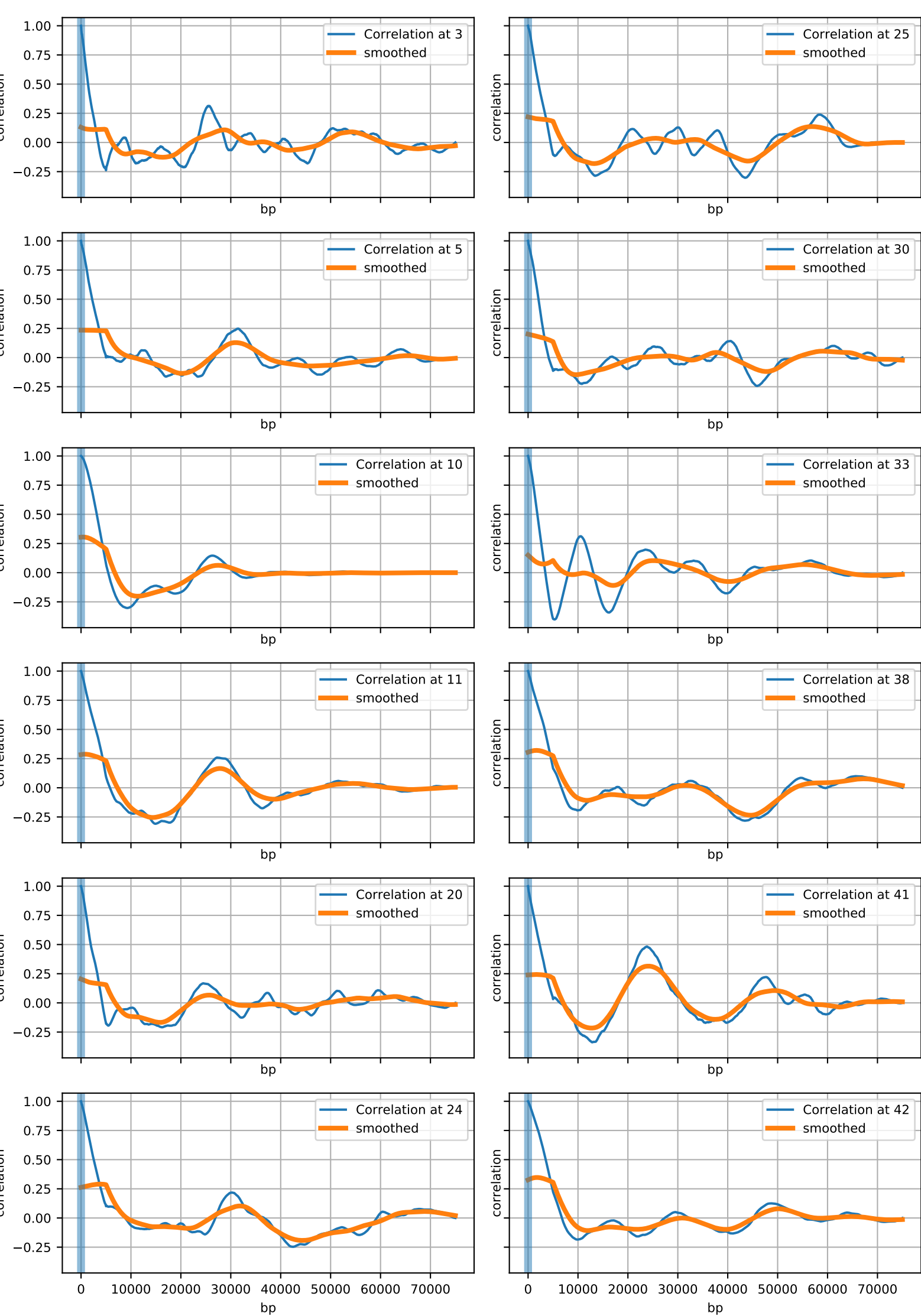

Supplement: Supplementary file 1 [file life-12-00541-s001.zip › life-1592845-supplementary/Heermann-cluster-correlation-function-1f77b4-chr2.pdf]

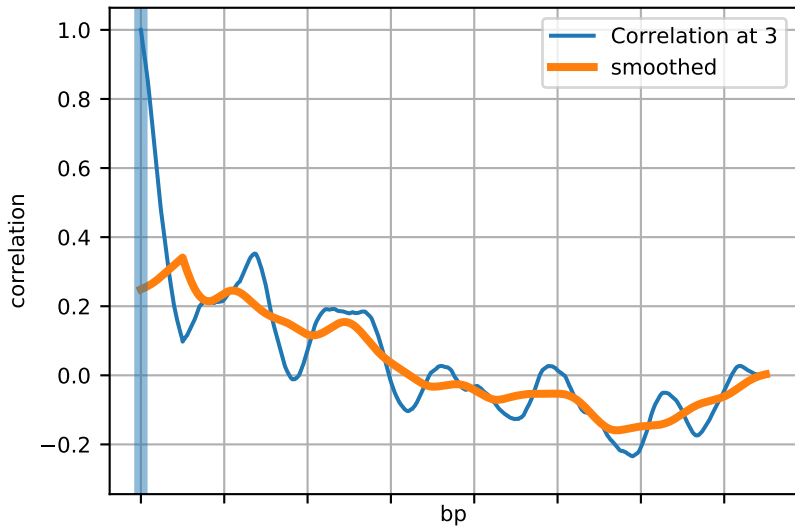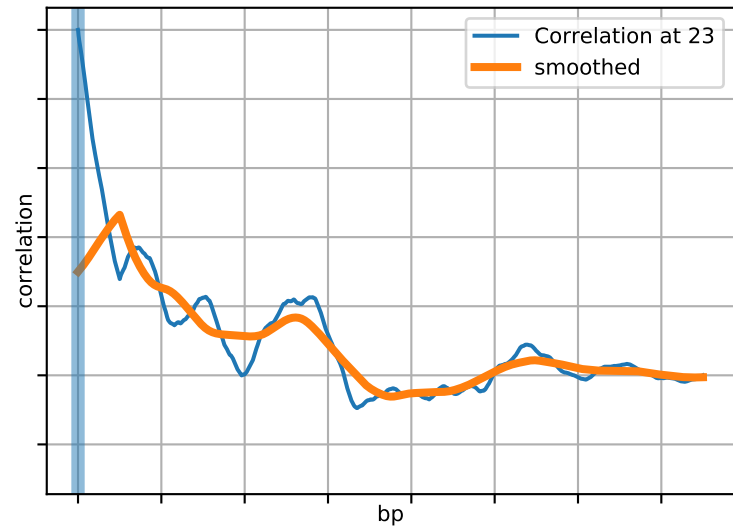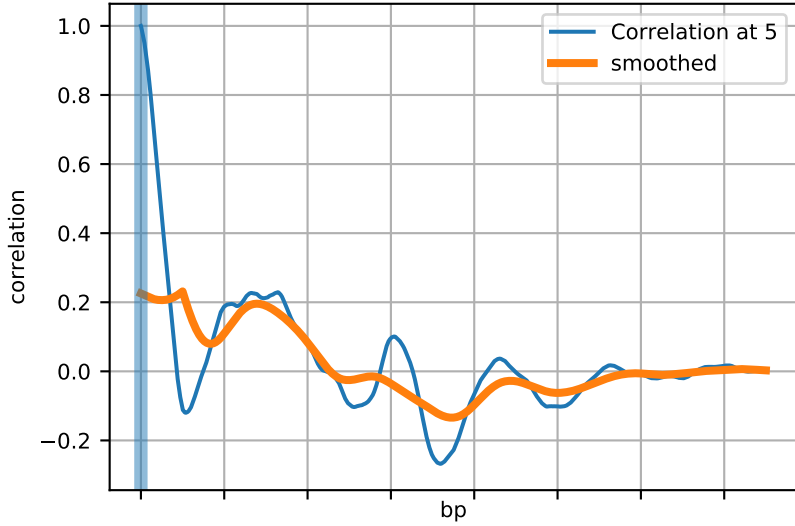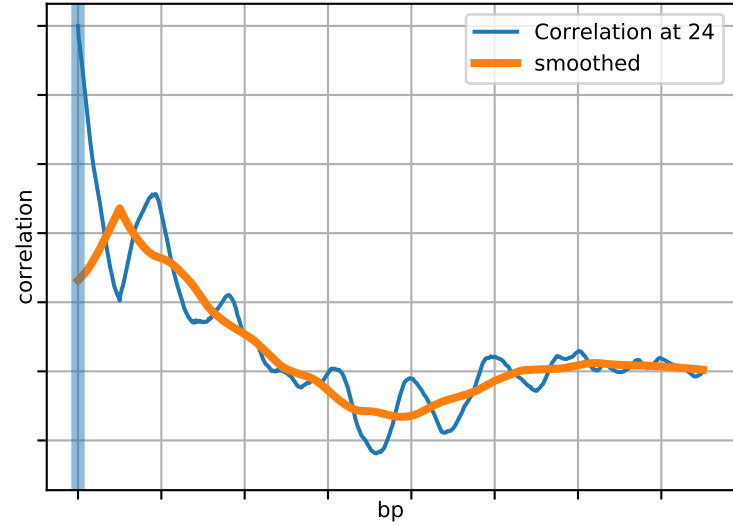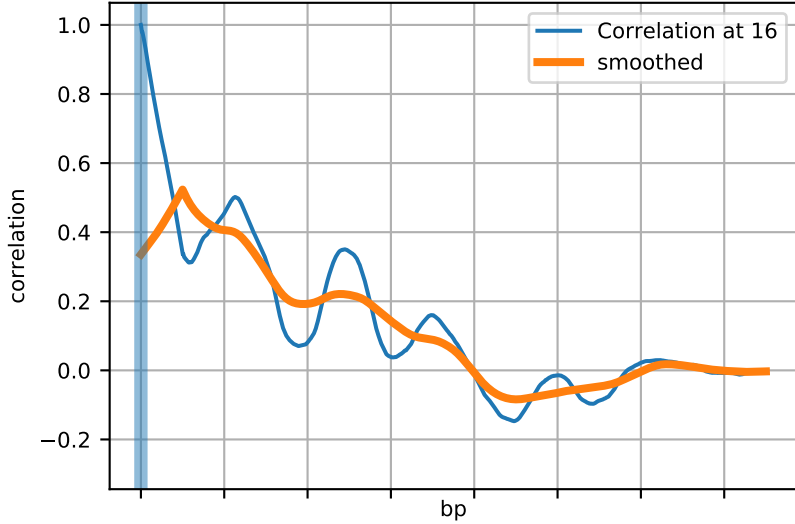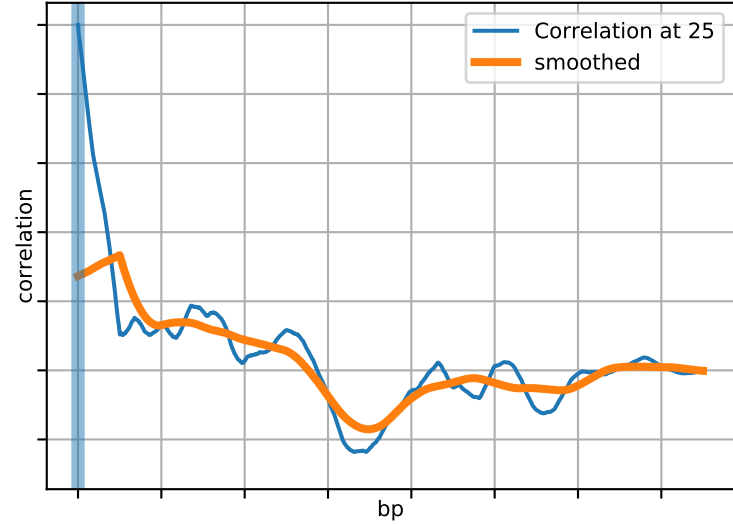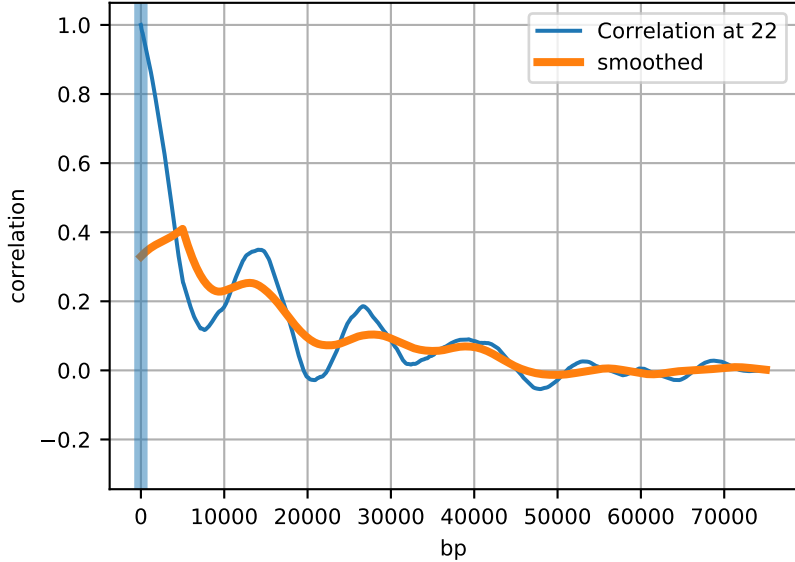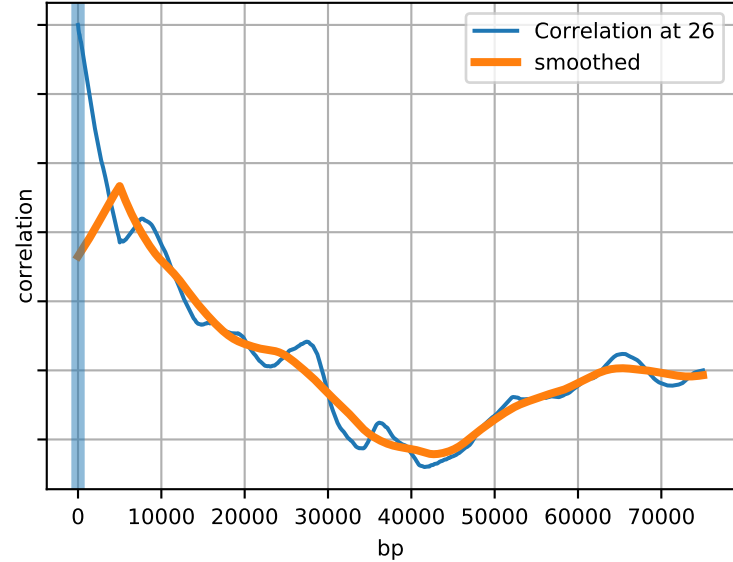

Supplement: Supplementary file 1 [file life-12-00541-s001.zip › life-1592845-supplementary/Heermann-cluster-correlation-function-1f77b4-chr3.pdf]

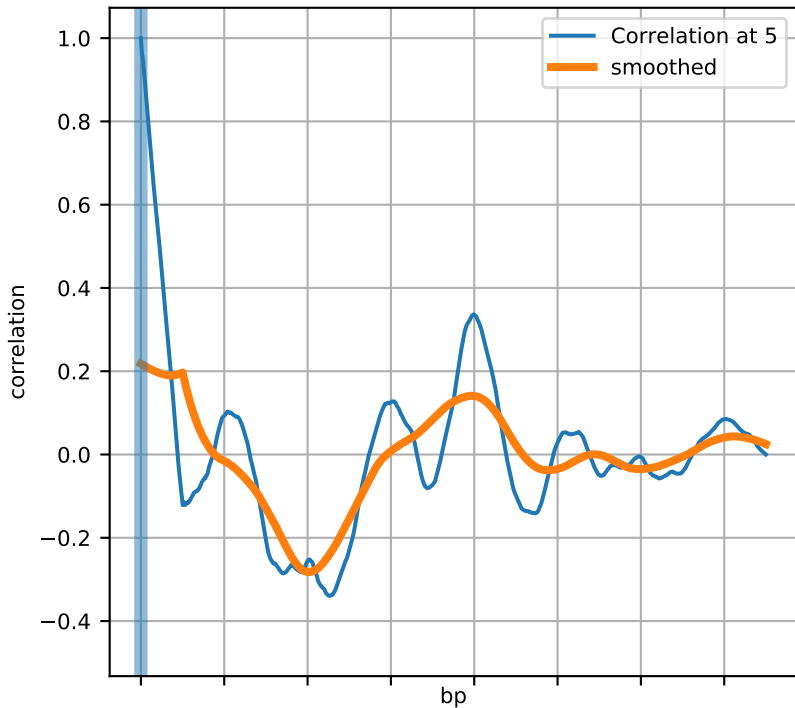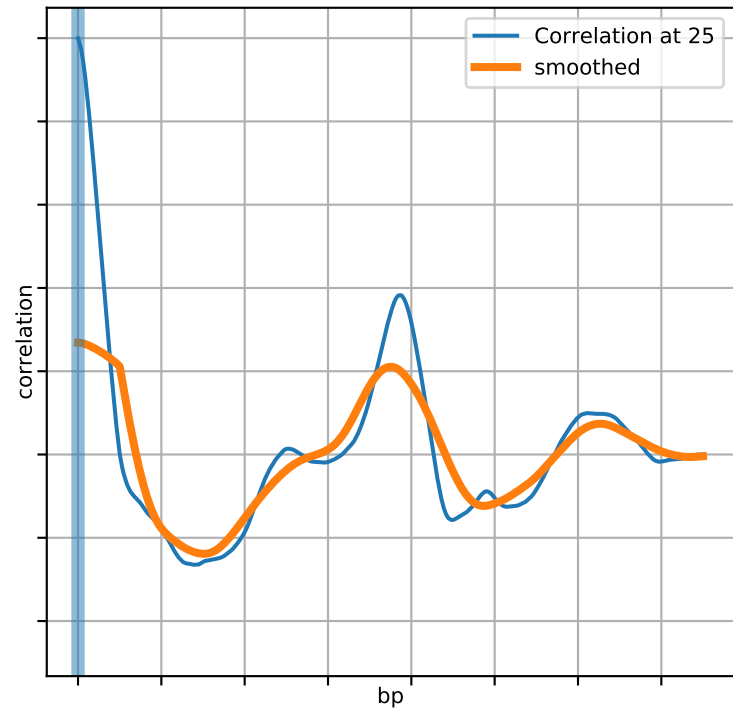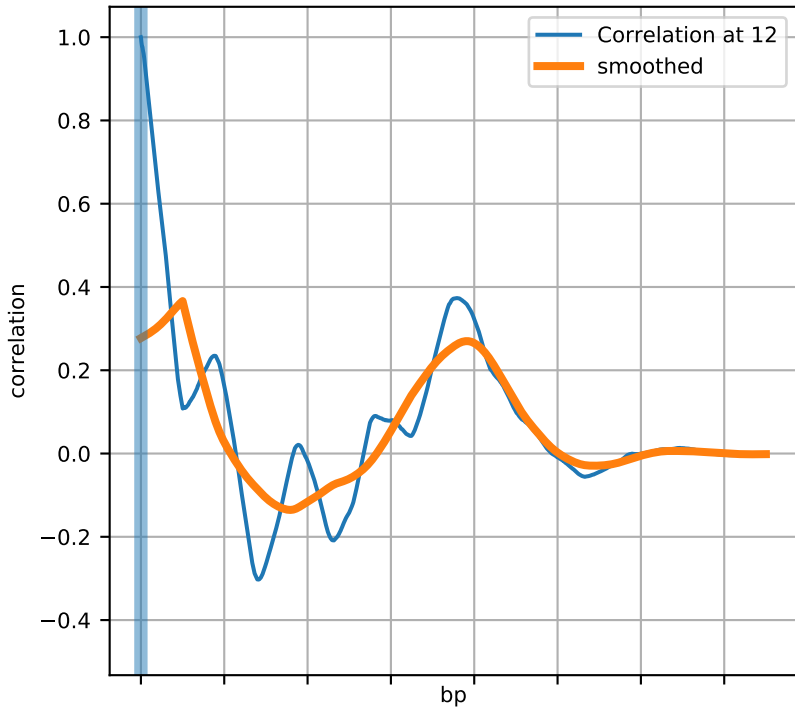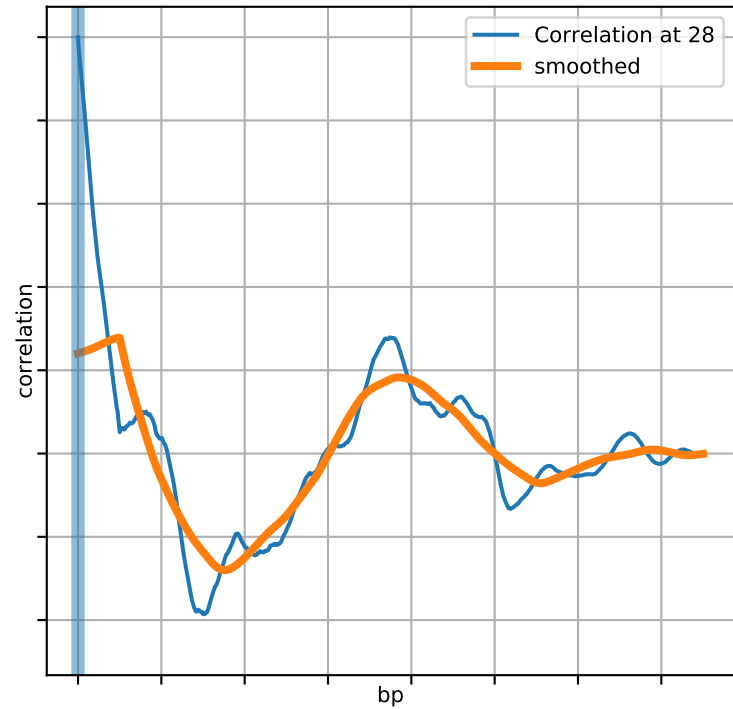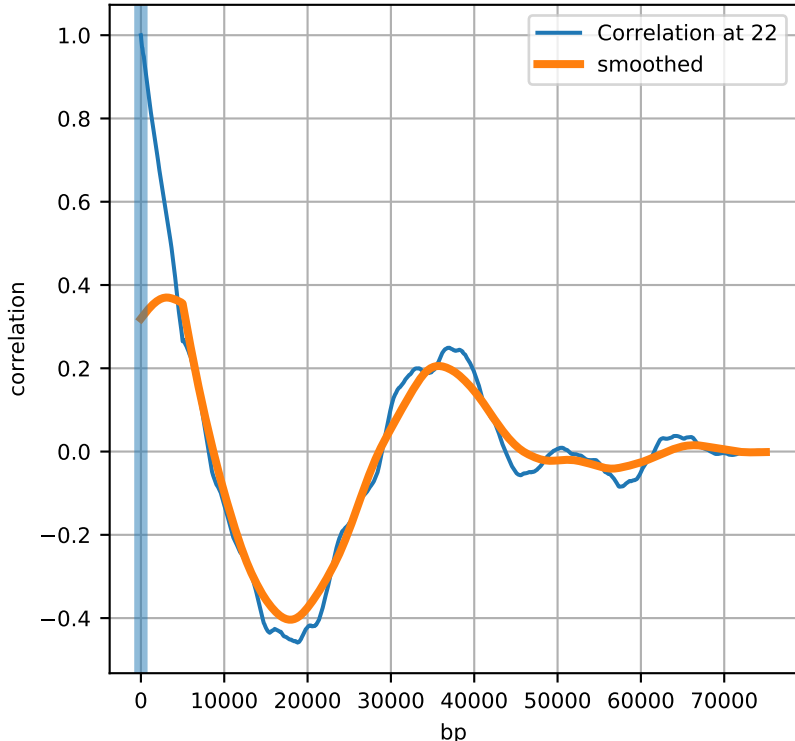

Supplement: Supplementary file 1 [file life-12-00541-s001.zip › life-1592845-supplementary/Heermann-cluster-correlation-function-1f77b4-chr4.pdf]

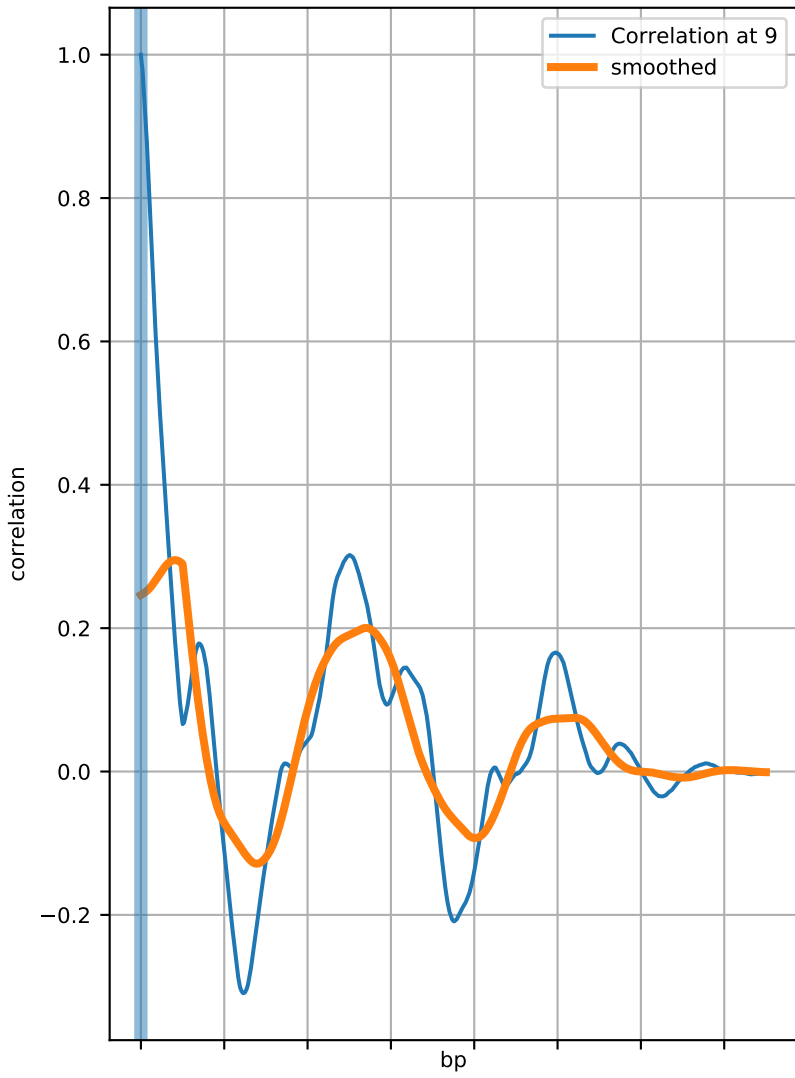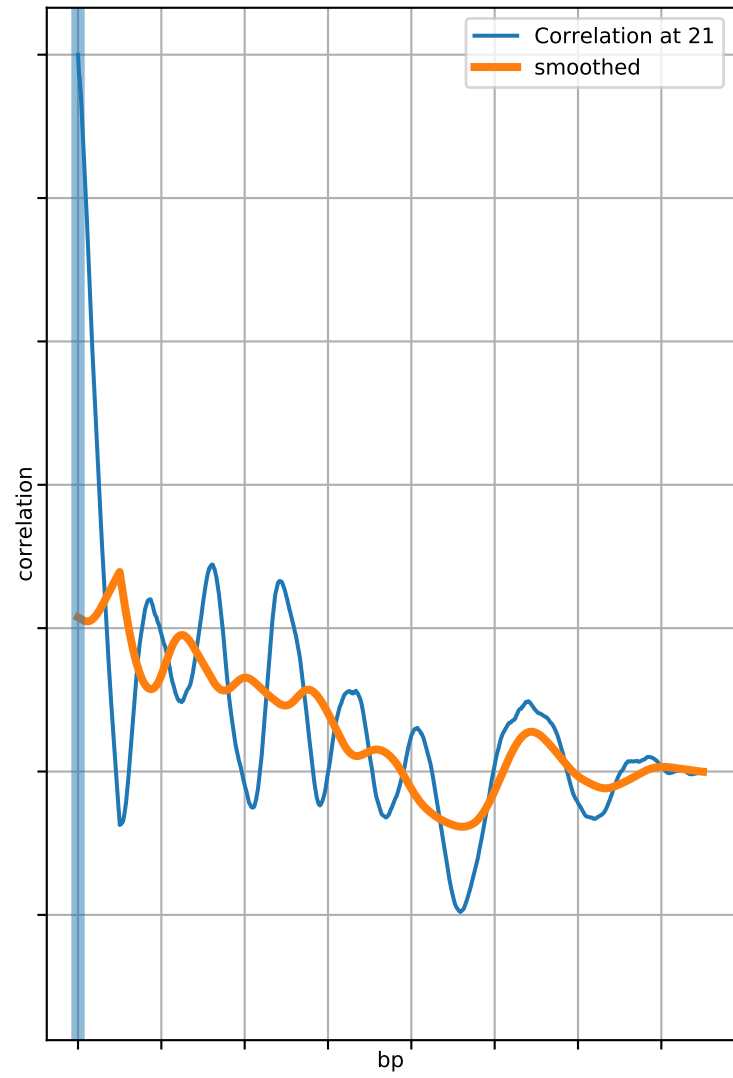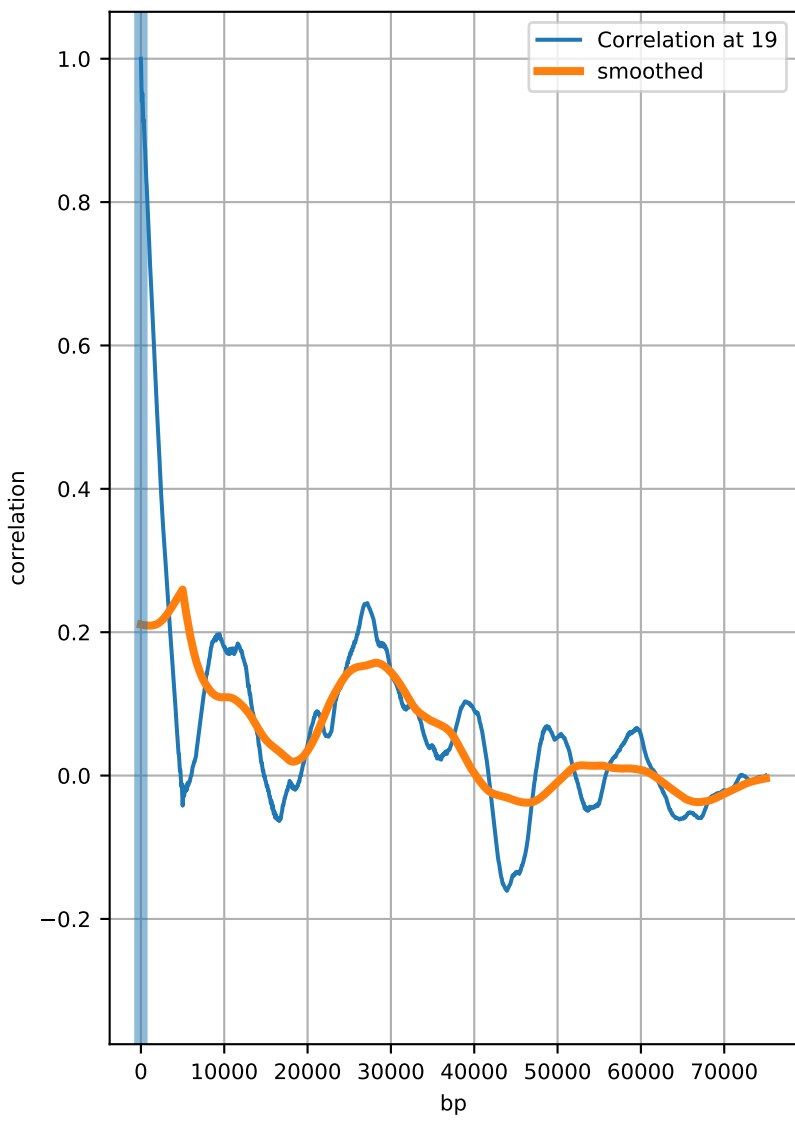

Supplement: Supplementary file 1 [file life-12-00541-s001.zip › life-1592845-supplementary/Heermann-cluster-correlation-function-1f77b4-chr5.pdf]

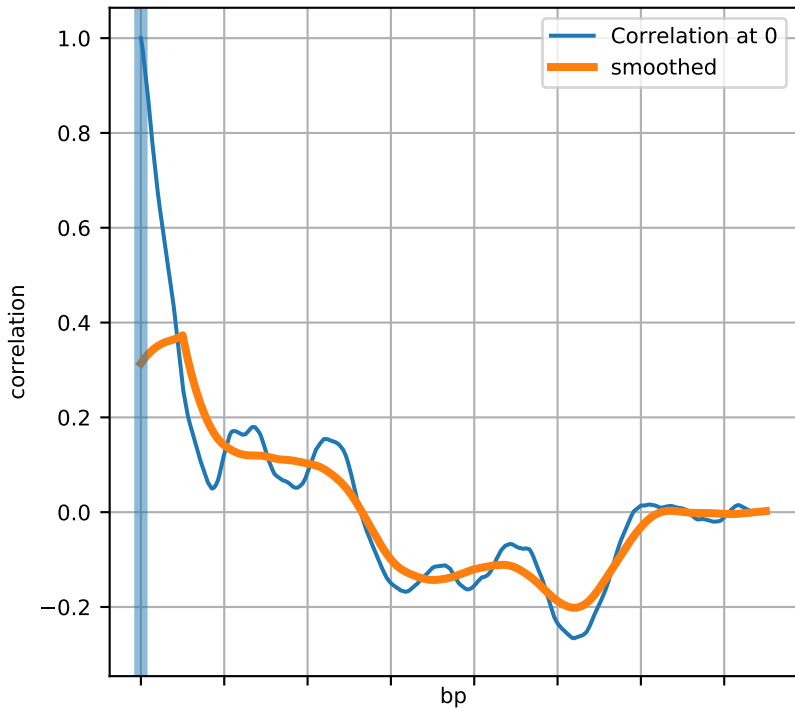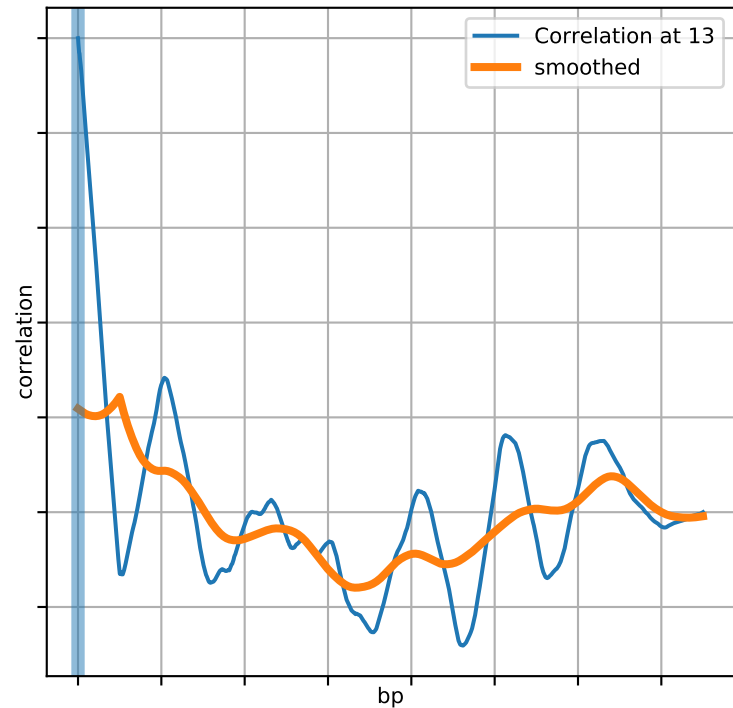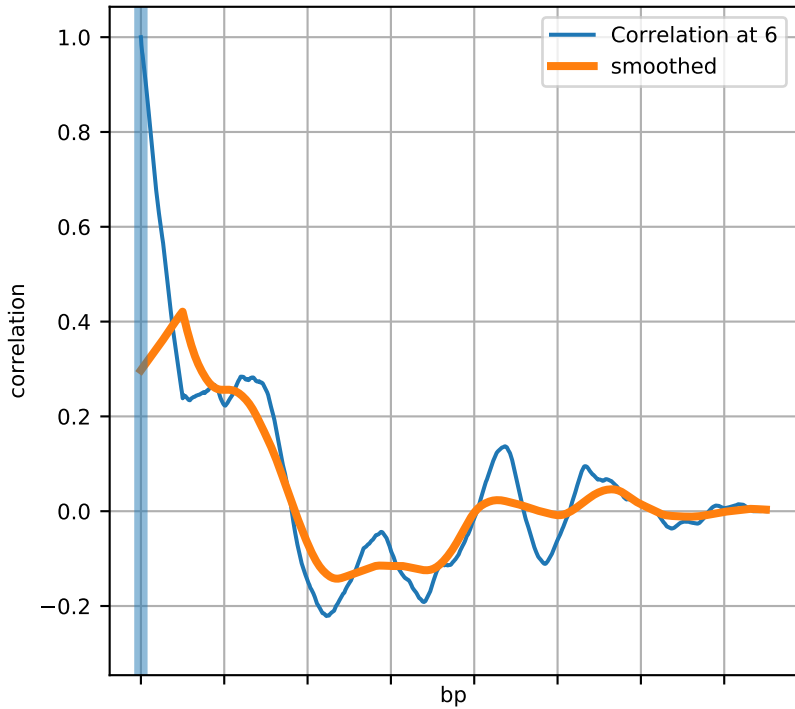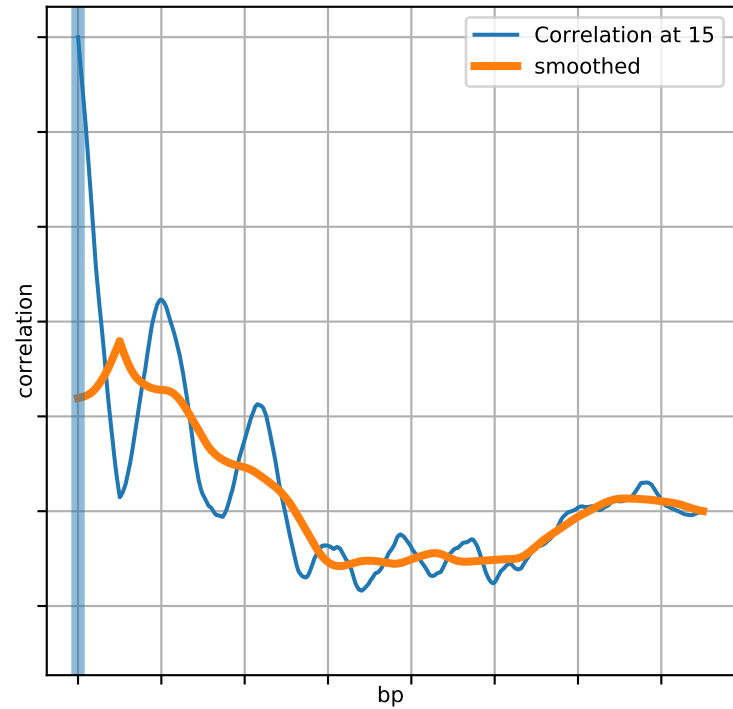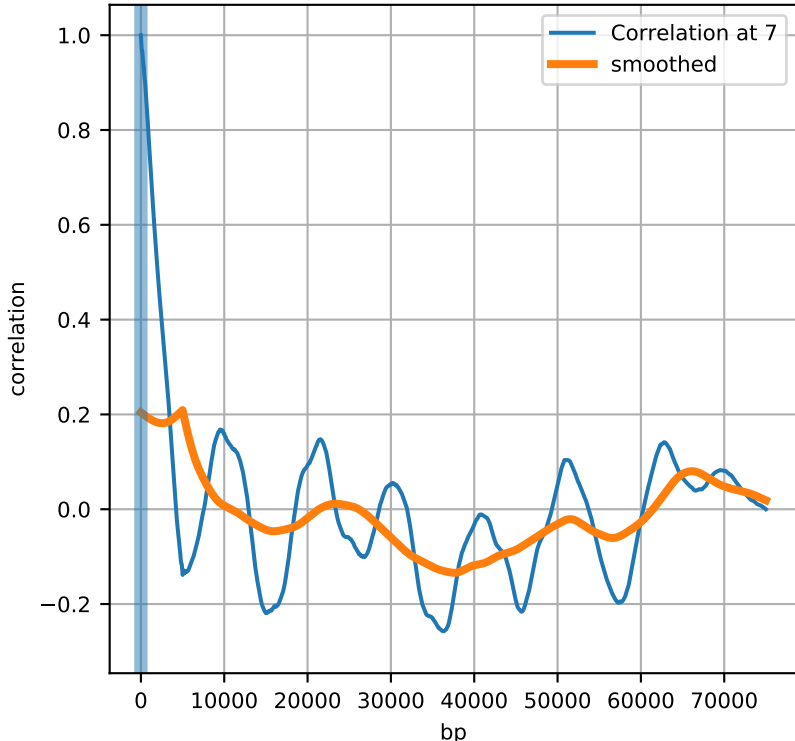

Supplement: Supplementary file 1 [file life-12-00541-s001.zip › life-1592845-supplementary/Heermann-cluster-correlation-function-1f77b4-chr6.pdf]

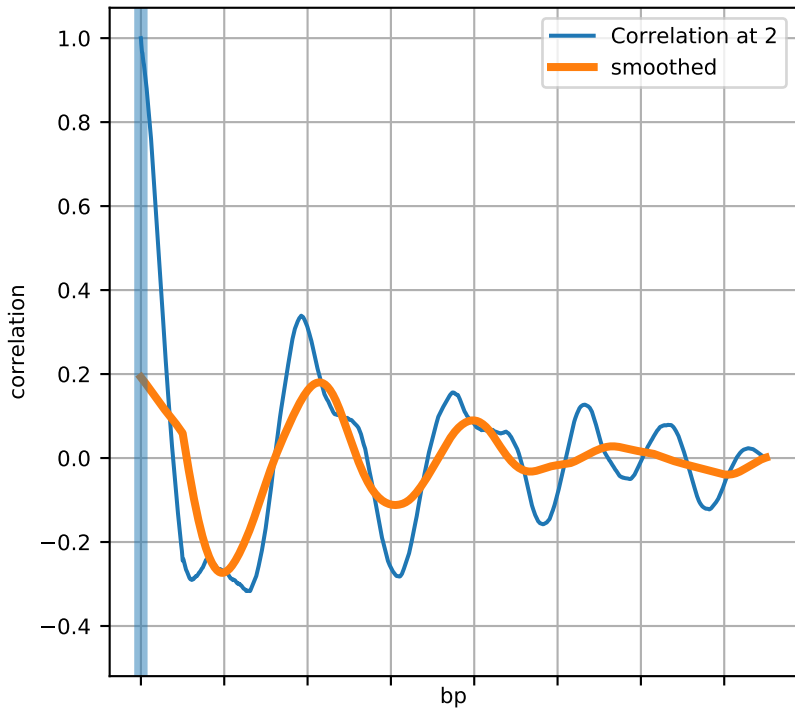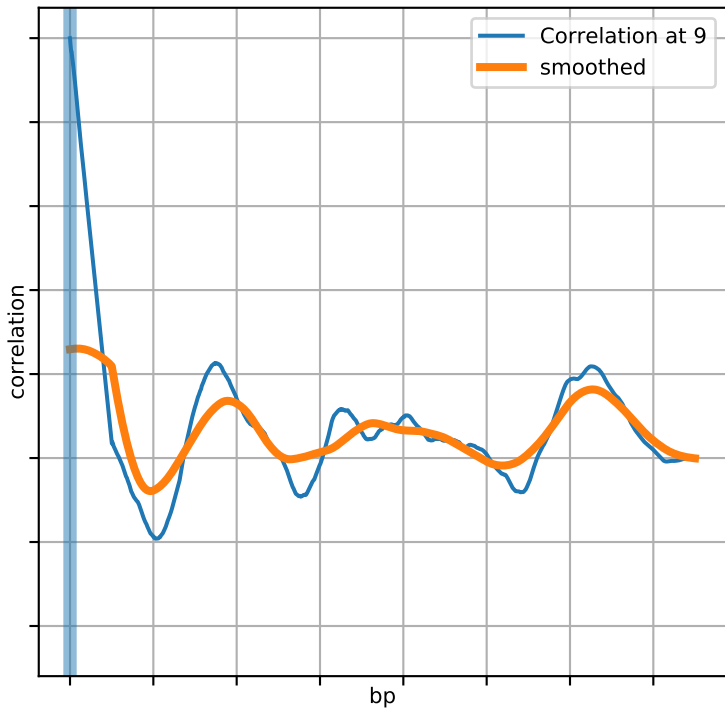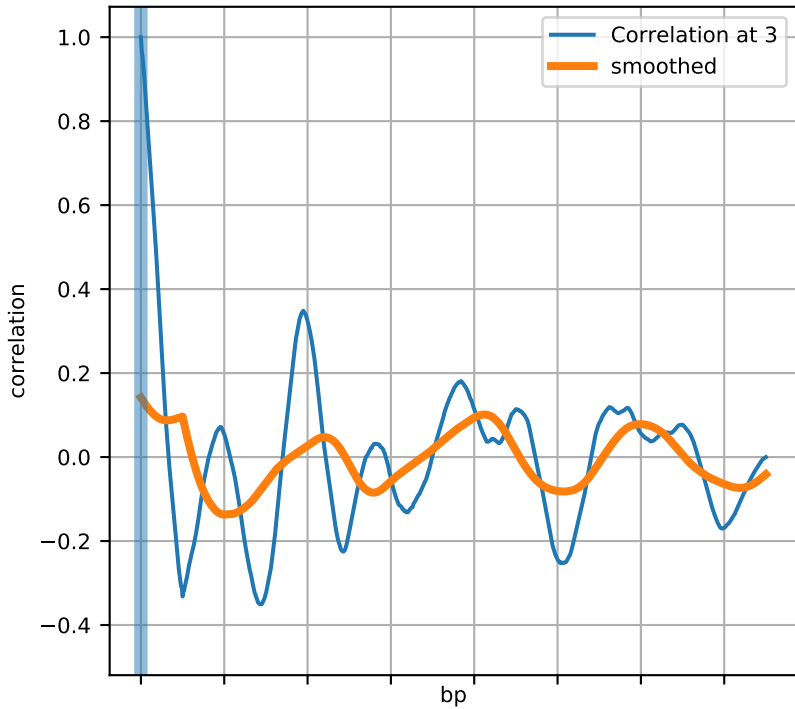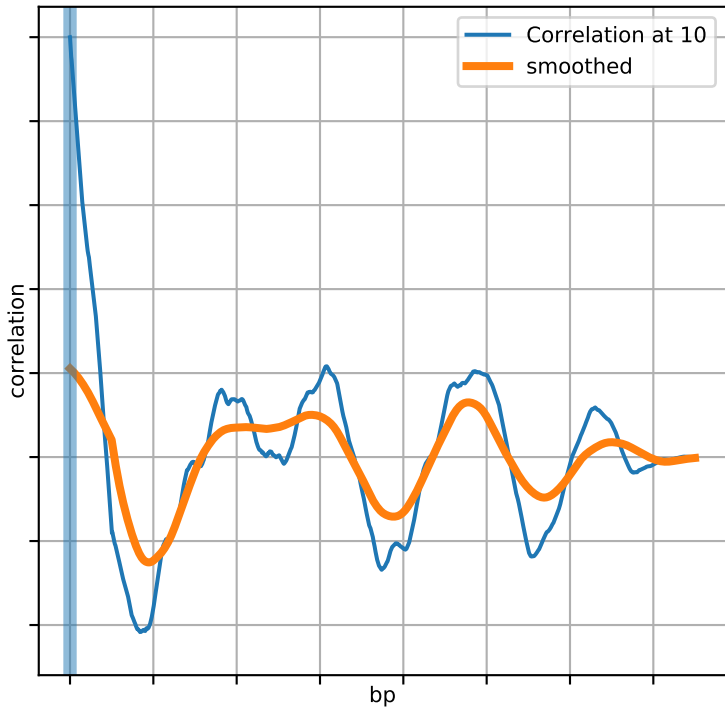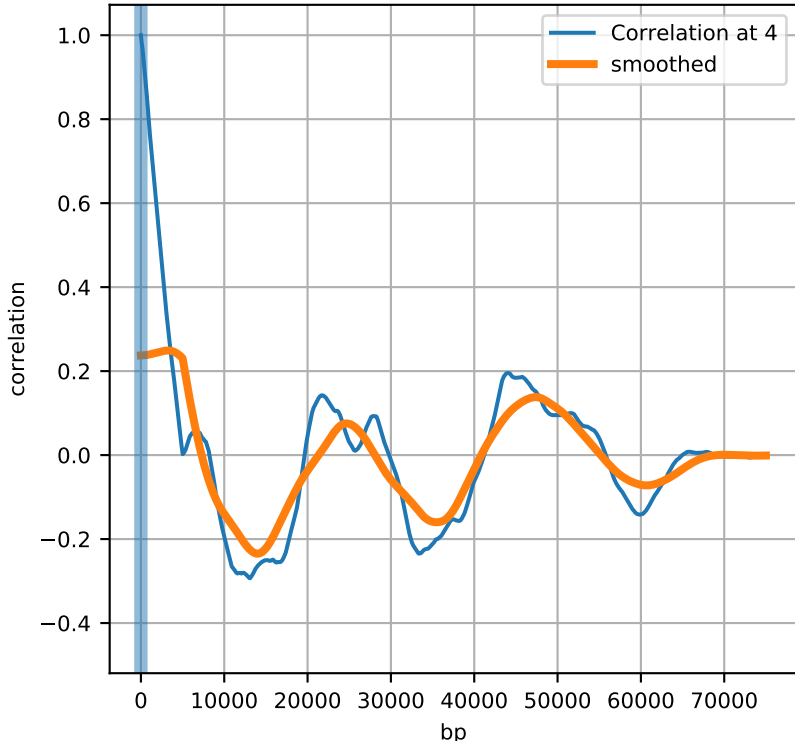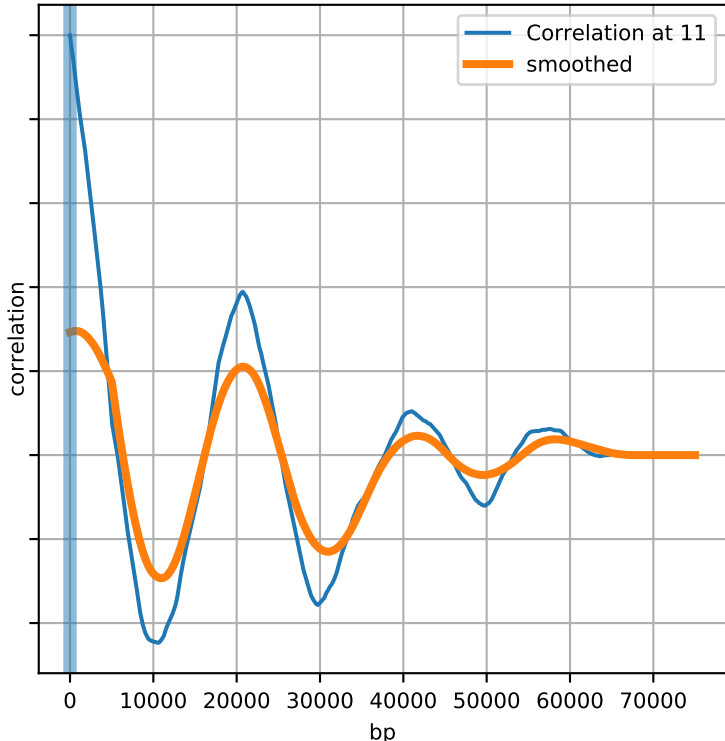

Supplement: Supplementary file 1 [file life-12-00541-s001.zip › life-1592845-supplementary/Heermann-cluster-correlation-function-1f77b4-chr7.pdf]

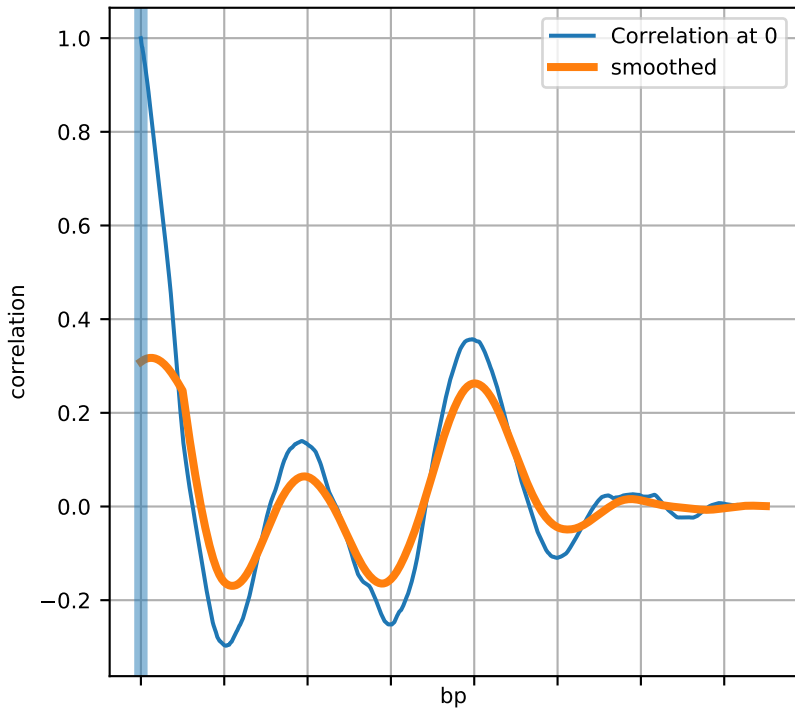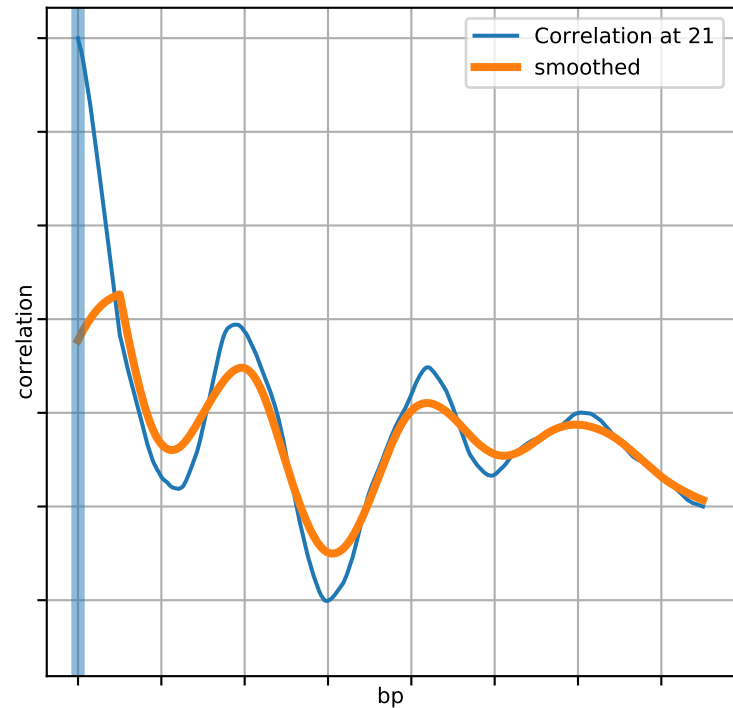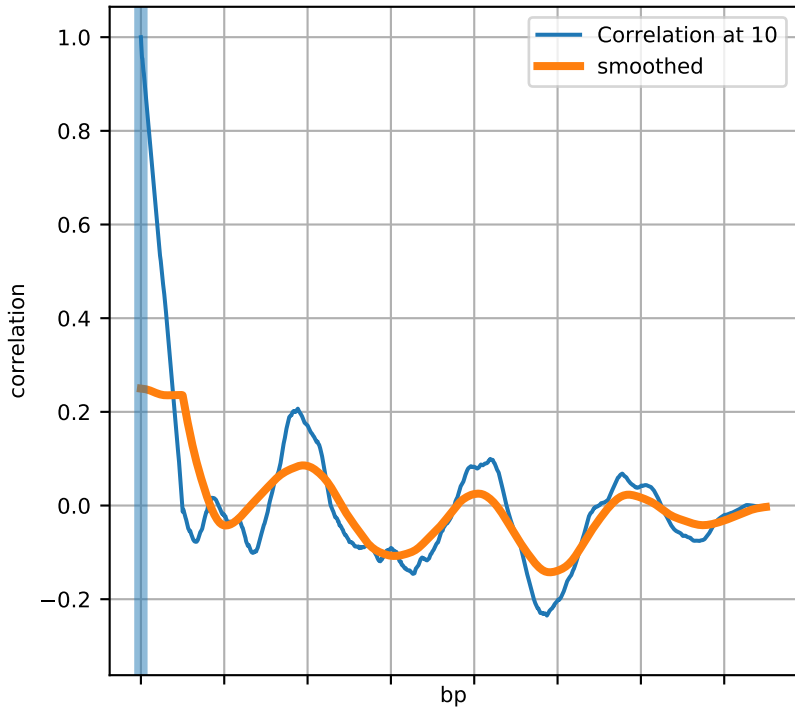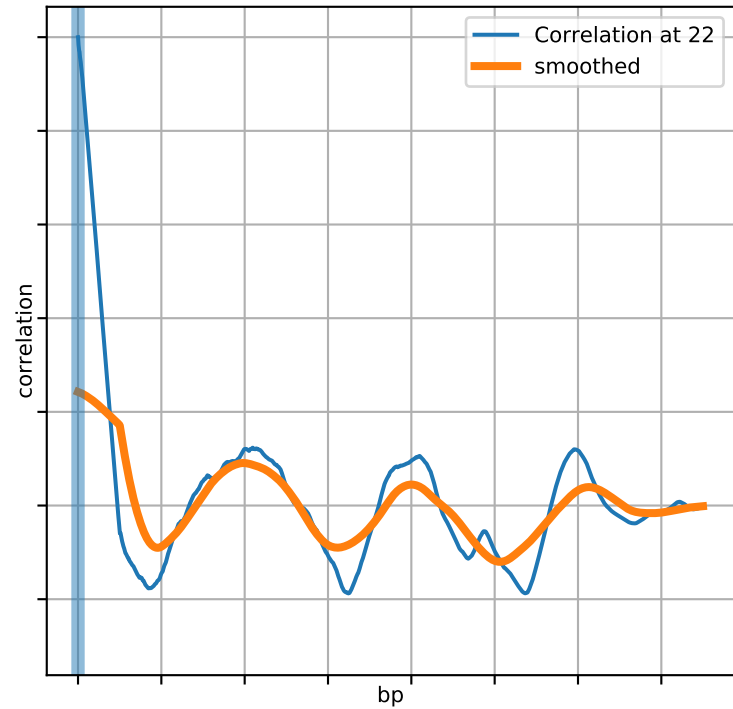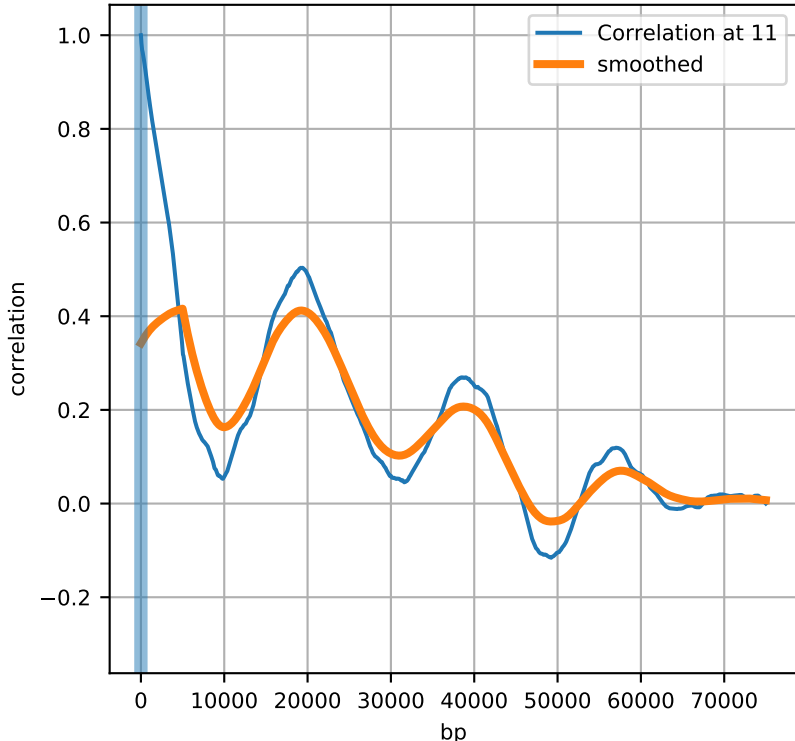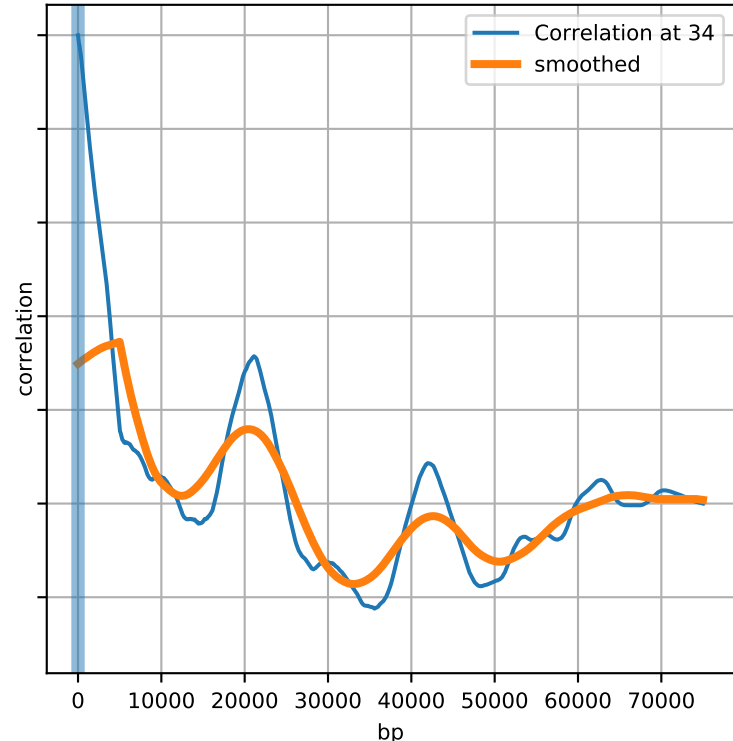

Supplement: Supplementary file 1 [file life-12-00541-s001.zip › life-1592845-supplementary/Heermann-cluster-correlation-function-1f77b4-chrR.pdf]

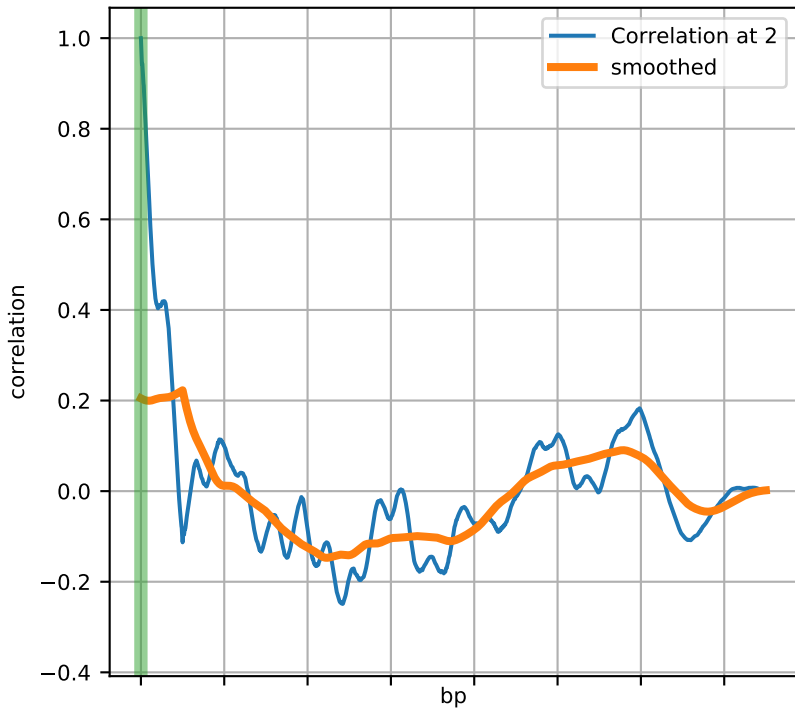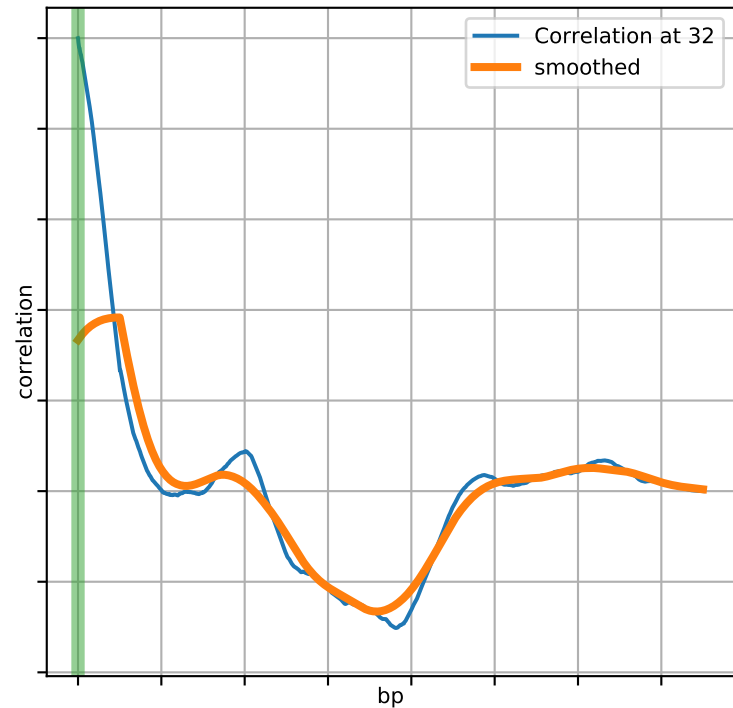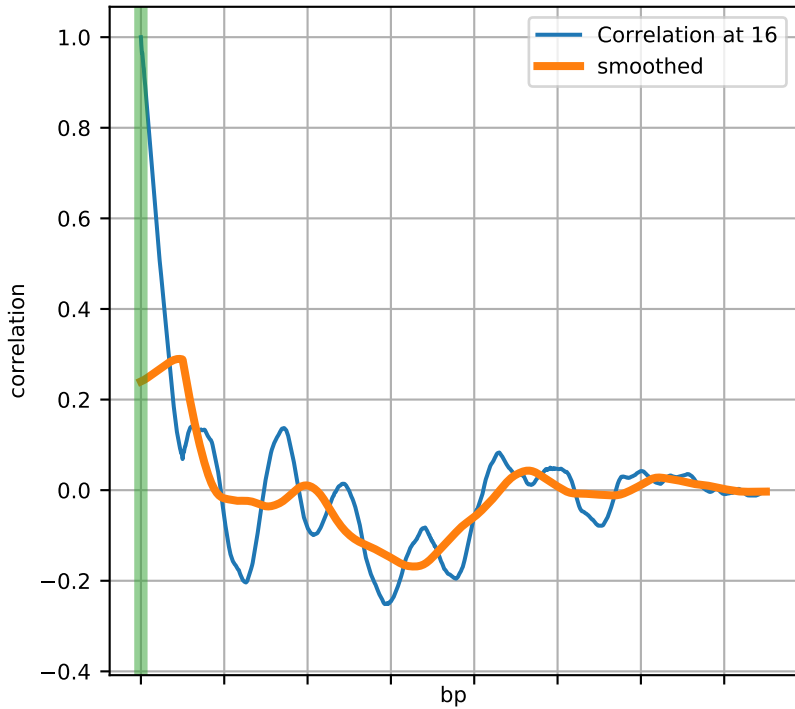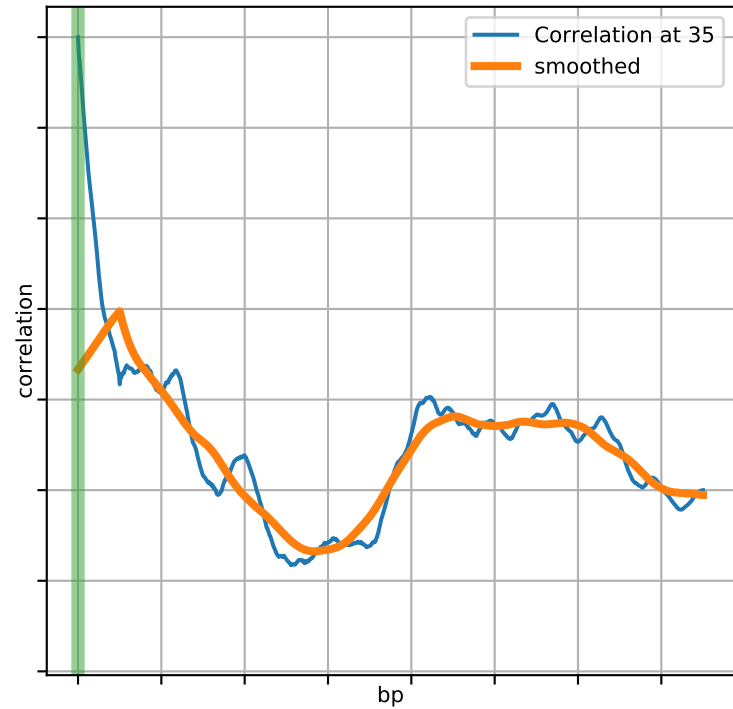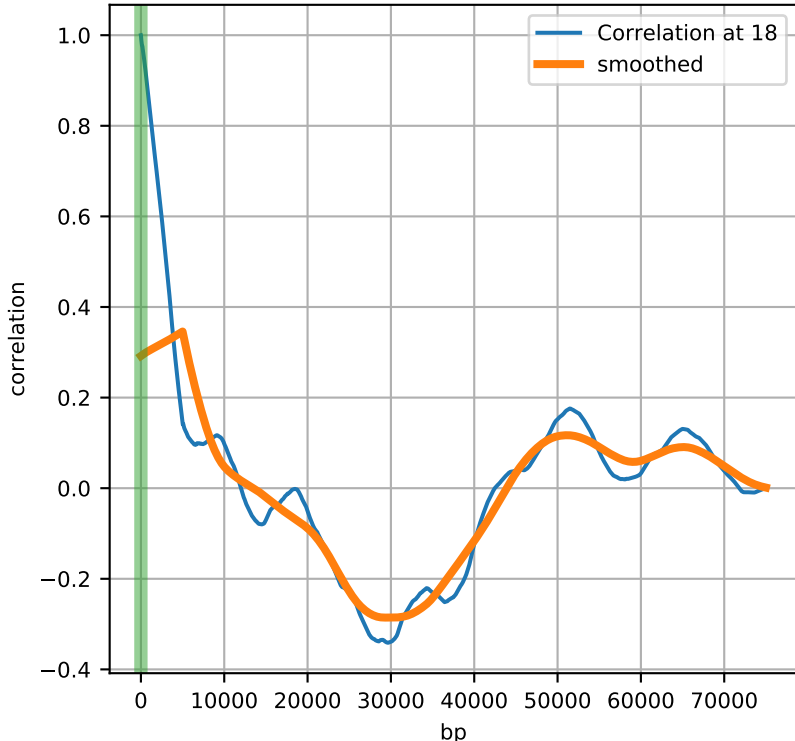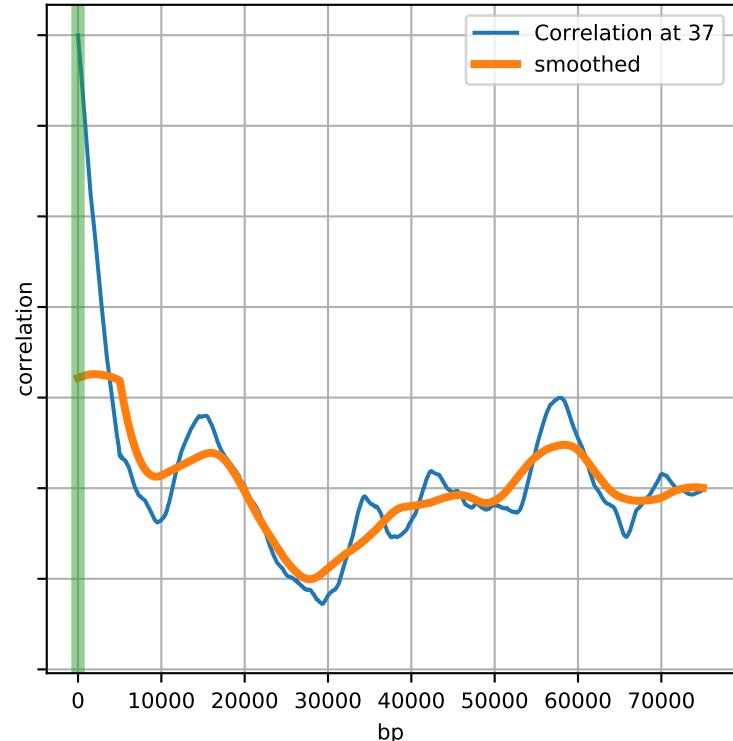

Supplement: Supplementary file 1 [file life-12-00541-s001.zip › life-1592845-supplementary/Heermann-cluster-correlation-function-2ca02c-chr2.pdf]

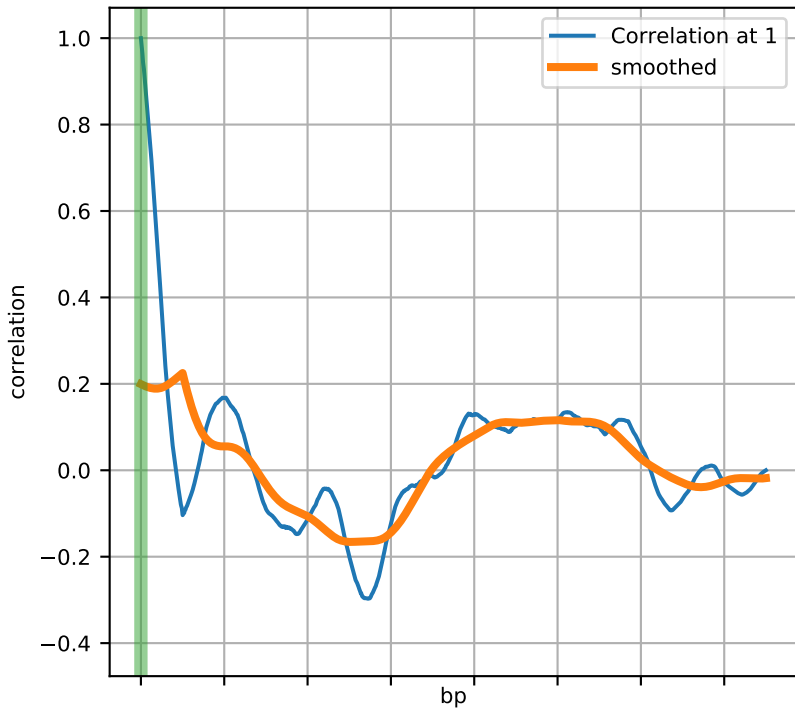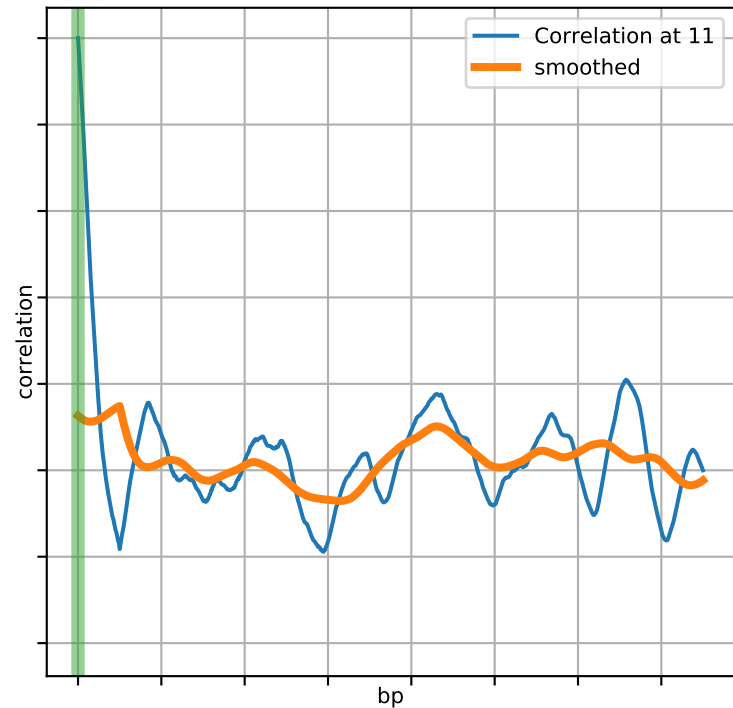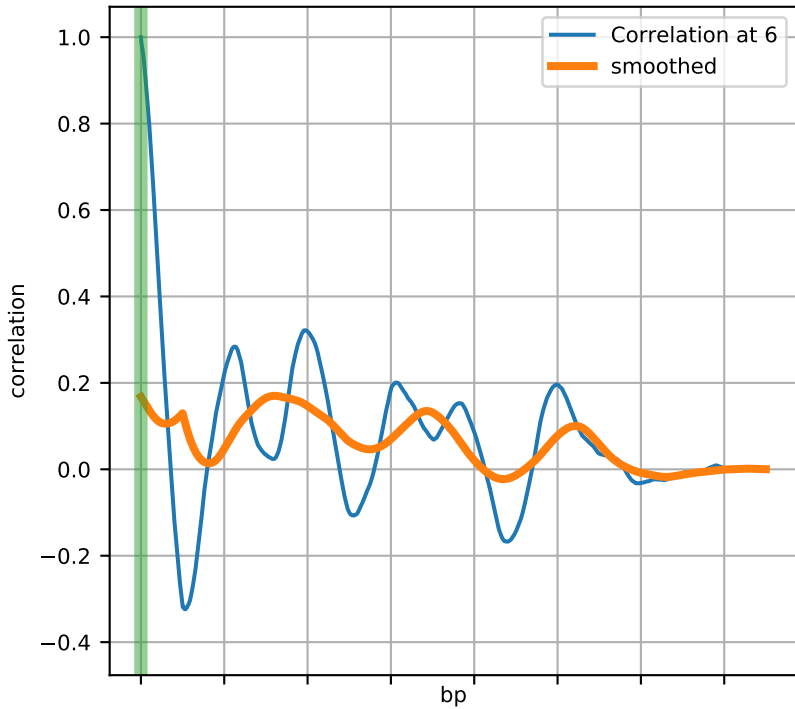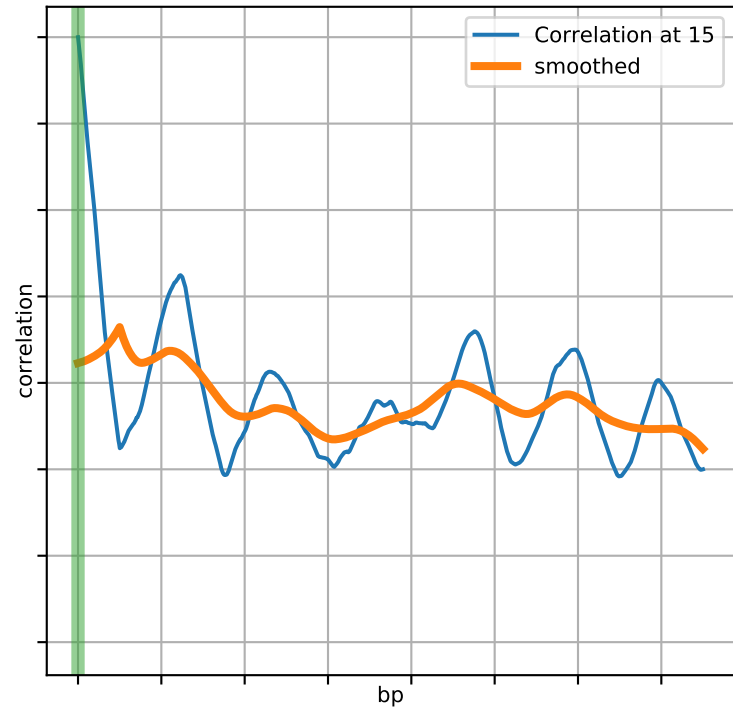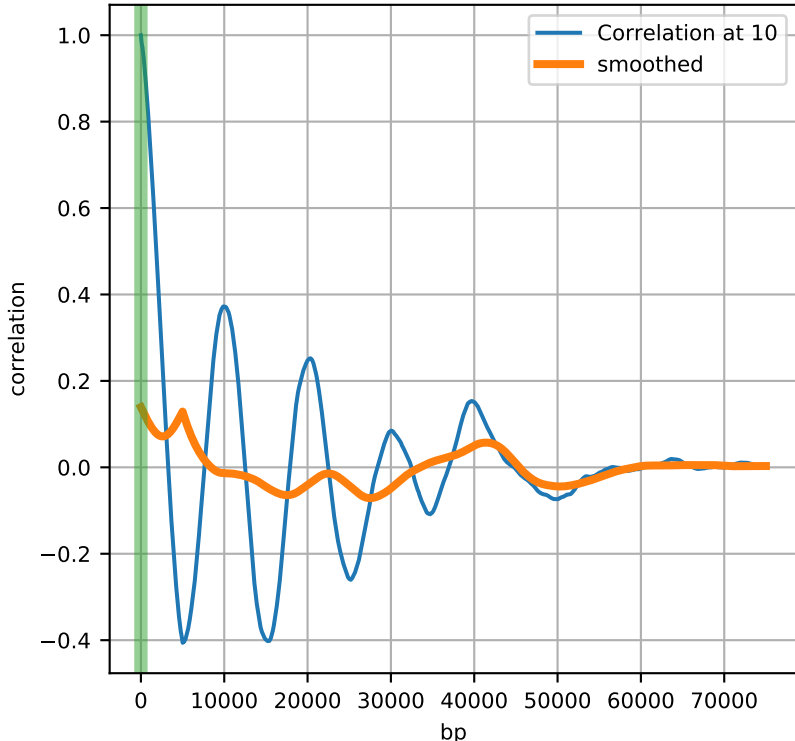

Supplement: Supplementary file 1 [file life-12-00541-s001.zip › life-1592845-supplementary/Heermann-cluster-correlation-function-2ca02c-chr5.pdf]

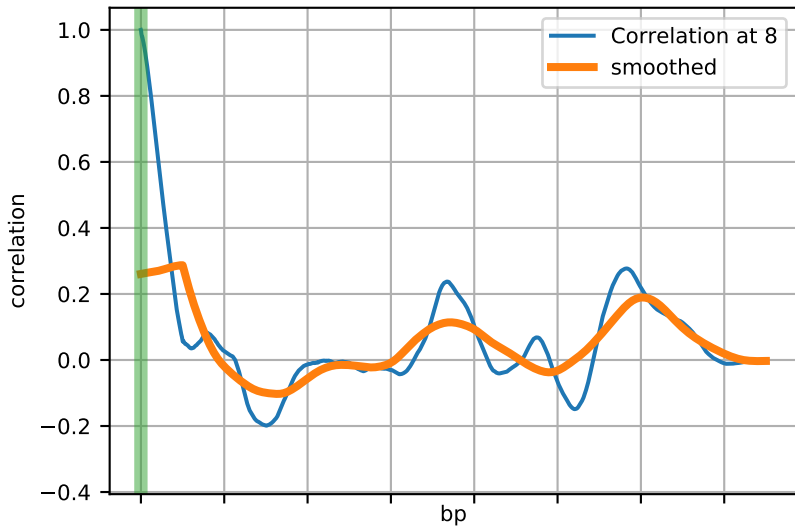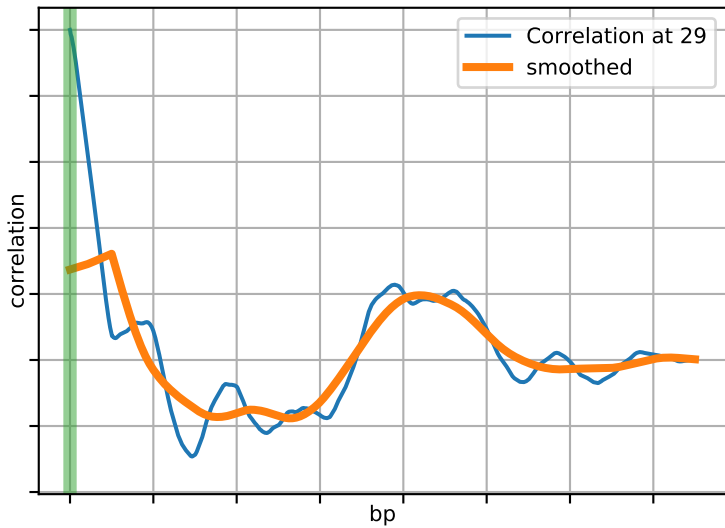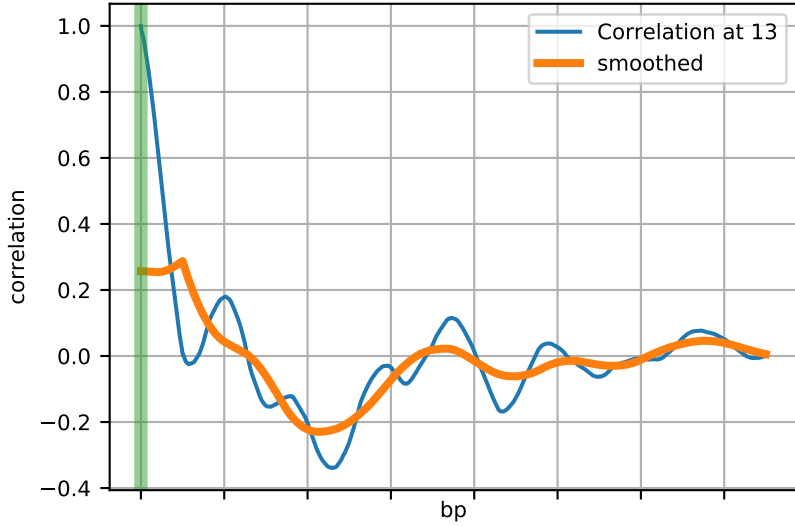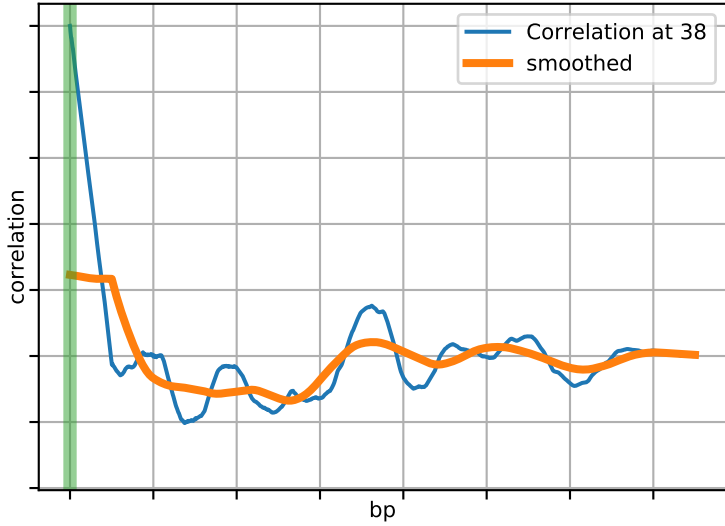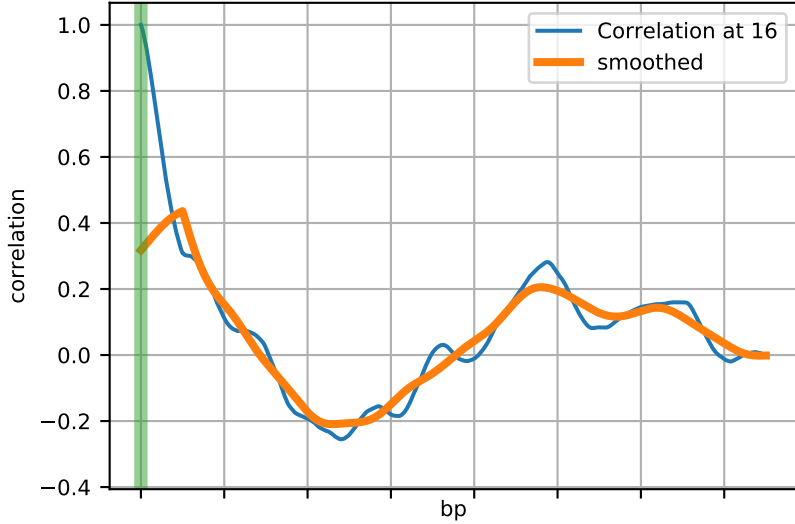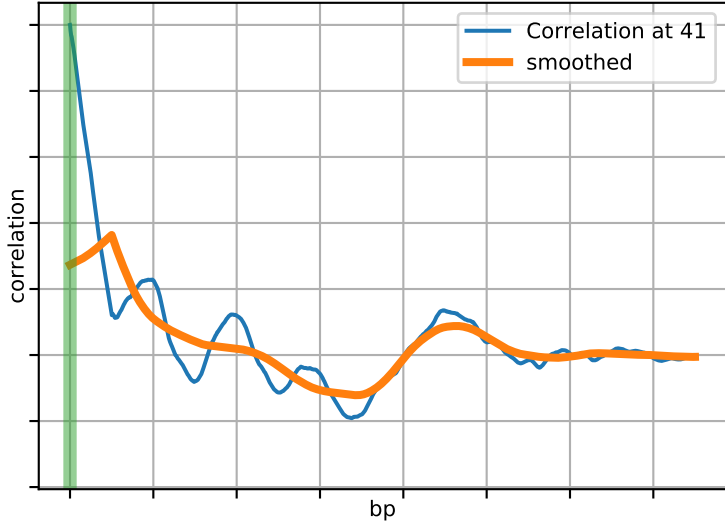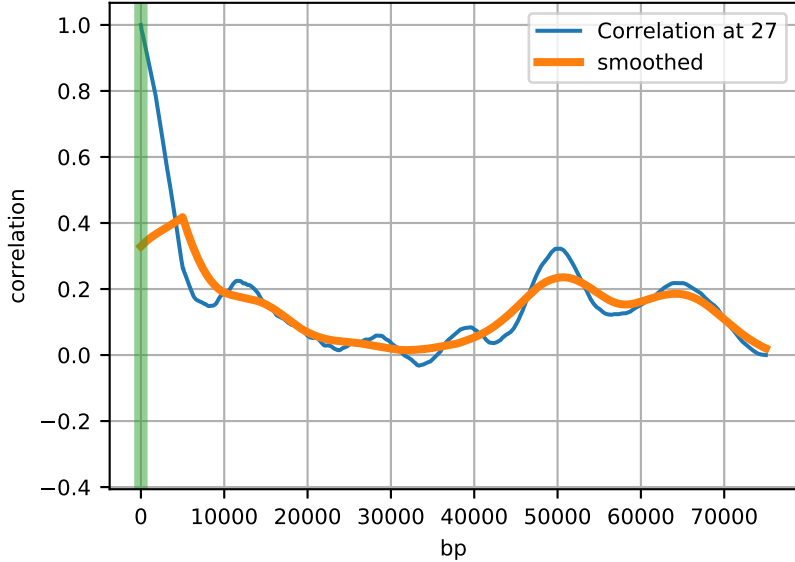

Supplement: Supplementary file 1 [file life-12-00541-s001.zip › life-1592845-supplementary/Heermann-cluster-correlation-function-2ca02c-chrR.pdf]

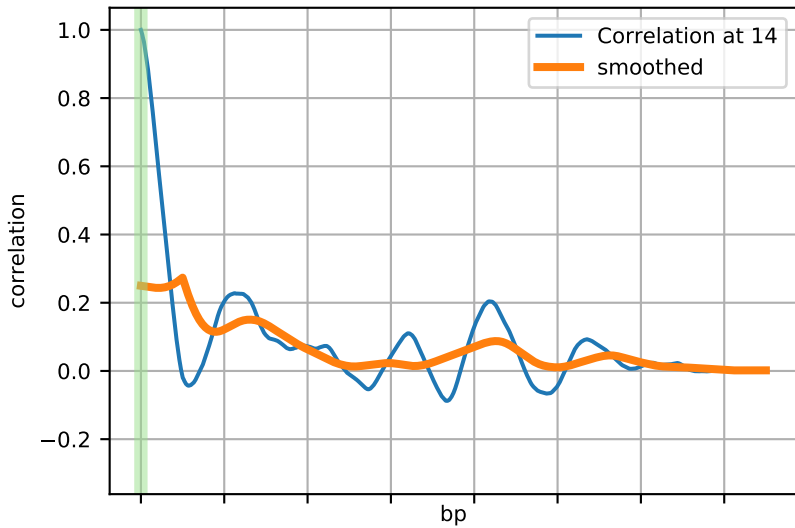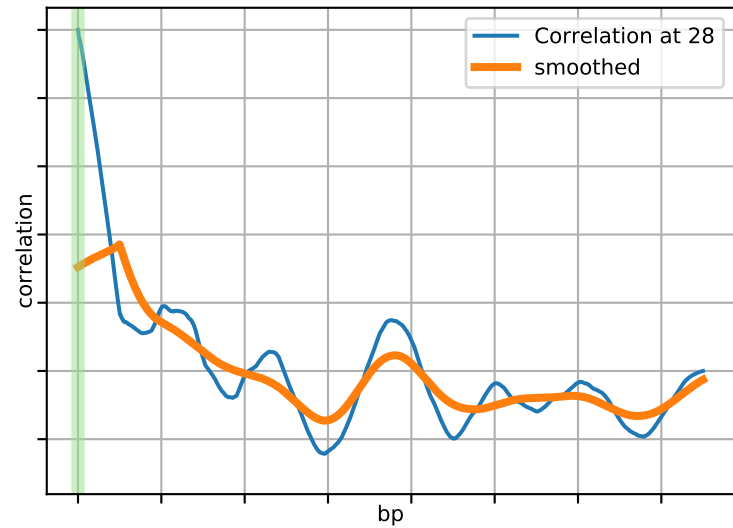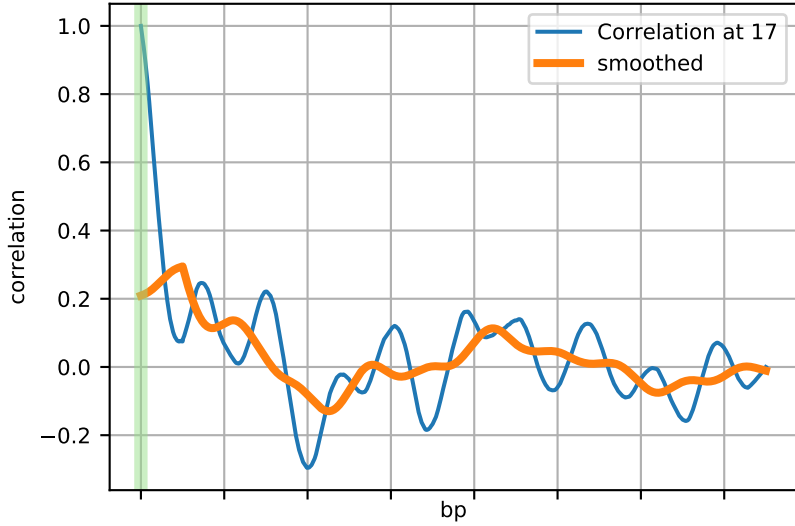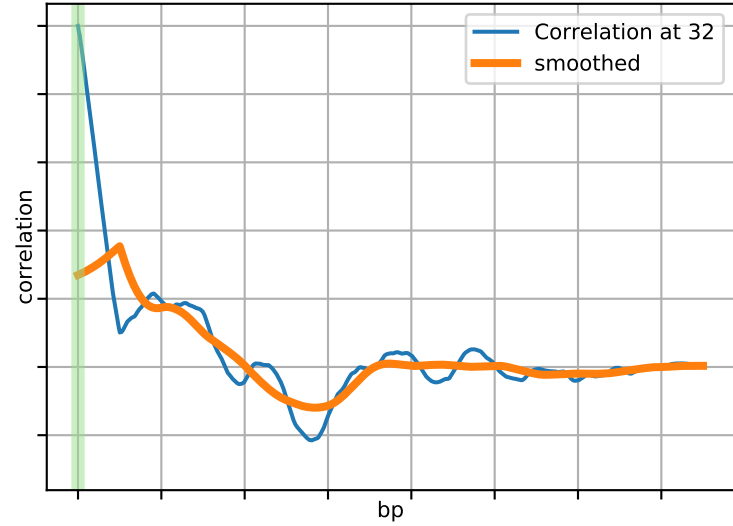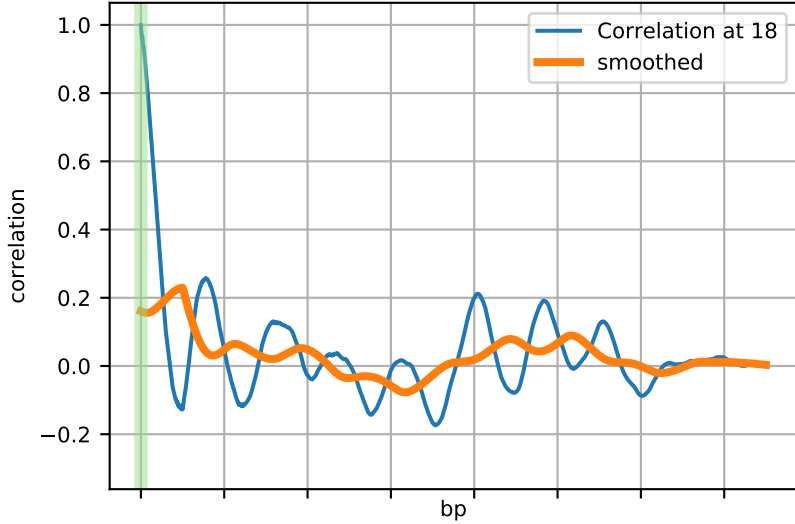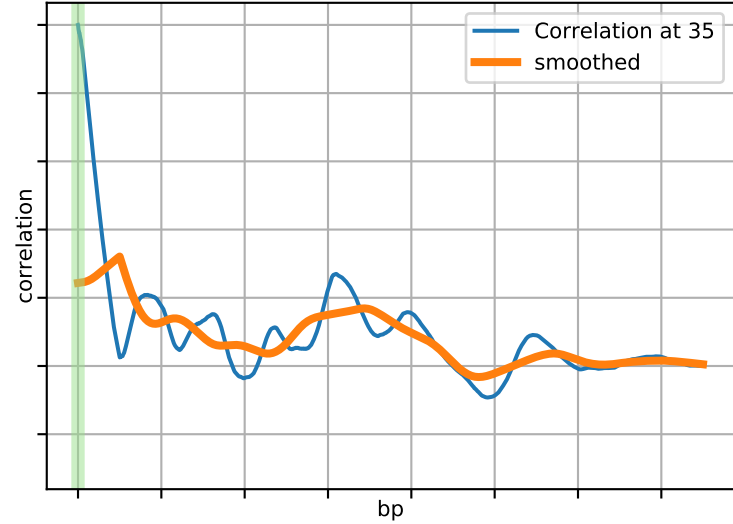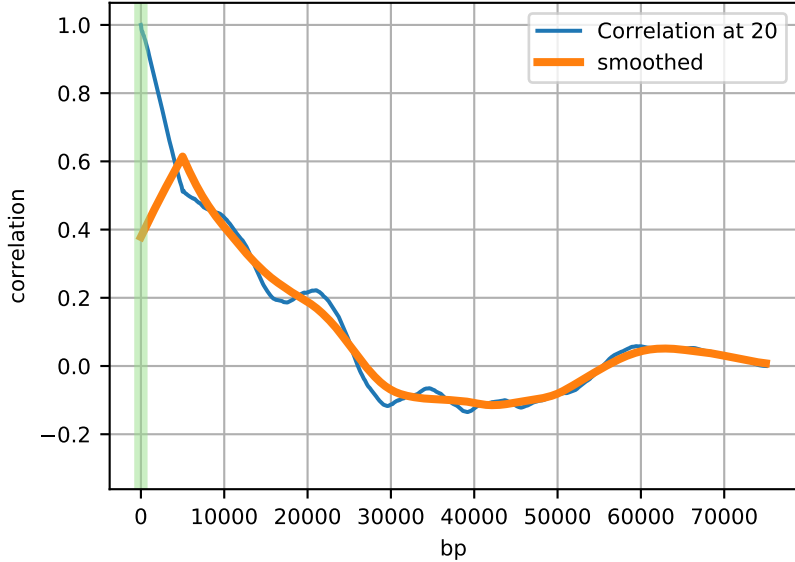

Supplement: Supplementary file 1 [file life-12-00541-s001.zip › life-1592845-supplementary/Heermann-cluster-correlation-function-98df8a-chrR.pdf]

Correlation and Cluster Analysis for Chromosomes chr1-chr2-chr3-chr4-chr5-chr6-chr7-chrR

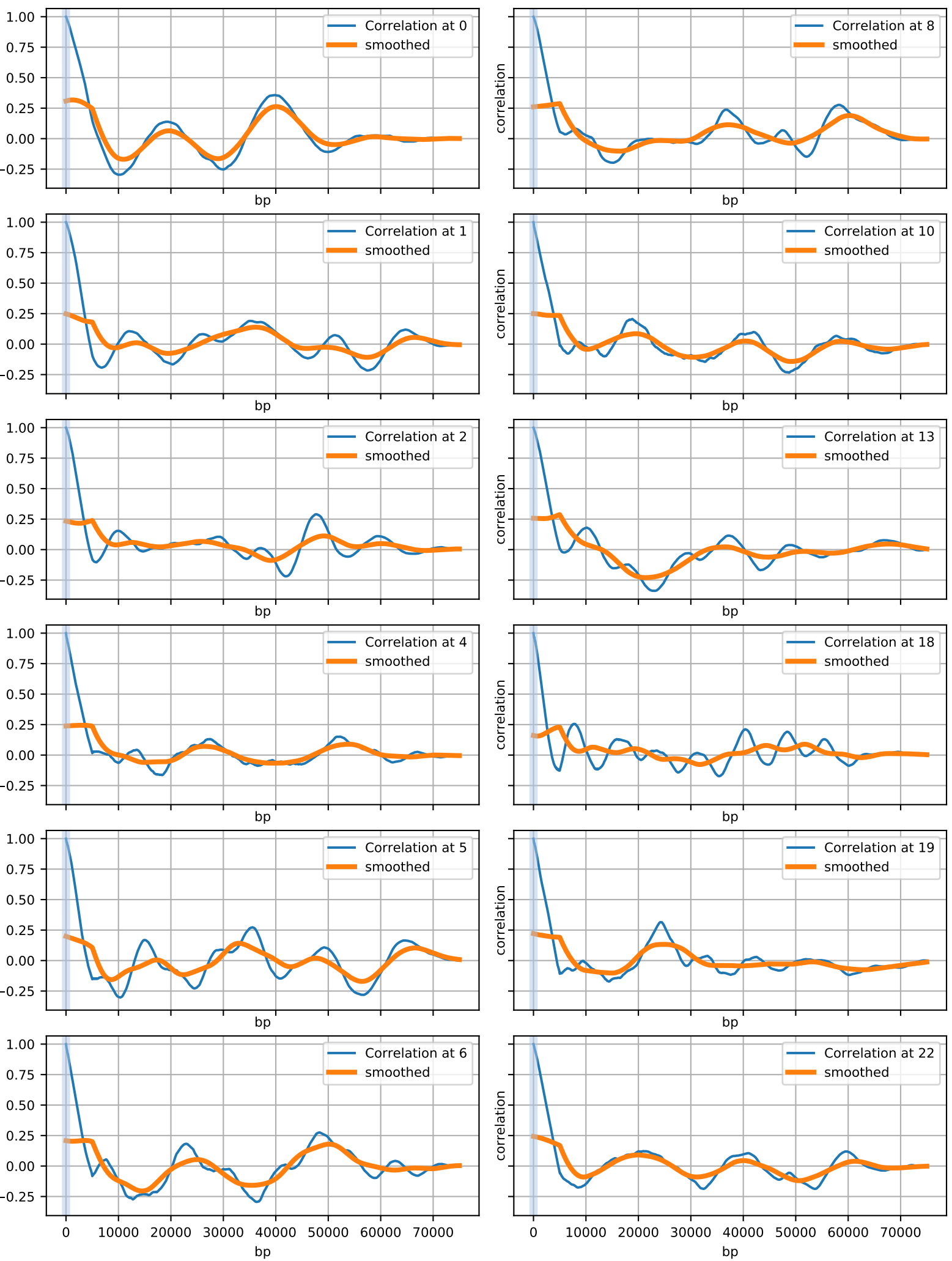

Supplement: Supplementary file 1 [file life-12-00541-s001.zip › life-1592845-supplementary/Heermann-cluster-correlation-function-aec7e8-chr1-chr2-chr3-chr4-chr5-chr6-chr7-chrR-1.pdf]

Correlation and Cluster Analysis for Chromosomes chr1-chr2-chr3-chr4-chr5-chr6-chr7-chrR

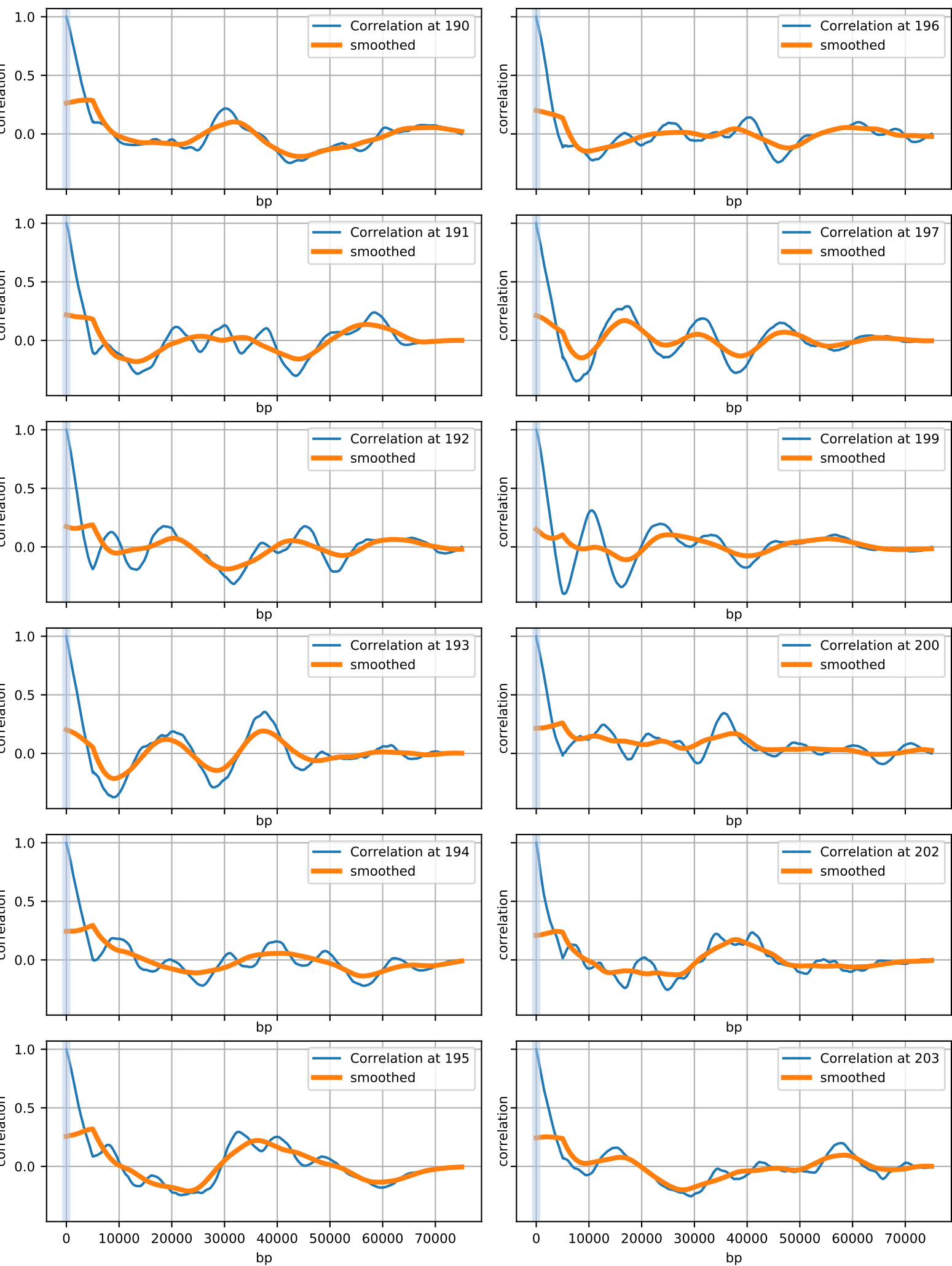

Supplement: Supplementary file 1 [file life-12-00541-s001.zip › life-1592845-supplementary/Heermann-cluster-correlation-function-aec7e8-chr1-chr2-chr3-chr4-chr5-chr6-chr7-chrR-10.pdf]

Correlation and Cluster Analysis for Chromosomes chr1-chr2-chr3-chr4-chr5-chr6-chr7-chrR

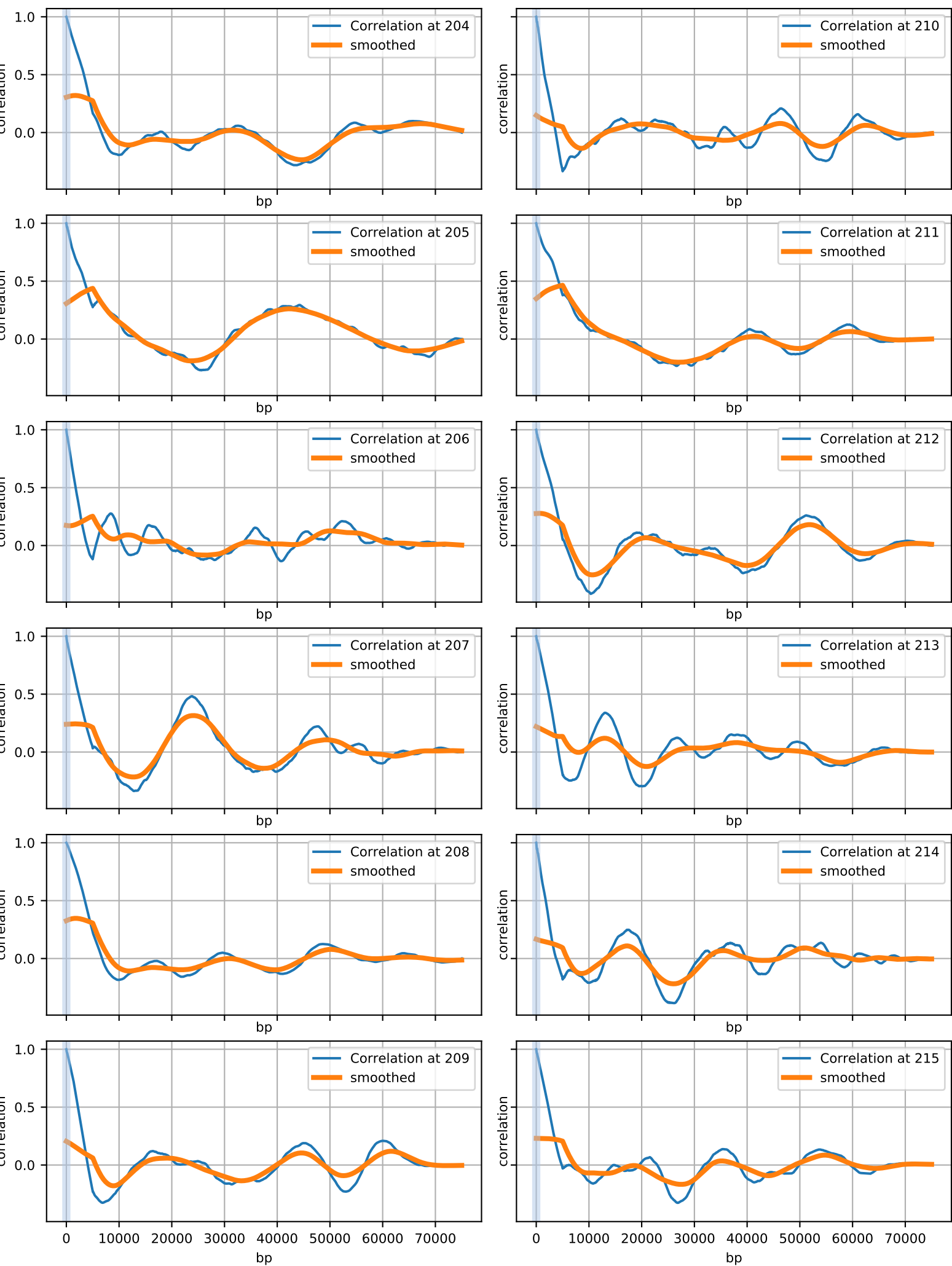

Supplement: Supplementary file 1 [file life-12-00541-s001.zip › life-1592845-supplementary/Heermann-cluster-correlation-function-aec7e8-chr1-chr2-chr3-chr4-chr5-chr6-chr7-chrR-11.pdf]

Correlation and Cluster Analysis for Chromosomes chr1-chr2-chr3-chr4-chr5-chr6-chr7-chrR

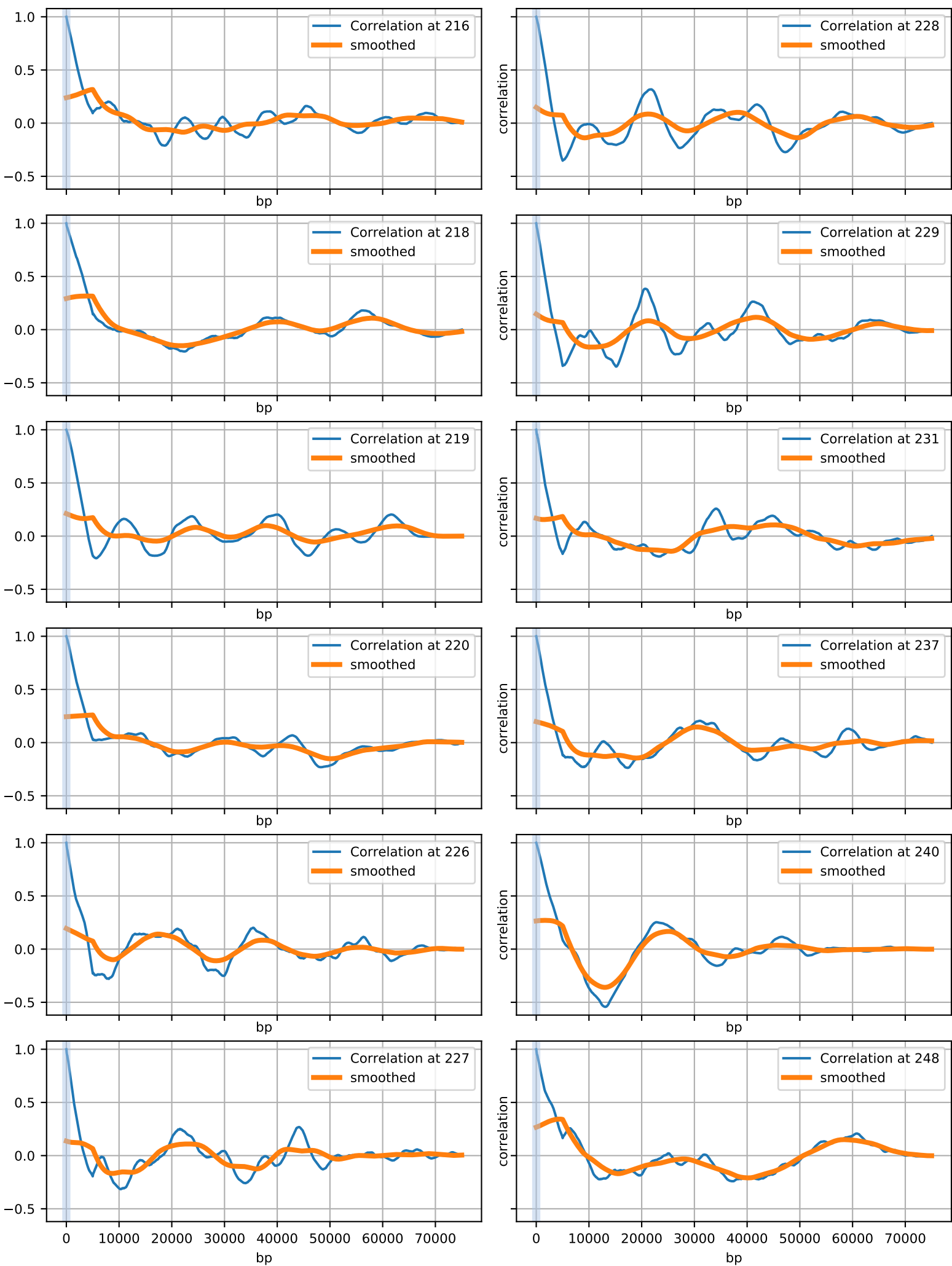

Supplement: Supplementary file 1 [file life-12-00541-s001.zip › life-1592845-supplementary/Heermann-cluster-correlation-function-aec7e8-chr1-chr2-chr3-chr4-chr5-chr6-chr7-chrR-12.pdf]

Correlation and Cluster Analysis for Chromosomes chr1-chr2-chr3-chr4-chr5-chr6-chr7-chrR

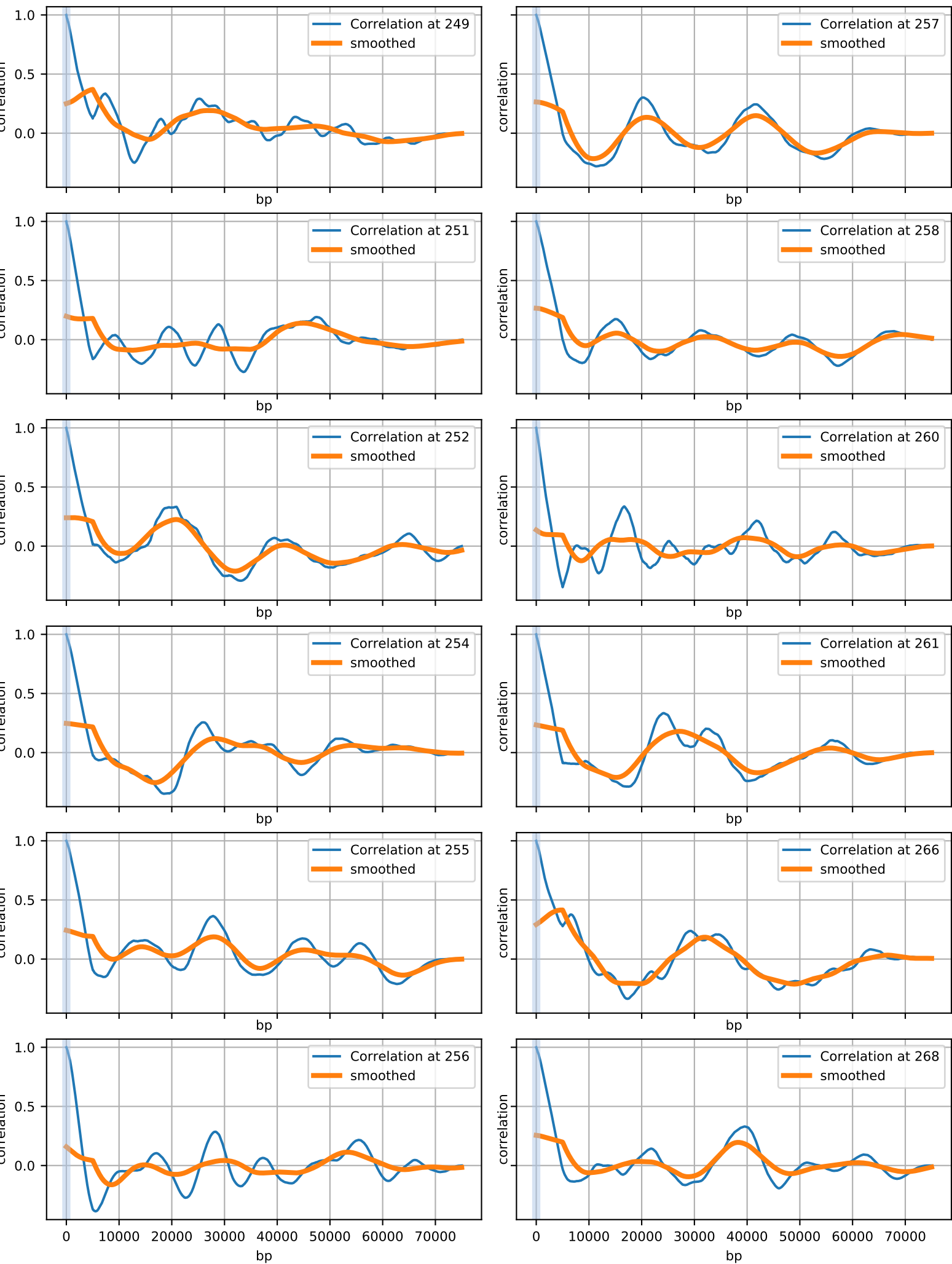

Supplement: Supplementary file 1 [file life-12-00541-s001.zip › life-1592845-supplementary/Heermann-cluster-correlation-function-aec7e8-chr1-chr2-chr3-chr4-chr5-chr6-chr7-chrR-13.pdf]

Correlation and Cluster Analysis for Chromosomes chr1-chr2-chr3-chr4-chr5-chr6-chr7-chrR

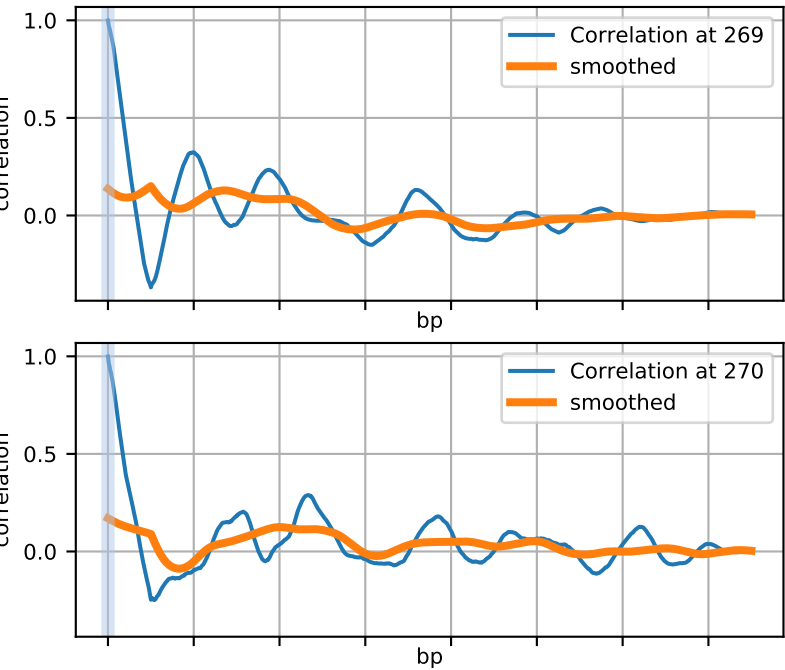

Supplement: Supplementary file 1 [file life-12-00541-s001.zip › life-1592845-supplementary/Heermann-cluster-correlation-function-aec7e8-chr1-chr2-chr3-chr4-chr5-chr6-chr7-chrR-14.pdf]

Correlation and Cluster Analysis for Chromosomes chr1-chr2-chr3-chr4-chr5-chr6-chr7-chrR

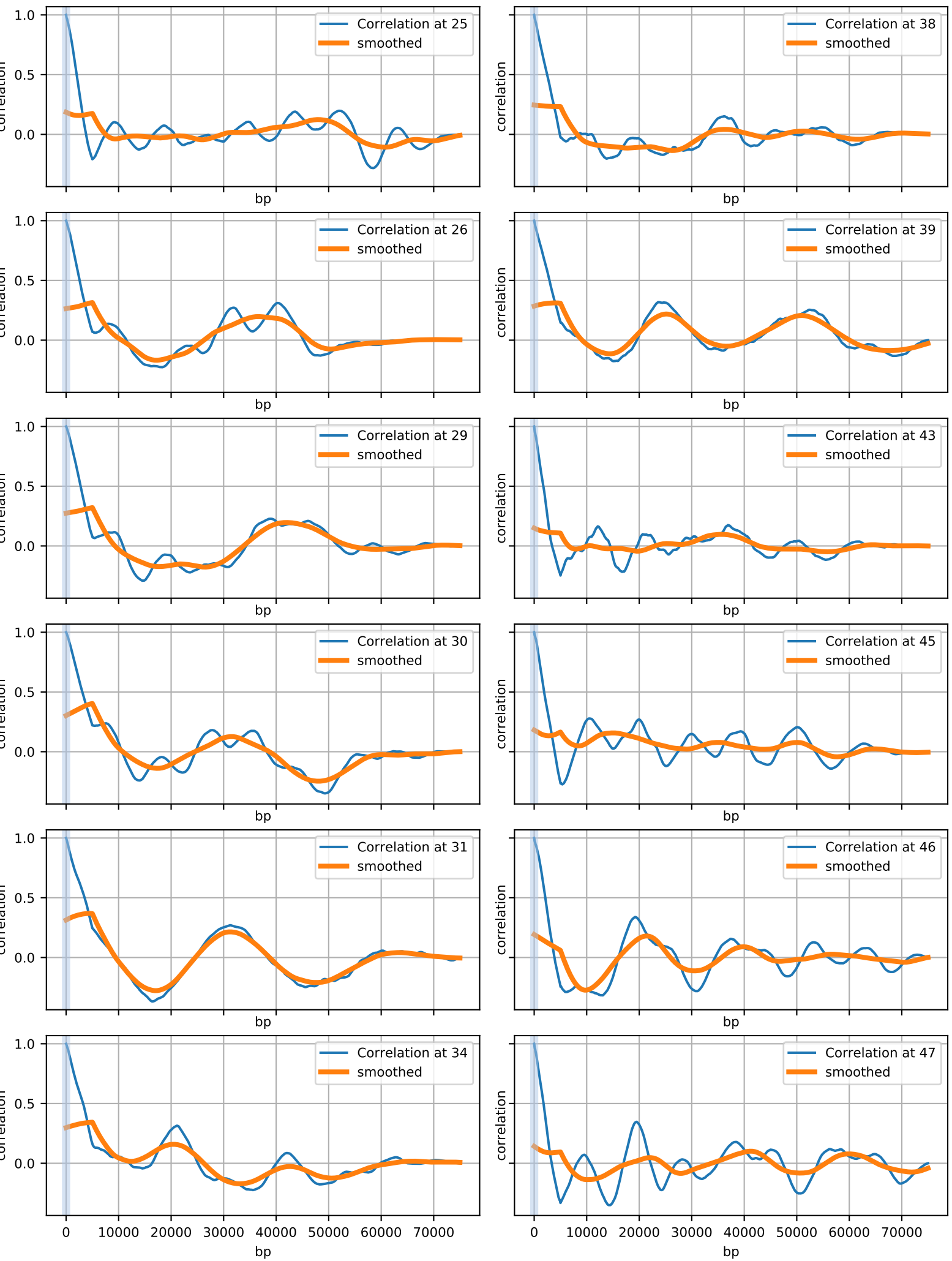

Supplement: Supplementary file 1 [file life-12-00541-s001.zip › life-1592845-supplementary/Heermann-cluster-correlation-function-aec7e8-chr1-chr2-chr3-chr4-chr5-chr6-chr7-chrR-2.pdf]

Correlation and Cluster Analysis for Chromosomes chr1-chr2-chr3-chr4-chr5-chr6-chr7-chrR

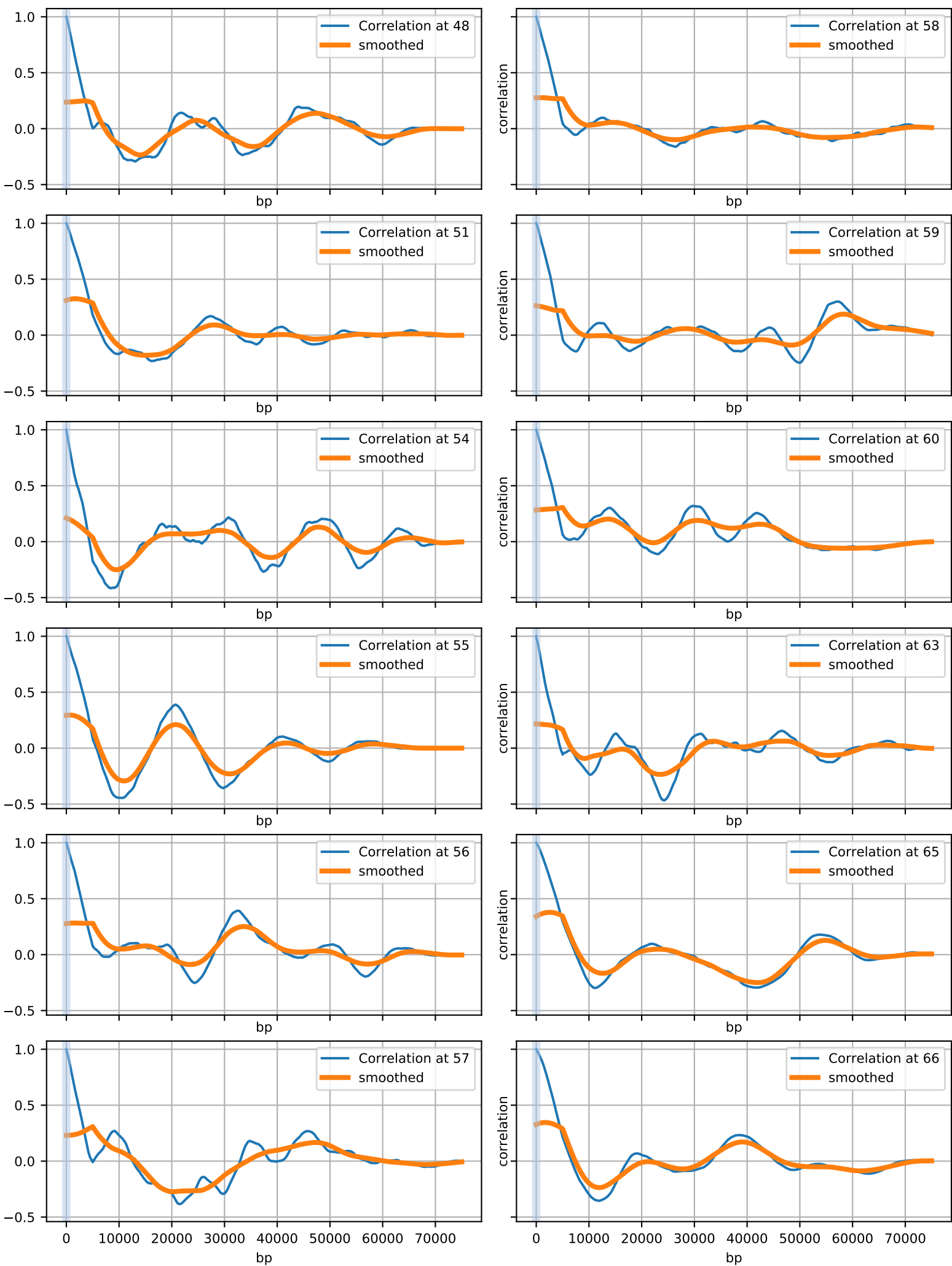

Supplement: Supplementary file 1 [file life-12-00541-s001.zip › life-1592845-supplementary/Heermann-cluster-correlation-function-aec7e8-chr1-chr2-chr3-chr4-chr5-chr6-chr7-chrR-3.pdf]

Correlation and Cluster Analysis for Chromosomes chr1-chr2-chr3-chr4-chr5-chr6-chr7-chrR

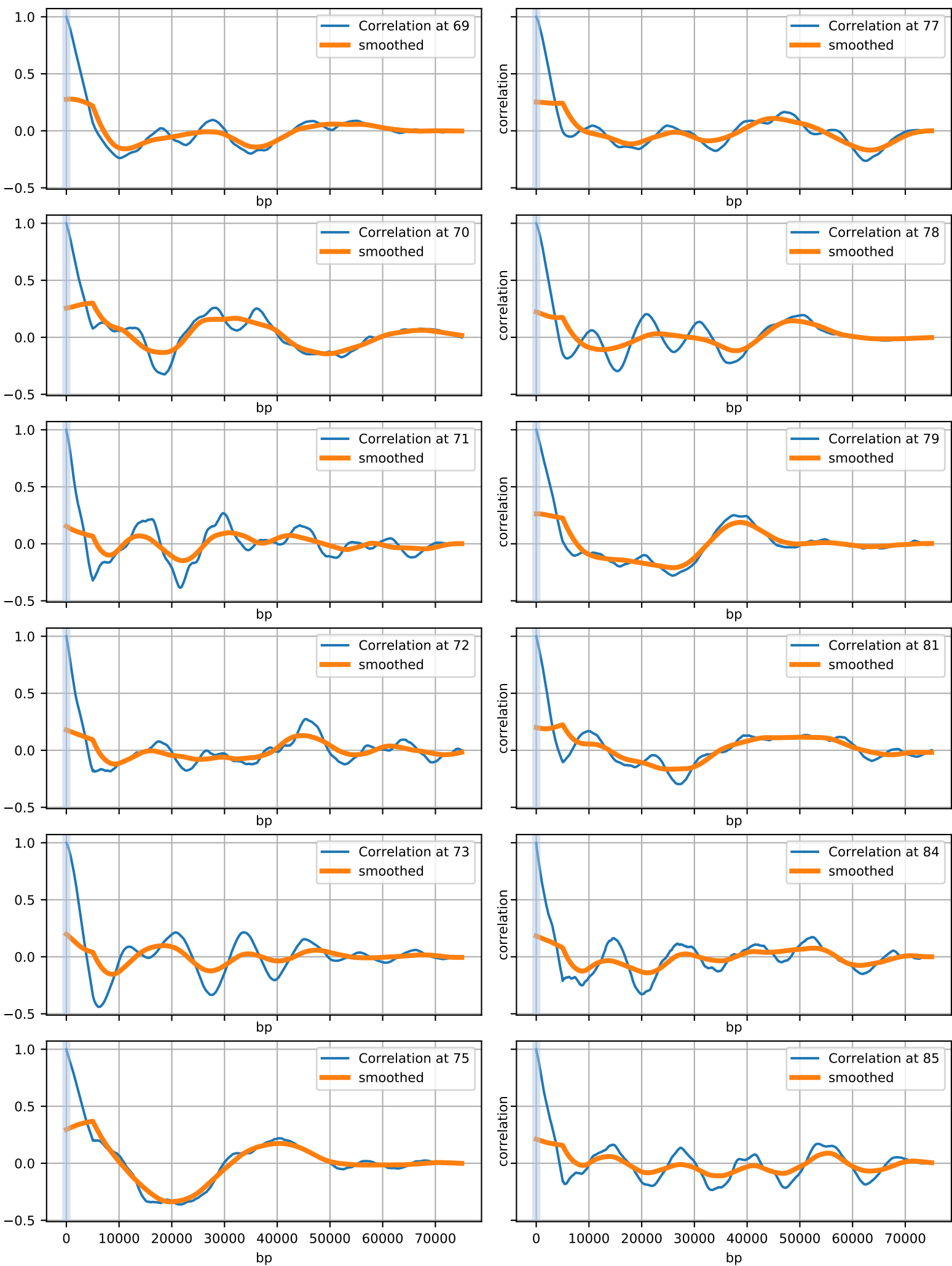

Supplement: Supplementary file 1 [file life-12-00541-s001.zip › life-1592845-supplementary/Heermann-cluster-correlation-function-aec7e8-chr1-chr2-chr3-chr4-chr5-chr6-chr7-chrR-4.pdf]

Correlation and Cluster Analysis for Chromosomes chr1-chr2-chr3-chr4-chr5-chr6-chr7-chrR

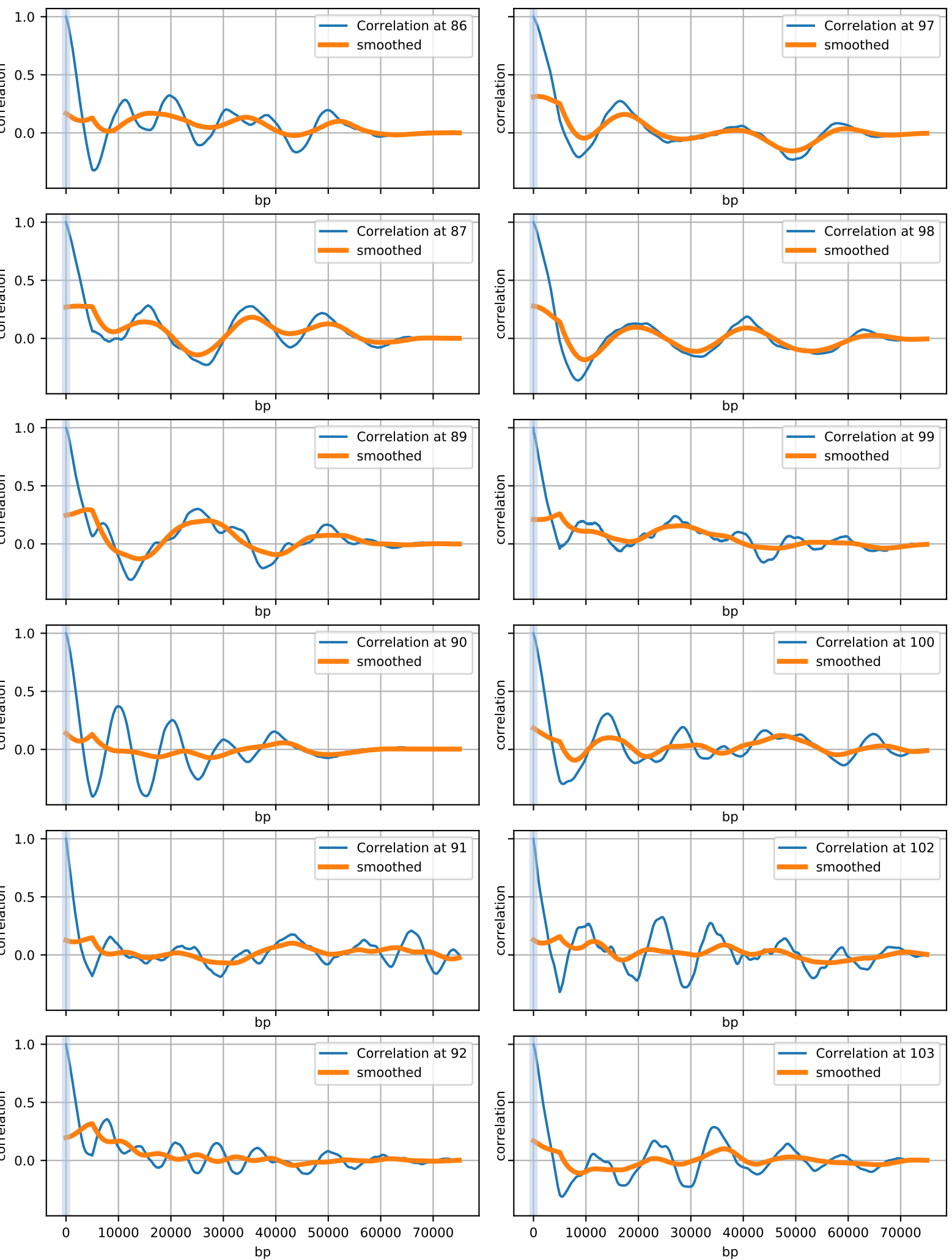

Supplement: Supplementary file 1 [file life-12-00541-s001.zip › life-1592845-supplementary/Heermann-cluster-correlation-function-aec7e8-chr1-chr2-chr3-chr4-chr5-chr6-chr7-chrR-5.pdf]

Correlation and Cluster Analysis for Chromosomes chr1-chr2-chr3-chr4-chr5-chr6-chr7-chrR

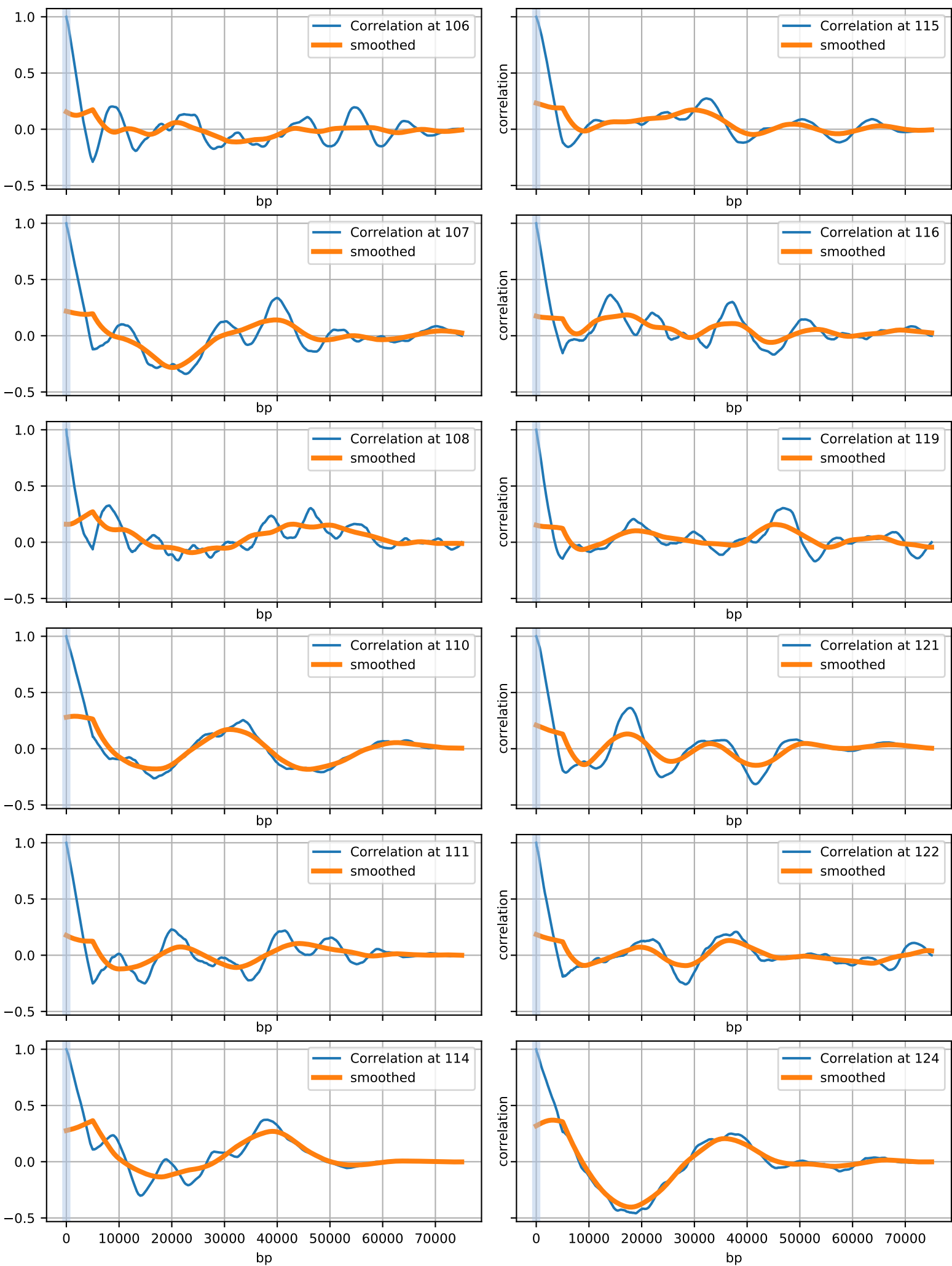

Supplement: Supplementary file 1 [file life-12-00541-s001.zip › life-1592845-supplementary/Heermann-cluster-correlation-function-aec7e8-chr1-chr2-chr3-chr4-chr5-chr6-chr7-chrR-6.pdf]

Correlation and Cluster Analysis for Chromosomes chr1-chr2-chr3-chr4-chr5-chr6-chr7-chrR

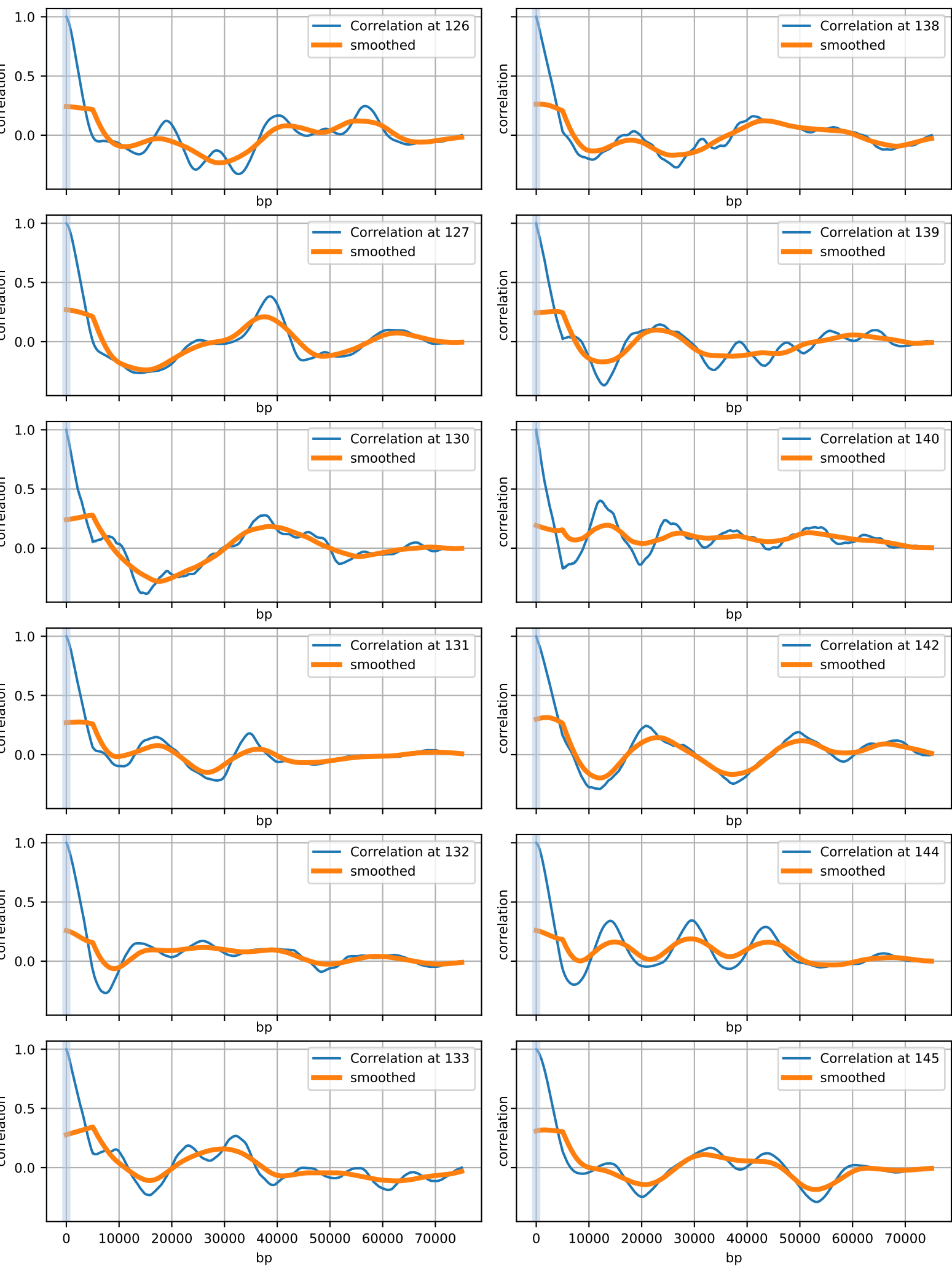

Supplement: Supplementary file 1 [file life-12-00541-s001.zip › life-1592845-supplementary/Heermann-cluster-correlation-function-aec7e8-chr1-chr2-chr3-chr4-chr5-chr6-chr7-chrR-7.pdf]

Correlation and Cluster Analysis for Chromosomes chr1-chr2-chr3-chr4-chr5-chr6-chr7-chrR

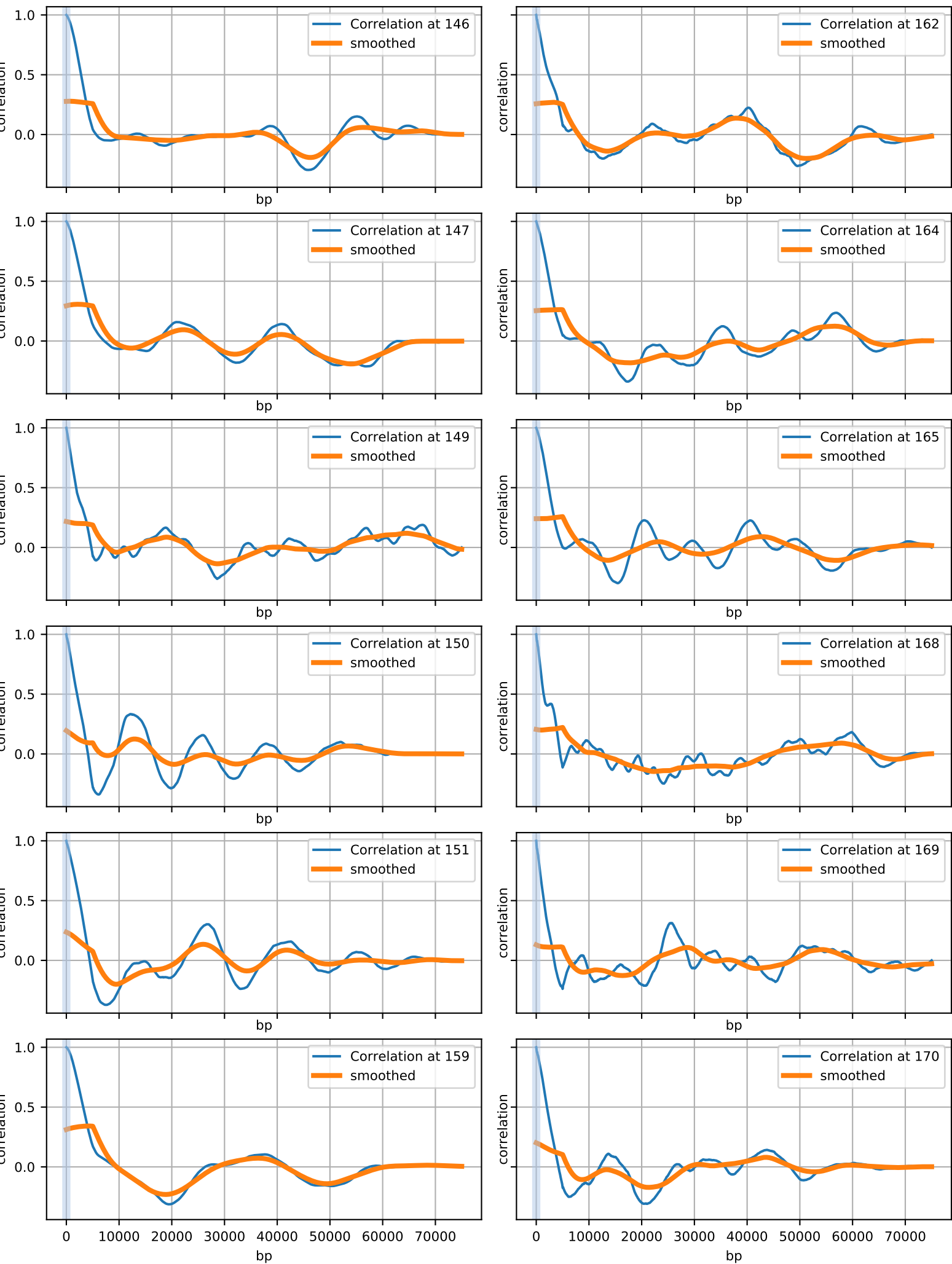

Supplement: Supplementary file 1 [file life-12-00541-s001.zip › life-1592845-supplementary/Heermann-cluster-correlation-function-aec7e8-chr1-chr2-chr3-chr4-chr5-chr6-chr7-chrR-8.pdf]

Correlation and Cluster Analysis for Chromosomes chr1-chr2-chr3-chr4-chr5-chr6-chr7-chrR

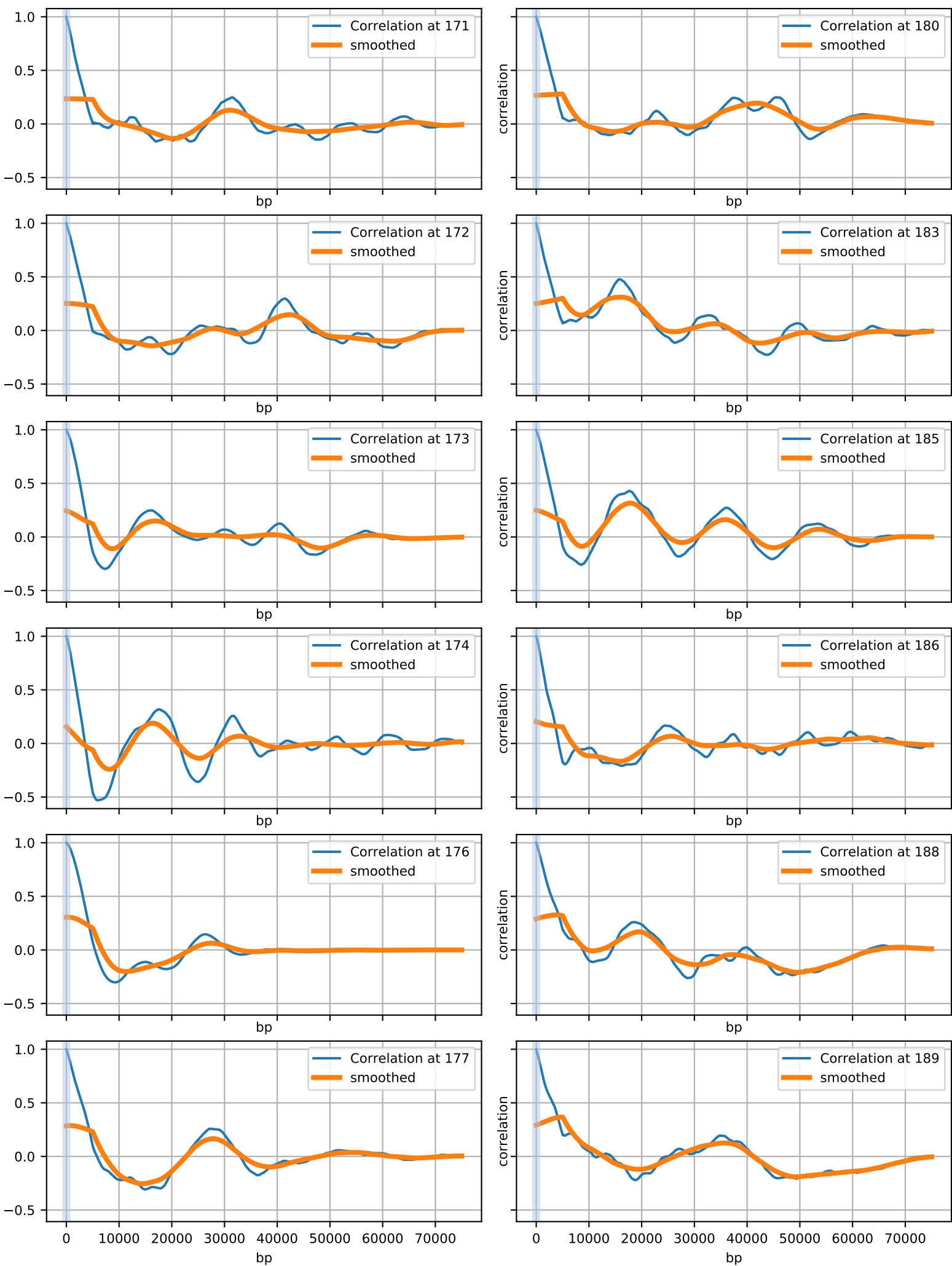

Supplement: Supplementary file 1 [file life-12-00541-s001.zip › life-1592845-supplementary/Heermann-cluster-correlation-function-aec7e8-chr1-chr2-chr3-chr4-chr5-chr6-chr7-chrR-9.pdf]

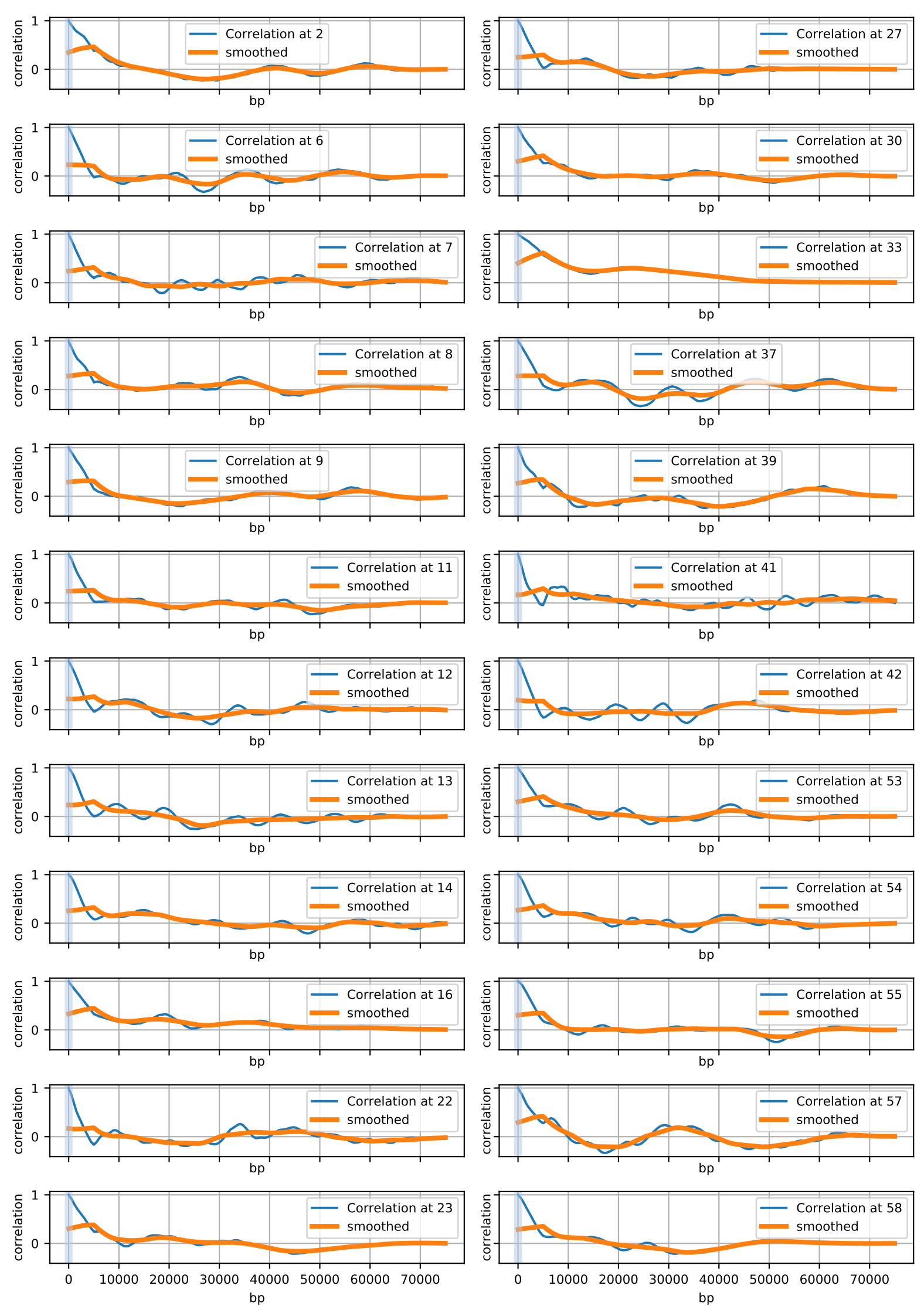

Supplement: Supplementary file 1 [file life-12-00541-s001.zip › life-1592845-supplementary/Heermann-cluster-correlation-function-aec7e8-chr1.pdf]

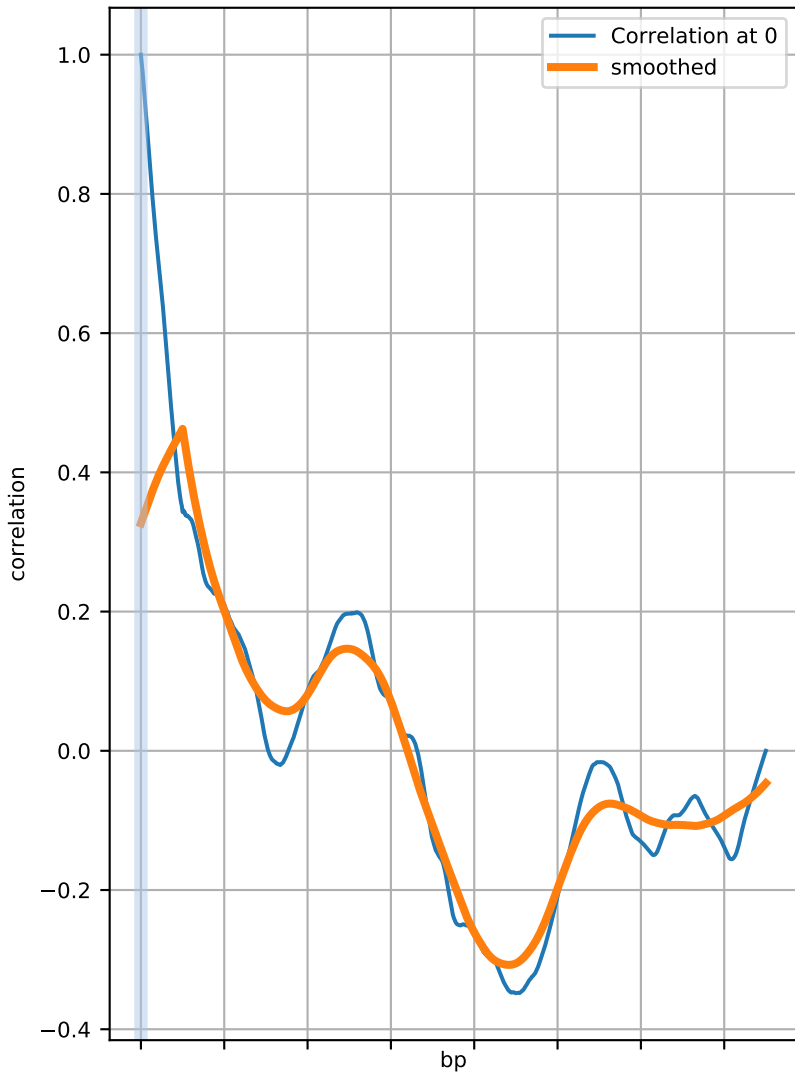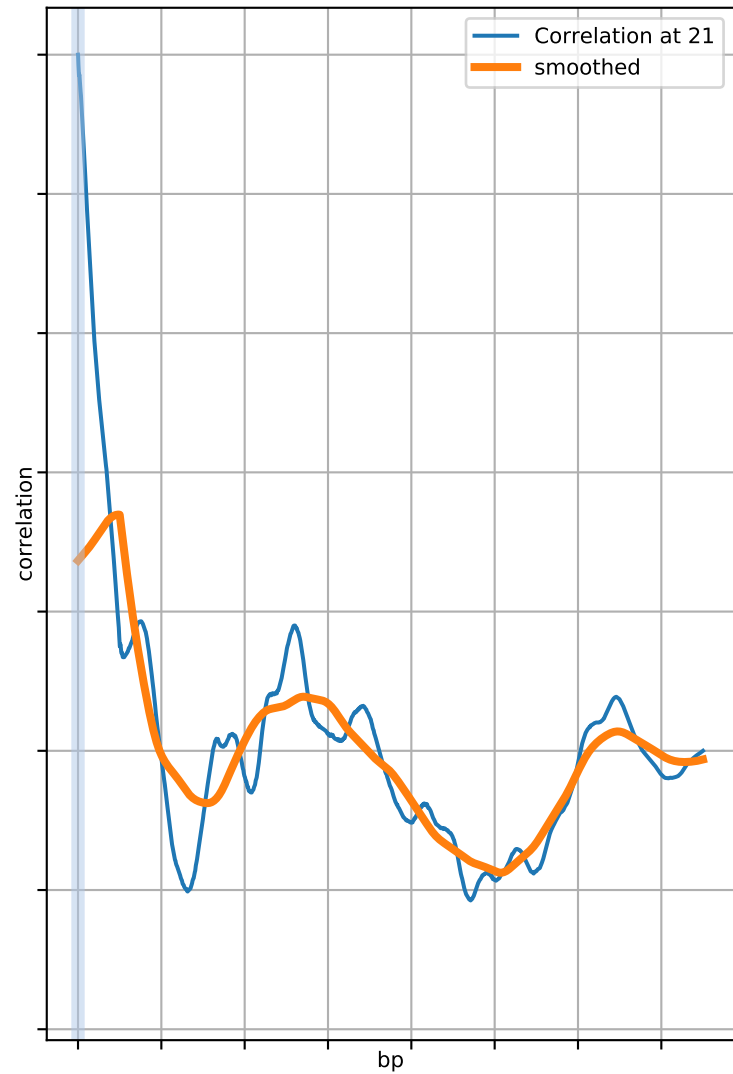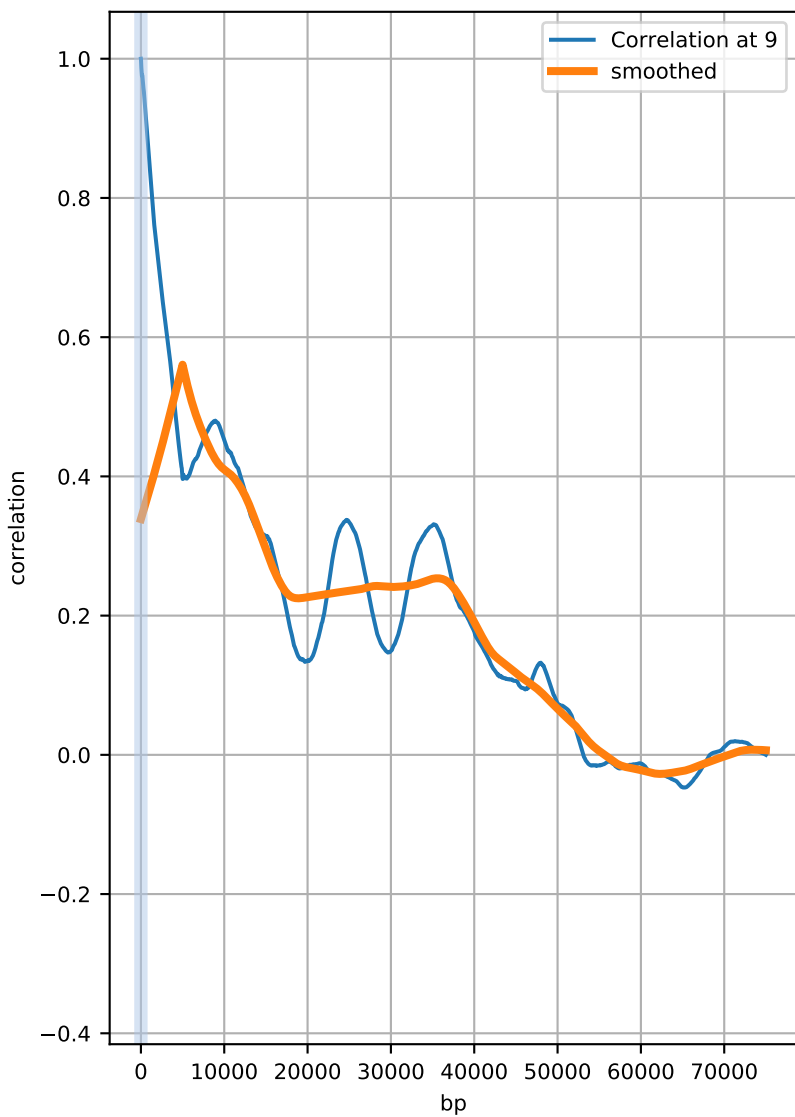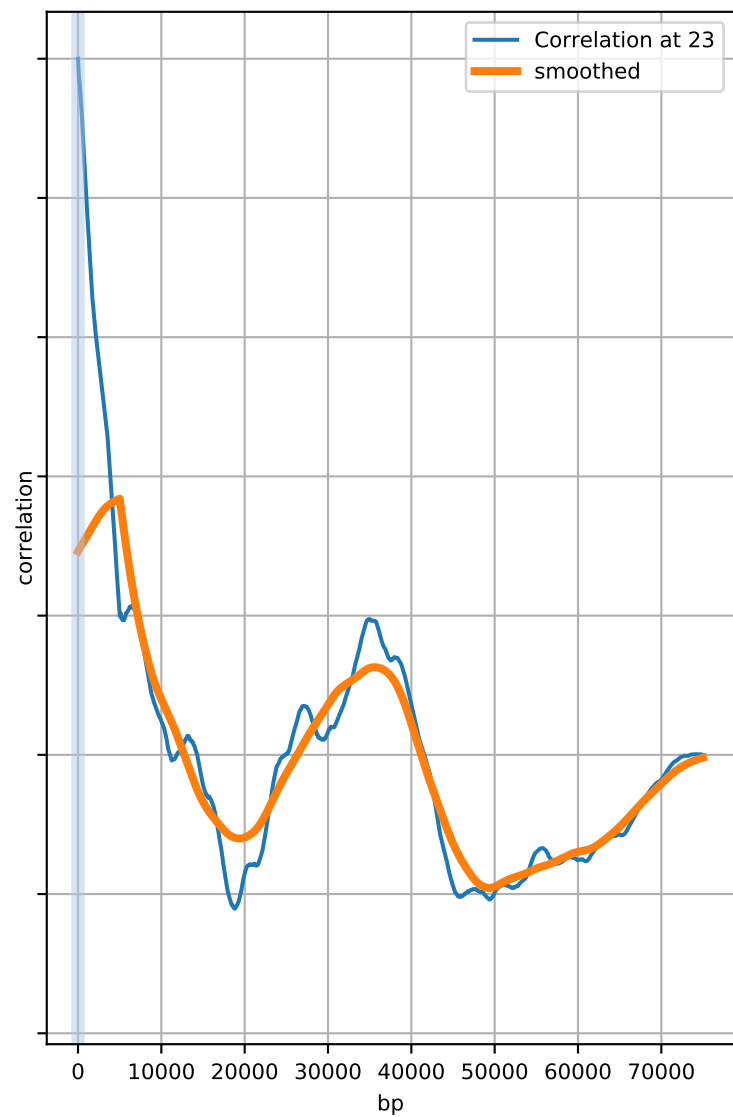

Supplement: Supplementary file 1 [file life-12-00541-s001.zip › life-1592845-supplementary/Heermann-cluster-correlation-function-aec7e8-chr2.pdf]

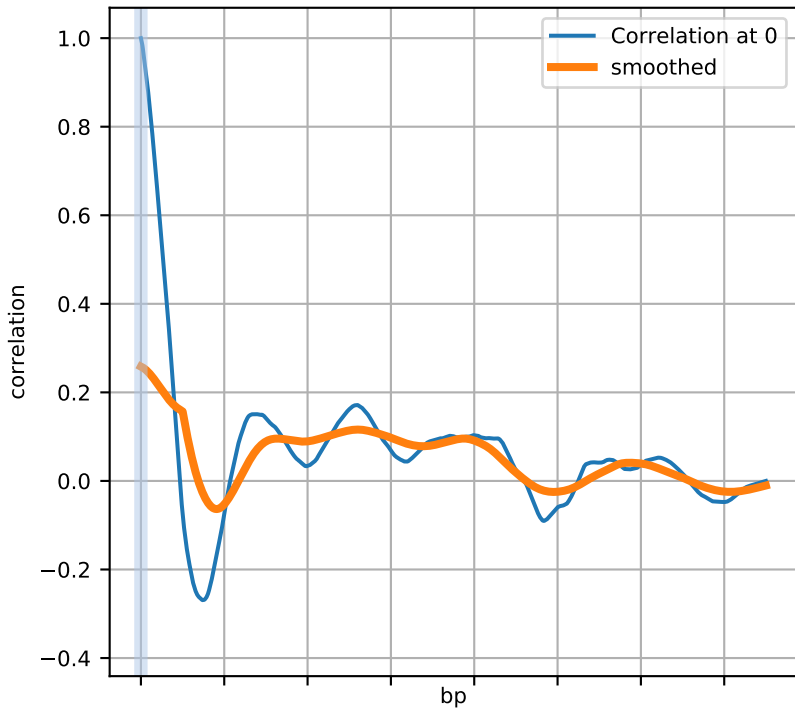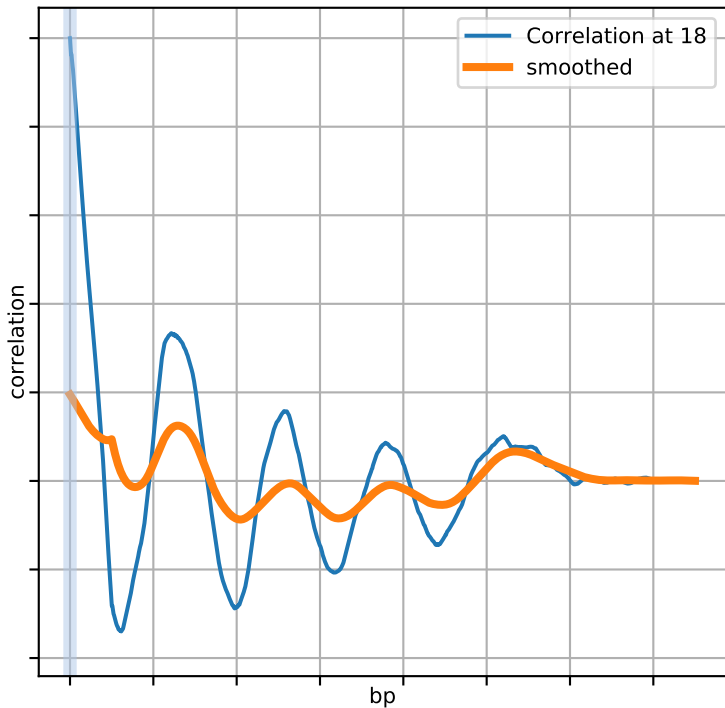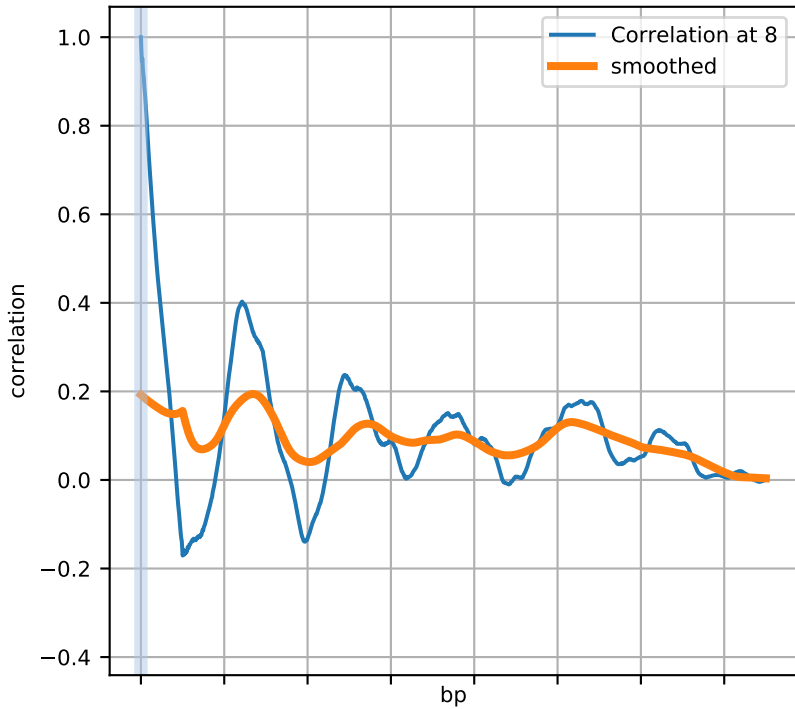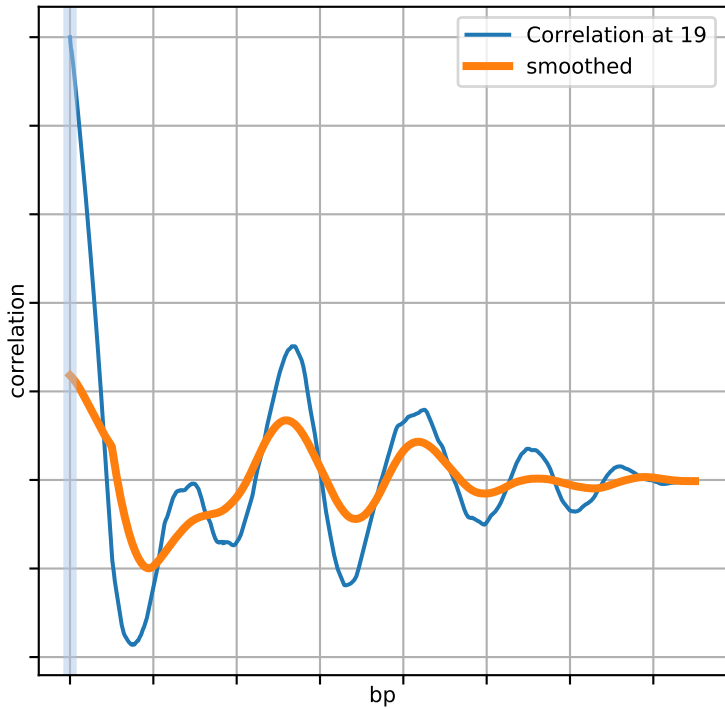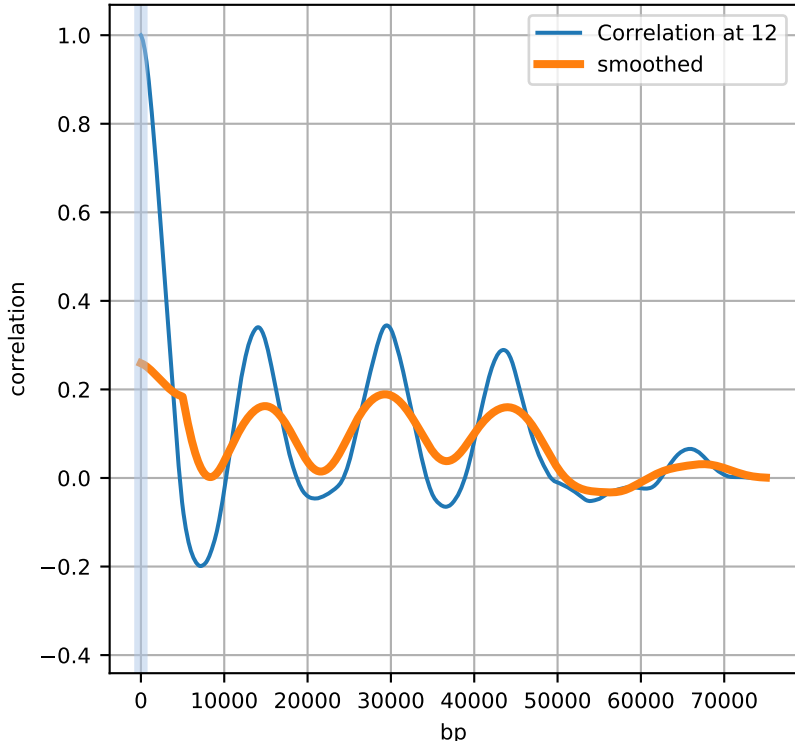

Supplement: Supplementary file 1 [file life-12-00541-s001.zip › life-1592845-supplementary/Heermann-cluster-correlation-function-aec7e8-chr3.pdf]

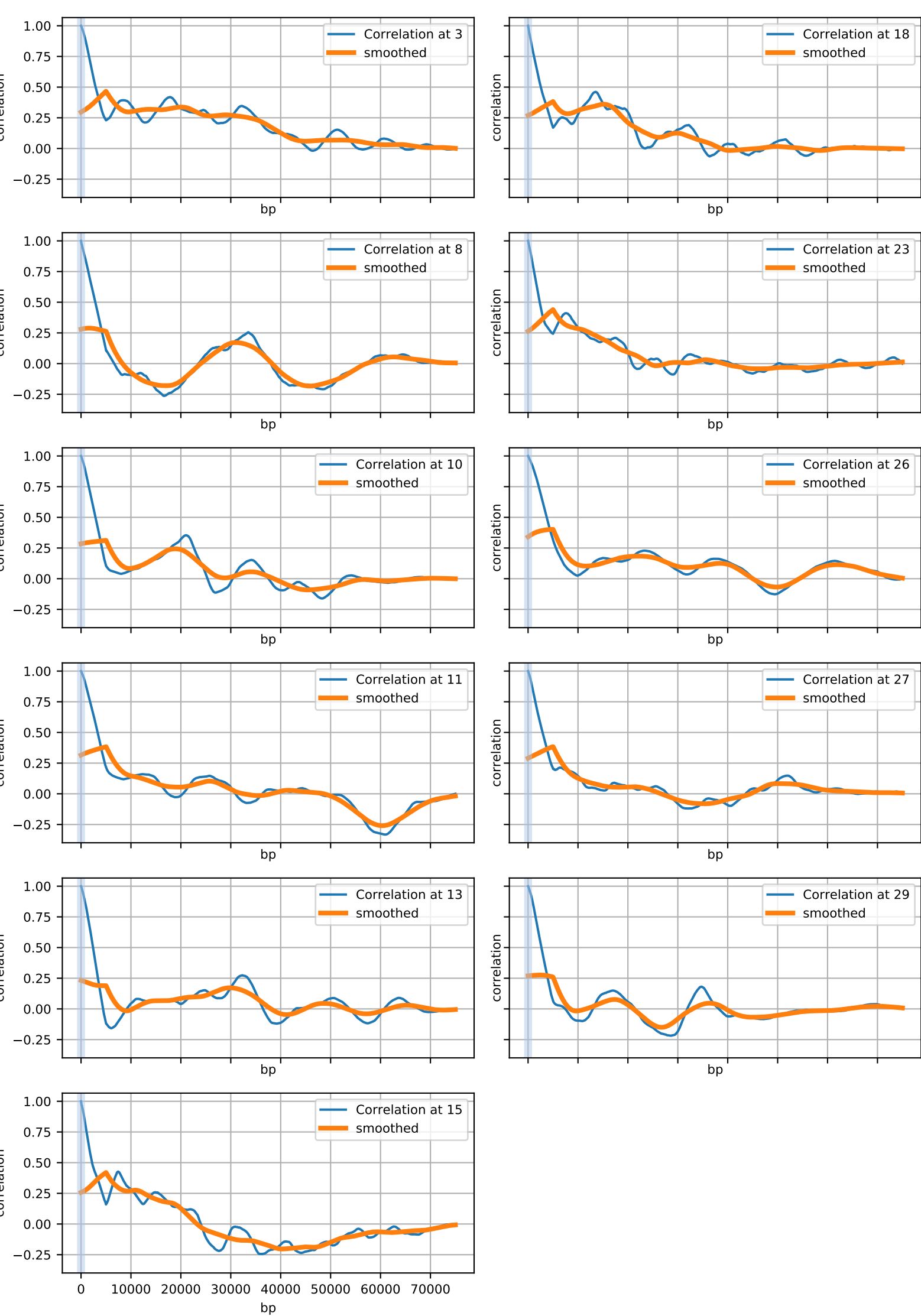

Supplement: Supplementary file 1 [file life-12-00541-s001.zip › life-1592845-supplementary/Heermann-cluster-correlation-function-aec7e8-chr4.pdf]

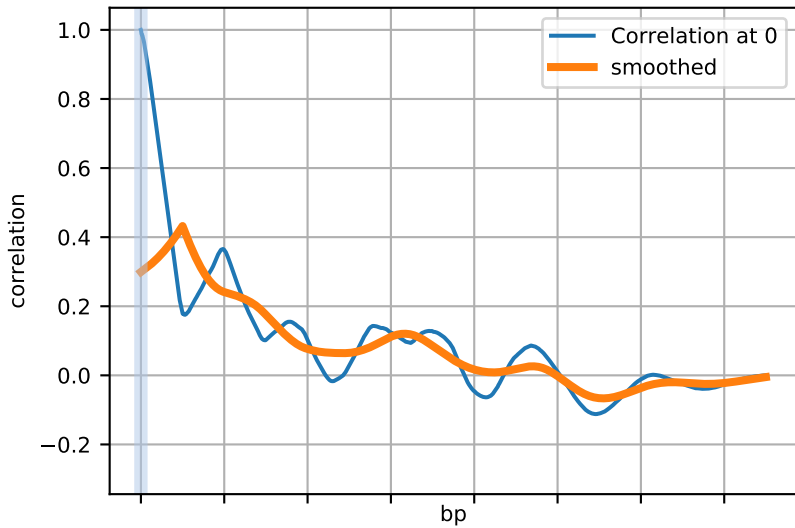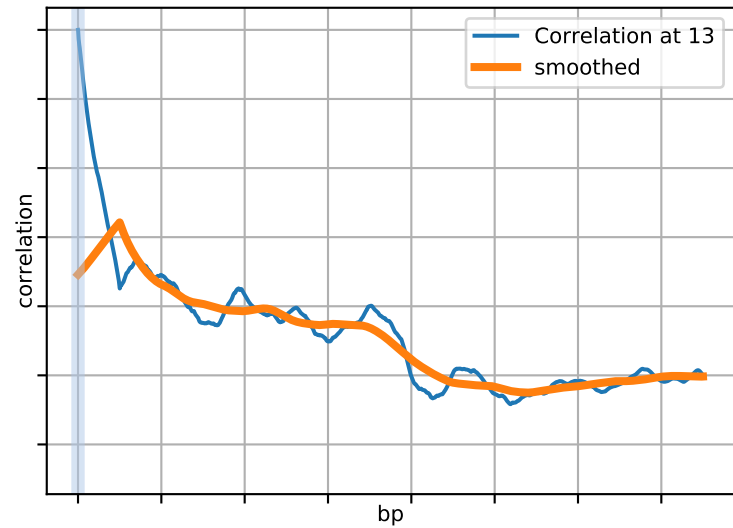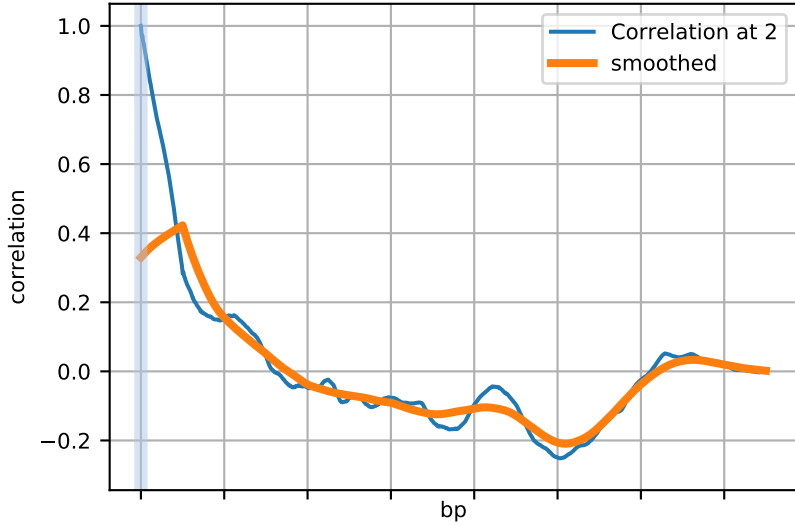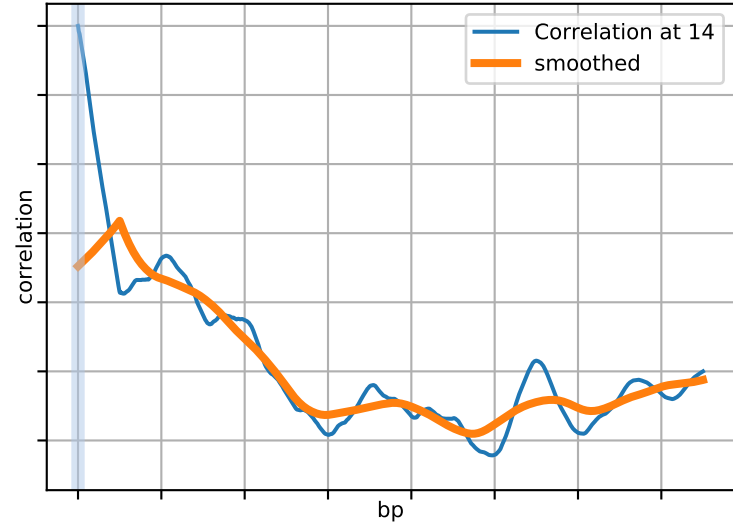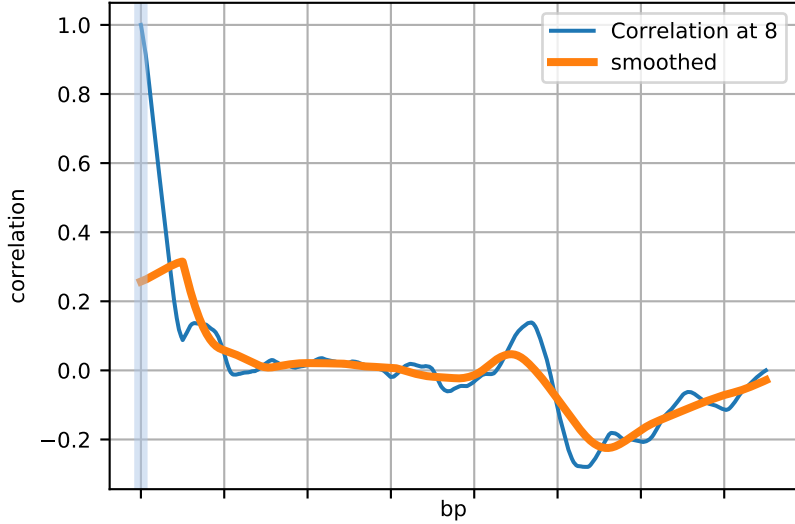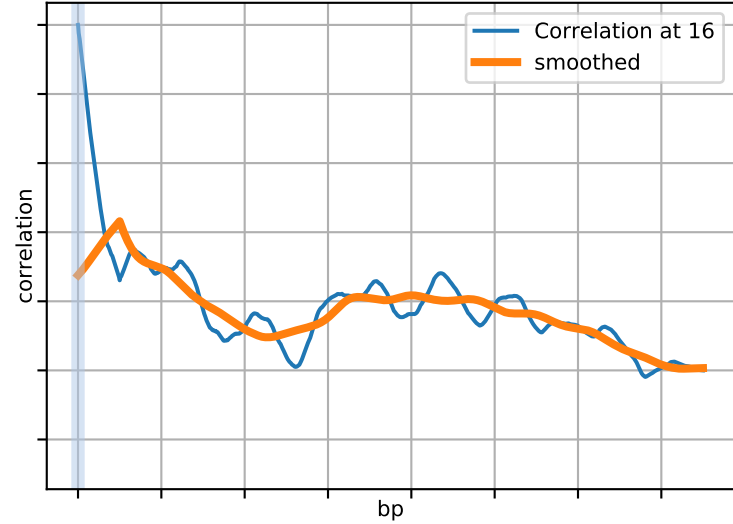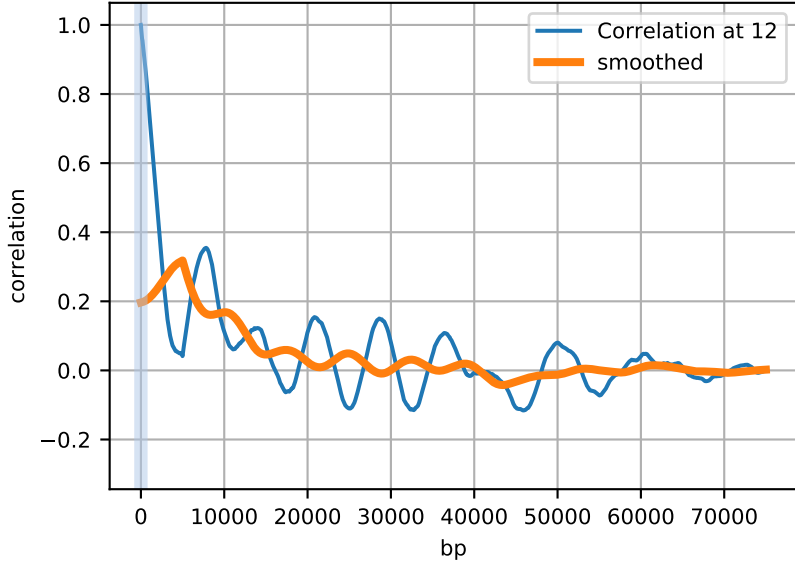

Supplement: Supplementary file 1 [file life-12-00541-s001.zip › life-1592845-supplementary/Heermann-cluster-correlation-function-aec7e8-chr5.pdf]

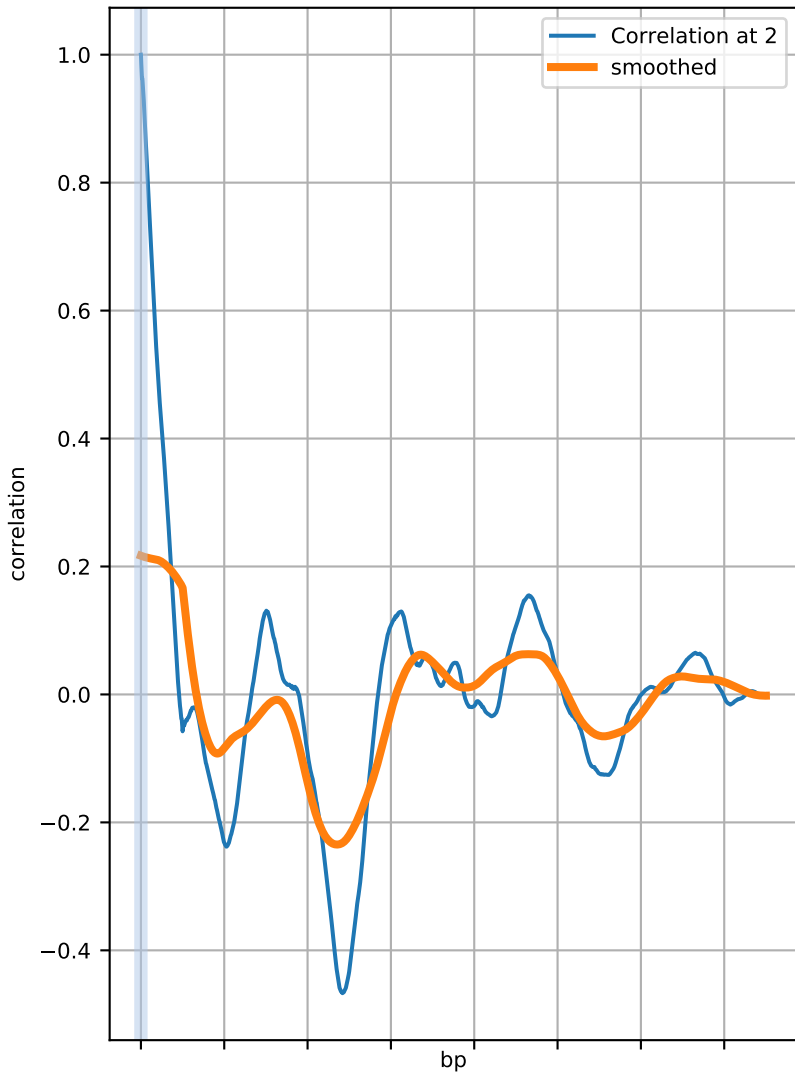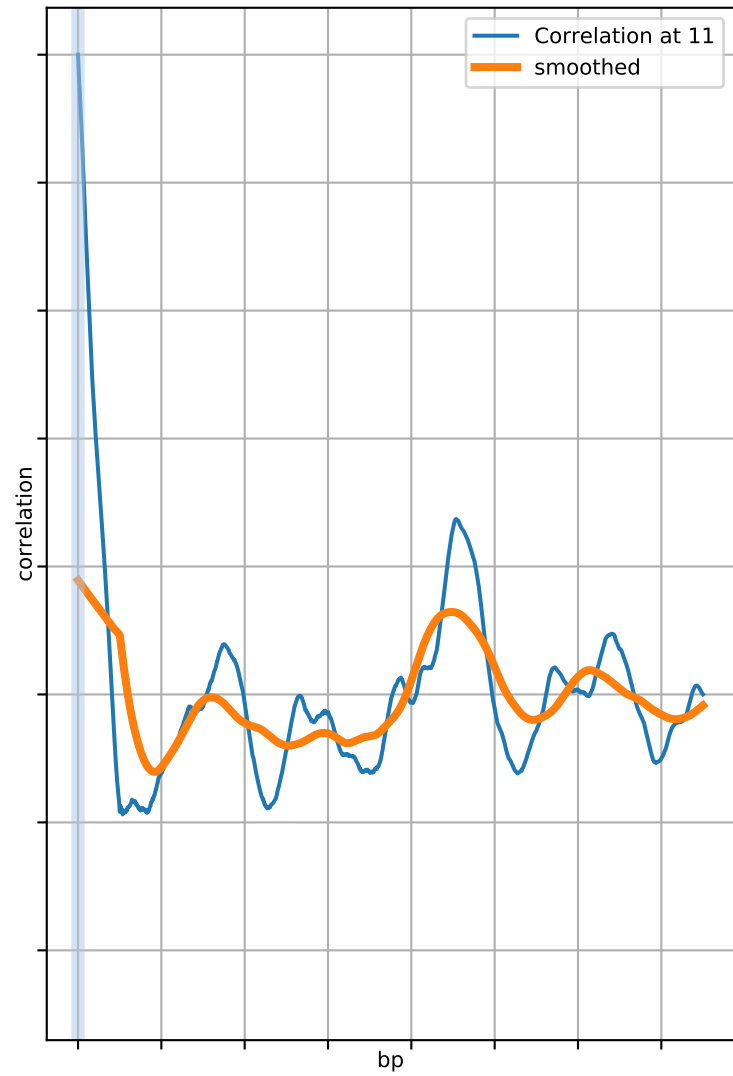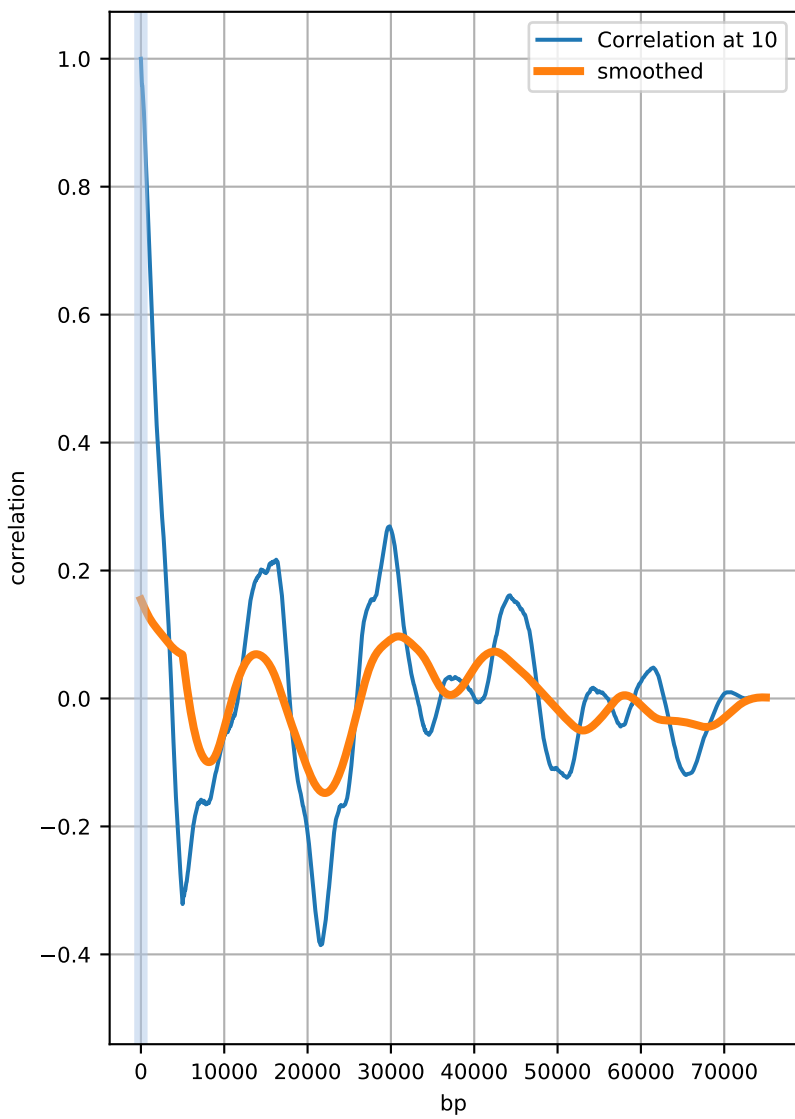

Supplement: Supplementary file 1 [file life-12-00541-s001.zip › life-1592845-supplementary/Heermann-cluster-correlation-function-aec7e8-chr6.pdf]

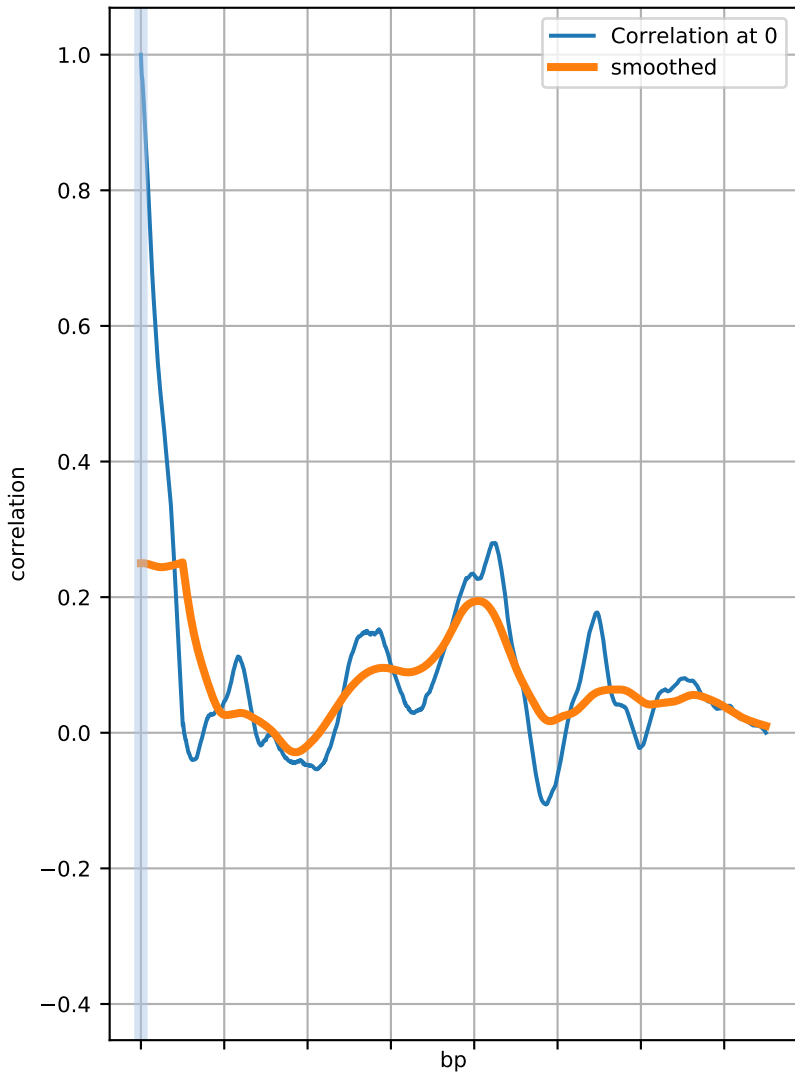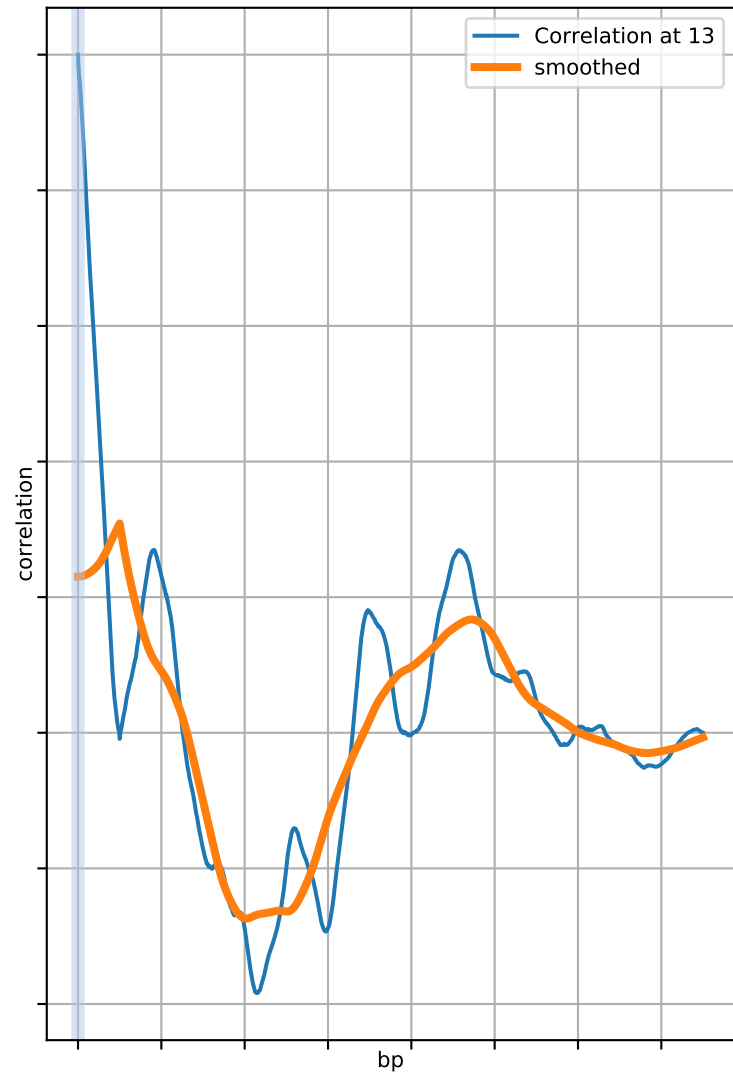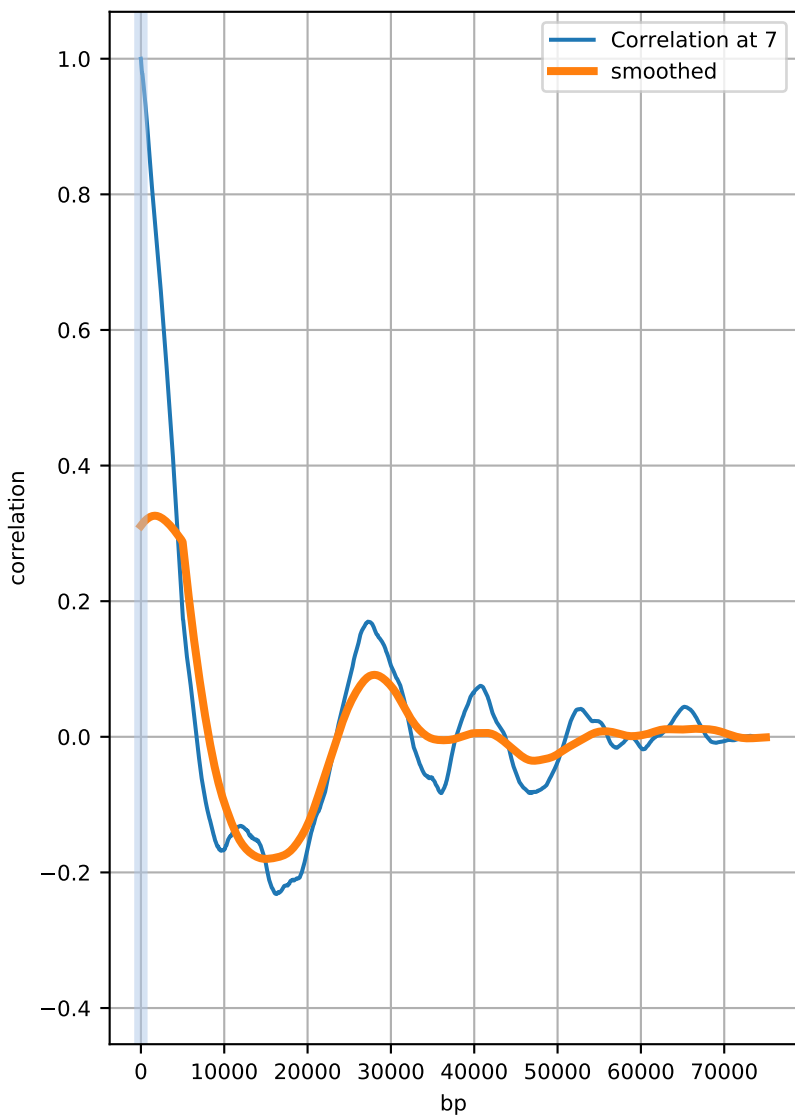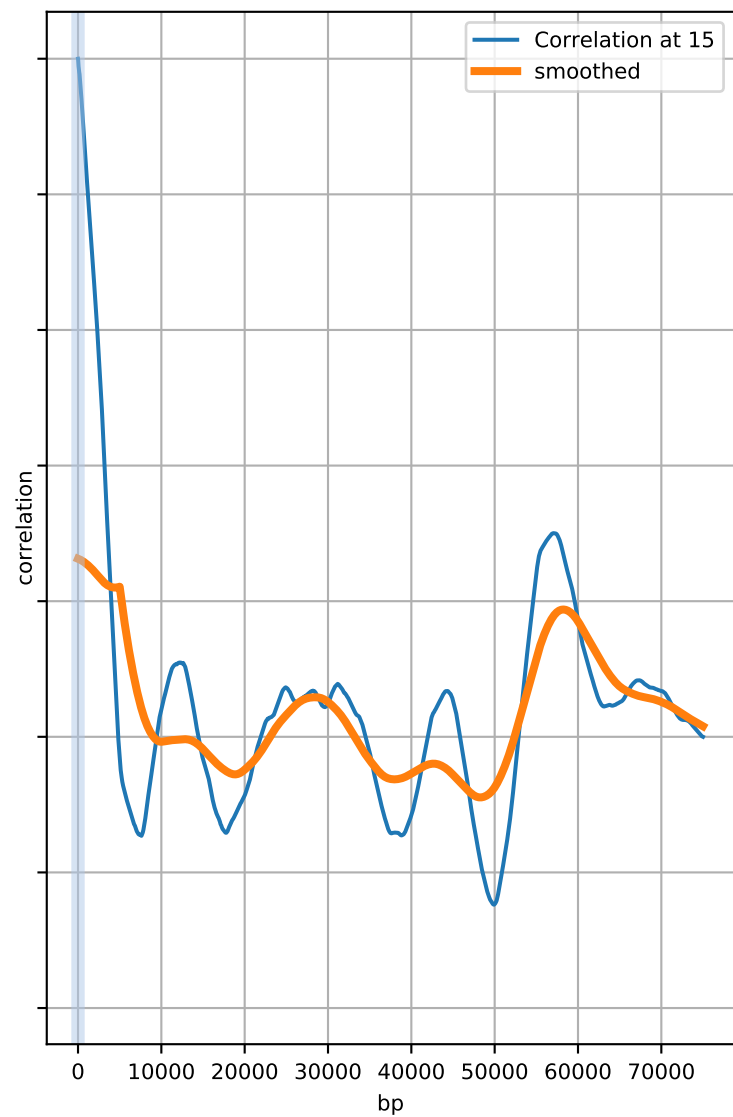

Supplement: Supplementary file 1 [file life-12-00541-s001.zip › life-1592845-supplementary/Heermann-cluster-correlation-function-aec7e8-chr7.pdf]

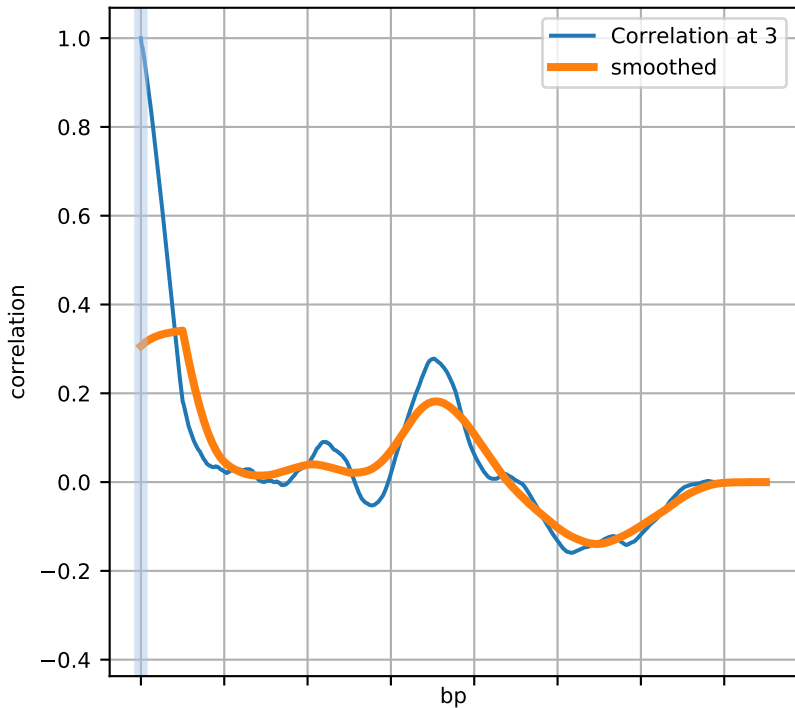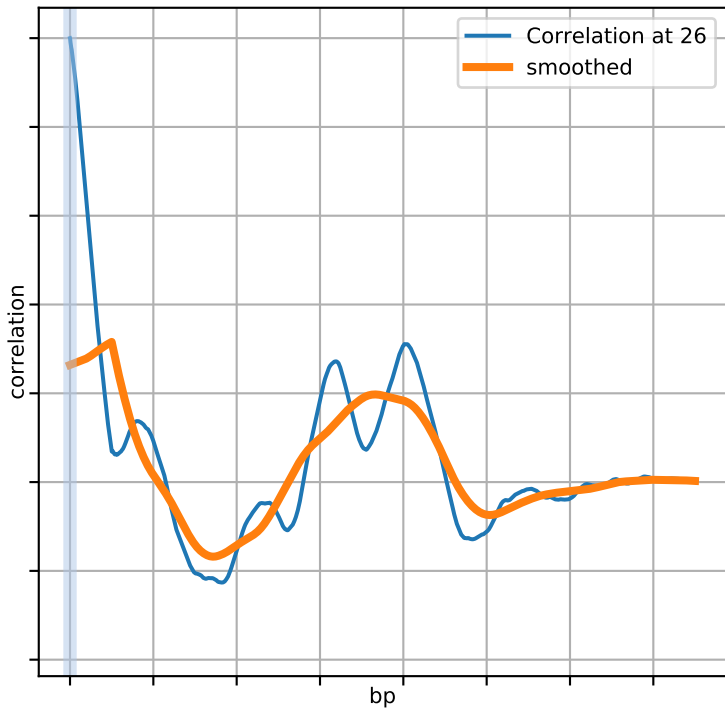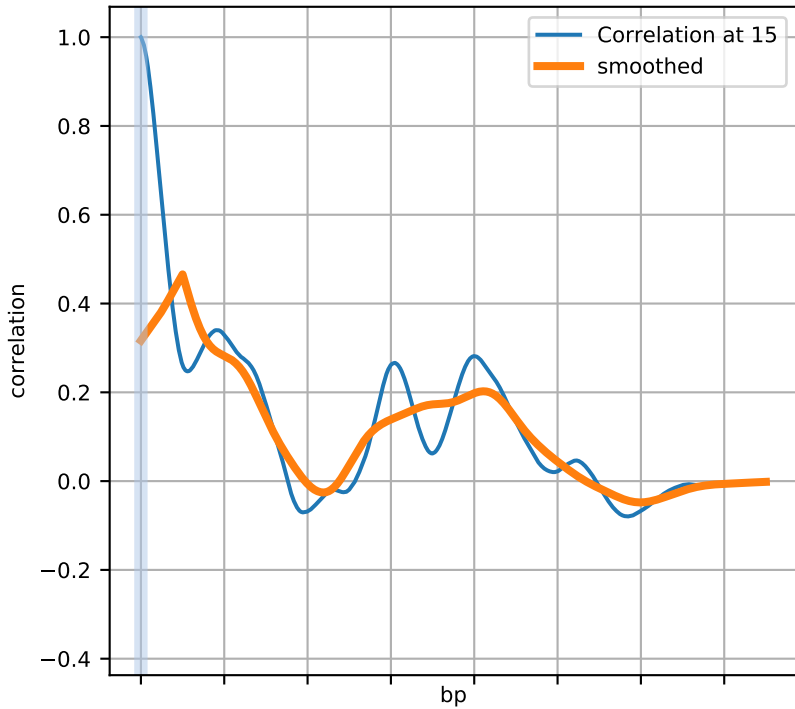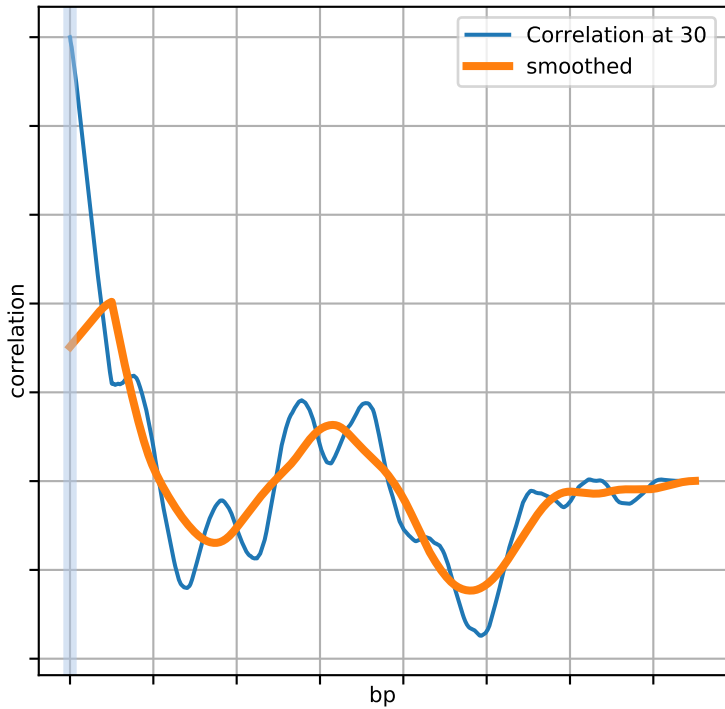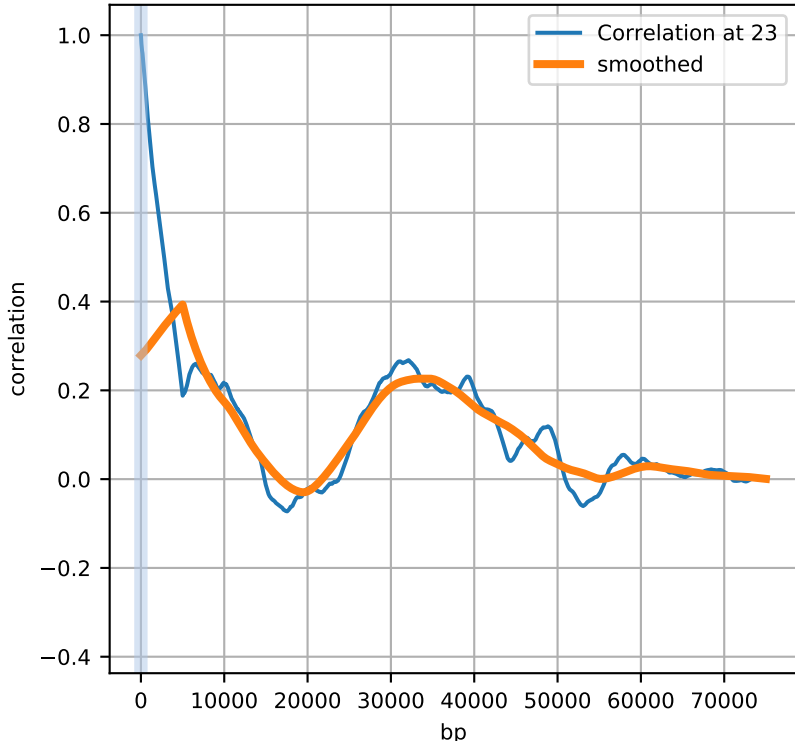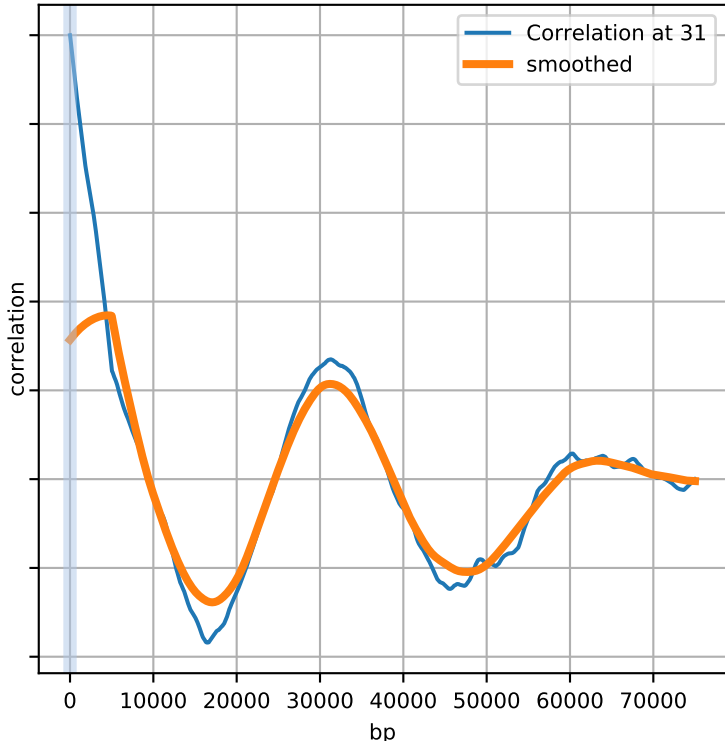

Supplement: Supplementary file 1 [file life-12-00541-s001.zip › life-1592845-supplementary/Heermann-cluster-correlation-function-aec7e8-chrR.pdf]

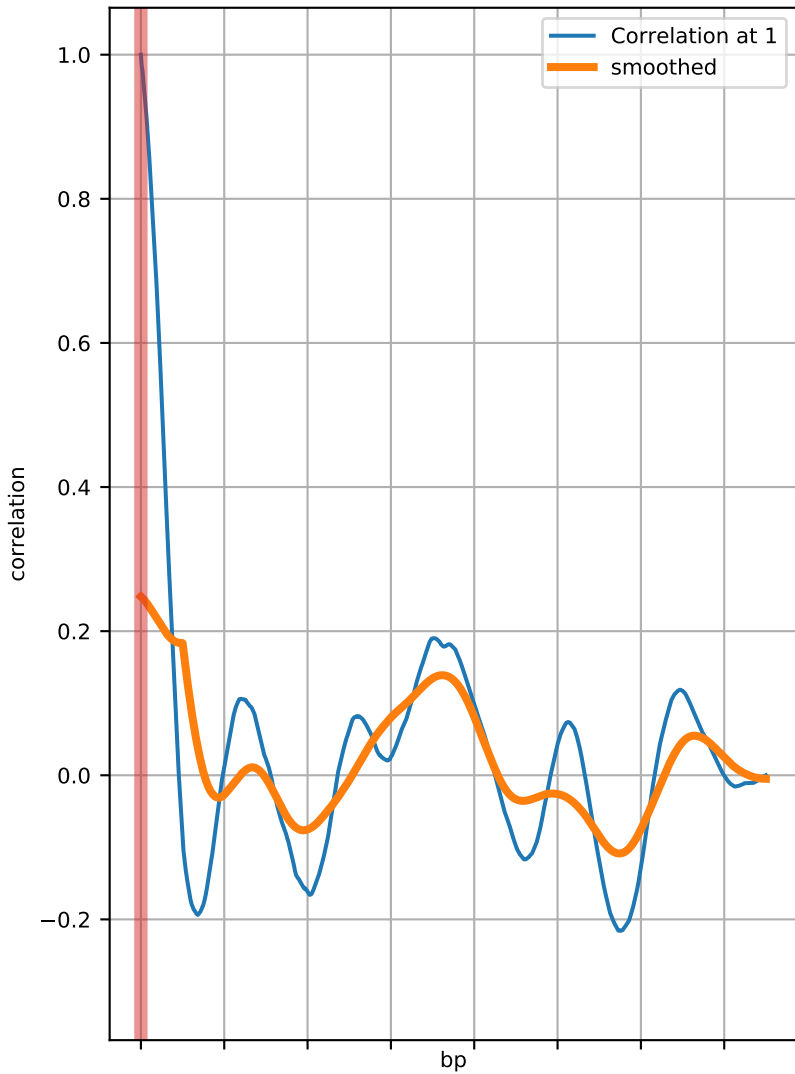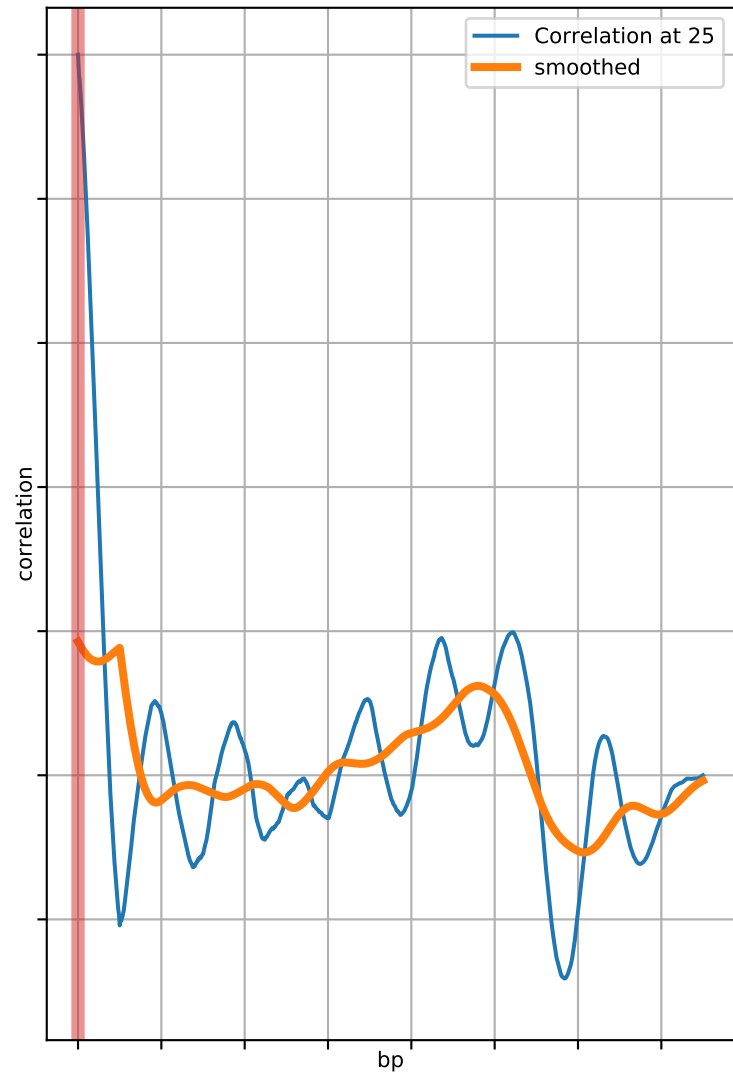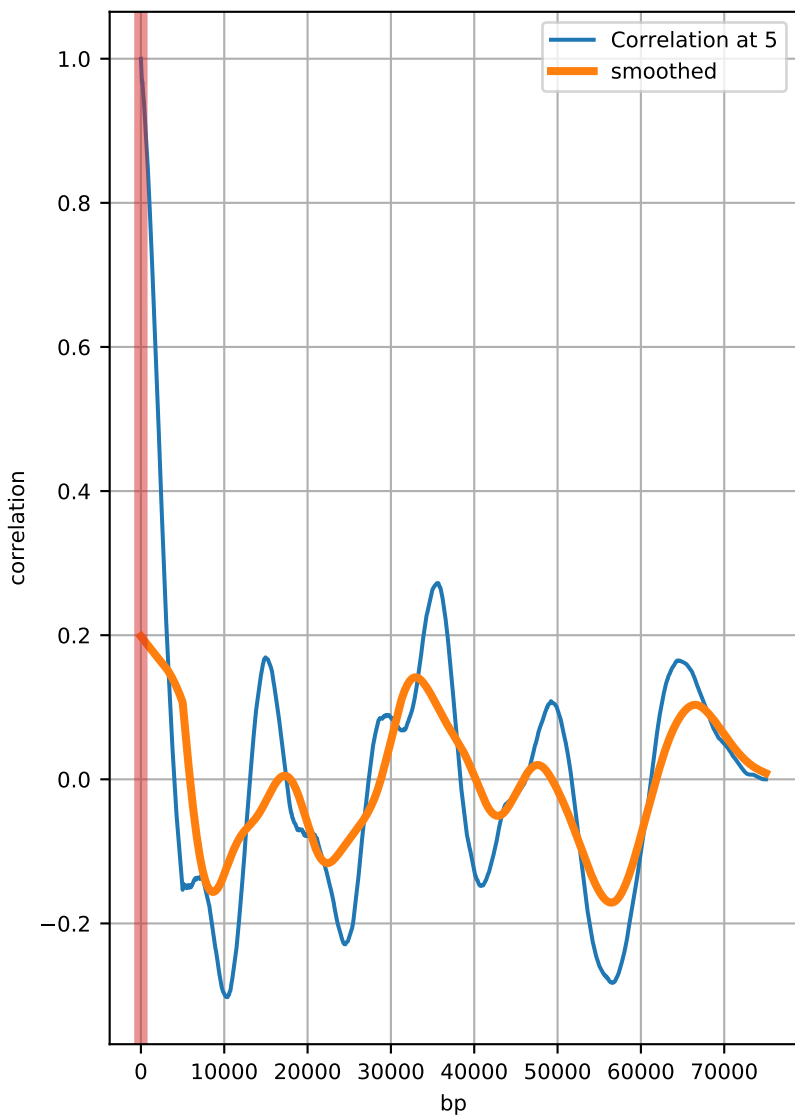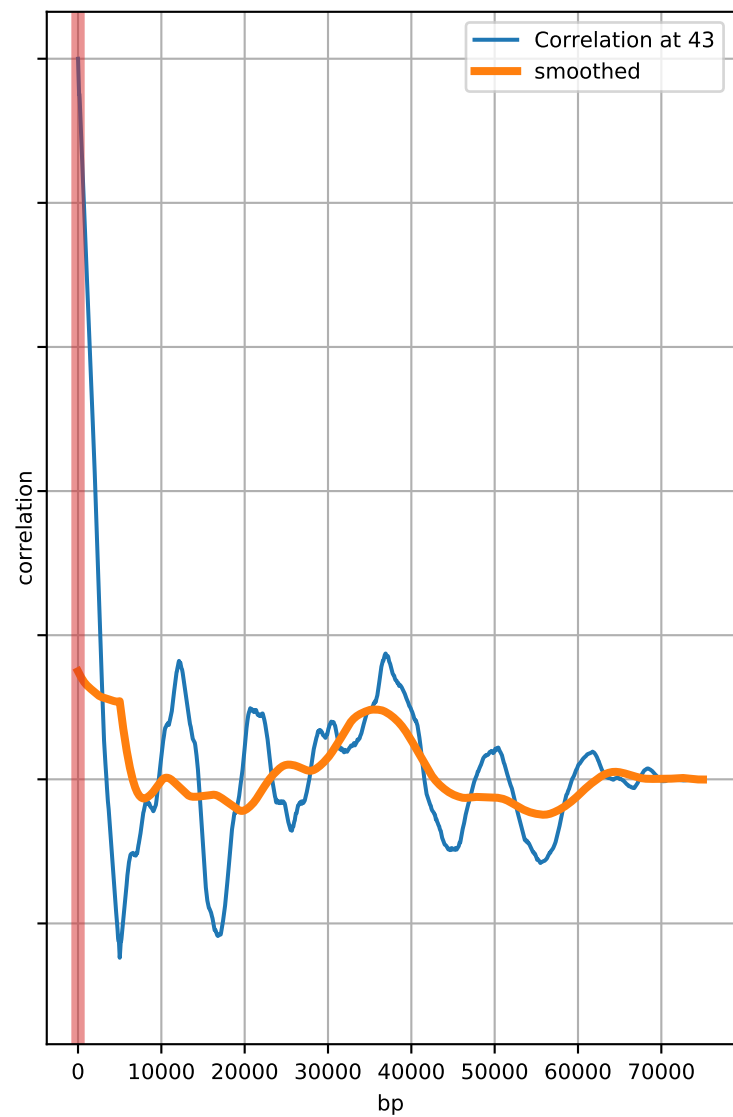

Supplement: Supplementary file 1 [file life-12-00541-s001.zip › life-1592845-supplementary/Heermann-cluster-correlation-function-d62728-chrR.pdf]

Correlation and Cluster Analysis for Chromosomes chr1-chr2-chr3-chr4-chr5-chr6-chr7-chrR

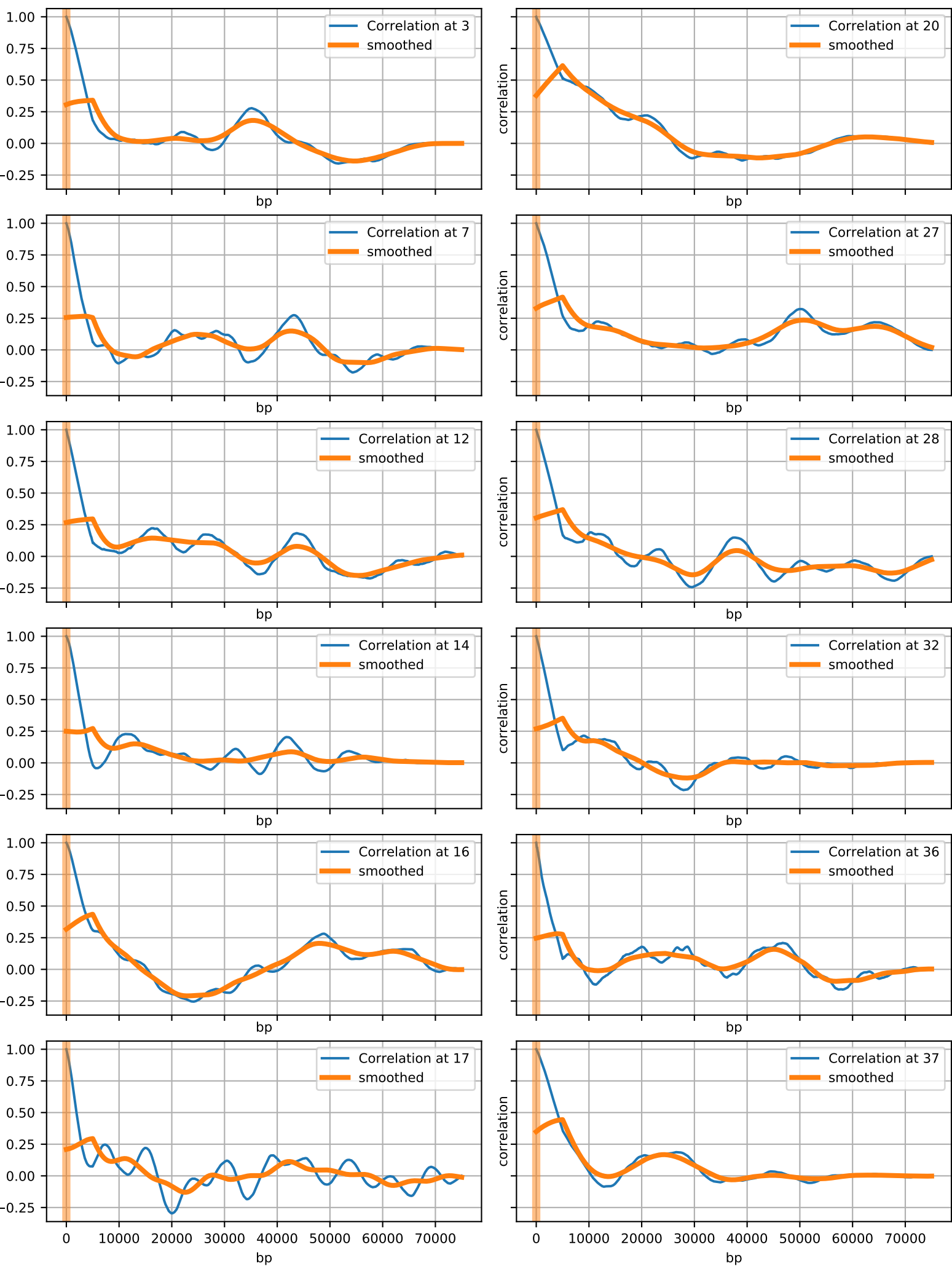

Supplement: Supplementary file 1 [file life-12-00541-s001.zip › life-1592845-supplementary/Heermann-cluster-correlation-function-ff7f0e-chr1-chr2-chr3-chr4-chr5-chr6-chr7-chrR-1.pdf]

Correlation and Cluster Analysis for Chromosomes chr1-chr2-chr3-chr4-chr5-chr6-chr7-chrR

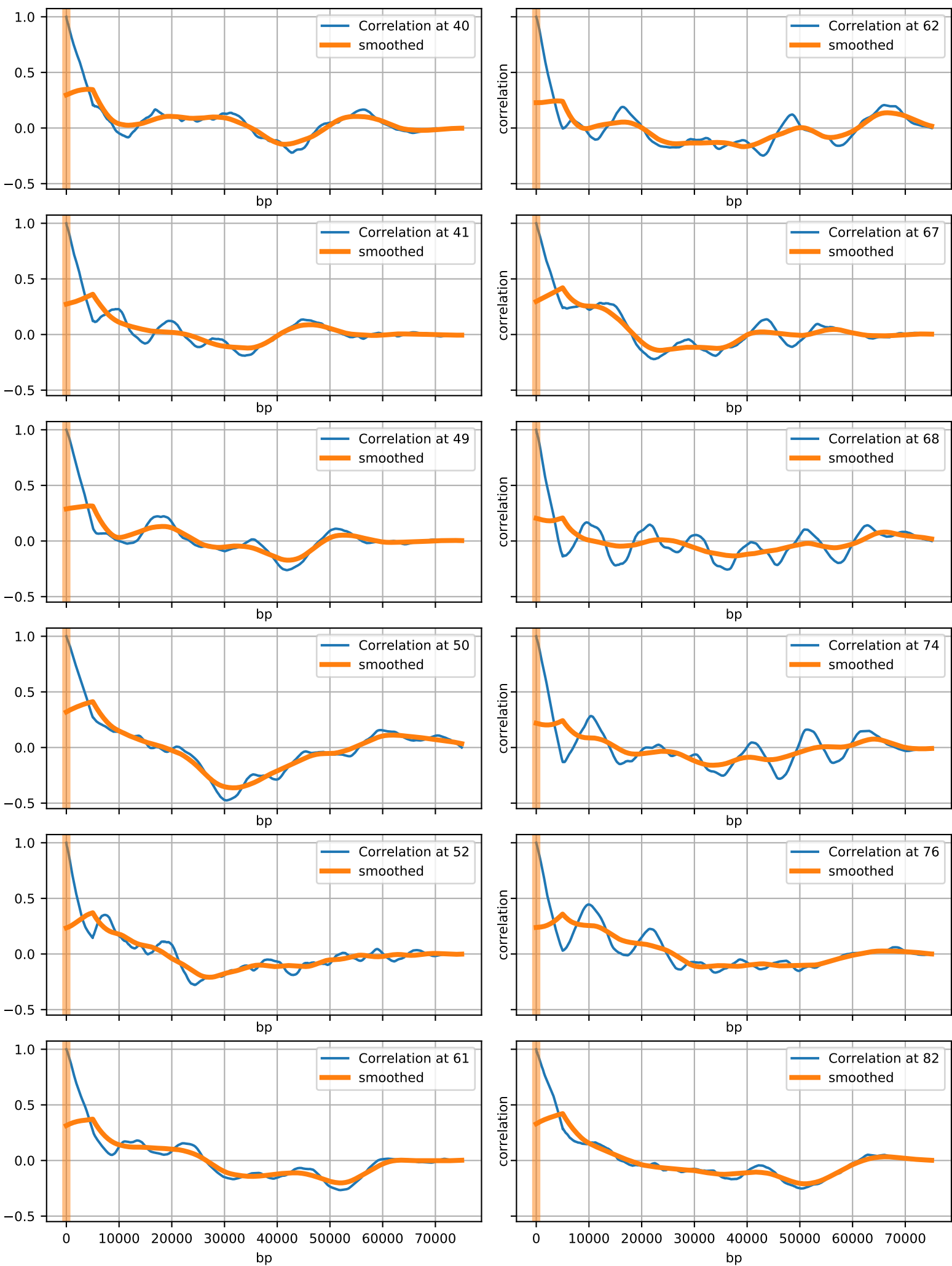

Supplement: Supplementary file 1 [file life-12-00541-s001.zip › life-1592845-supplementary/Heermann-cluster-correlation-function-ff7f0e-chr1-chr2-chr3-chr4-chr5-chr6-chr7-chrR-2.pdf]

Correlation and Cluster Analysis for Chromosomes chr1-chr2-chr3-chr4-chr5-chr6-chr7-chrR

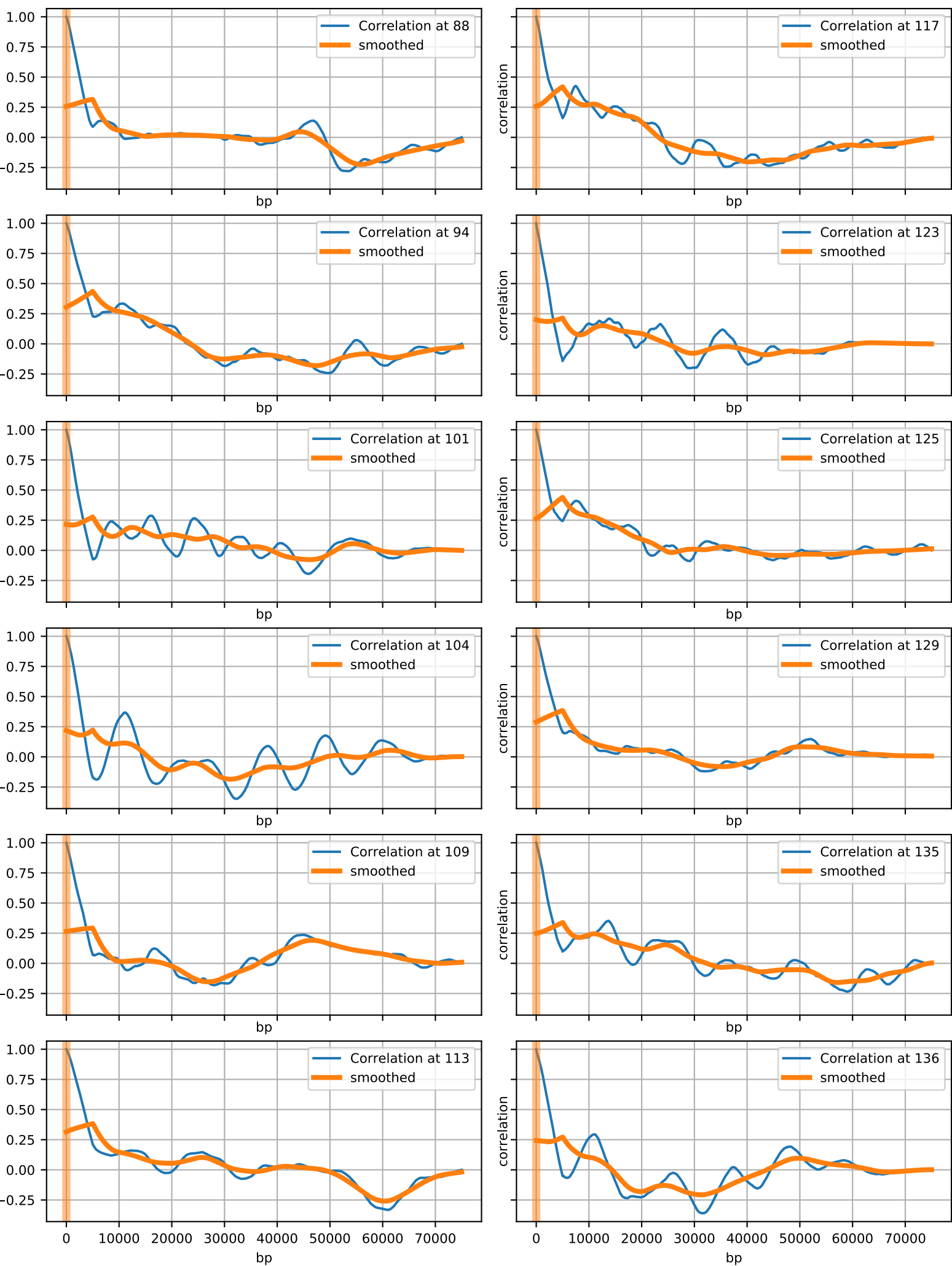

Supplement: Supplementary file 1 [file life-12-00541-s001.zip › life-1592845-supplementary/Heermann-cluster-correlation-function-ff7f0e-chr1-chr2-chr3-chr4-chr5-chr6-chr7-chrR-3.pdf]

Correlation and Cluster Analysis for Chromosomes chr1-chr2-chr3-chr4-chr5-chr6-chr7-chrR

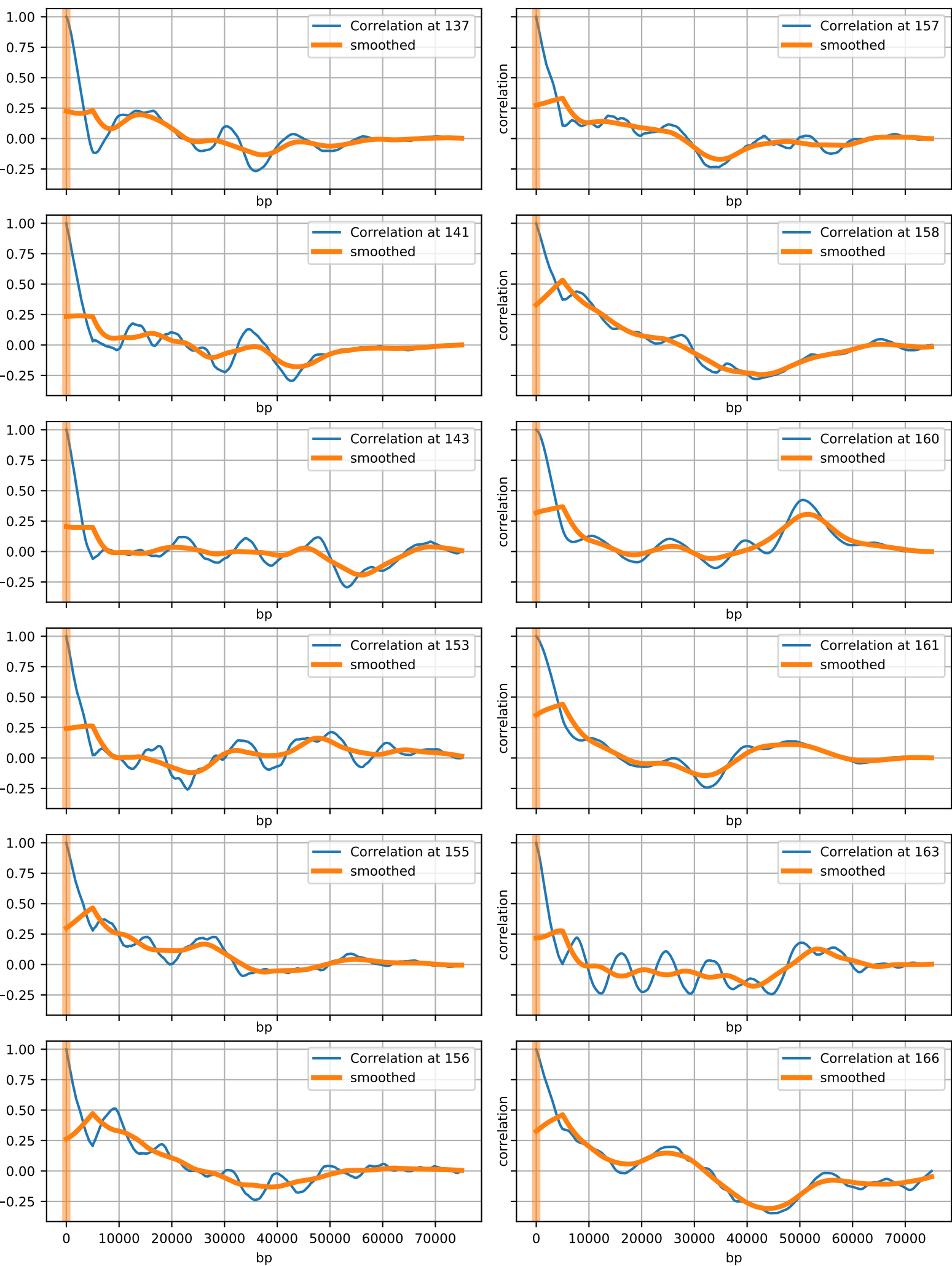

Supplement: Supplementary file 1 [file life-12-00541-s001.zip › life-1592845-supplementary/Heermann-cluster-correlation-function-ff7f0e-chr1-chr2-chr3-chr4-chr5-chr6-chr7-chrR-4.pdf]

Correlation and Cluster Analysis for Chromosomes chr1-chr2-chr3-chr4-chr5-chr6-chr7-chrR

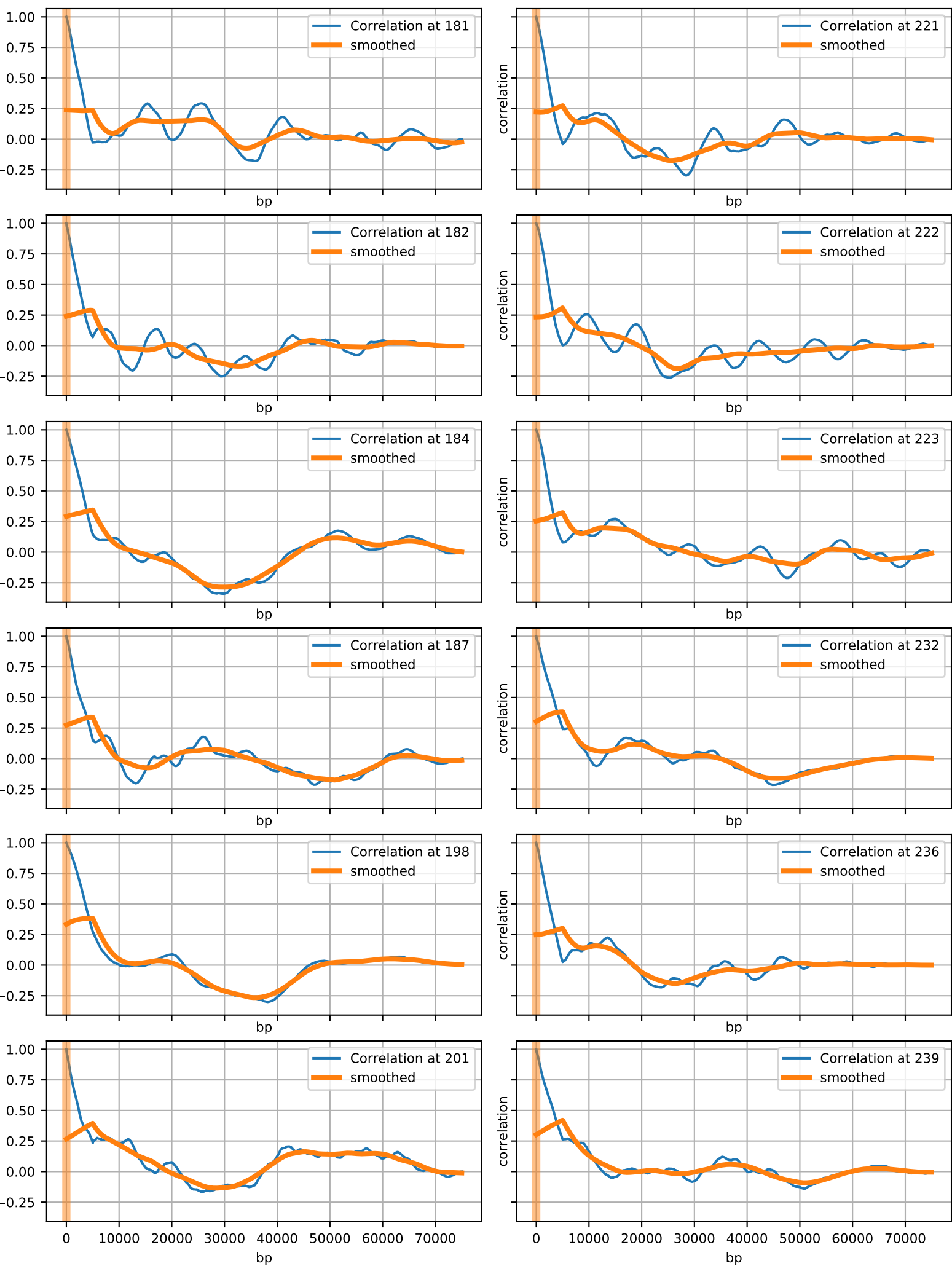

Supplement: Supplementary file 1 [file life-12-00541-s001.zip › life-1592845-supplementary/Heermann-cluster-correlation-function-ff7f0e-chr1-chr2-chr3-chr4-chr5-chr6-chr7-chrR-5.pdf]

Correlation and Cluster Analysis for Chromosomes chr1-chr2-chr3-chr4-chr5-chr6-chr7-chrR

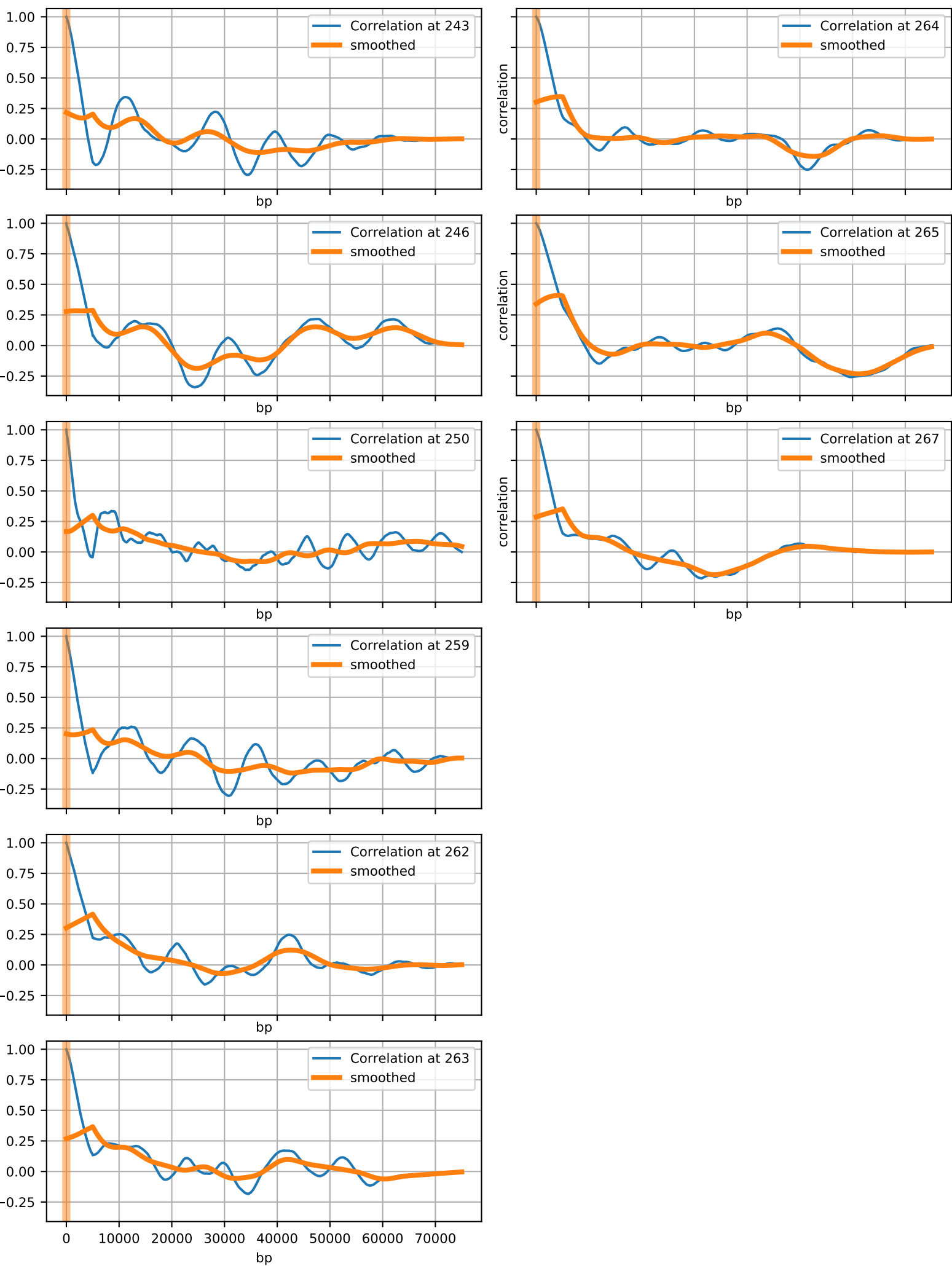

Supplement: Supplementary file 1 [file life-12-00541-s001.zip › life-1592845-supplementary/Heermann-cluster-correlation-function-ff7f0e-chr1-chr2-chr3-chr4-chr5-chr6-chr7-chrR-6.pdf]

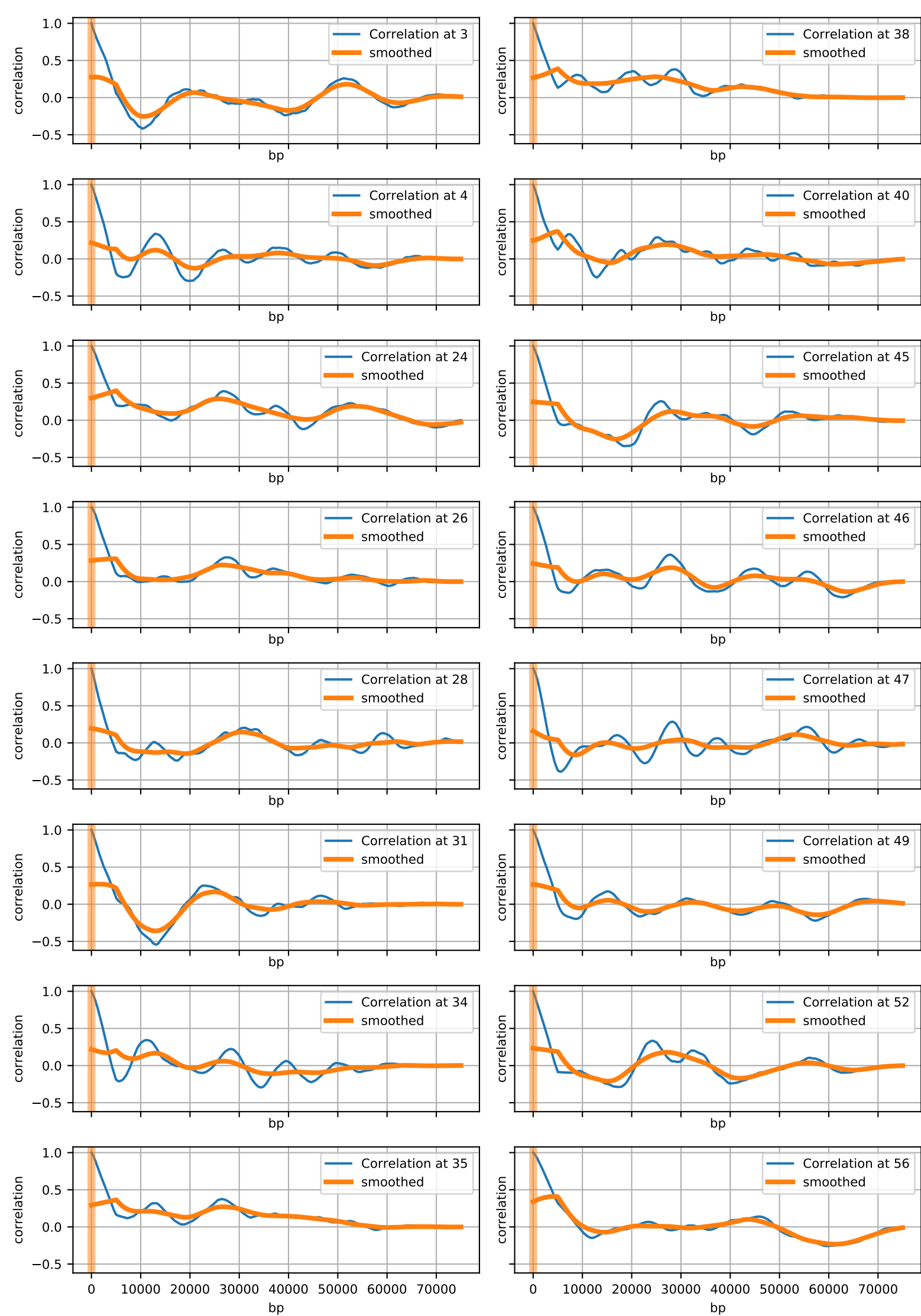

Supplement: Supplementary file 1 [file life-12-00541-s001.zip › life-1592845-supplementary/Heermann-cluster-correlation-function-ff7f0e-chr1.pdf]

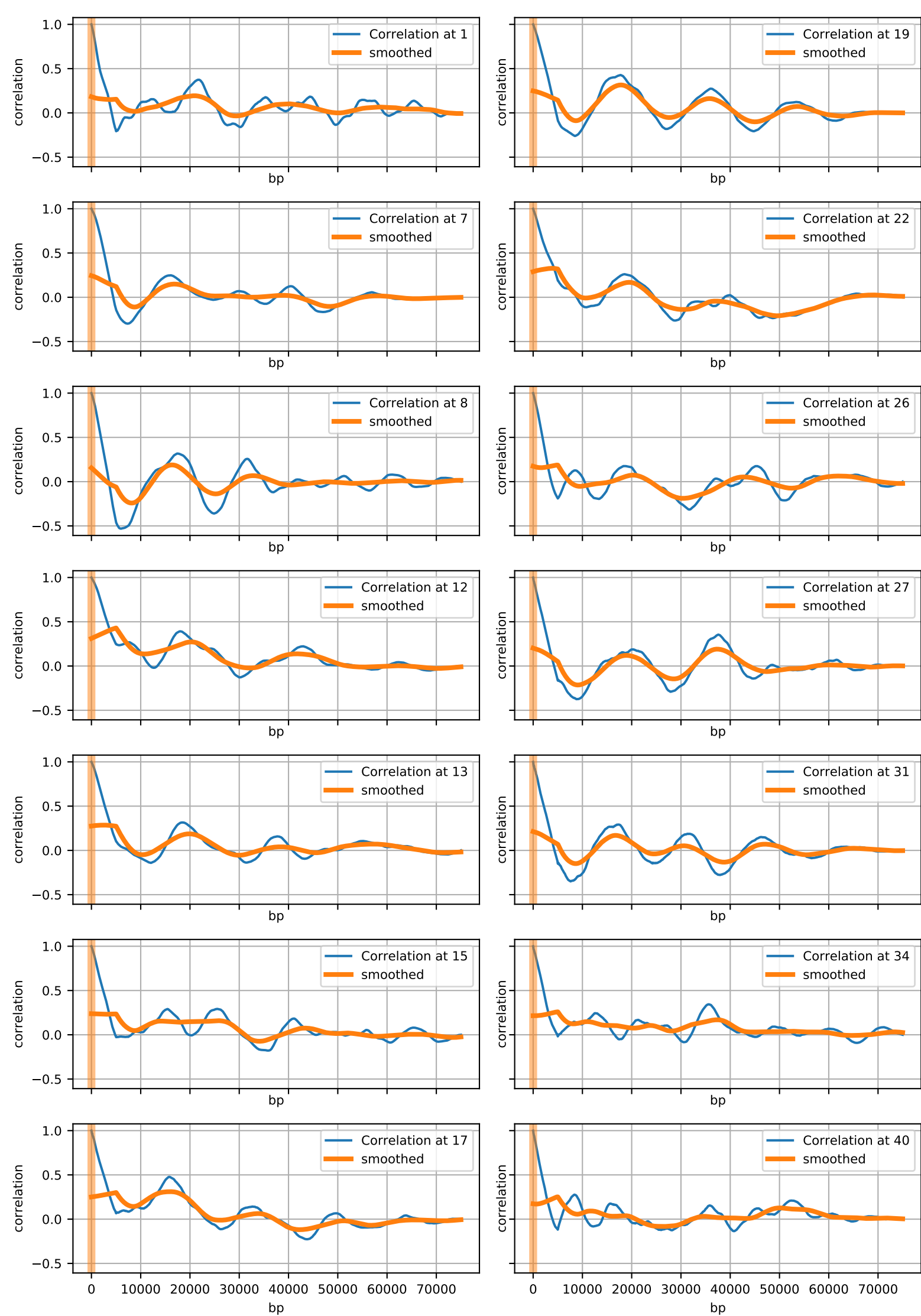

Supplement: Supplementary file 1 [file life-12-00541-s001.zip › life-1592845-supplementary/Heermann-cluster-correlation-function-ff7f0e-chr2.pdf]

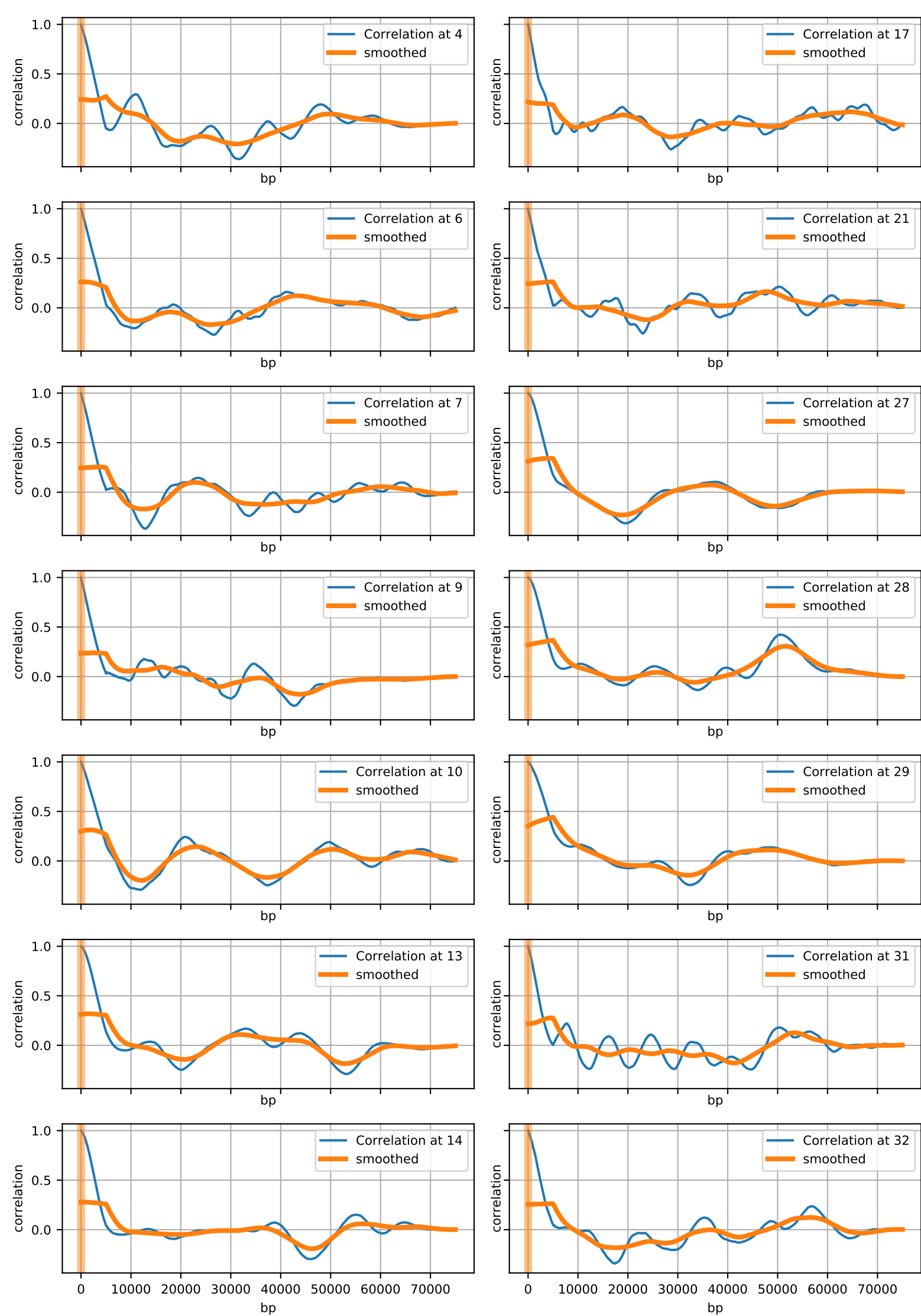

Supplement: Supplementary file 1 [file life-12-00541-s001.zip › life-1592845-supplementary/Heermann-cluster-correlation-function-ff7f0e-chr3.pdf]

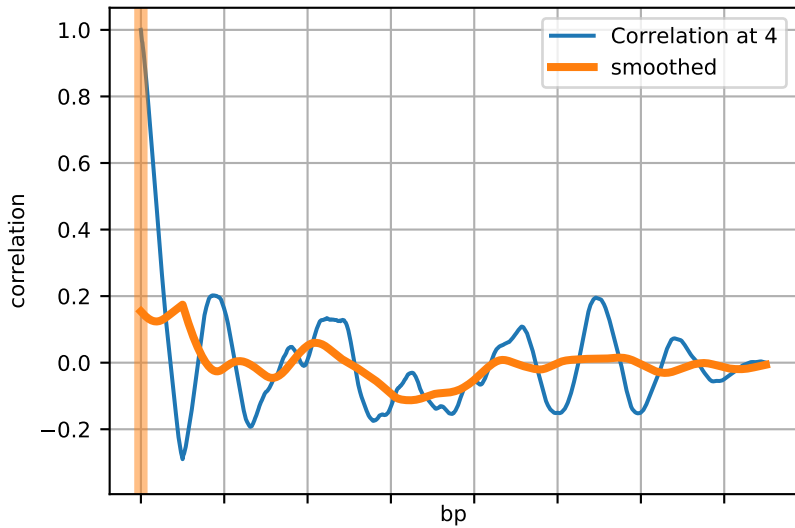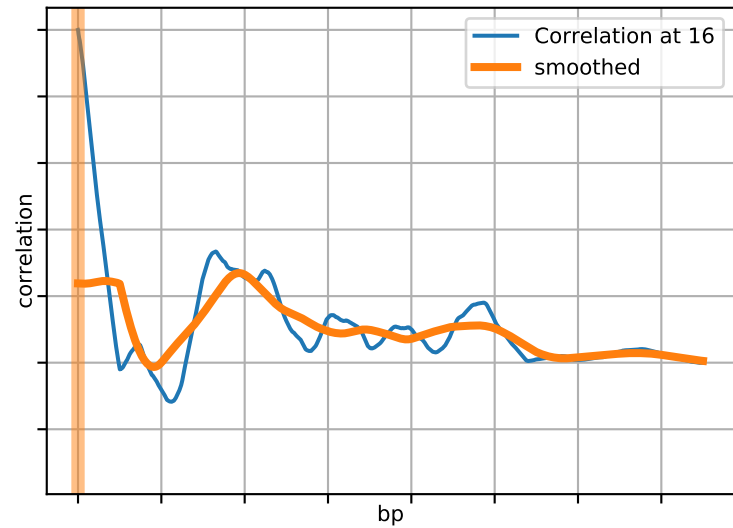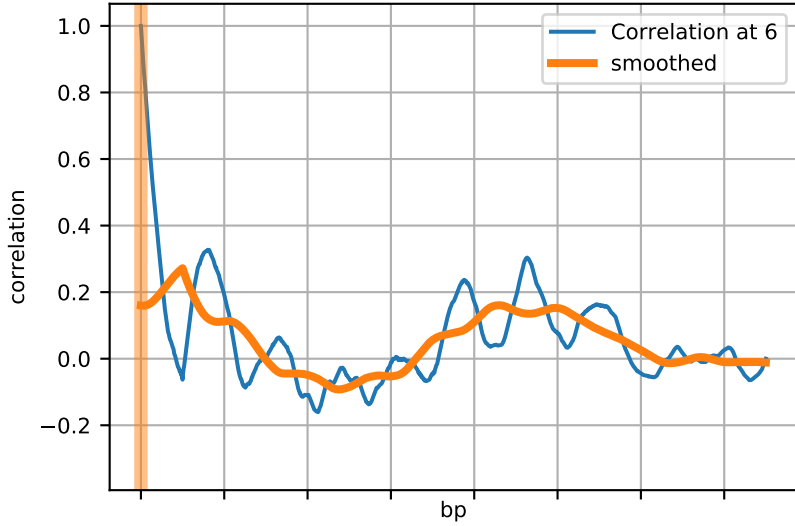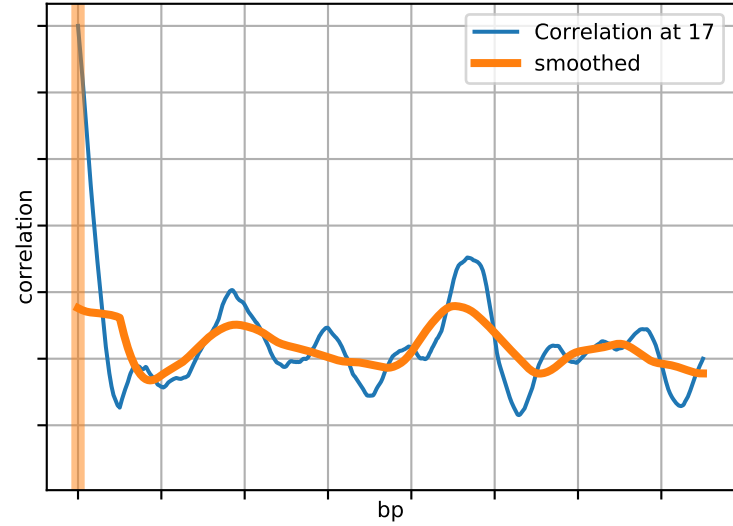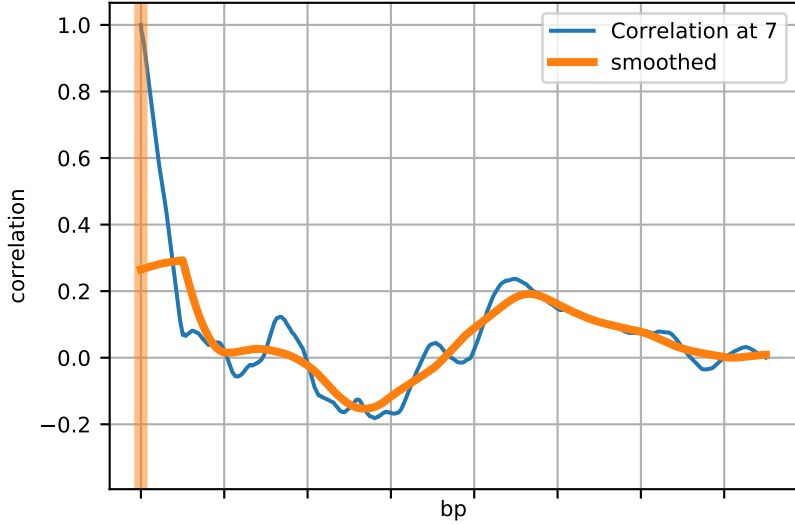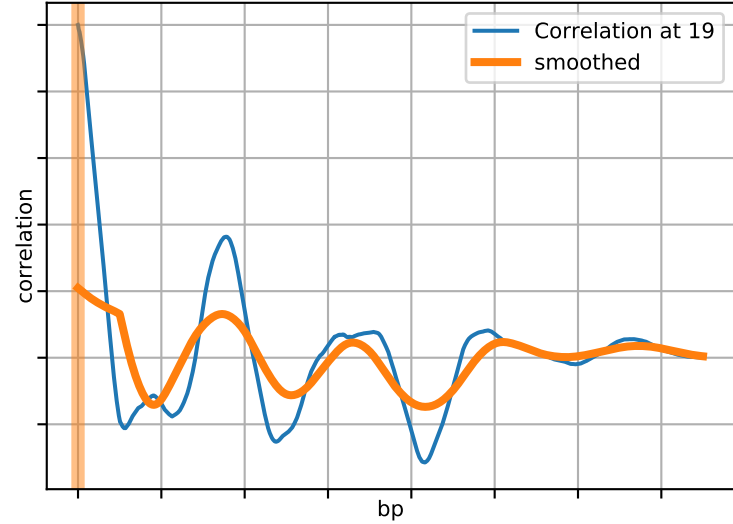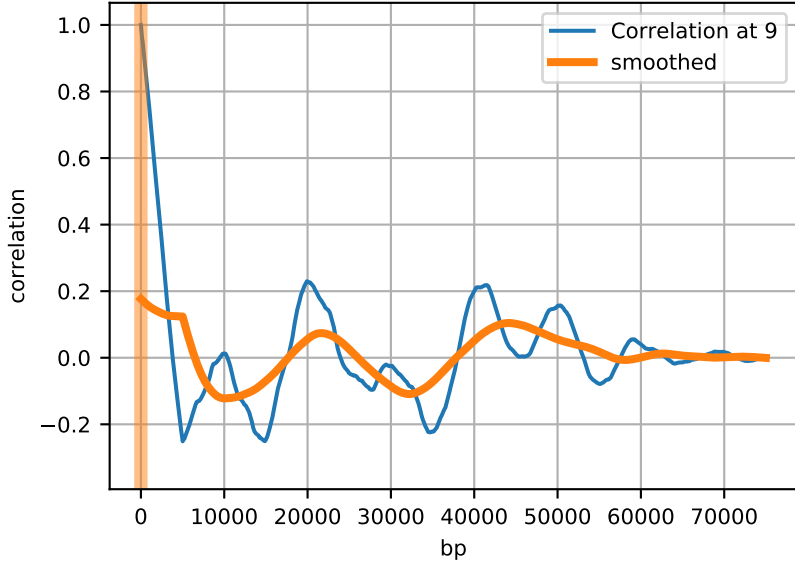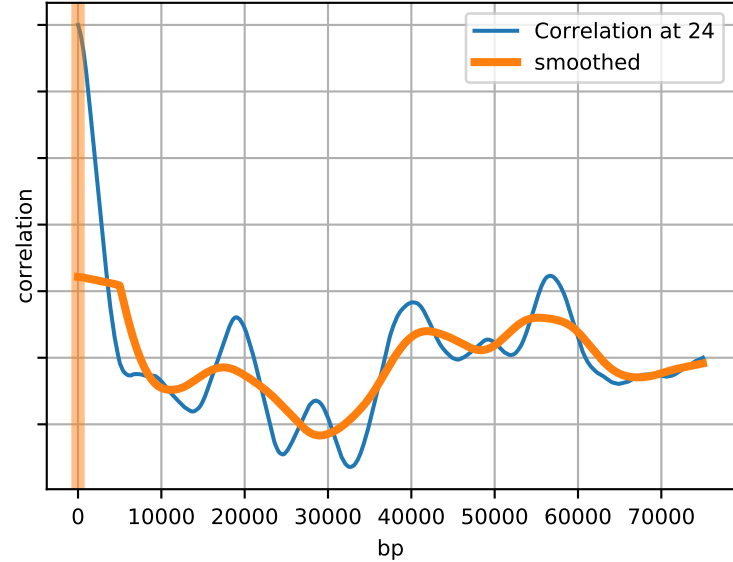

Supplement: Supplementary file 1 [file life-12-00541-s001.zip › life-1592845-supplementary/Heermann-cluster-correlation-function-ff7f0e-chr4.pdf]

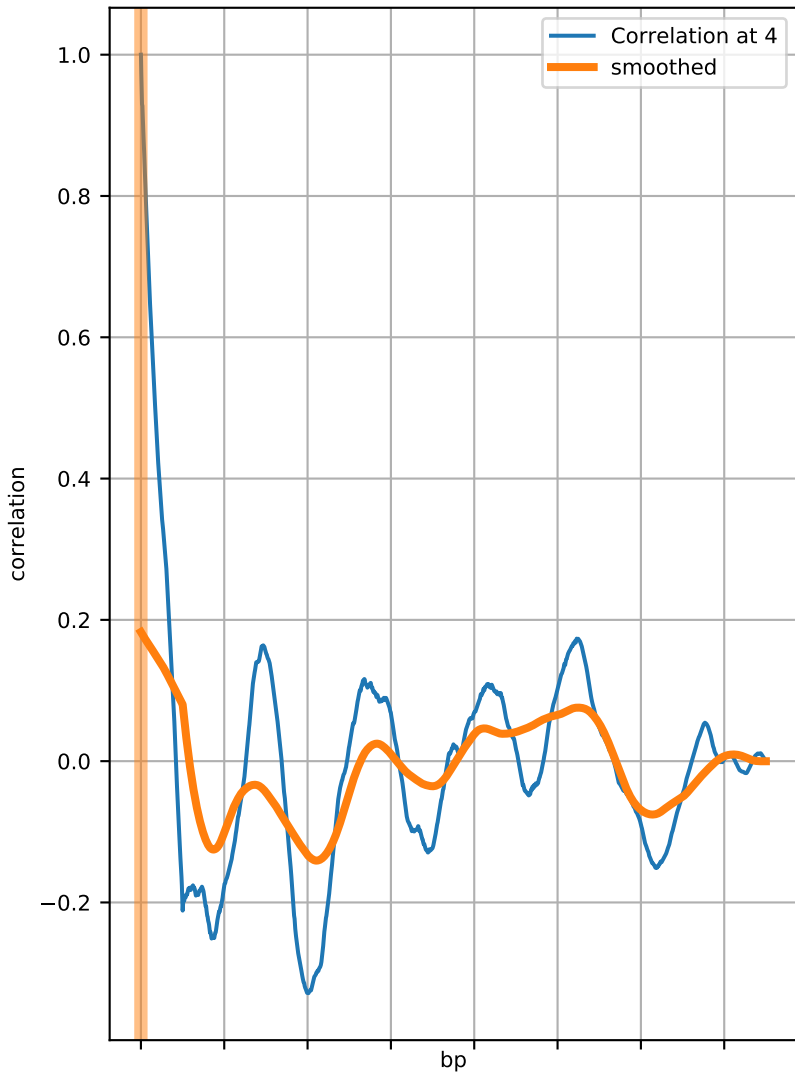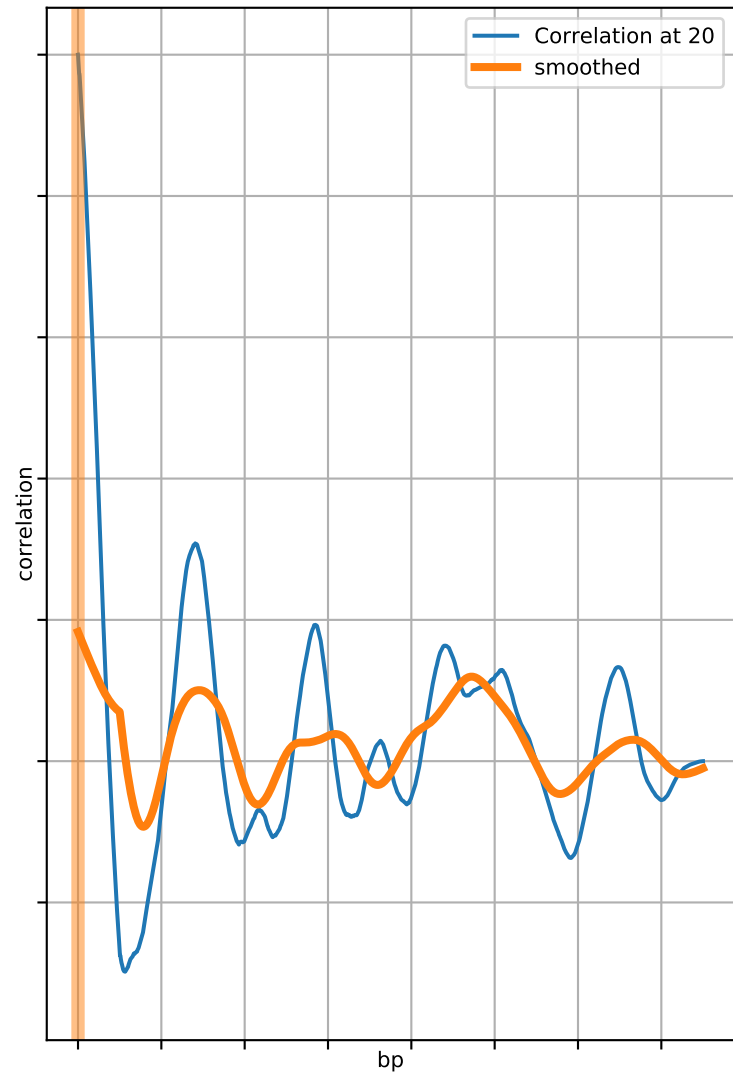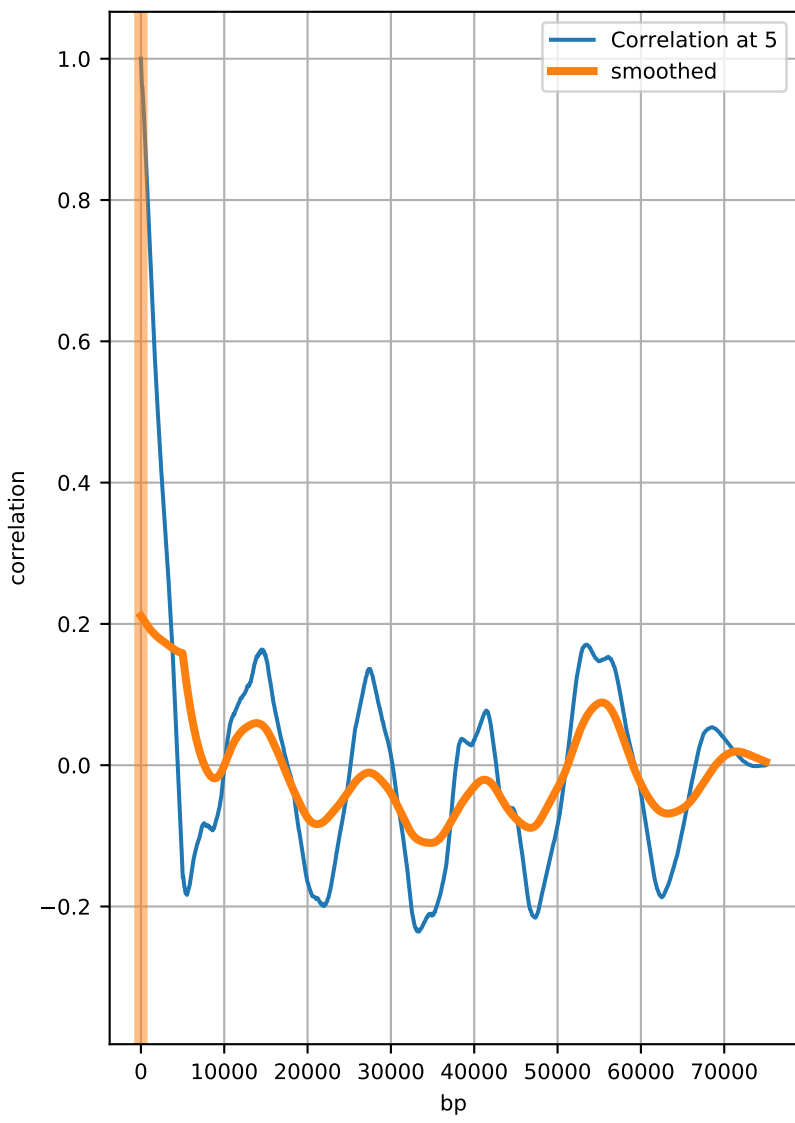

Supplement: Supplementary file 1 [file life-12-00541-s001.zip › life-1592845-supplementary/Heermann-cluster-correlation-function-ff7f0e-chr5.pdf]

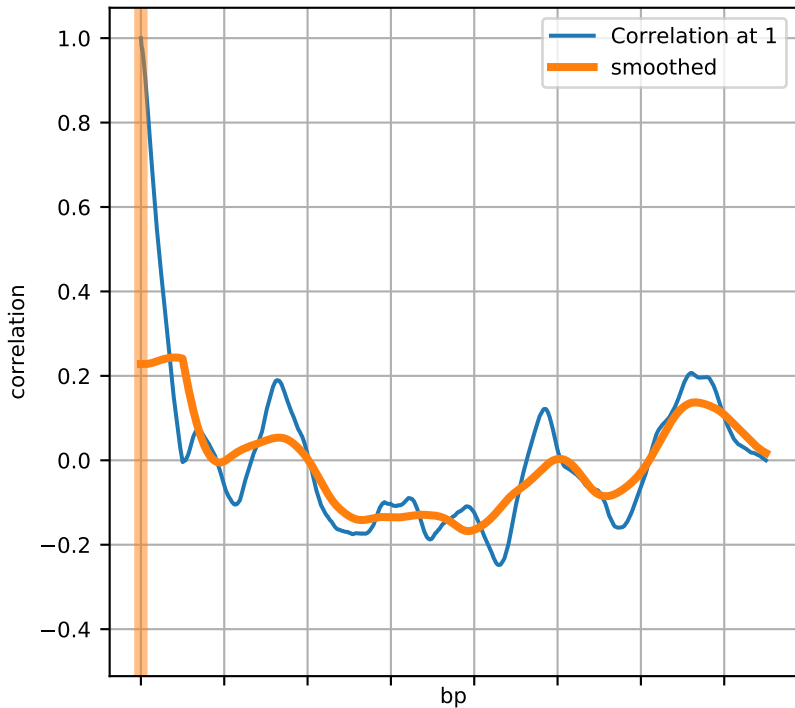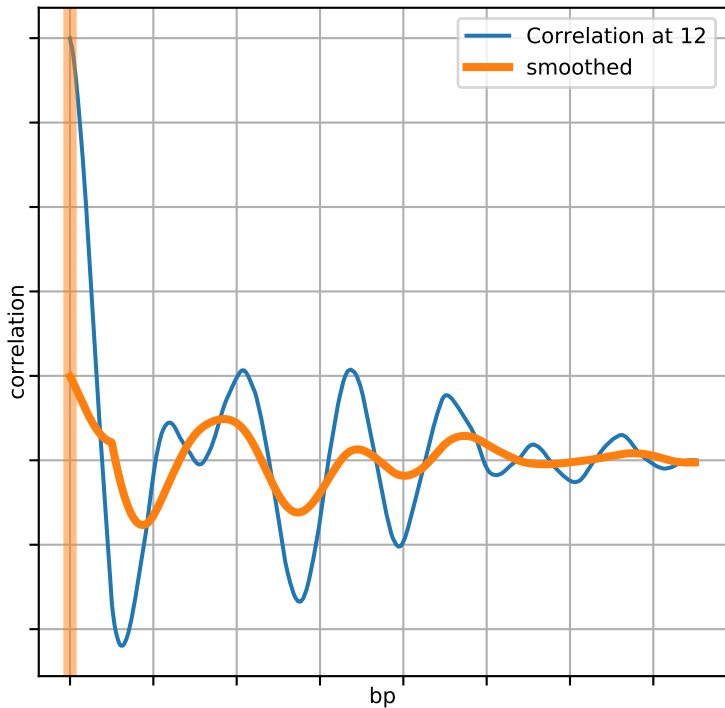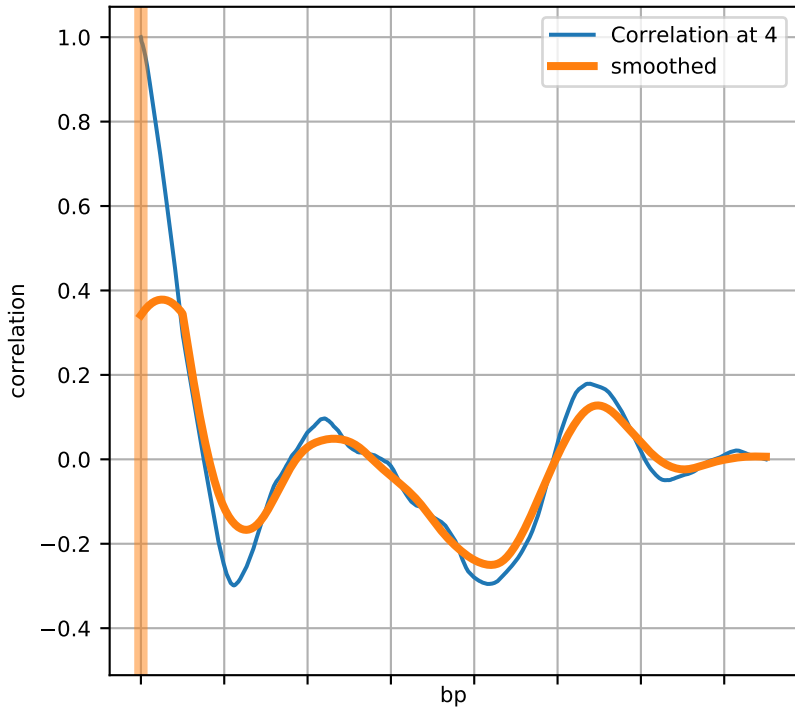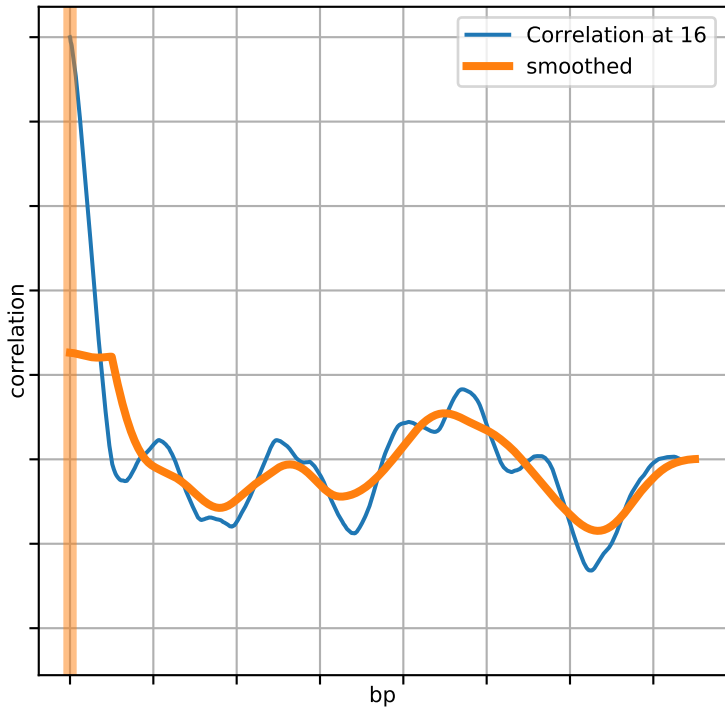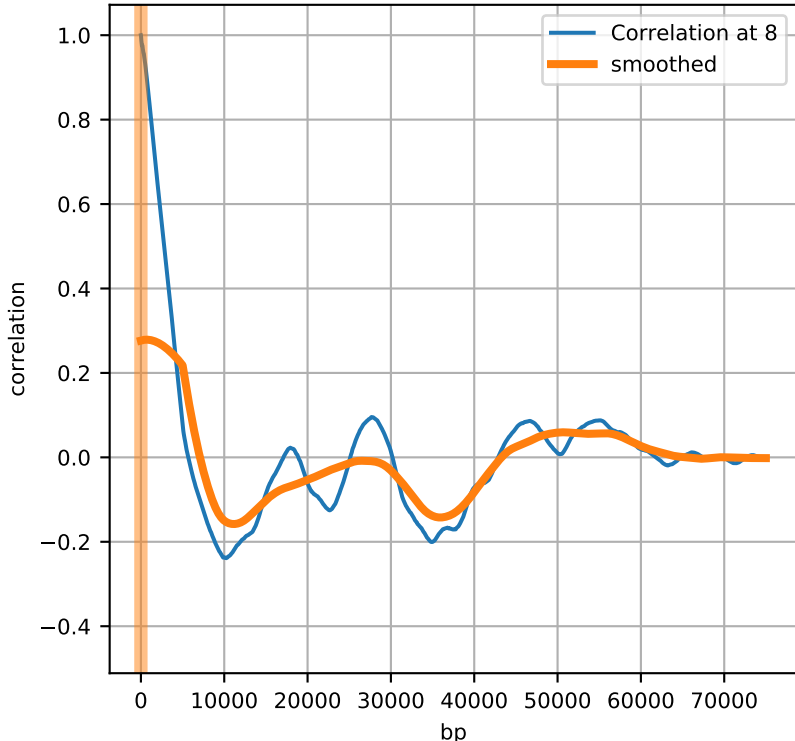

Supplement: Supplementary file 1 [file life-12-00541-s001.zip › life-1592845-supplementary/Heermann-cluster-correlation-function-ff7f0e-chr6.pdf]

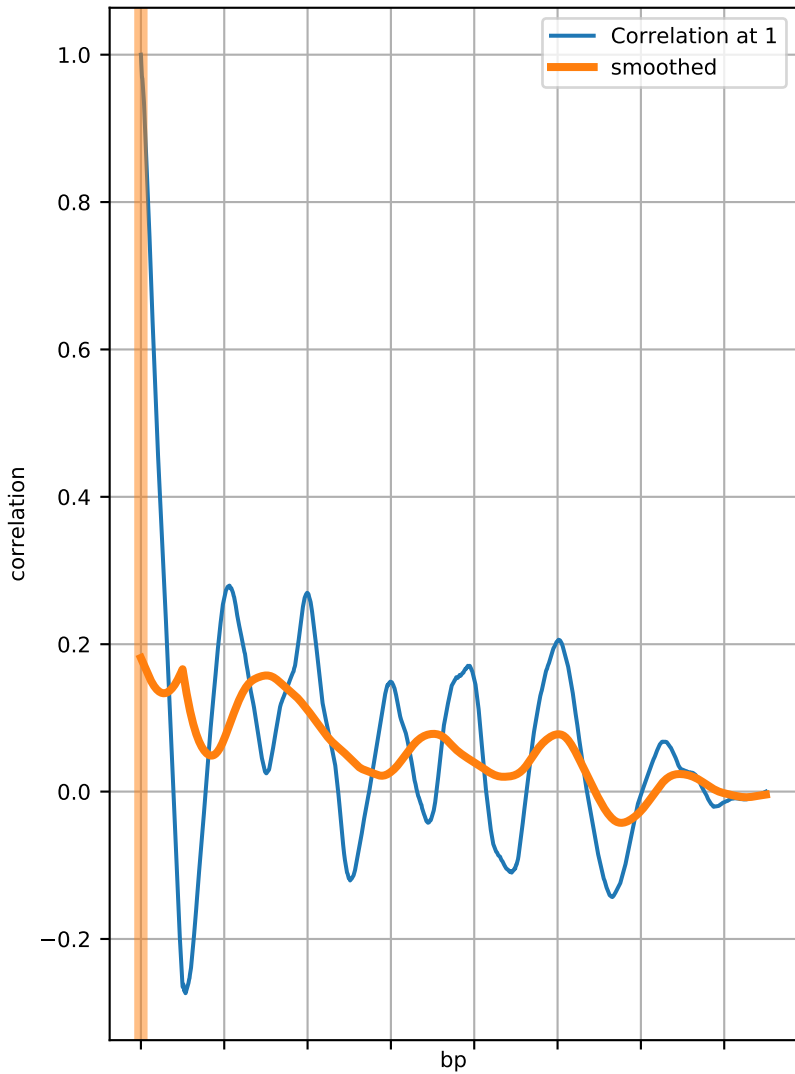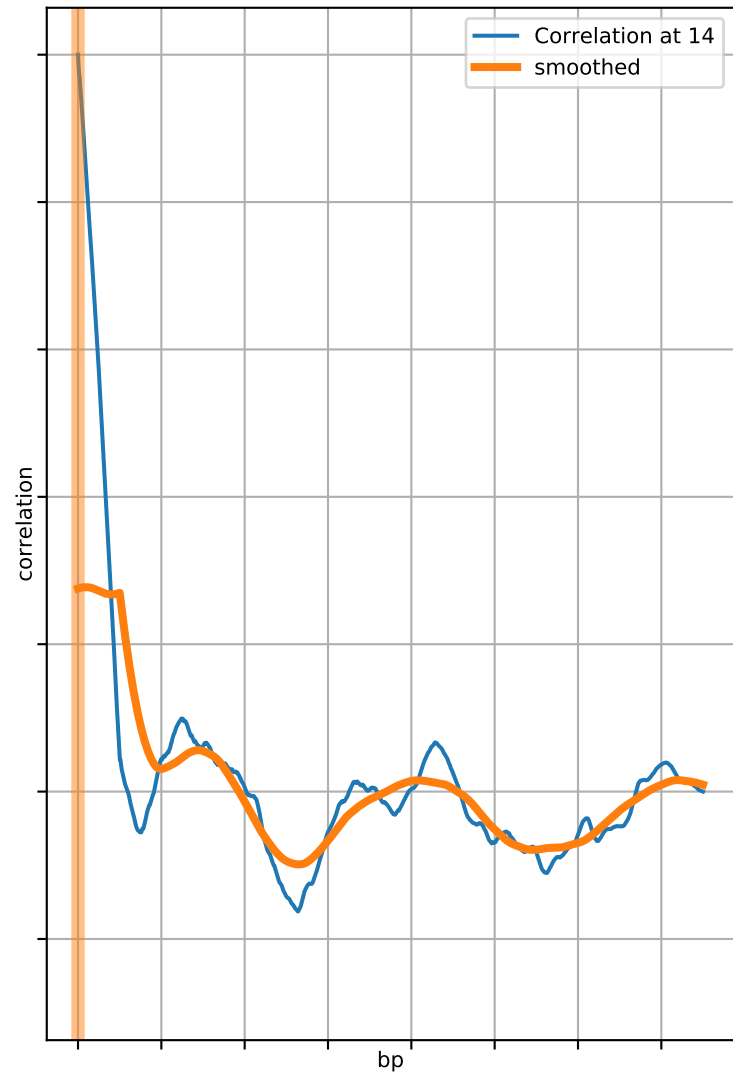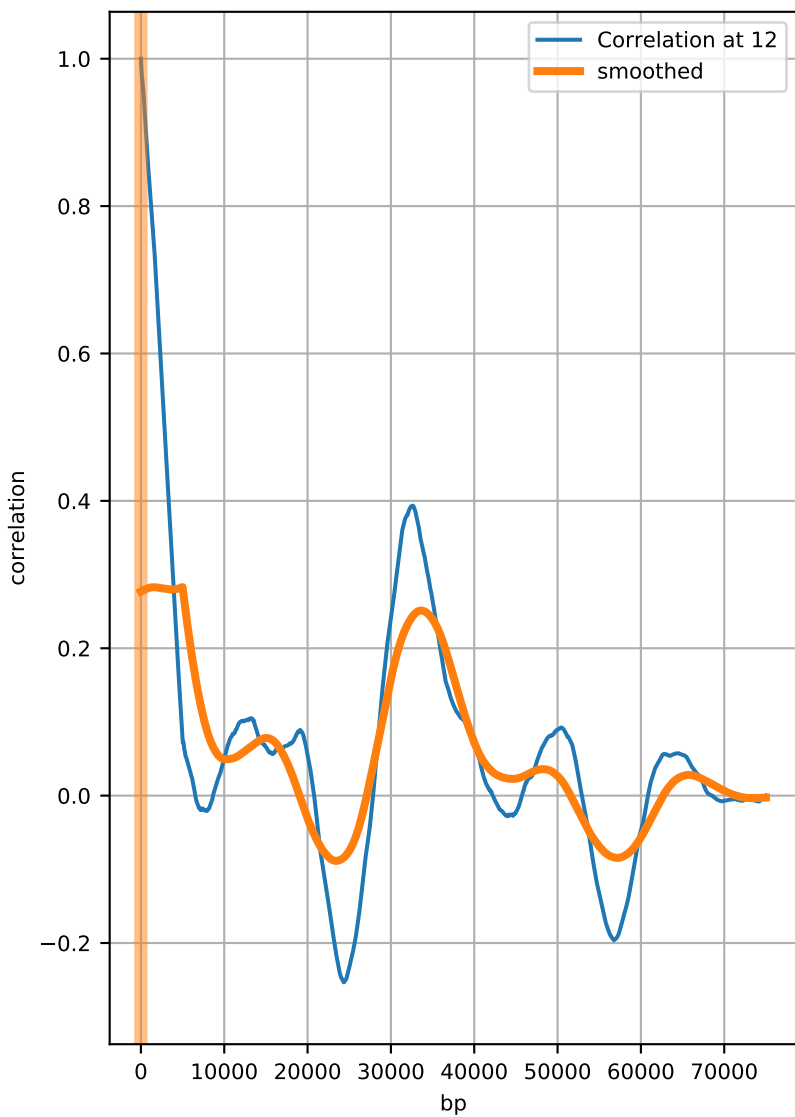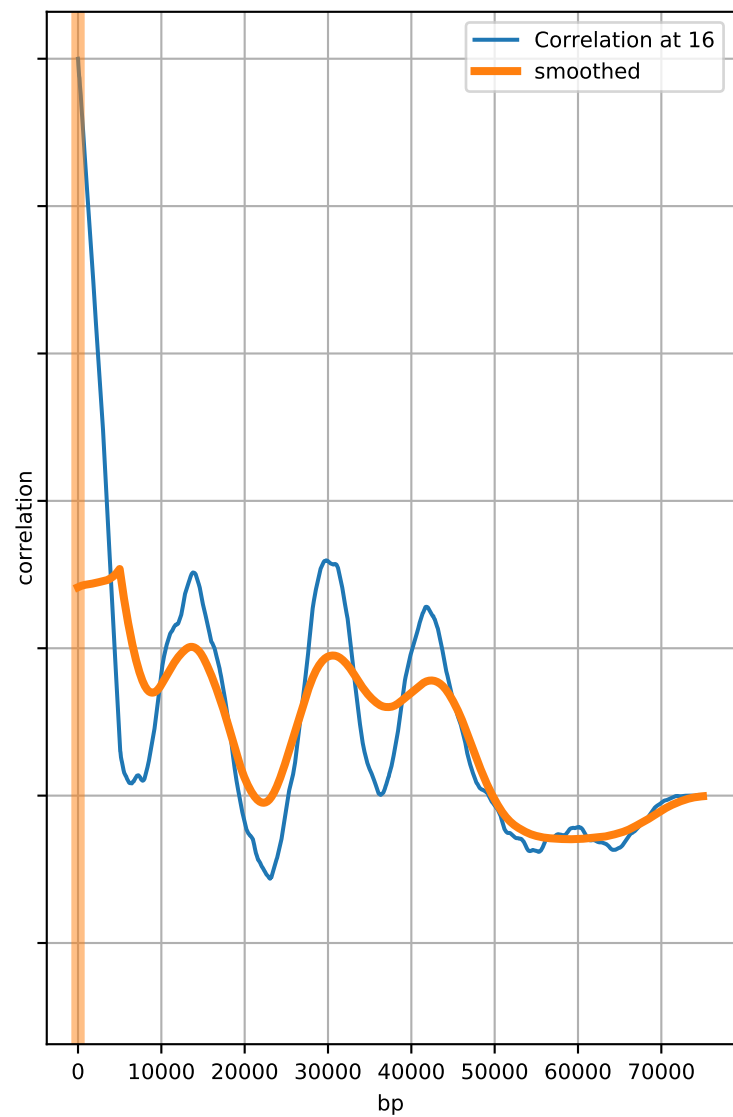

Supplement: Supplementary file 1 [file life-12-00541-s001.zip › life-1592845-supplementary/Heermann-cluster-correlation-function-ff7f0e-chr7.pdf]

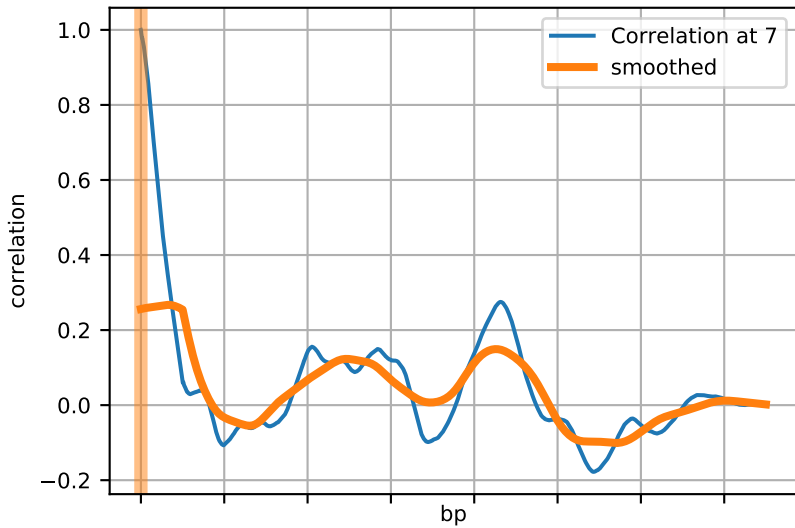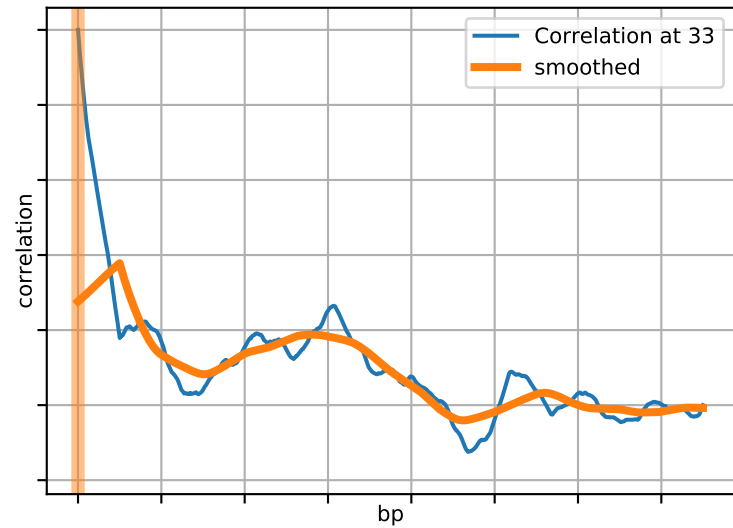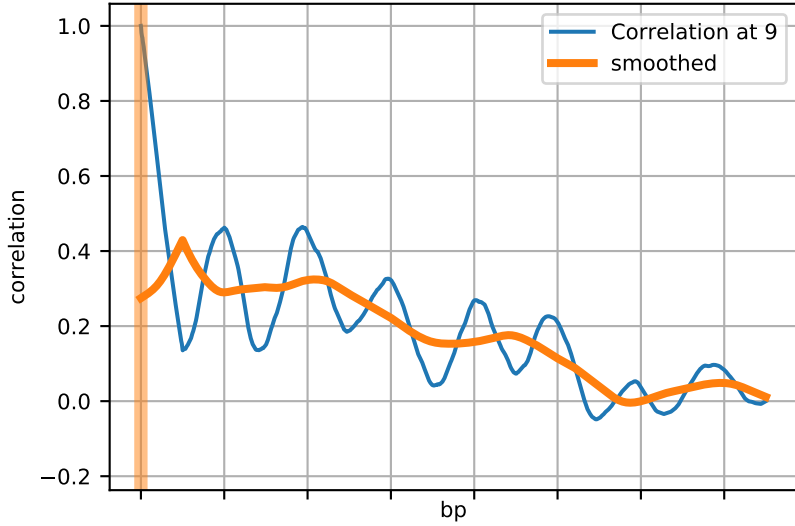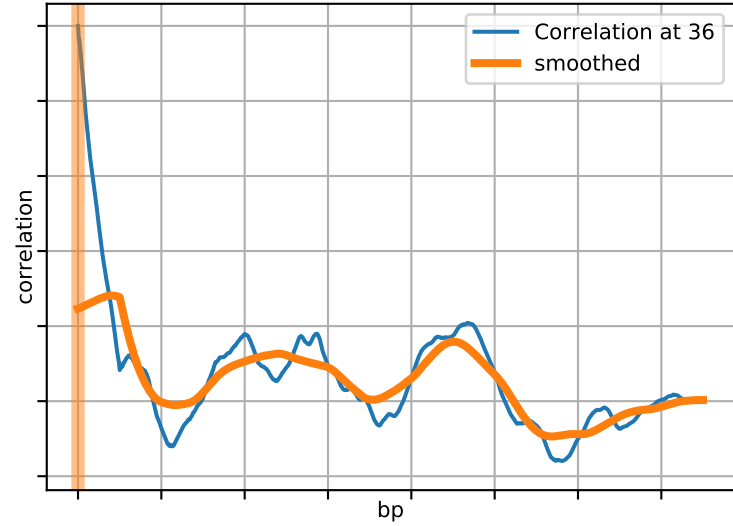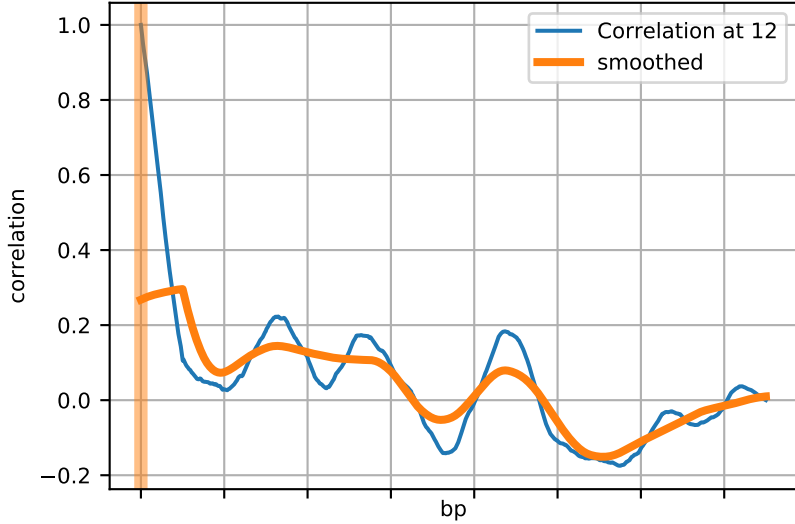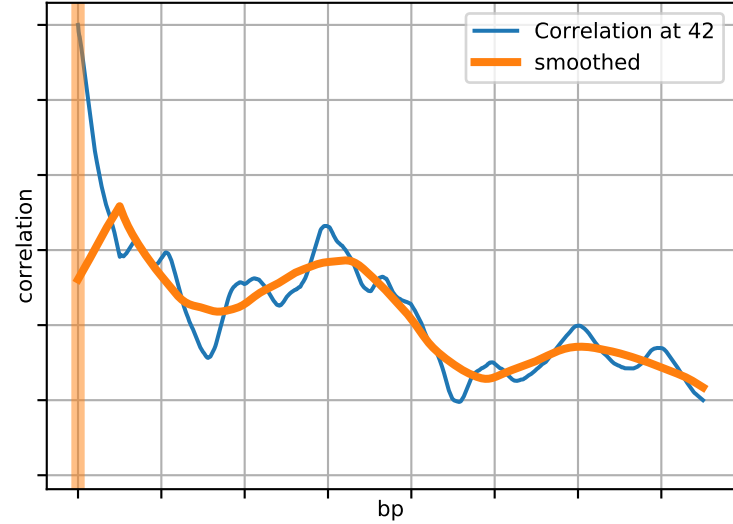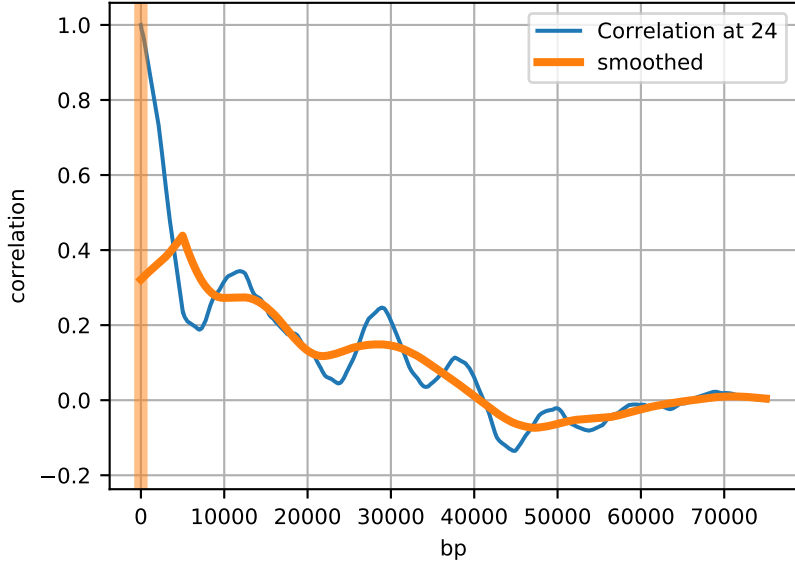

Supplement: Supplementary file 1 [file life-12-00541-s001.zip › life-1592845-supplementary/Heermann-cluster-correlation-function-ff7f0e-chrR.pdf]

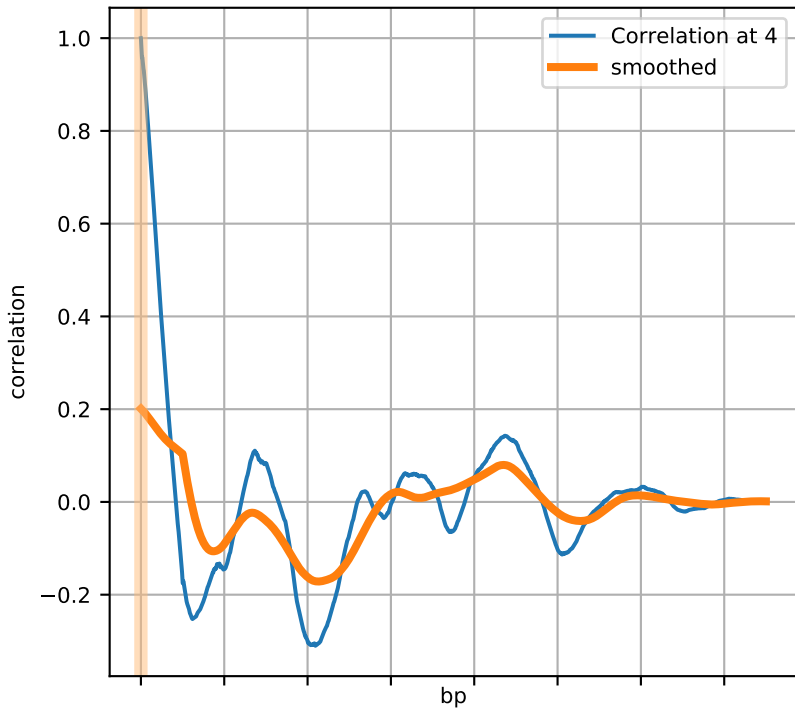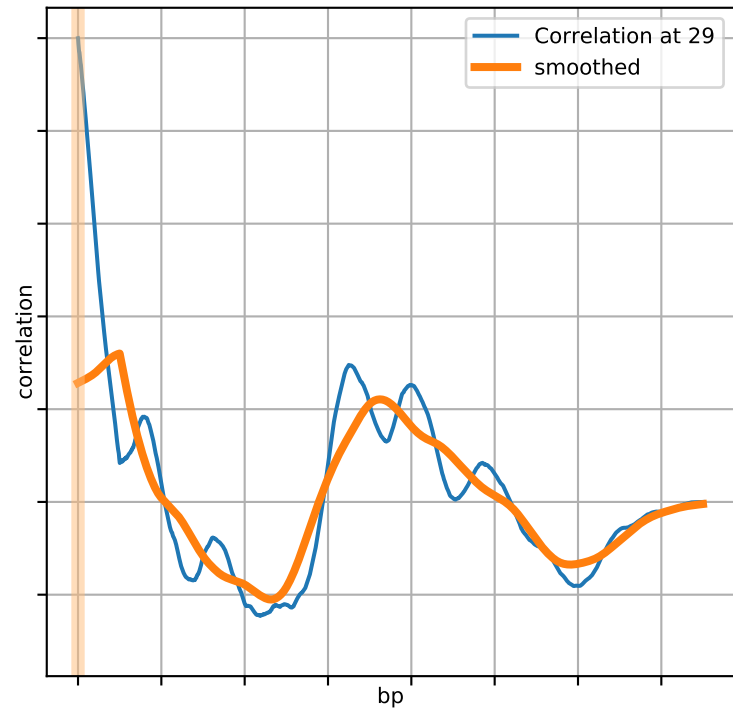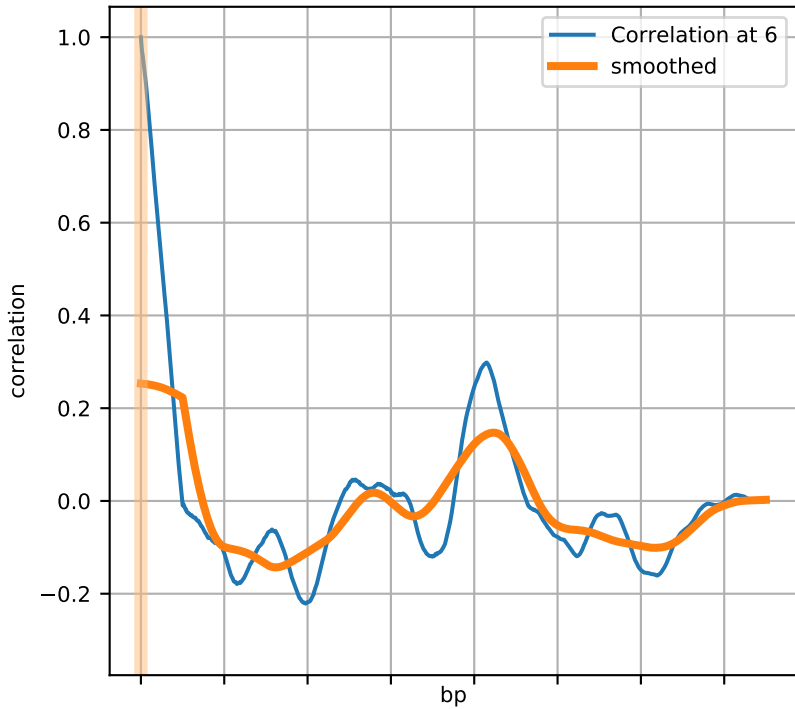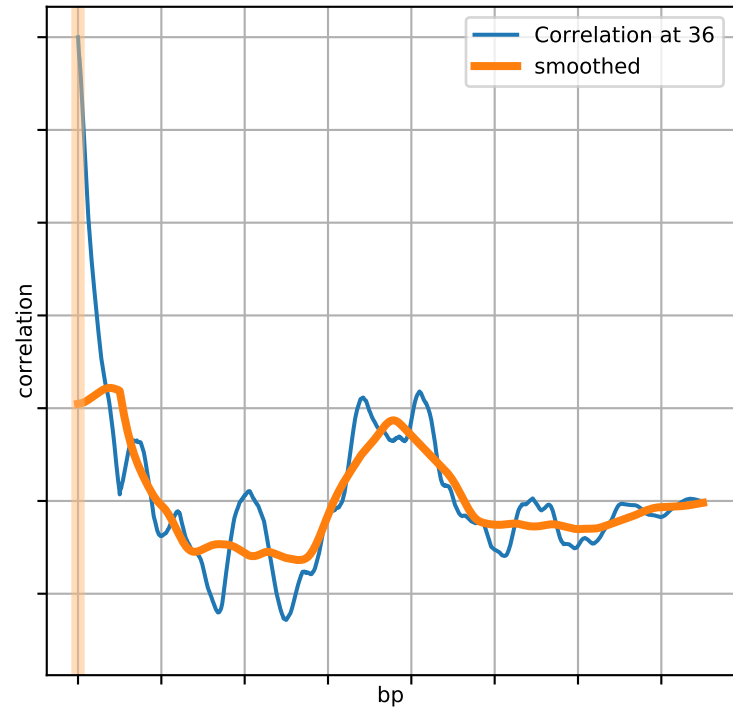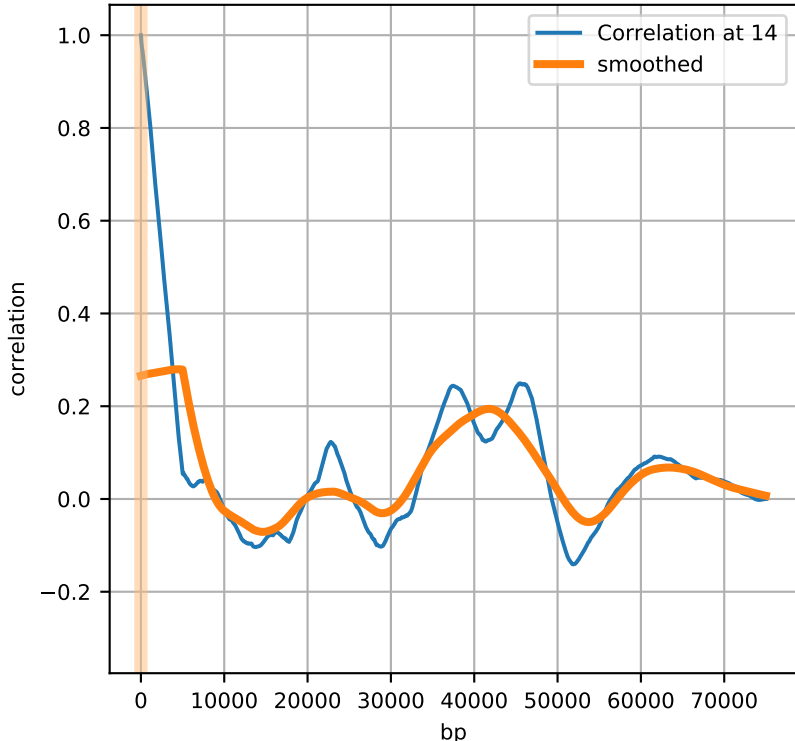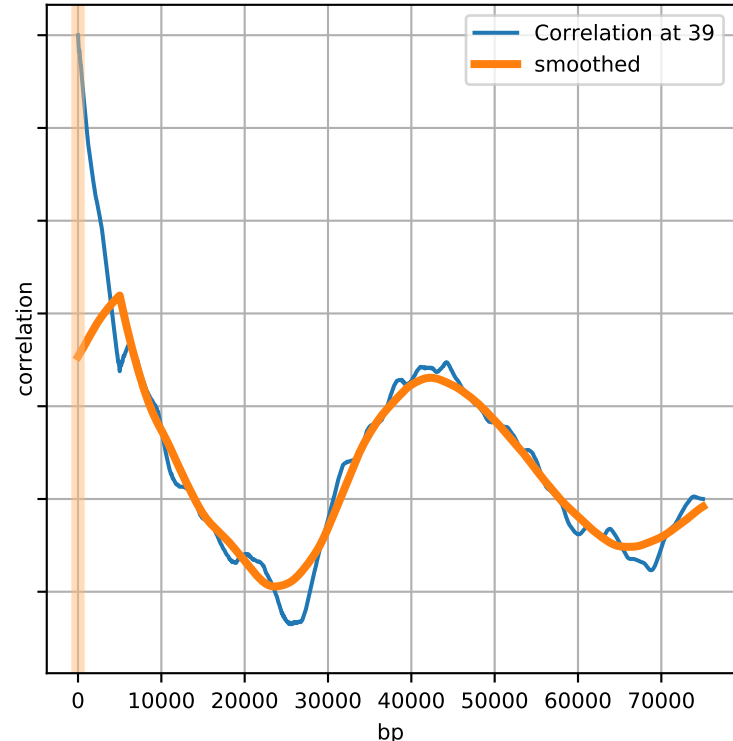

Supplement: Supplementary file 1 [file life-12-00541-s001.zip › life-1592845-supplementary/Heermann-cluster-correlation-function-ffbb78-chr2.pdf]

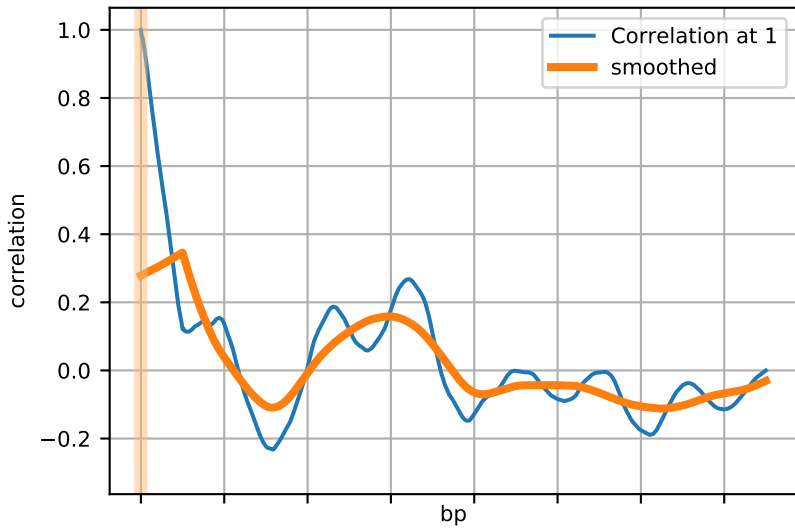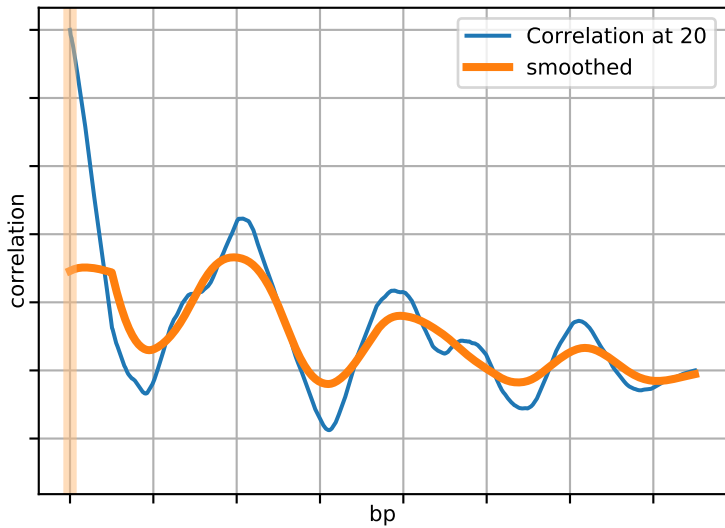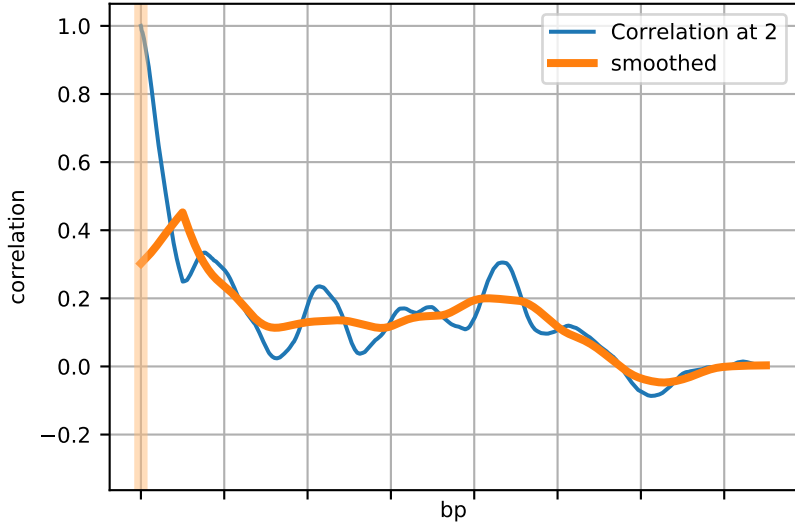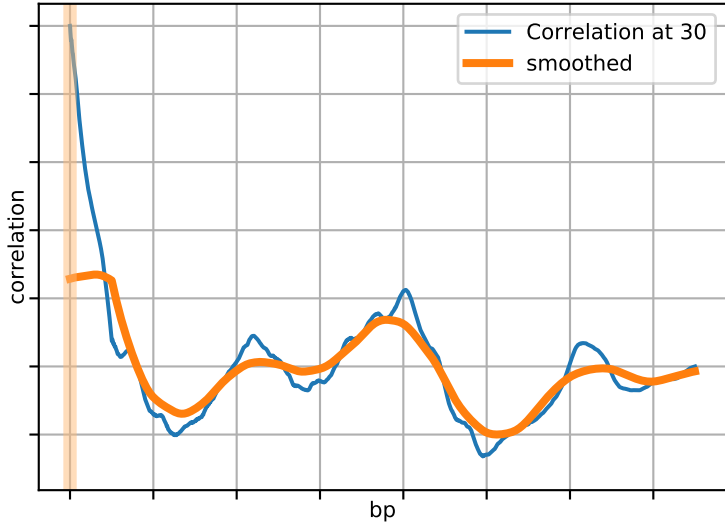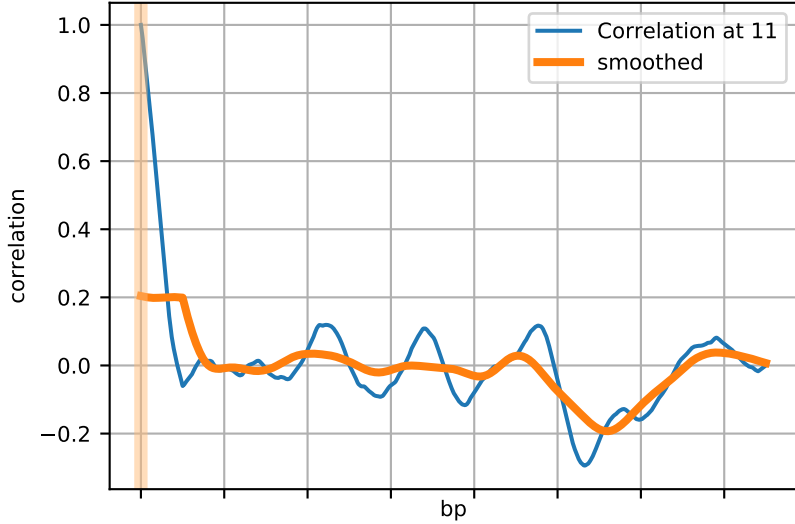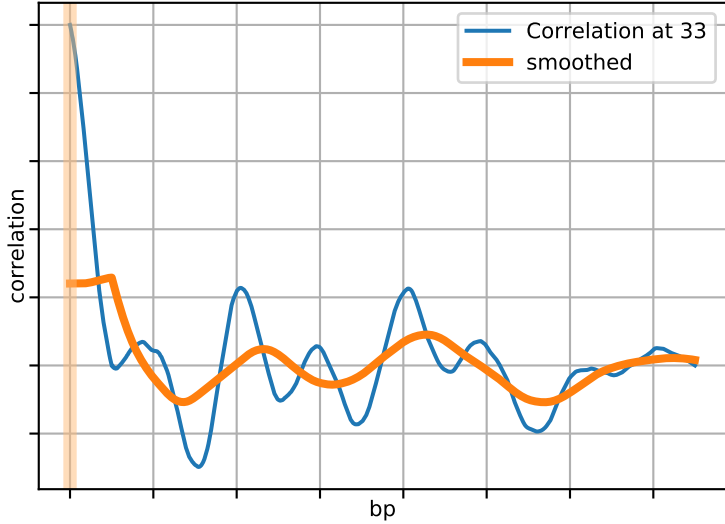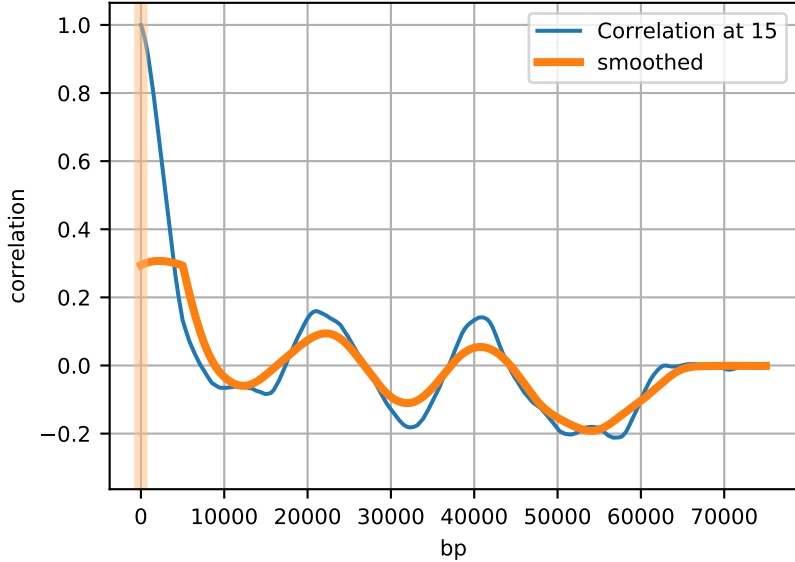

Supplement: Supplementary file 1 [file life-12-00541-s001.zip › life-1592845-supplementary/Heermann-cluster-correlation-function-ffbb78-chr3.pdf]

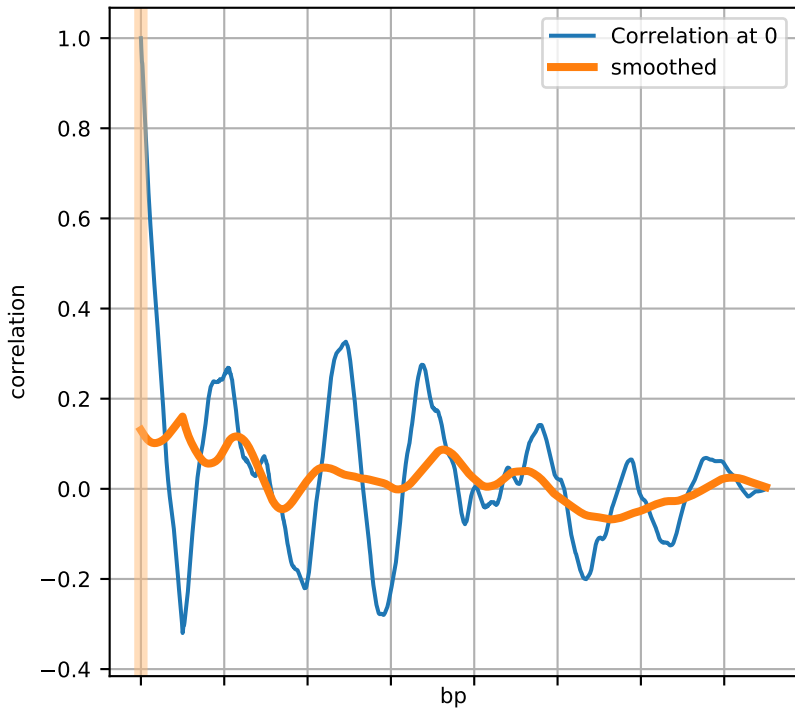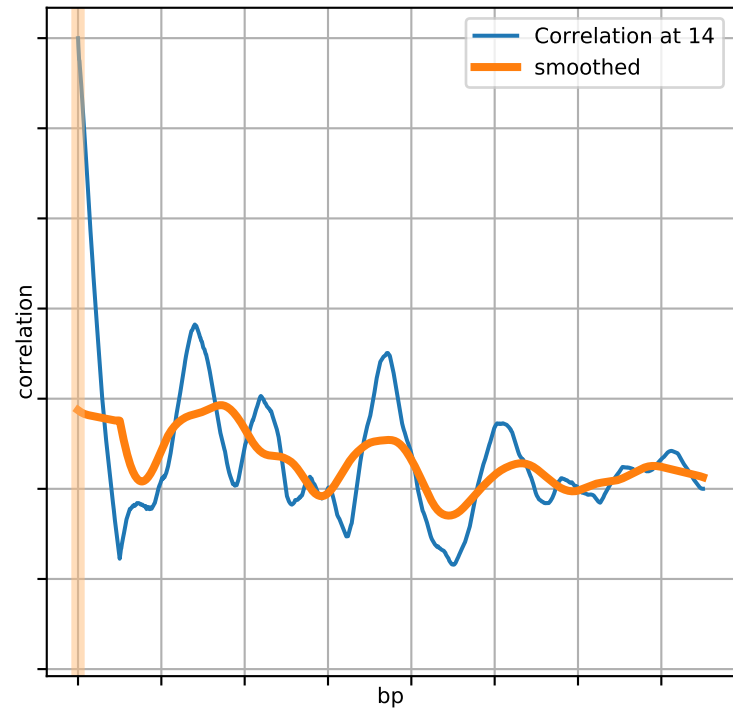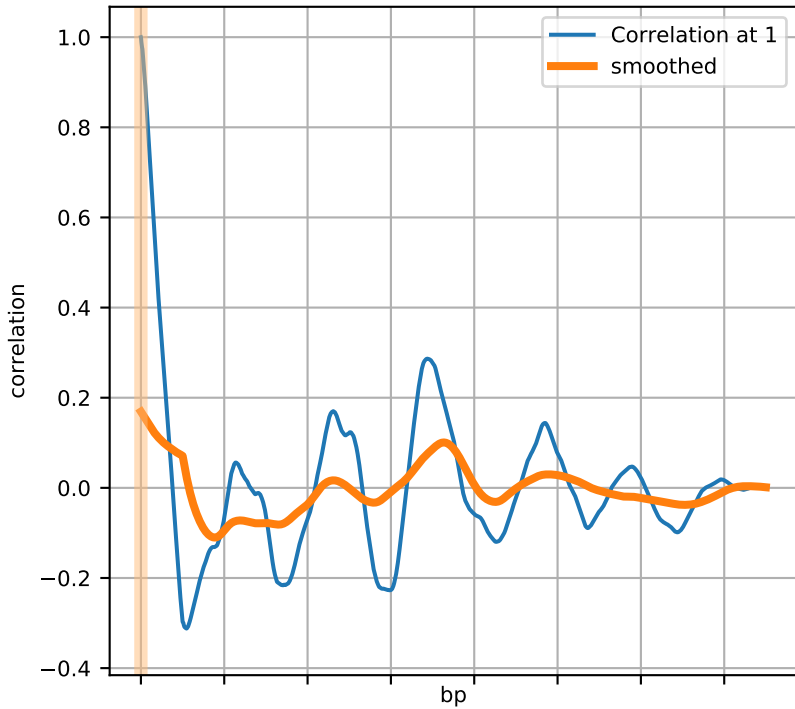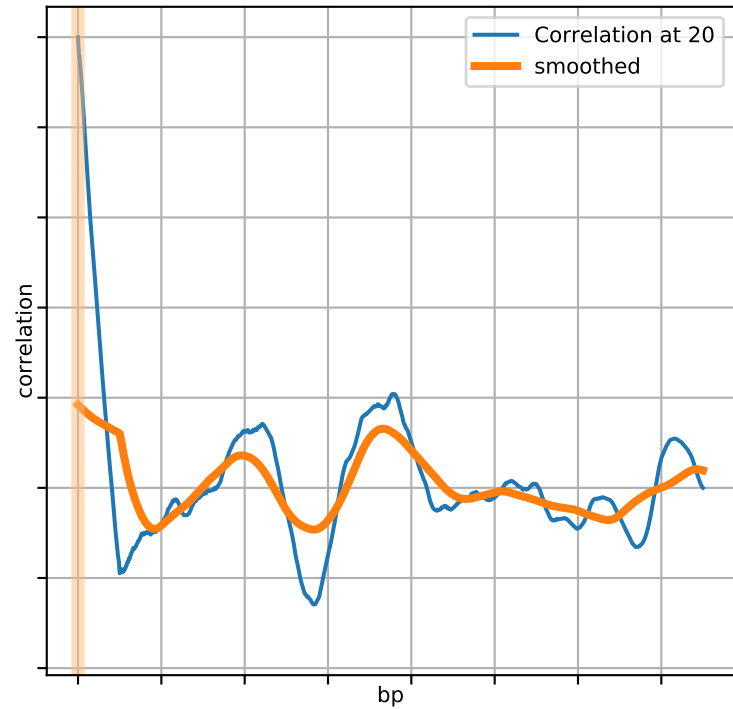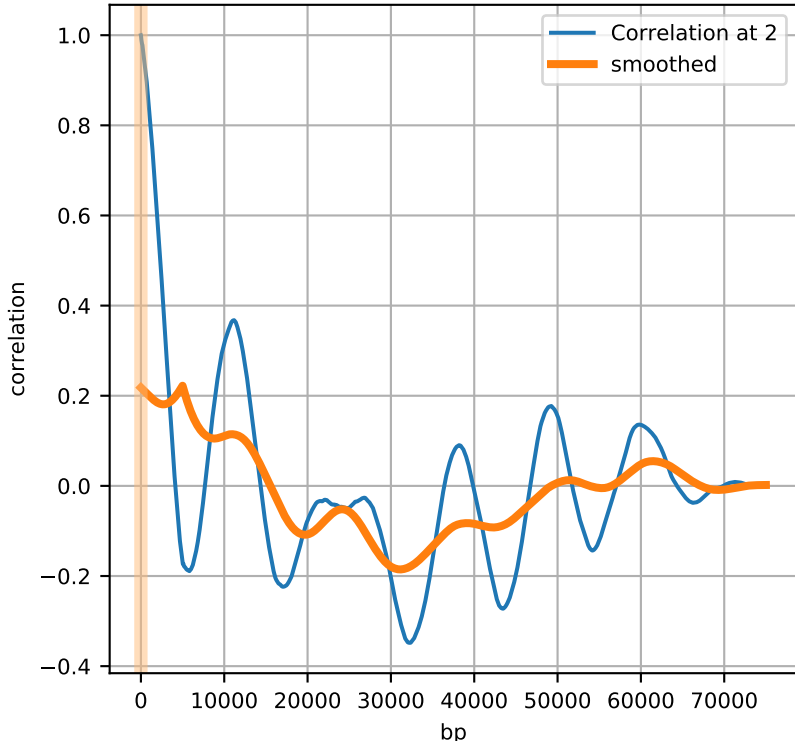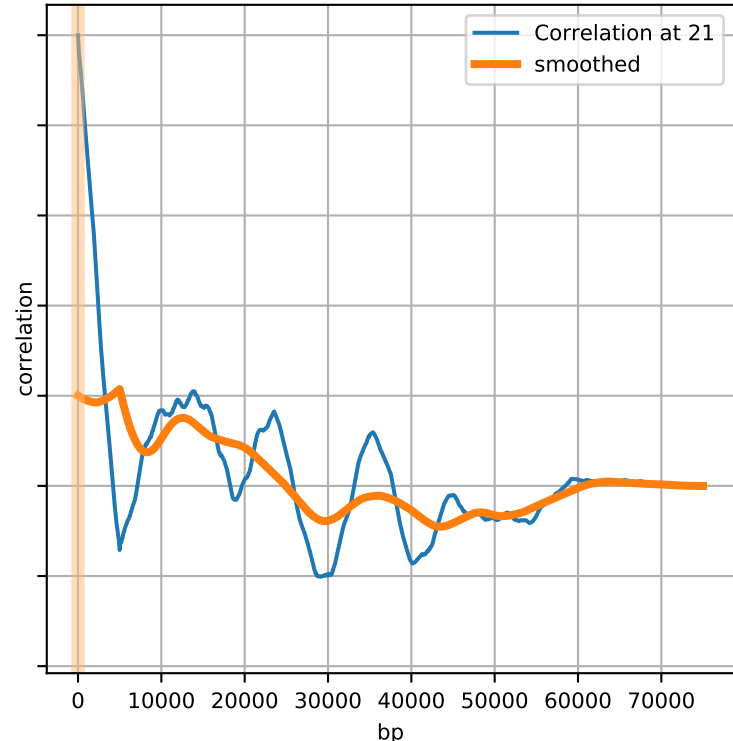

Supplement: Supplementary file 1 [file life-12-00541-s001.zip › life-1592845-supplementary/Heermann-cluster-correlation-function-ffbb78-chr4.pdf]

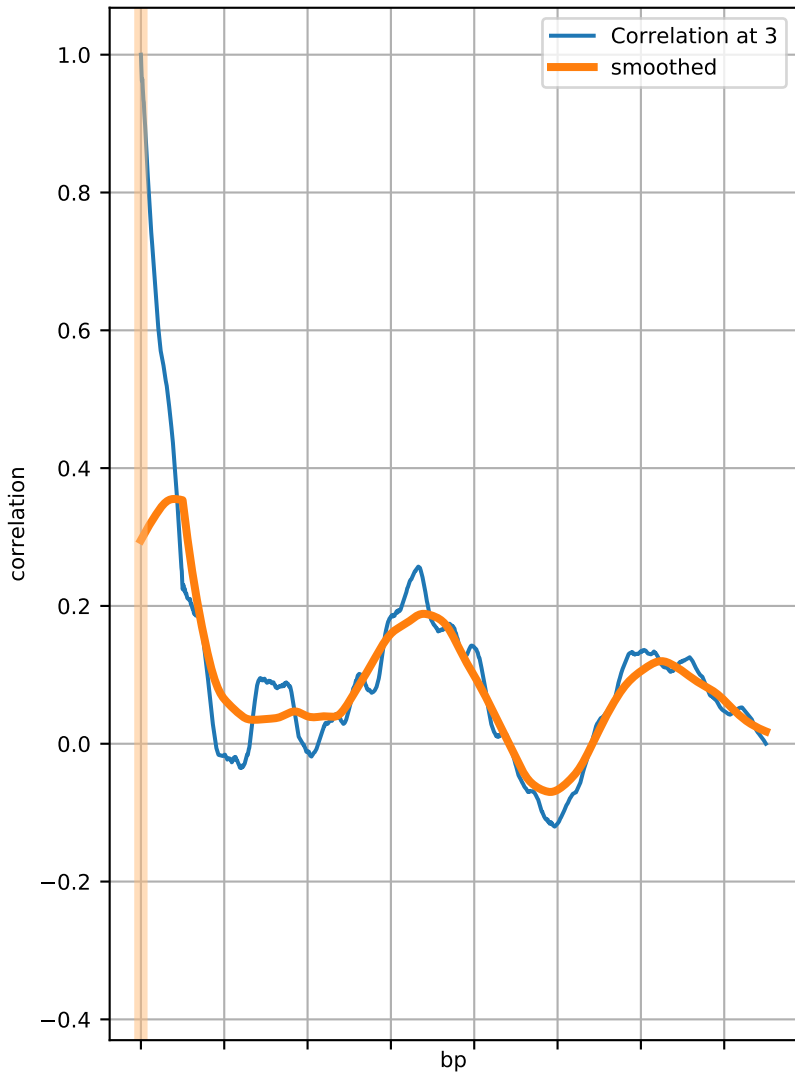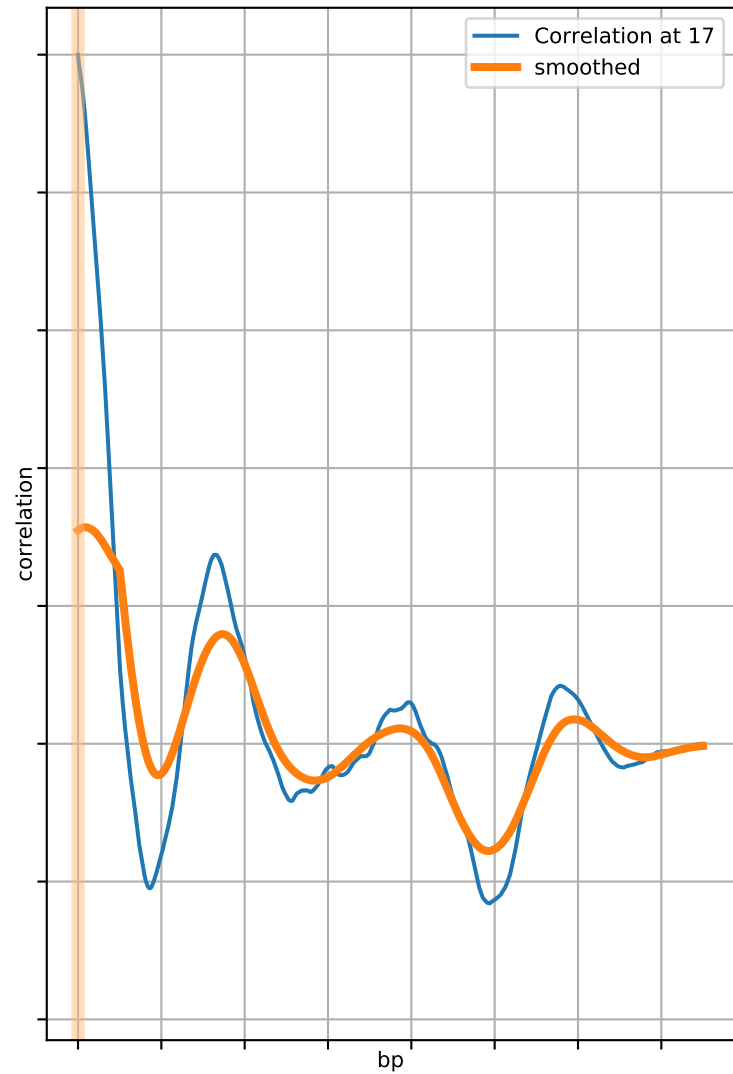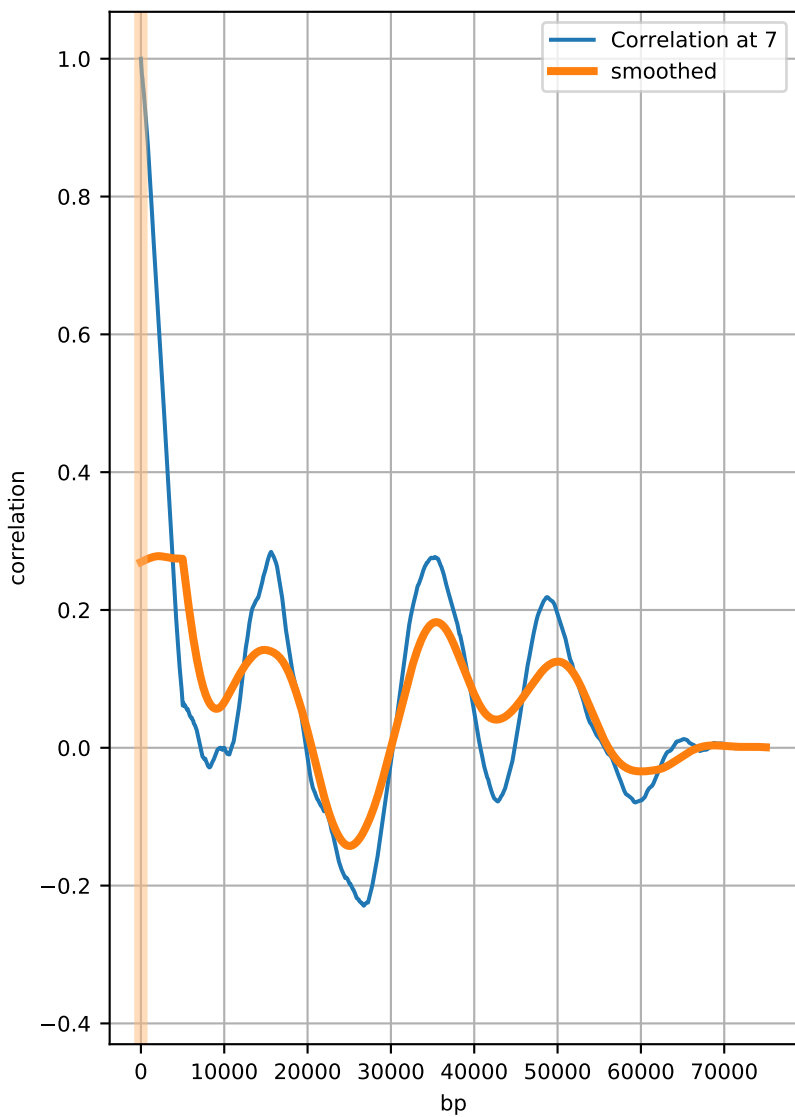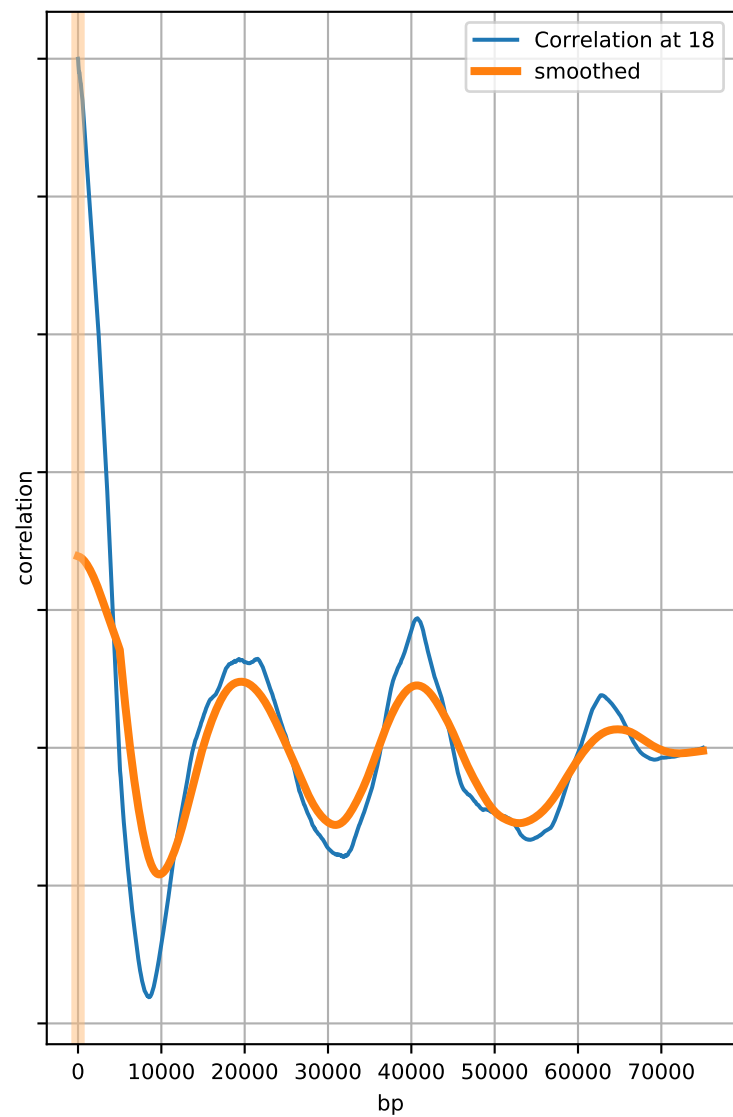

Supplement: Supplementary file 1 [file life-12-00541-s001.zip › life-1592845-supplementary/Heermann-cluster-correlation-function-ffbb78-chr5.pdf]

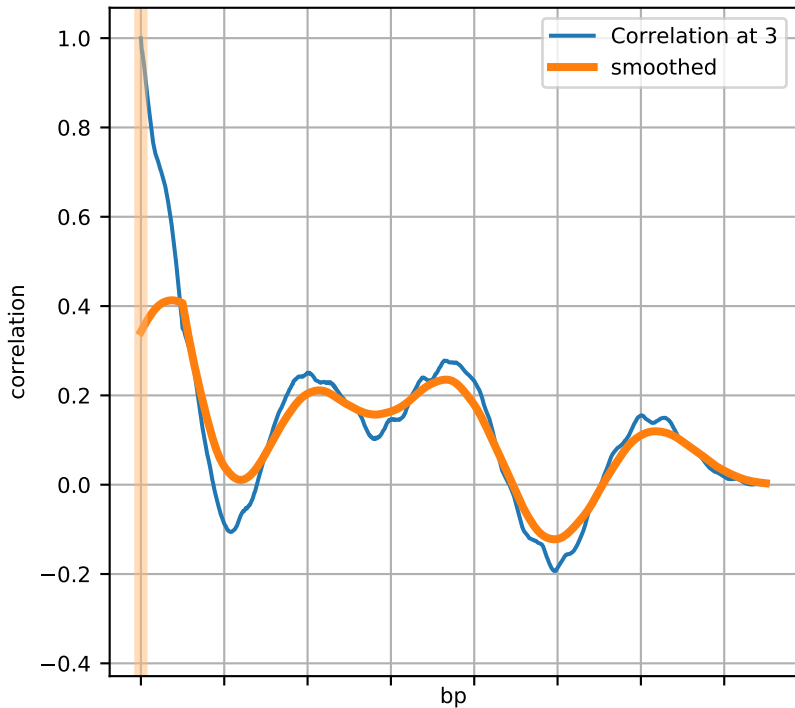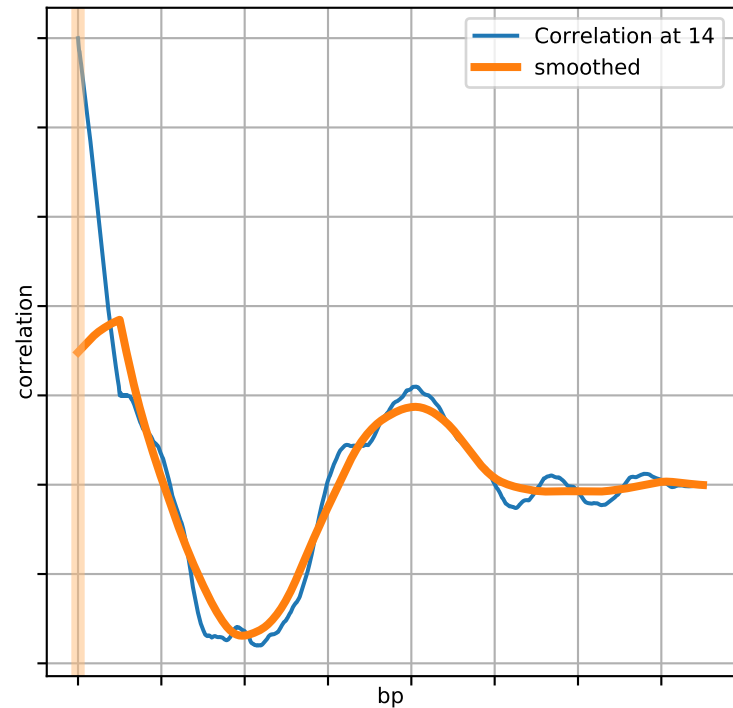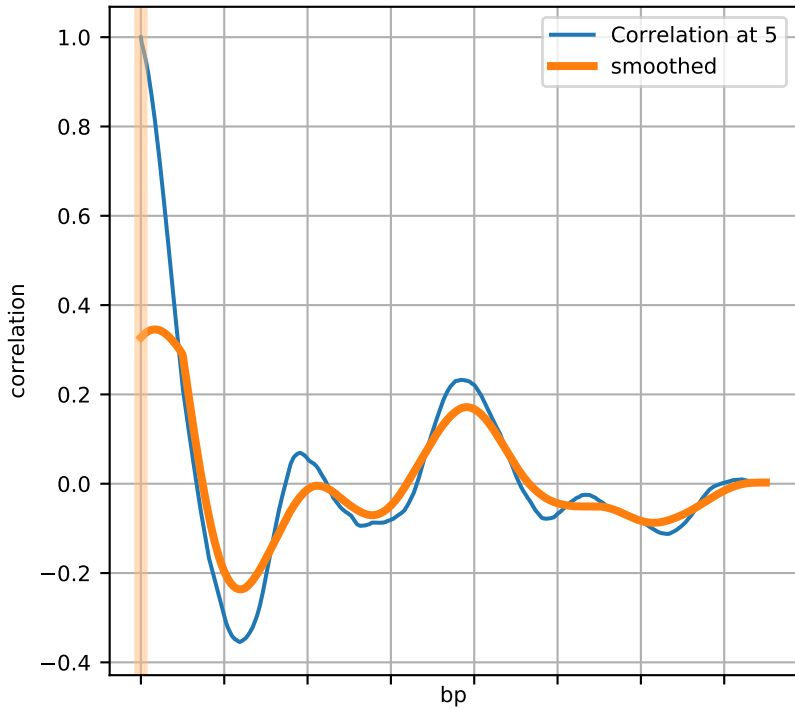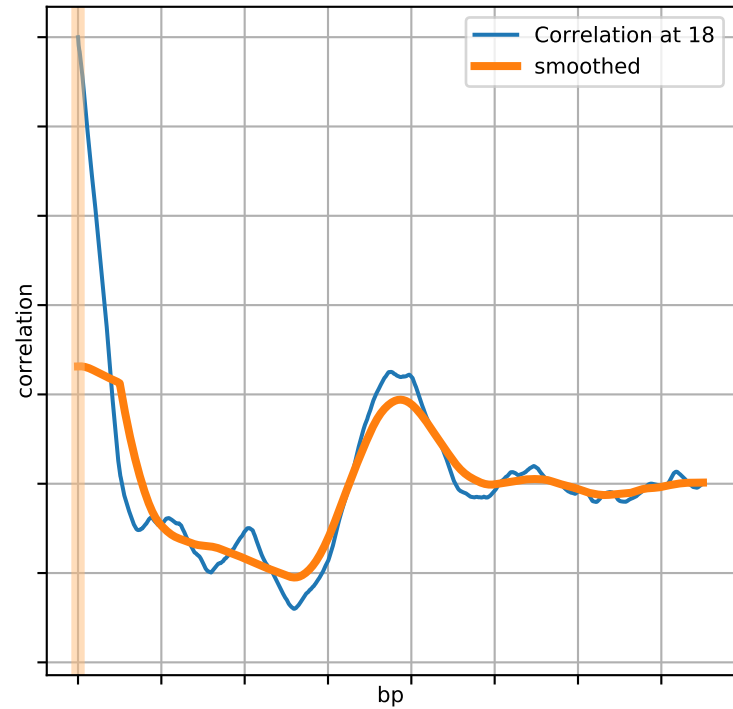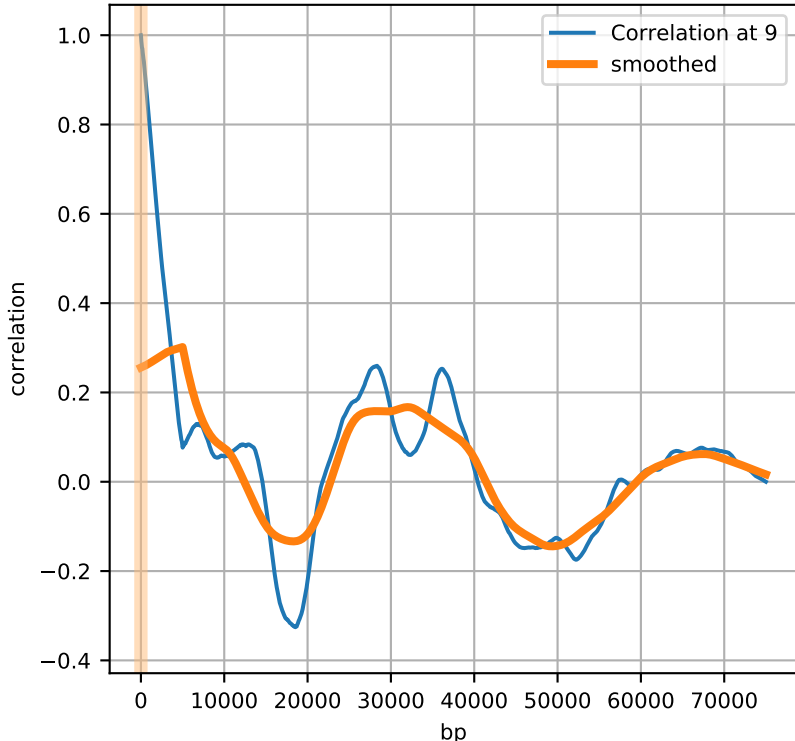

Supplement: Supplementary file 1 [file life-12-00541-s001.zip › life-1592845-supplementary/Heermann-cluster-correlation-function-ffbb78-chr6.pdf]

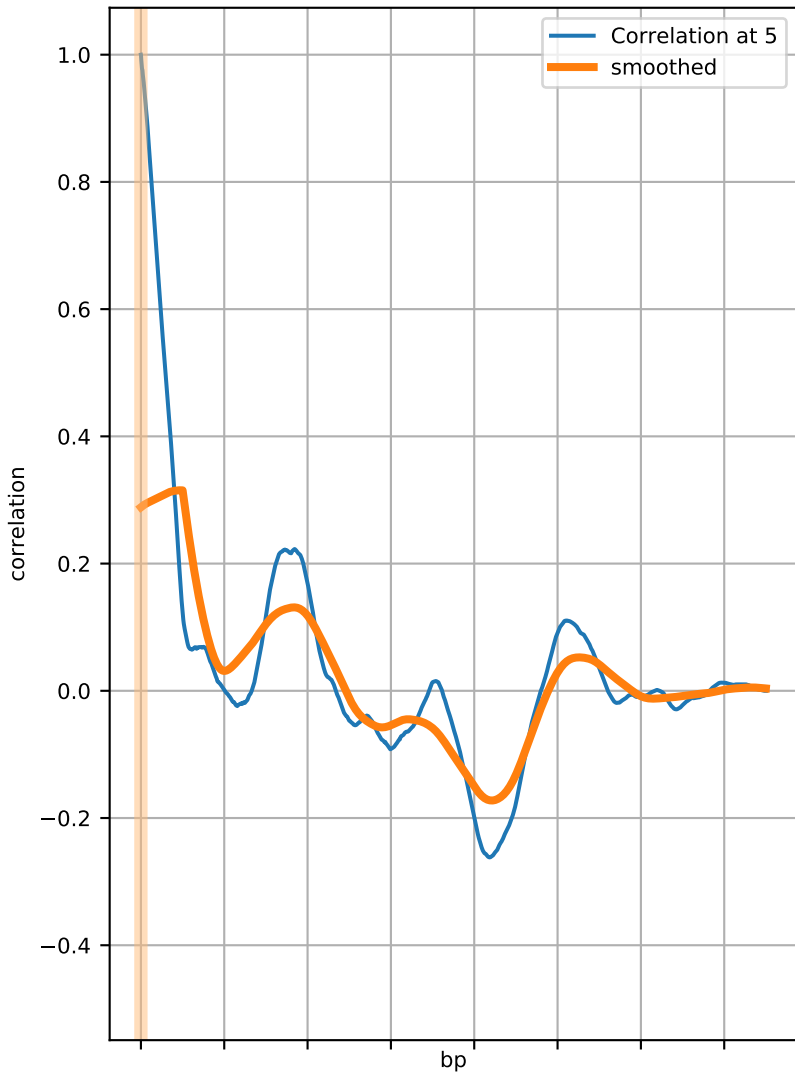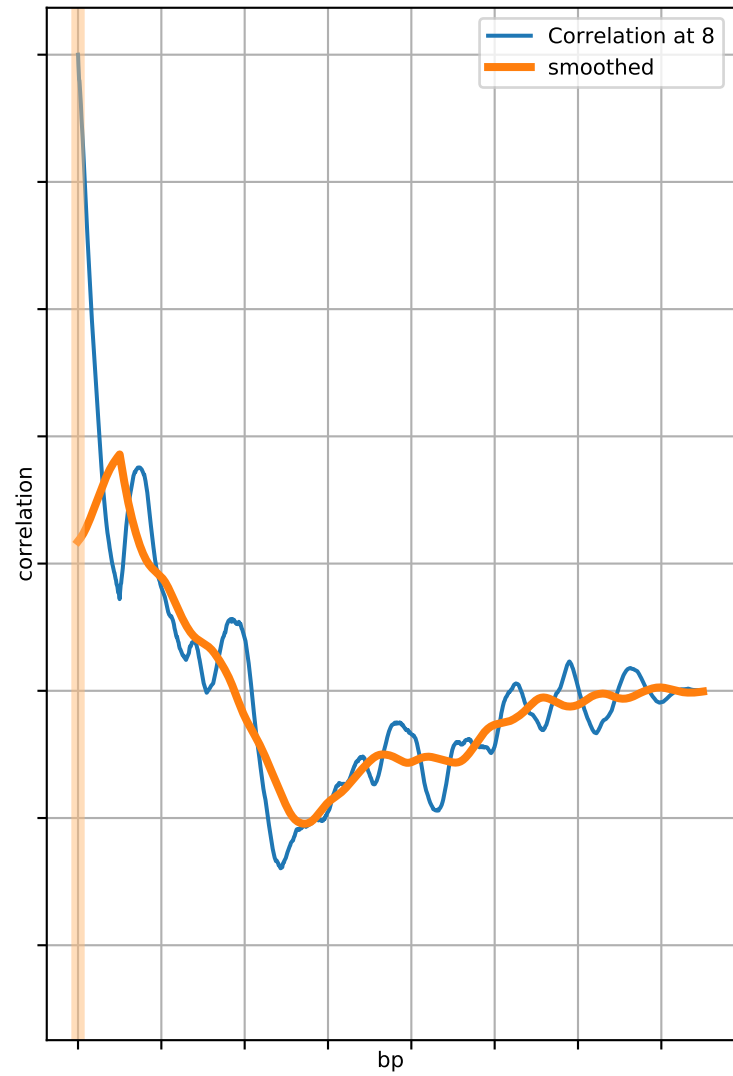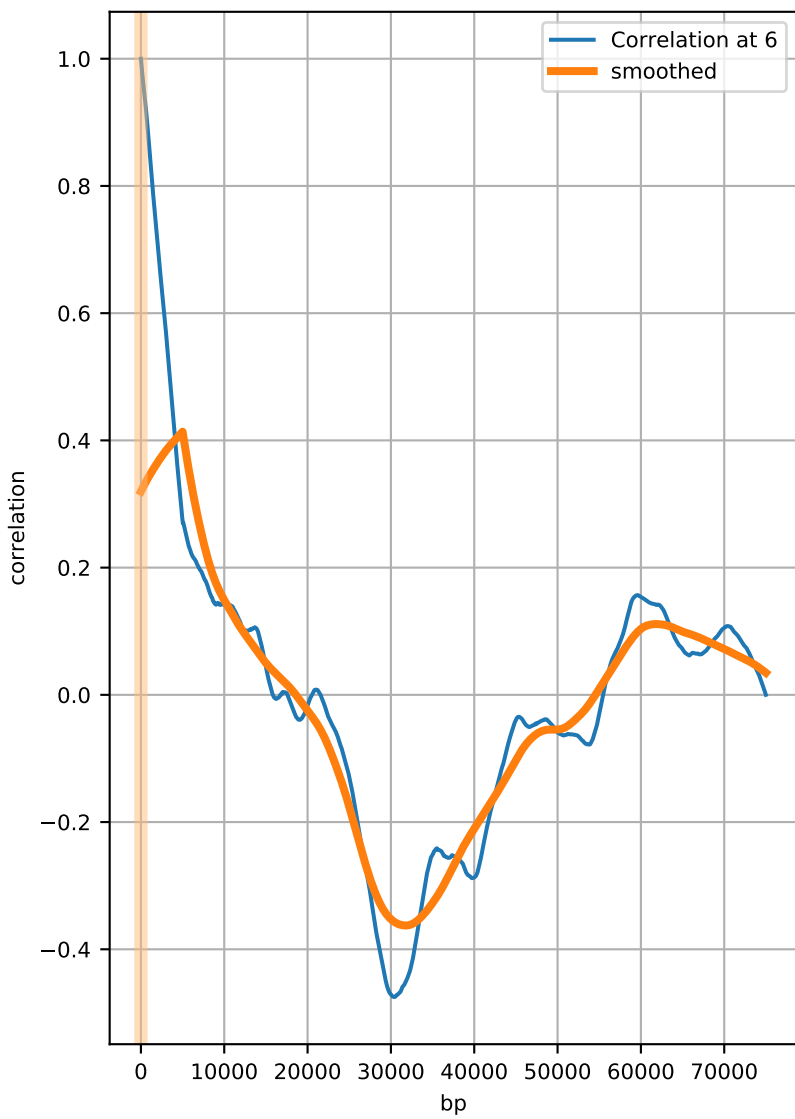

Supplement: Supplementary file 1 [file life-12-00541-s001.zip › life-1592845-supplementary/Heermann-cluster-correlation-function-ffbb78-chr7.pdf]

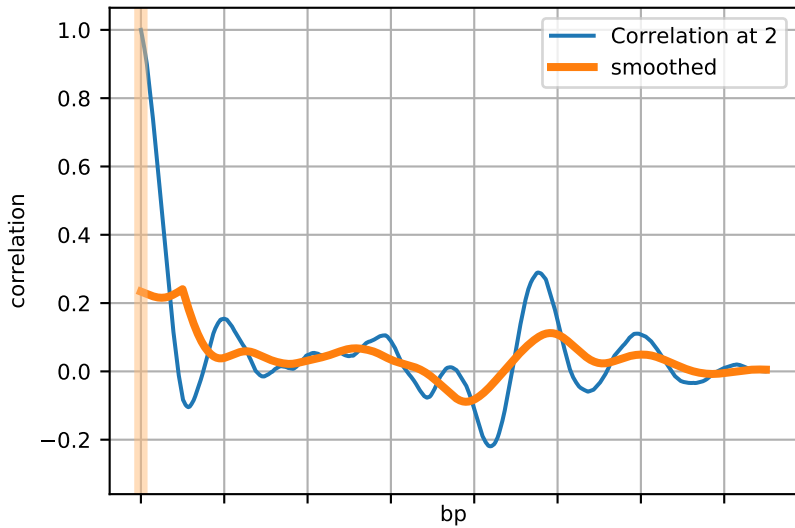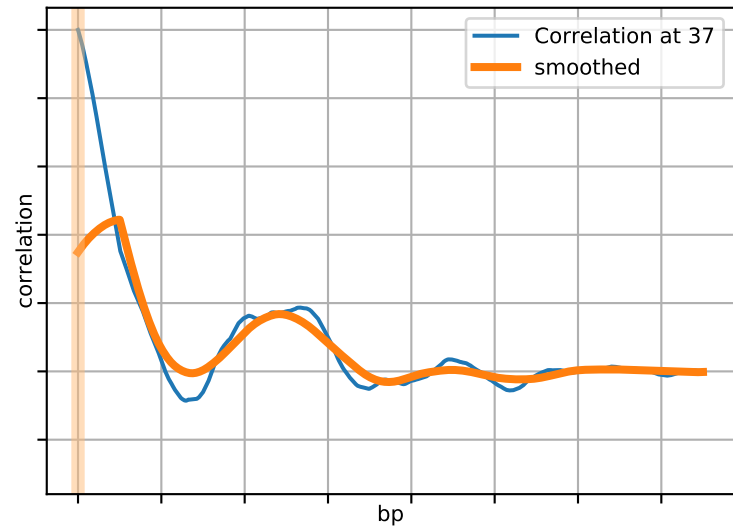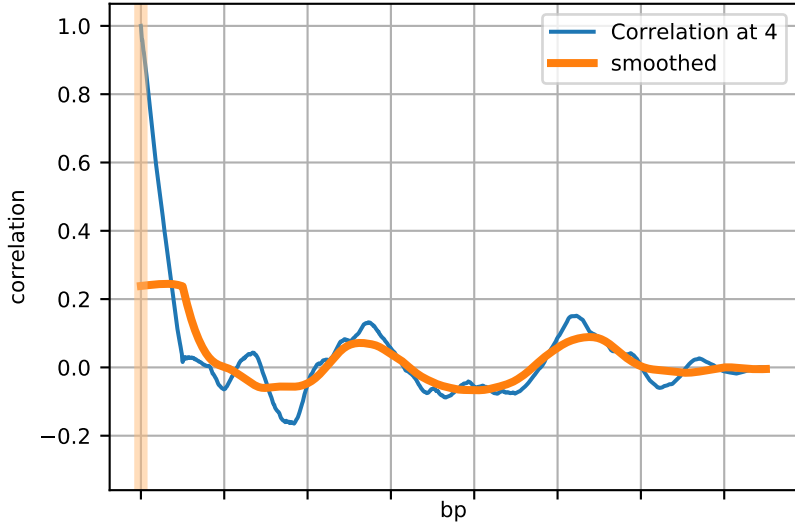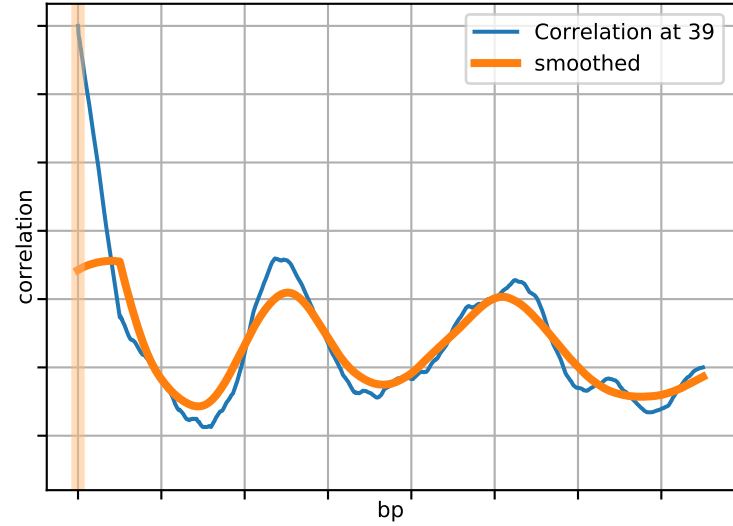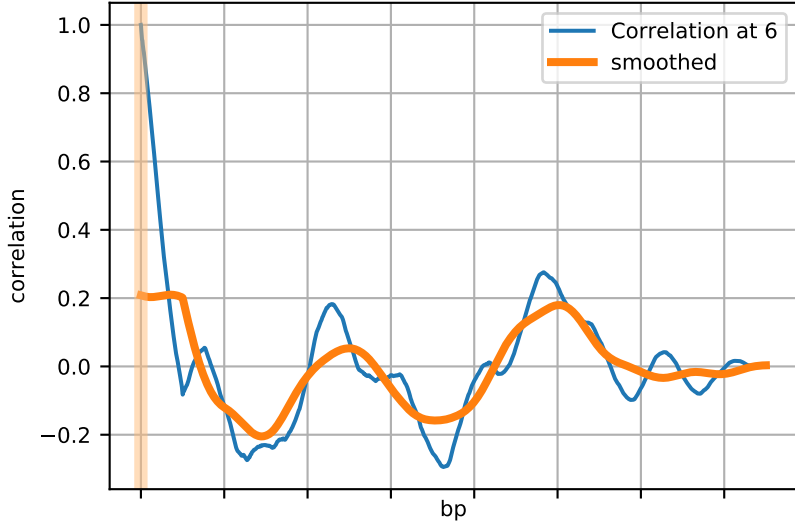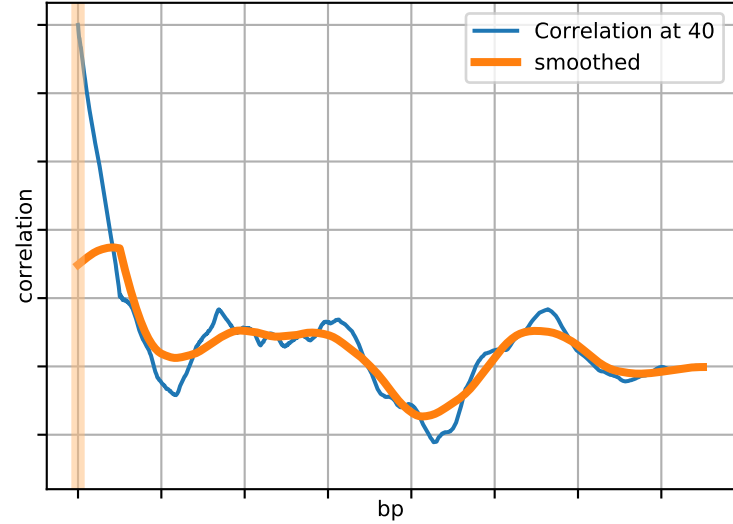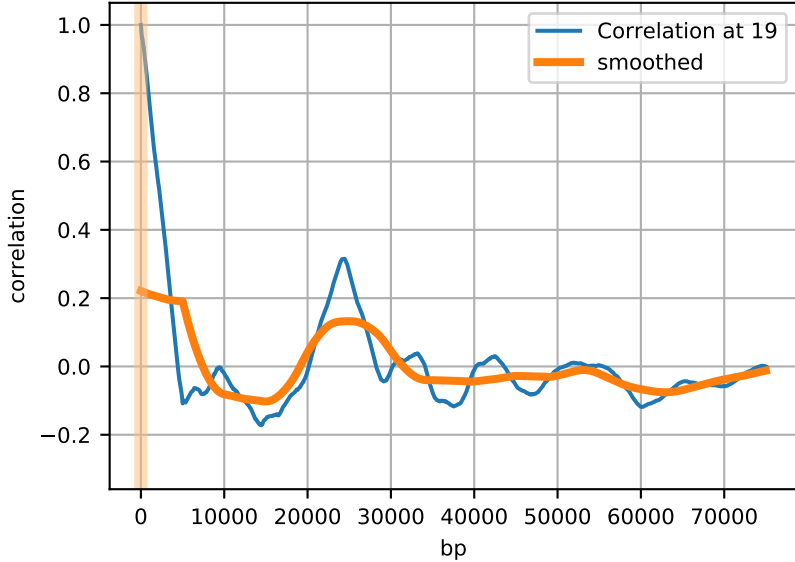

Supplement: Supplementary file 1 [file life-12-00541-s001.zip › life-1592845-supplementary/Heermann-cluster-correlation-function-ffbb78-chrR.pdf]

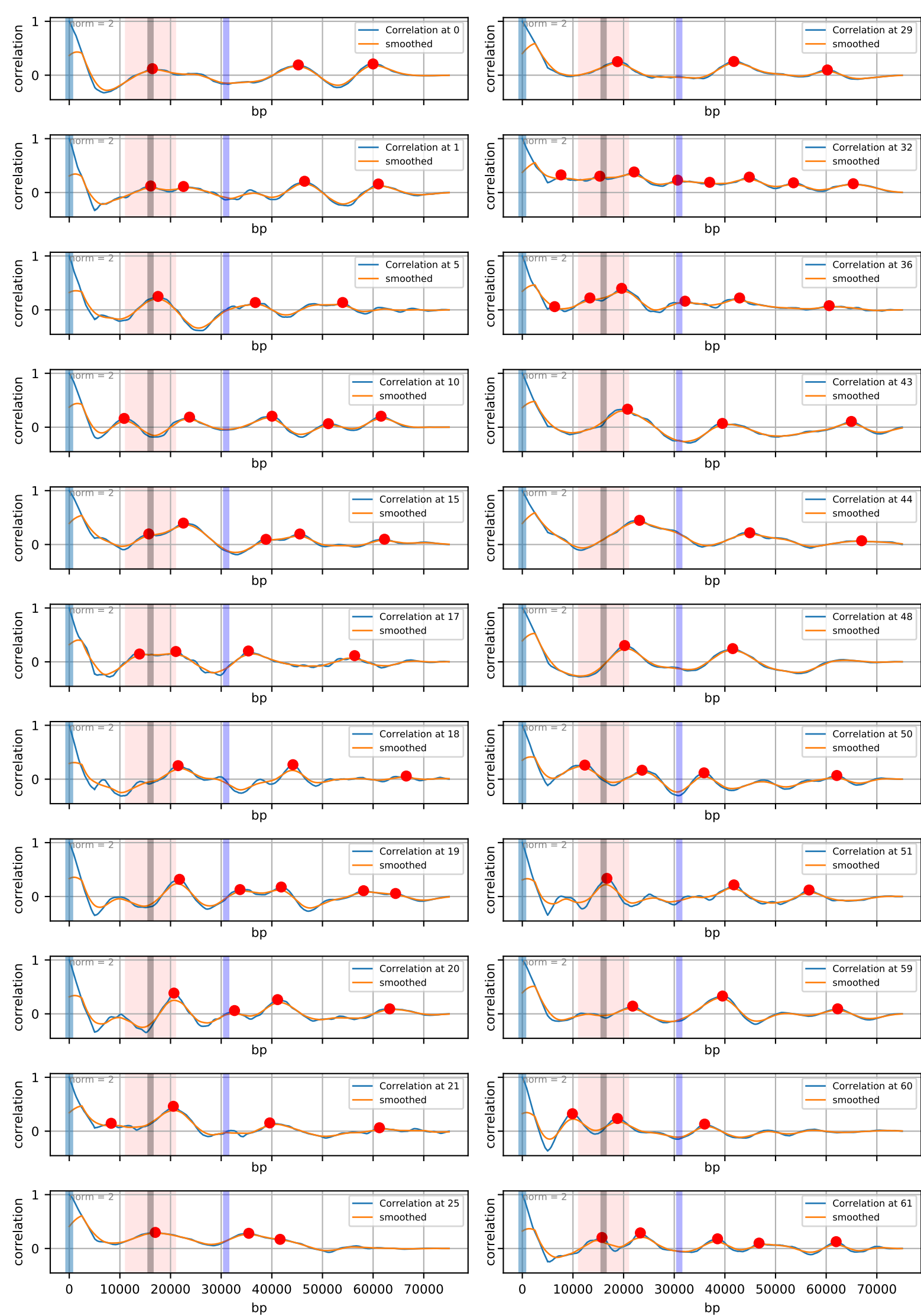

Supplement: Supplementary file 1 [file life-12-00541-s001.zip › life-1592845-supplementary/Heermann-cluster-correlation-function-peak-1f77b4-chr1.pdf]

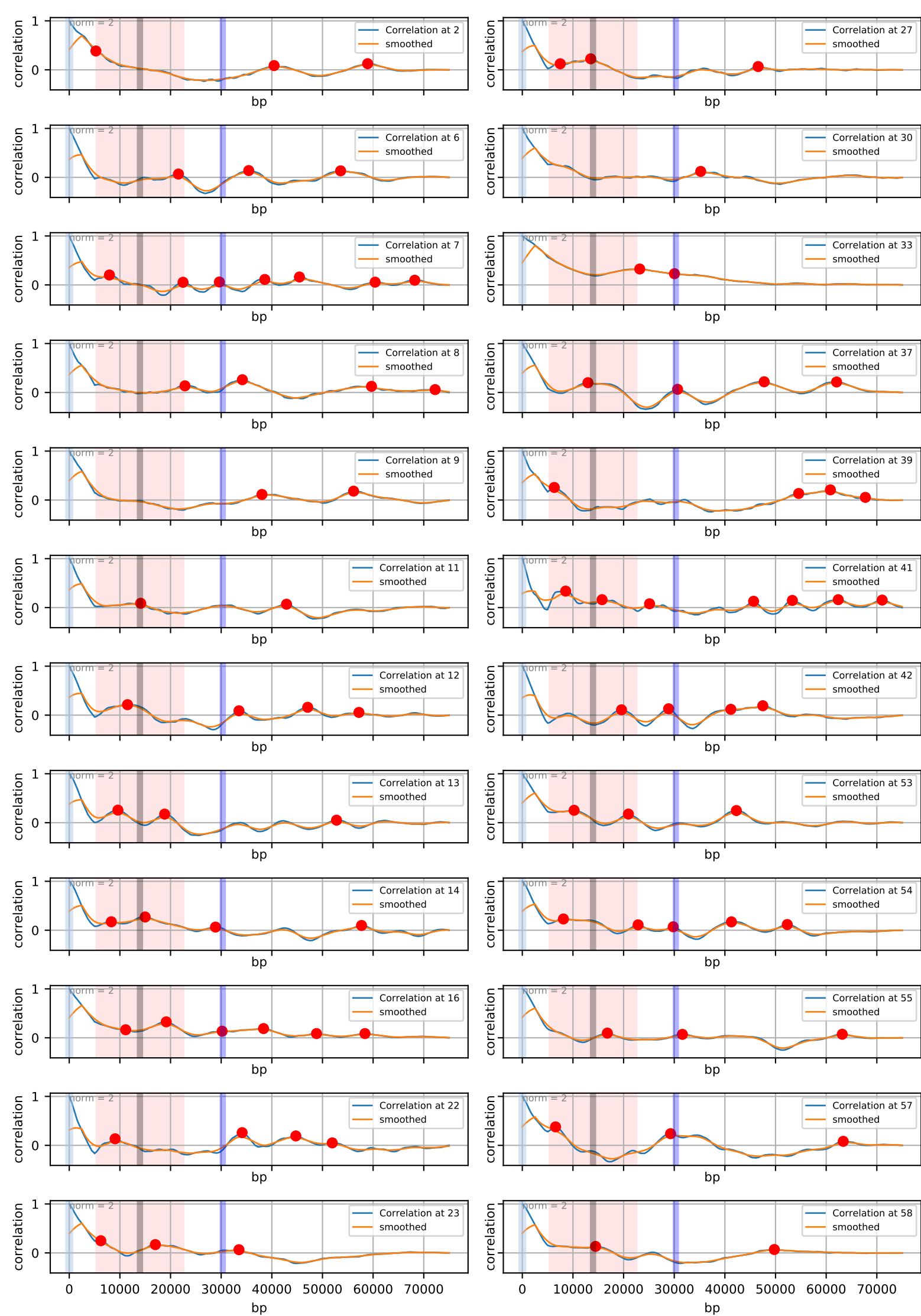

Supplement: Supplementary file 1 [file life-12-00541-s001.zip › life-1592845-supplementary/Heermann-cluster-correlation-function-peak-aec7e8-chr1.pdf]

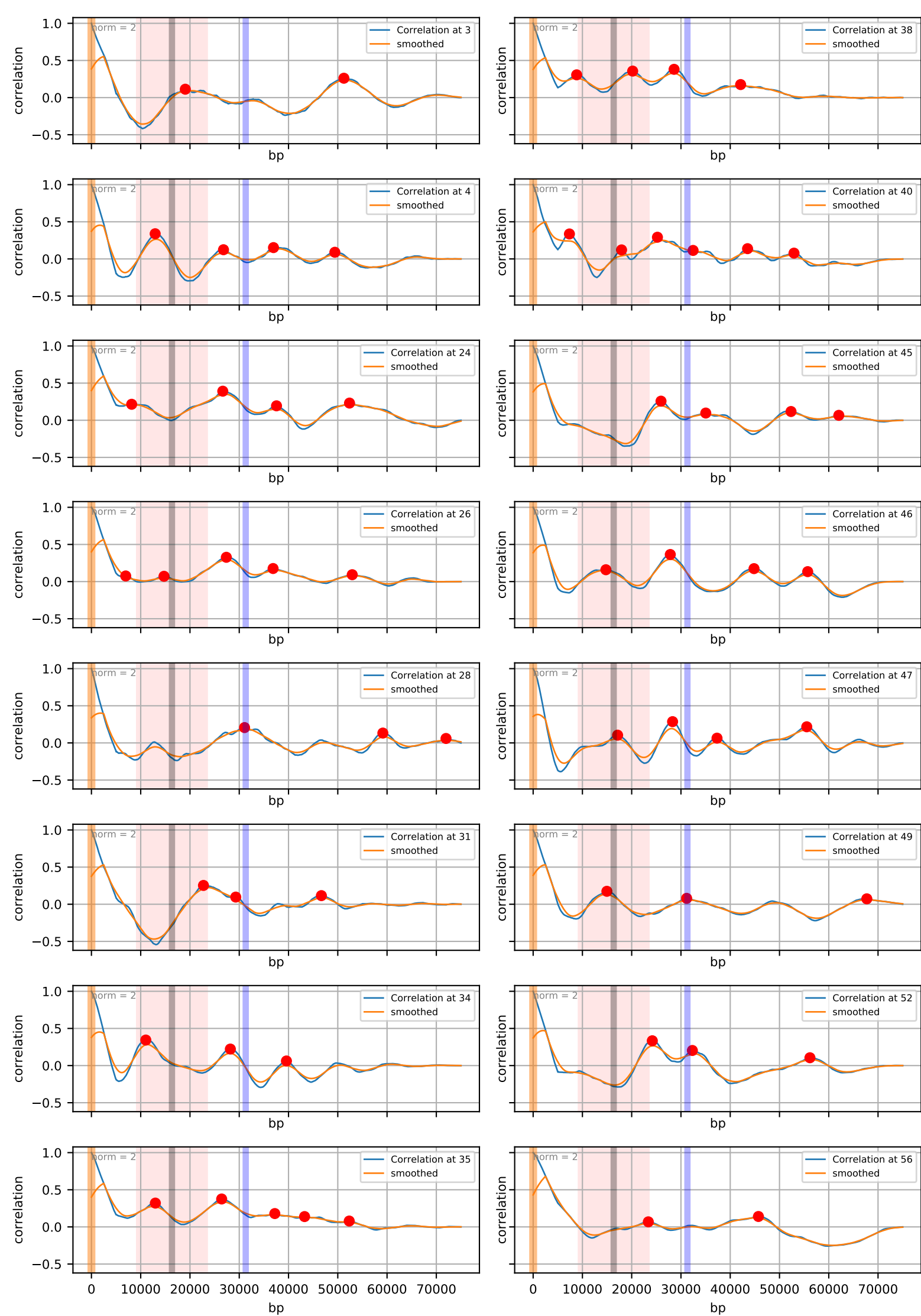

Supplement: Supplementary file 1 [file life-12-00541-s001.zip › life-1592845-supplementary/Heermann-cluster-correlation-function-peak-ff7f0e-chr1.pdf]

chr1

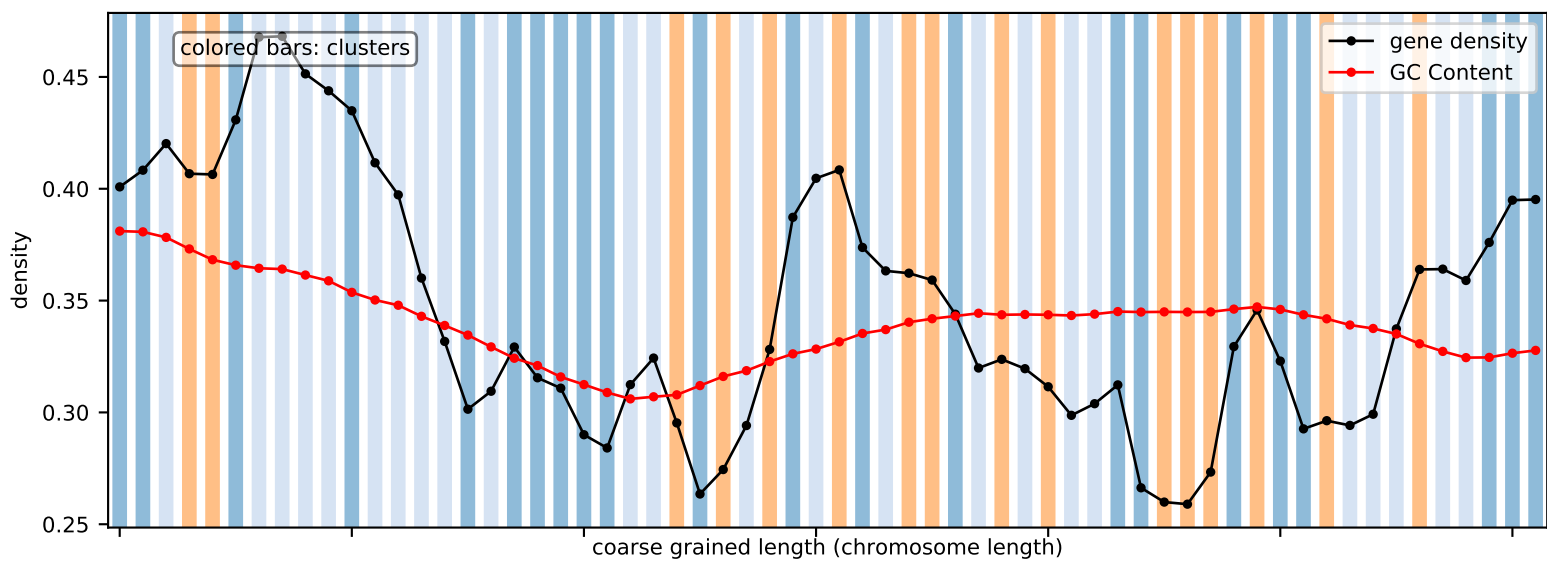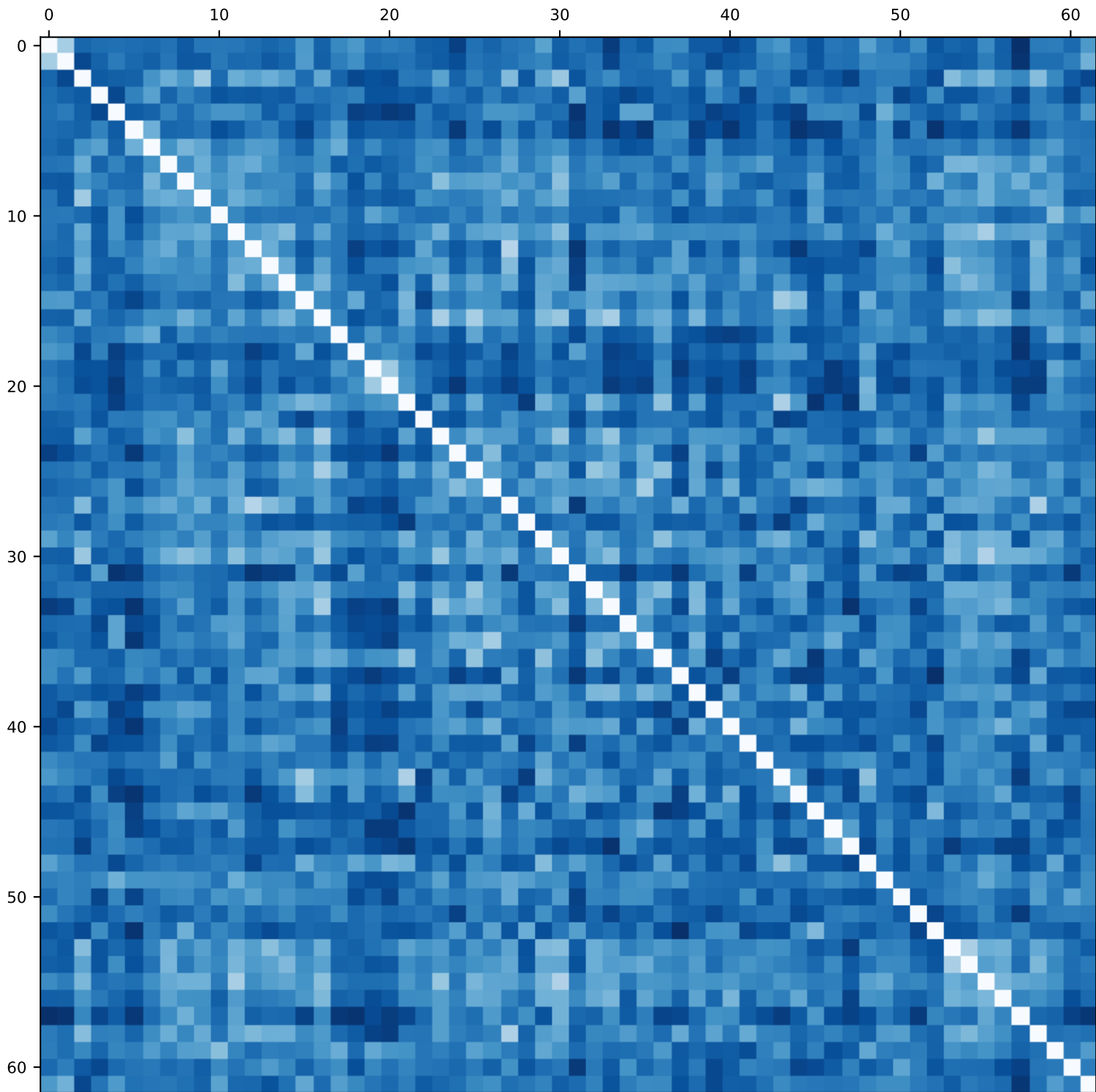

Supplement: Supplementary file 1 [file life-12-00541-s001.zip › life-1592845-supplementary/Heermann-cluster-gene-density-chr1.pdf]

chr2

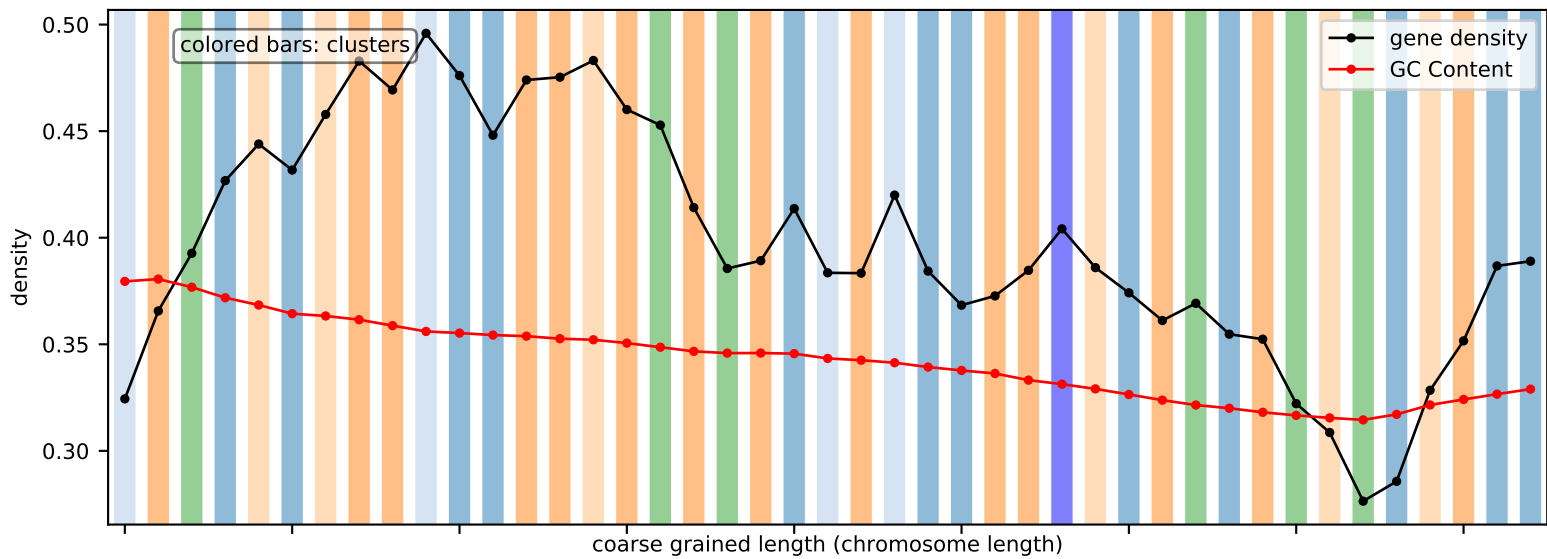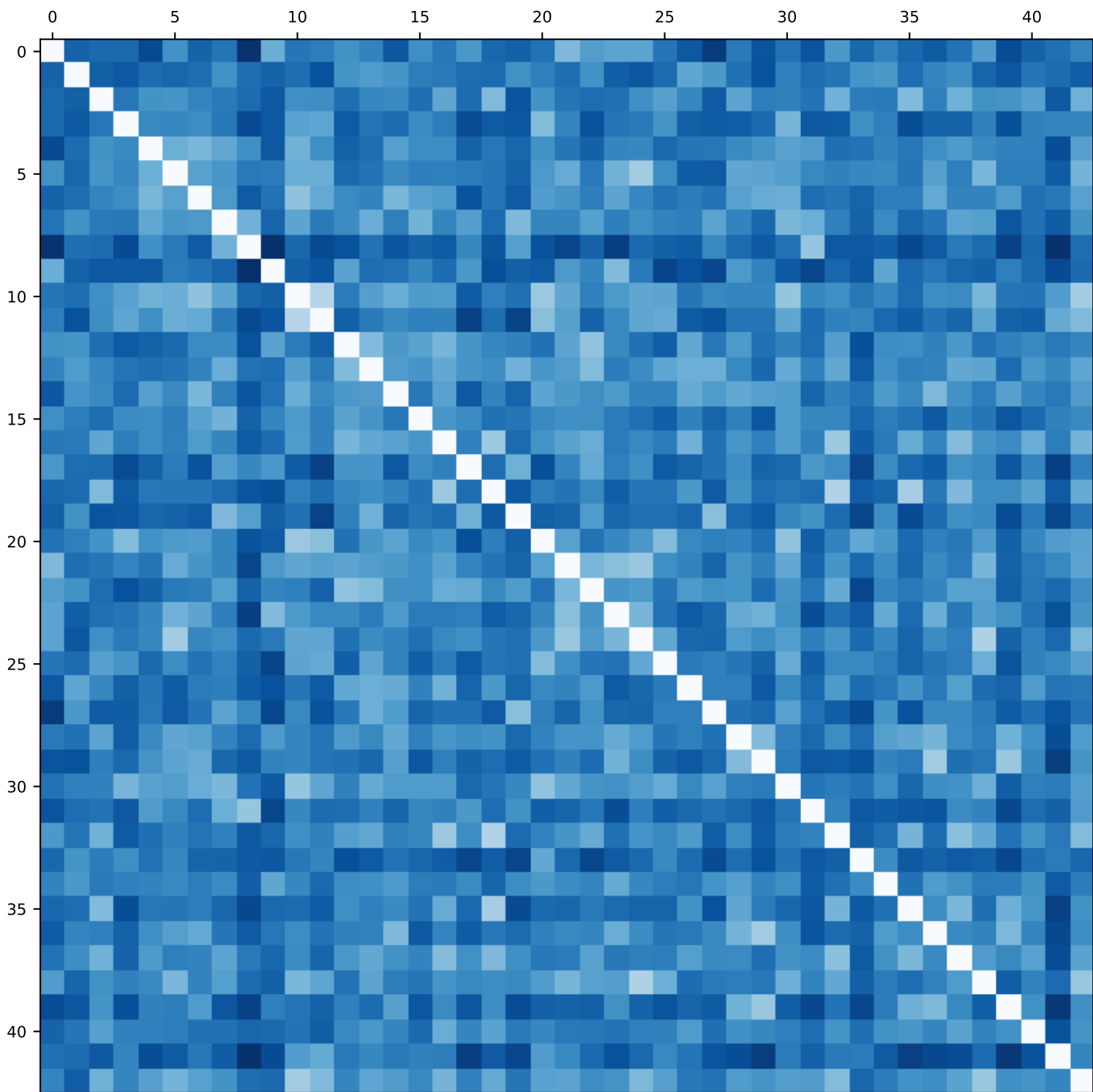

Supplement: Supplementary file 1 [file life-12-00541-s001.zip › life-1592845-supplementary/Heermann-cluster-gene-density-chr2.pdf]

chr3

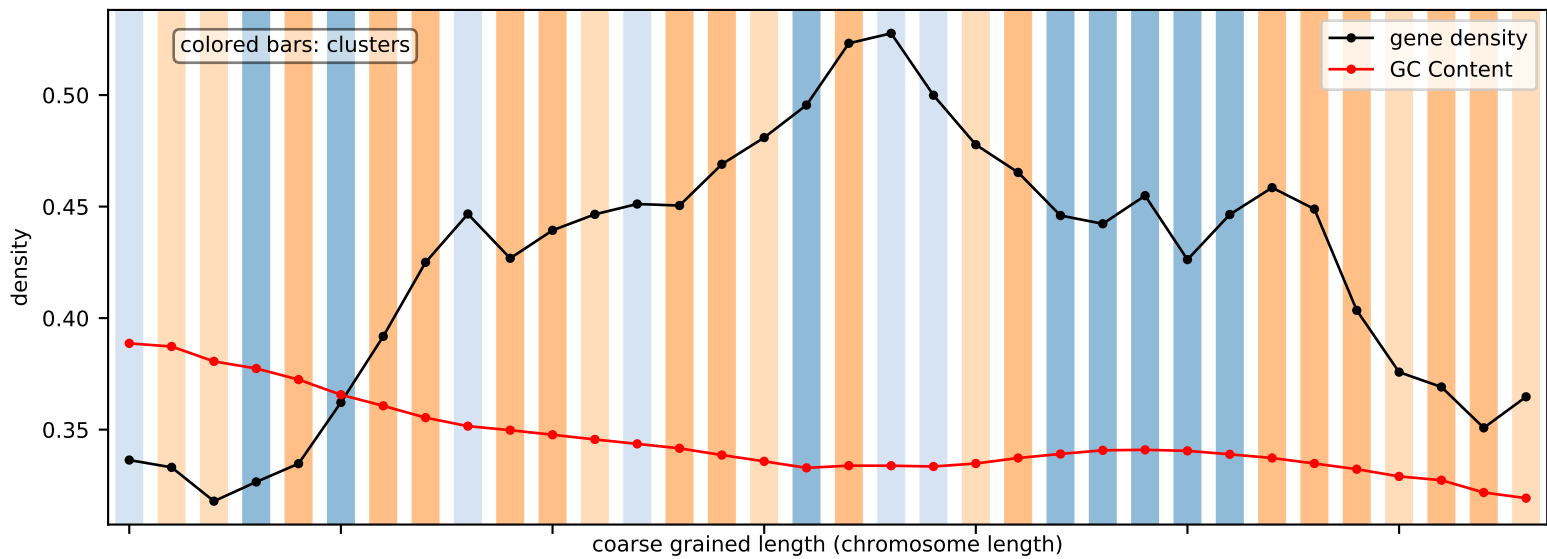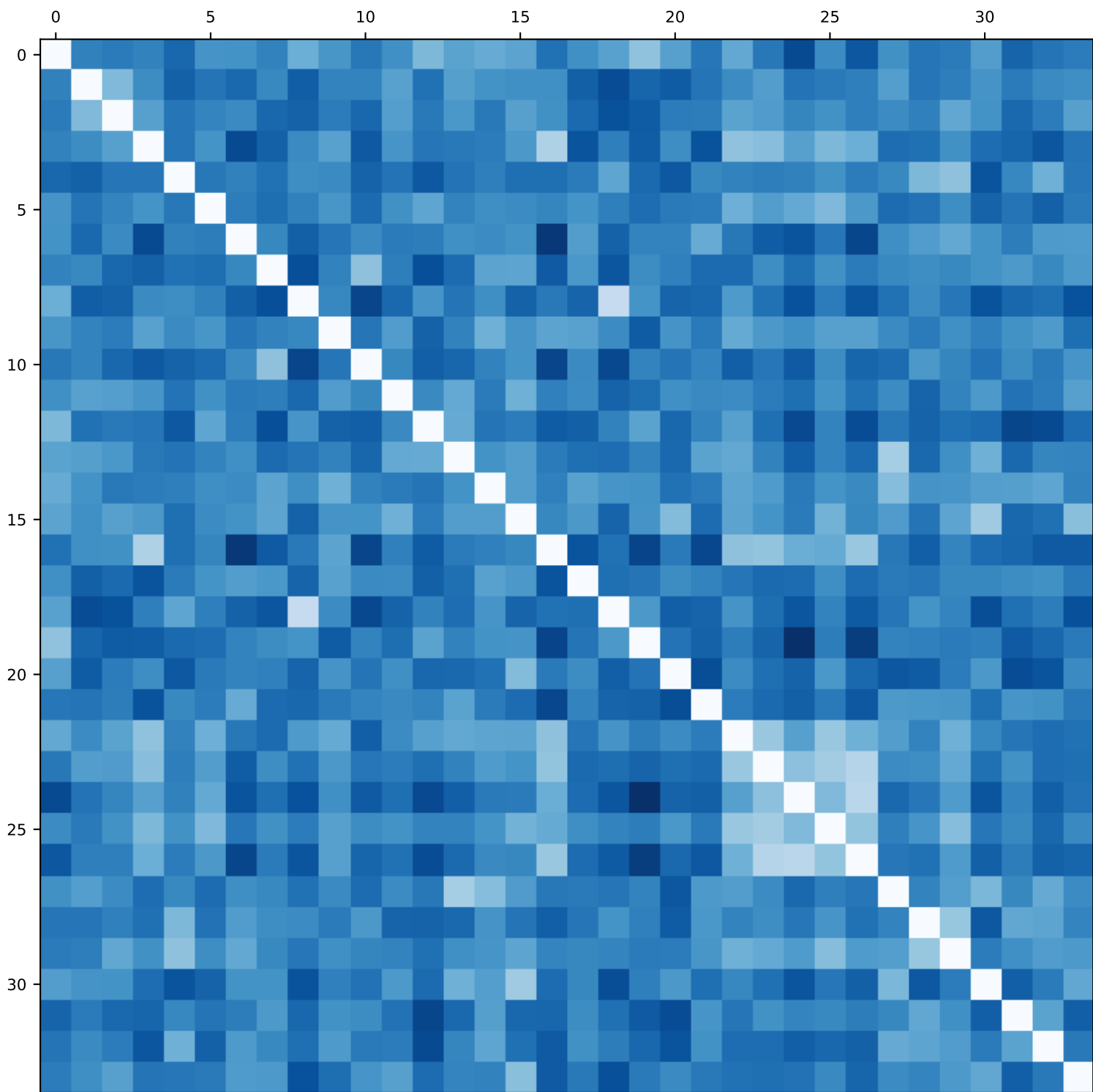

Supplement: Supplementary file 1 [file life-12-00541-s001.zip › life-1592845-supplementary/Heermann-cluster-gene-density-chr3.pdf]

# chr4

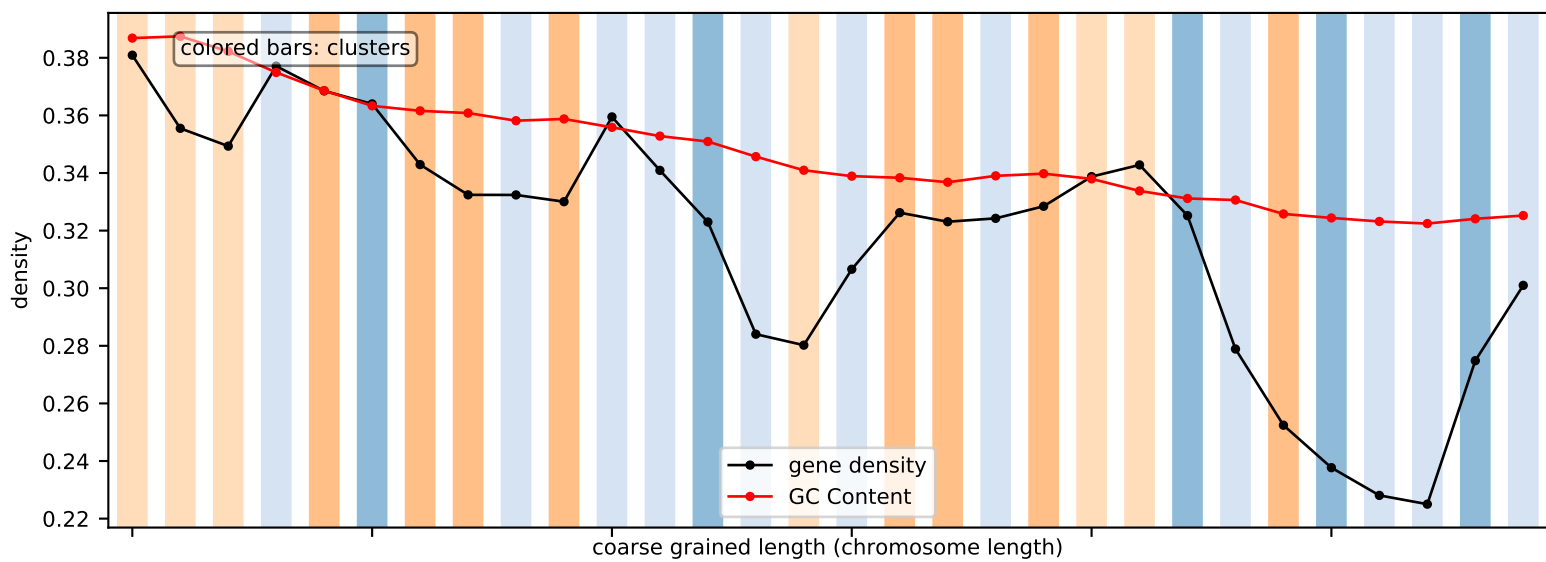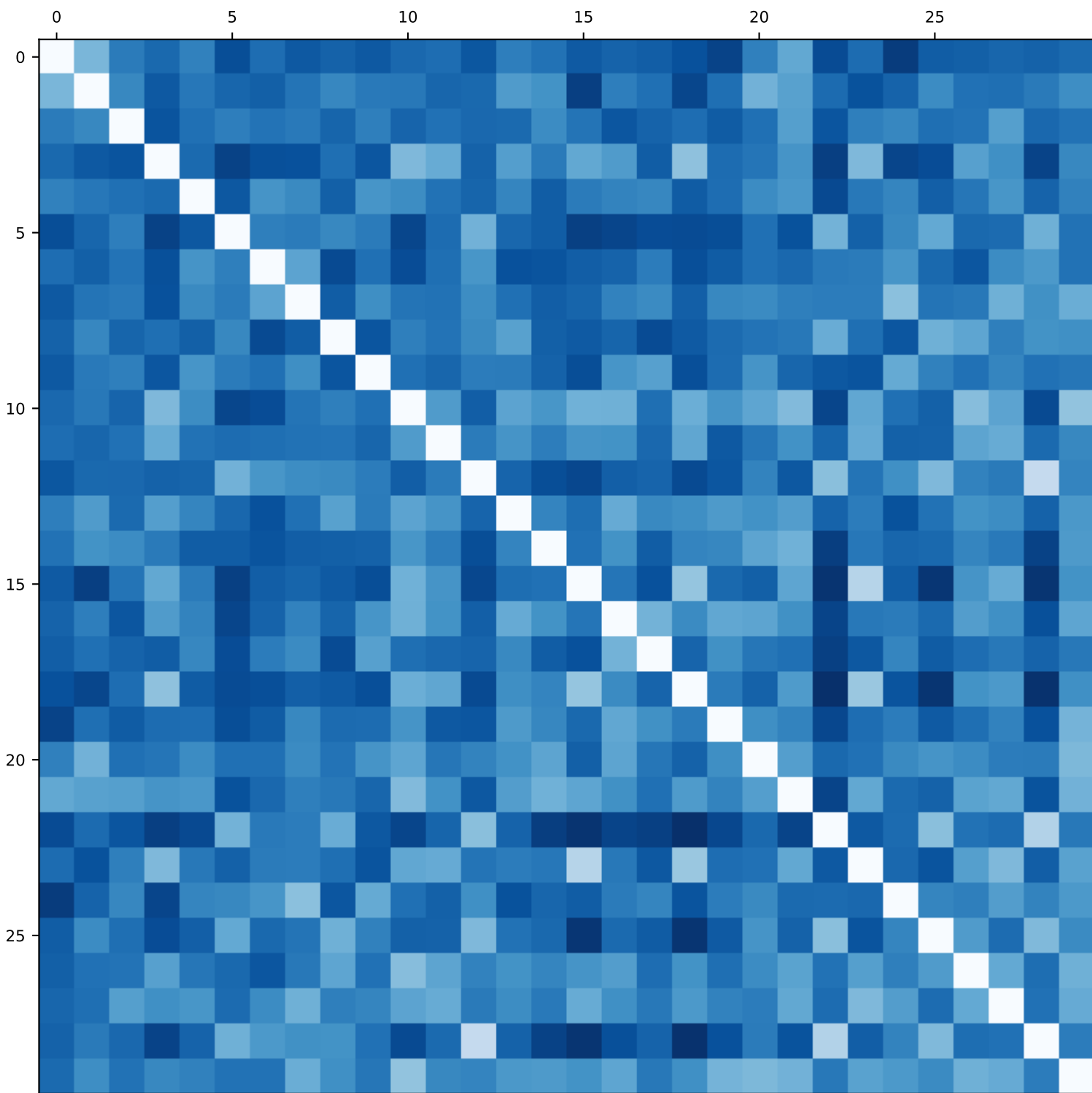

Supplement: Supplementary file 1 [file life-12-00541-s001.zip › life-1592845-supplementary/Heermann-cluster-gene-density-chr4.pdf]

chr5

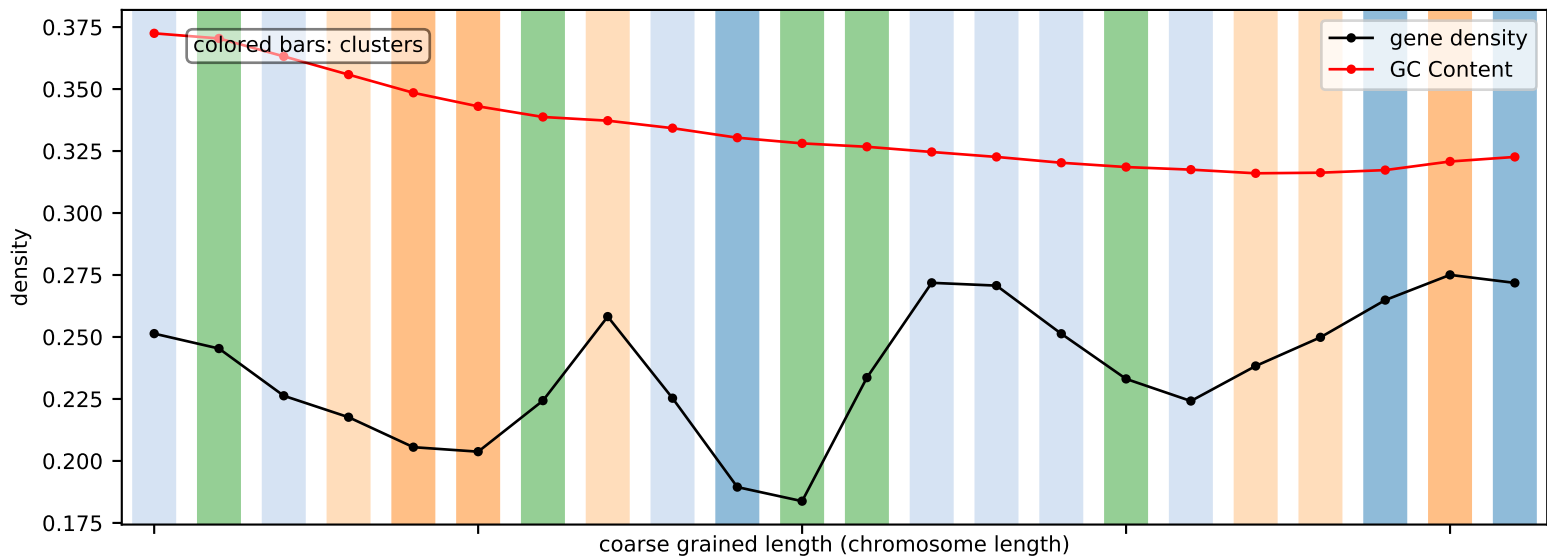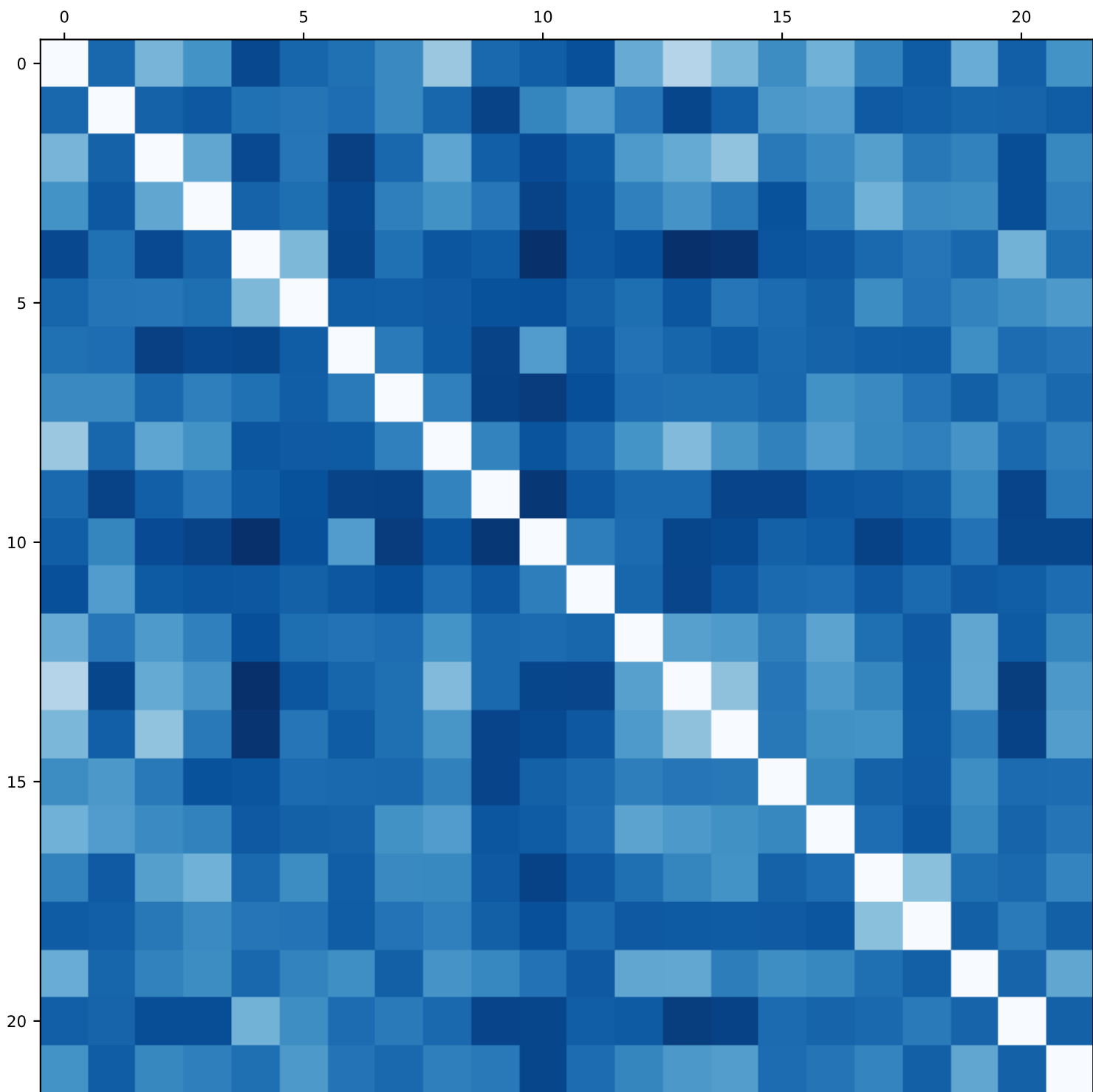

Supplement: Supplementary file 1 [file life-12-00541-s001.zip › life-1592845-supplementary/Heermann-cluster-gene-density-chr5.pdf]

chr6

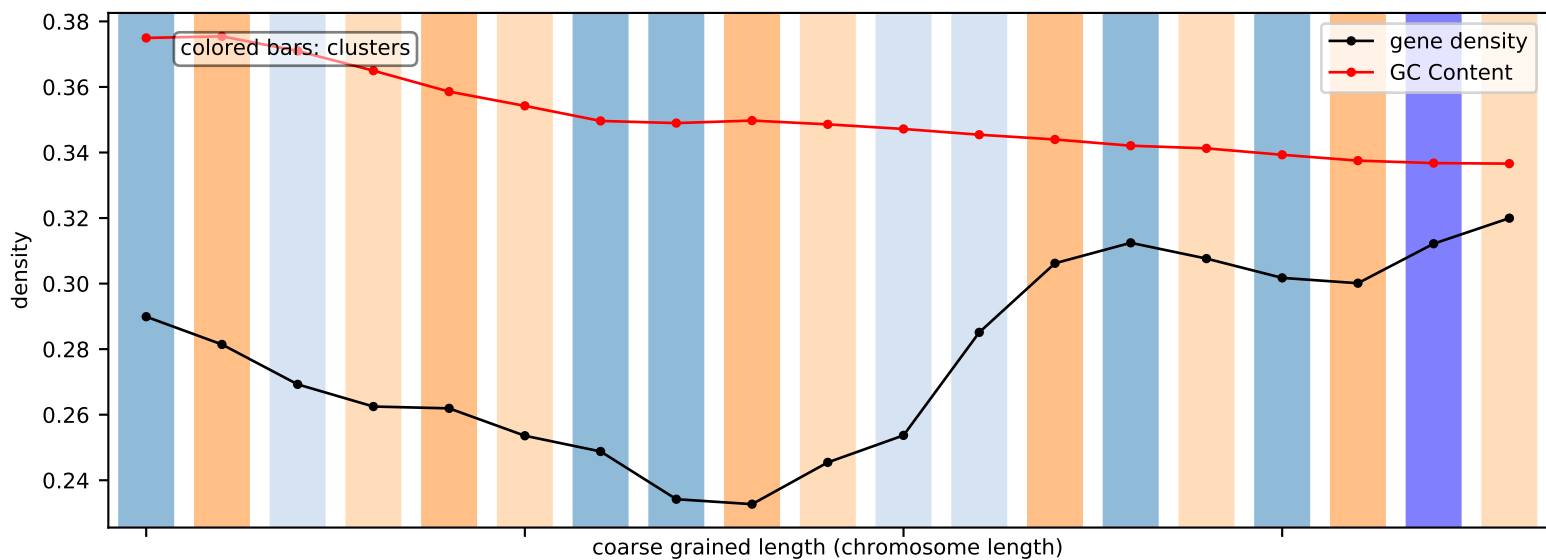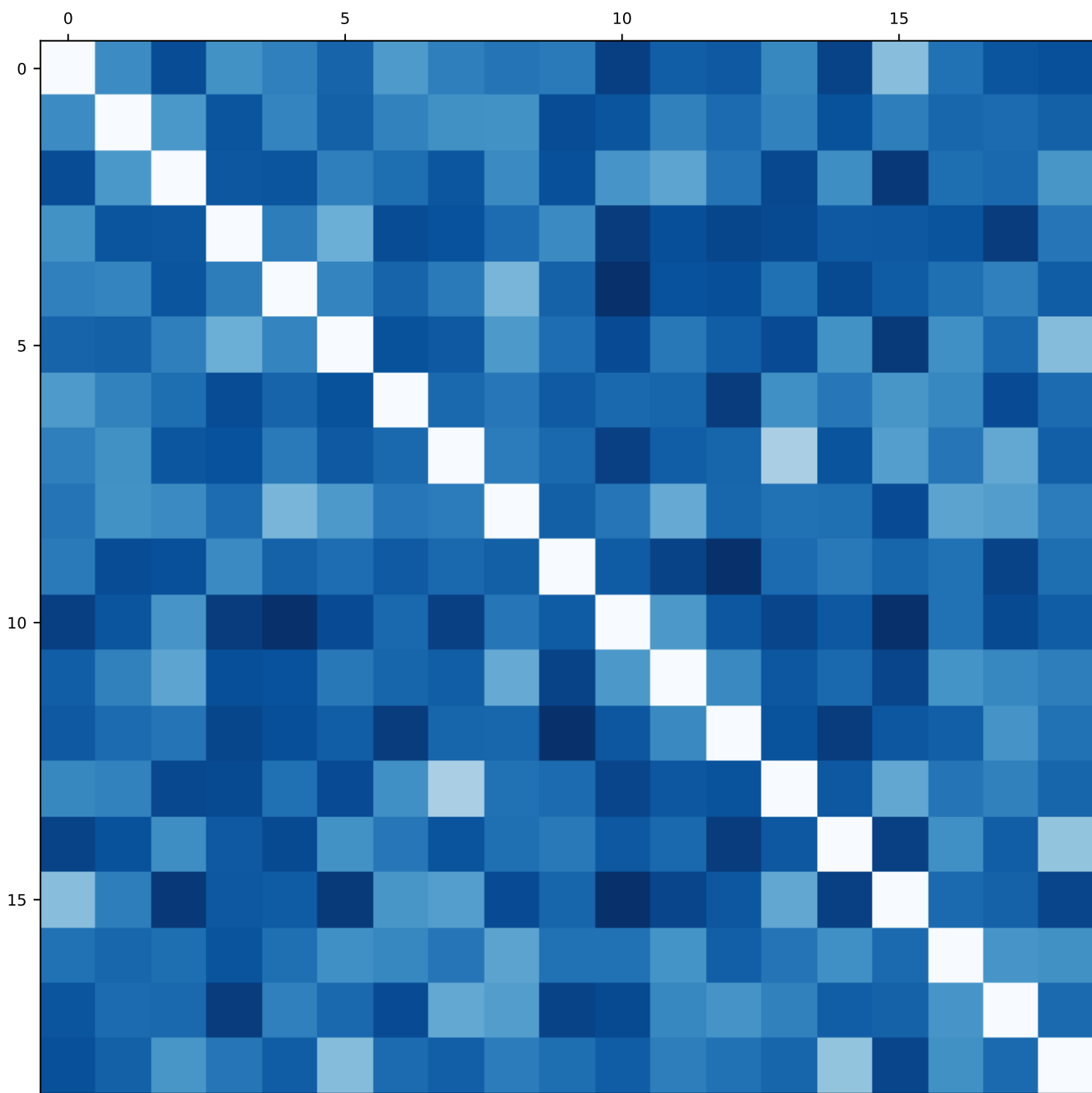

Supplement: Supplementary file 1 [file life-12-00541-s001.zip › life-1592845-supplementary/Heermann-cluster-gene-density-chr6.pdf]

chr7

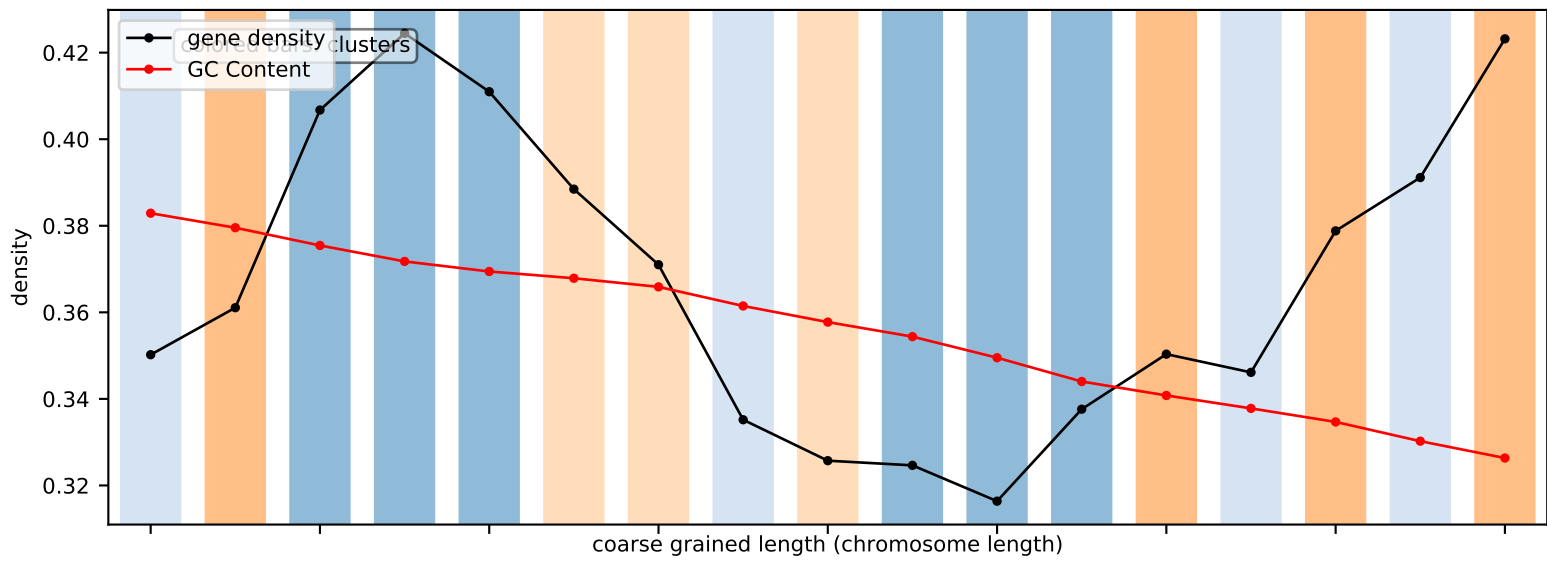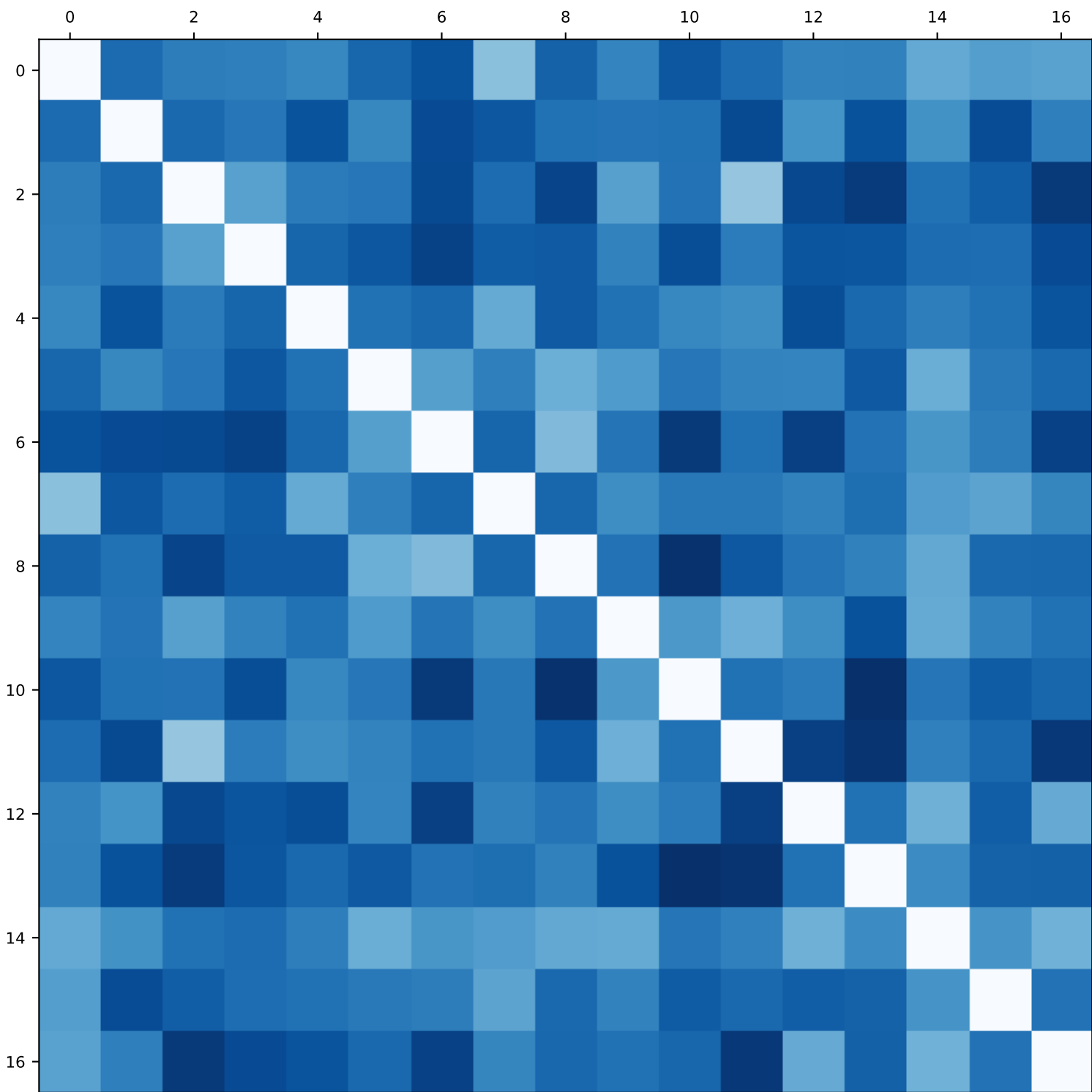

Supplement: Supplementary file 1 [file life-12-00541-s001.zip › life-1592845-supplementary/Heermann-cluster-gene-density-chr7.pdf]

# chrR

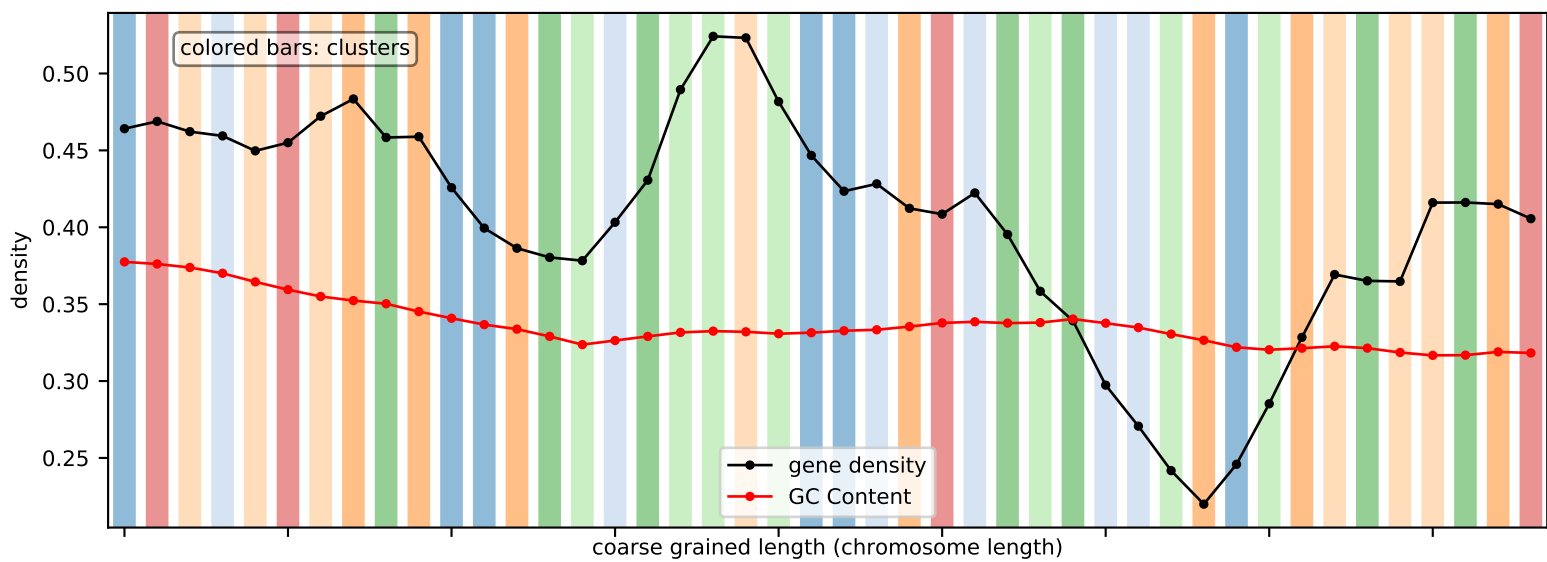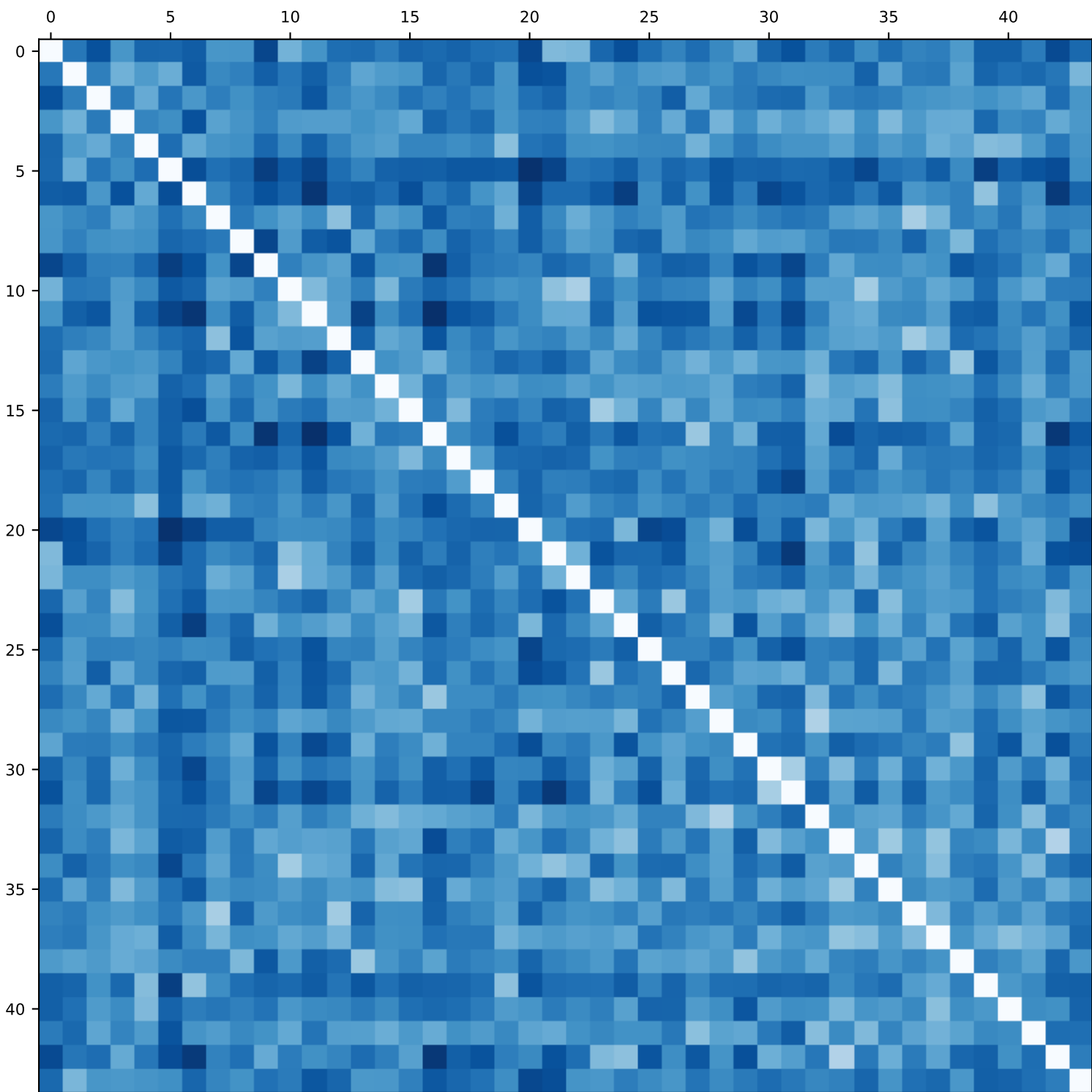

Supplement: Supplementary file 1 [file life-12-00541-s001.zip › life-1592845-supplementary/Heermann-cluster-gene-density-chrR.pdf]

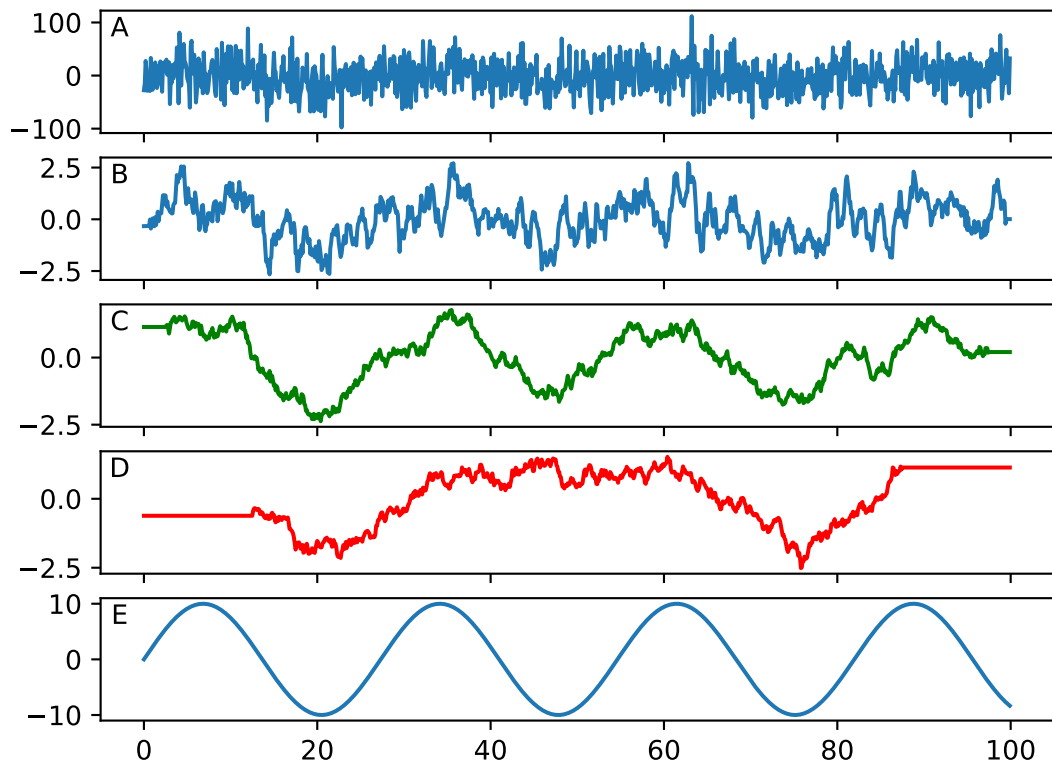

Supplement: Supplementary file 1 [file life-12-00541-s001.zip › life-1592845-supplementary/Heermann-coarse-graining-scheme.pdf]

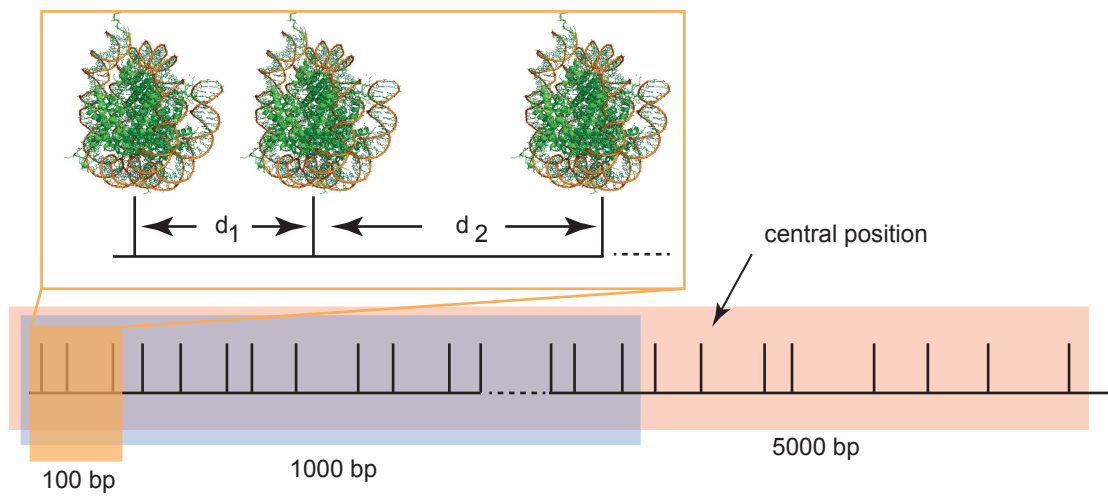

Supplement: Supplementary file 1 [file life-12-00541-s001.zip › life-1592845-supplementary/Heermann-coarse-graining.pdf]

Dendrogram for Chromosomes: chr1-chr2-chr3-chr4-chr5-chr6-chr7-chrR

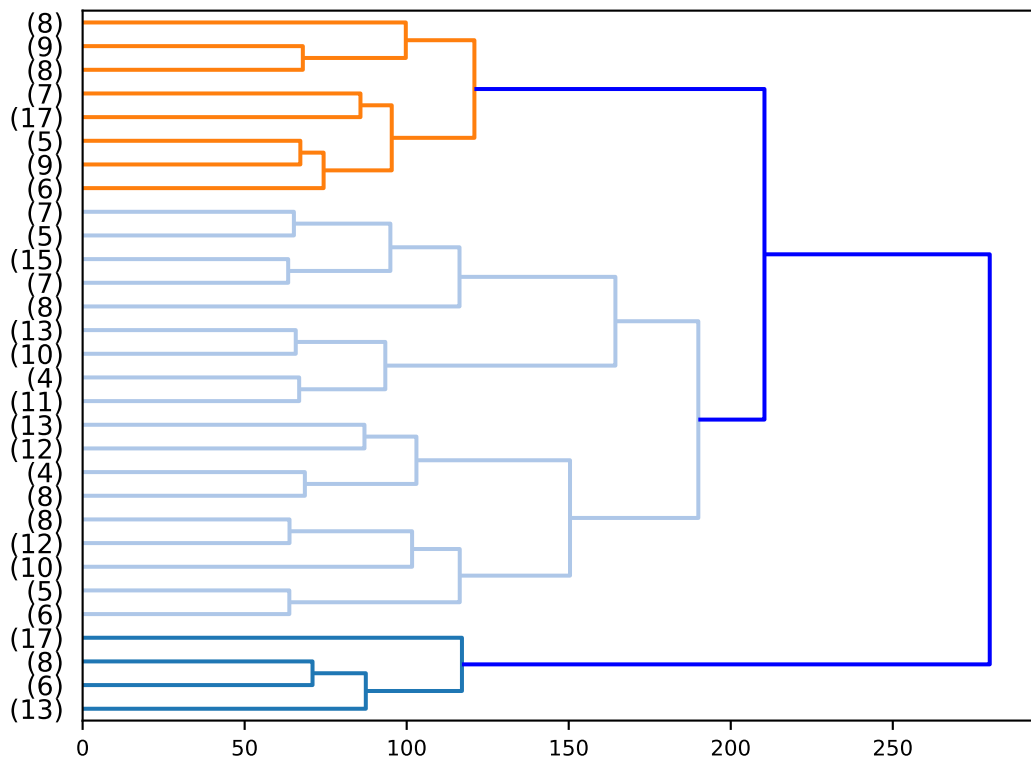

Supplement: Supplementary file 1 [file life-12-00541-s001.zip › life-1592845-supplementary/Heermann-dendrogram-chr1-chr2-chr3-chr4-chr5-chr6-chr7-chrR.pdf]

# Analysis for Chromosome: chr1

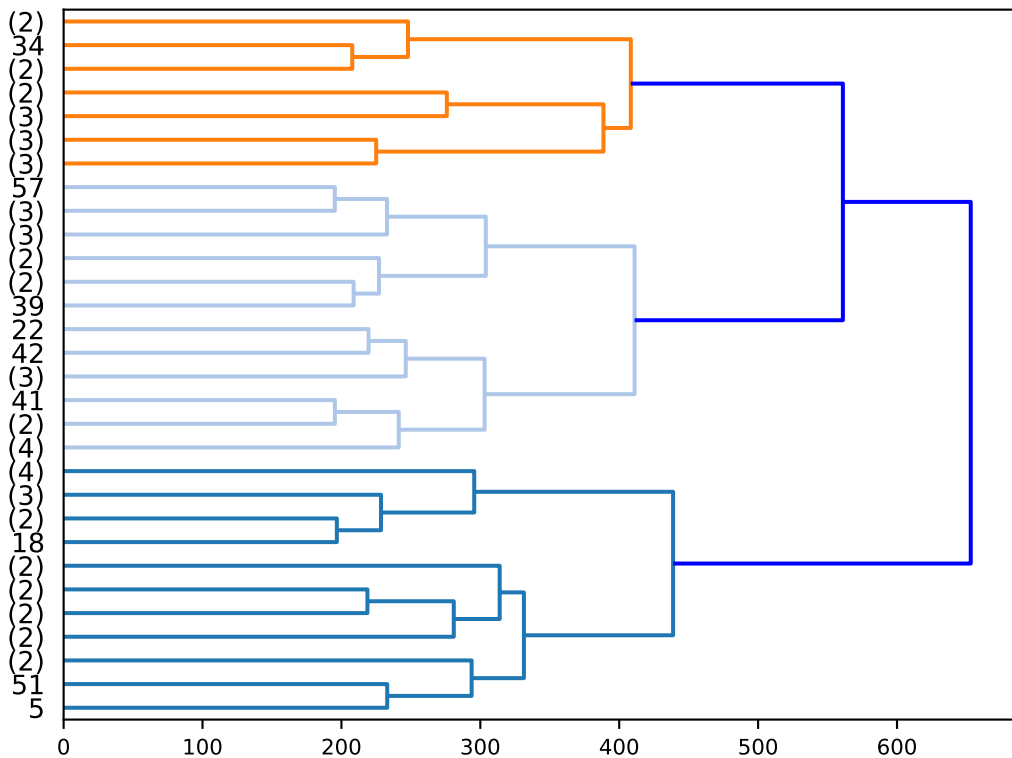

Supplement: Supplementary file 1 [file life-12-00541-s001.zip › life-1592845-supplementary/Heermann-dendrogram-chr1.pdf]

# Analysis for Chromosome: chr2

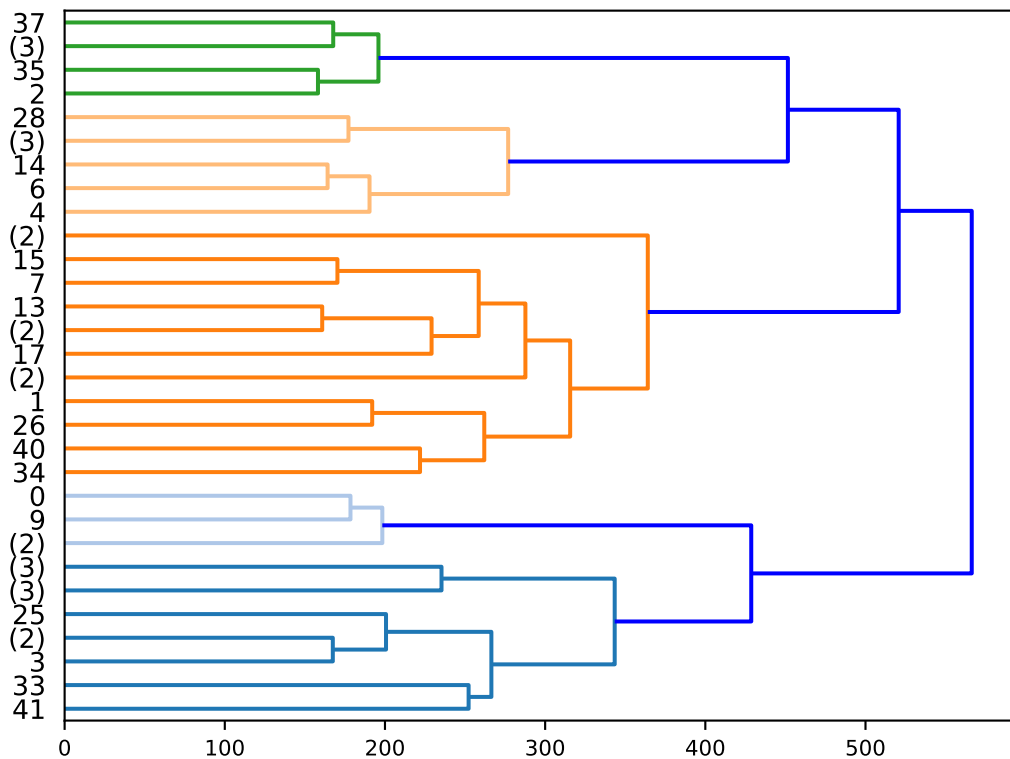

Supplement: Supplementary file 1 [file life-12-00541-s001.zip › life-1592845-supplementary/Heermann-dendrogram-chr2.pdf]

# Analysis for Chromosome: chr3

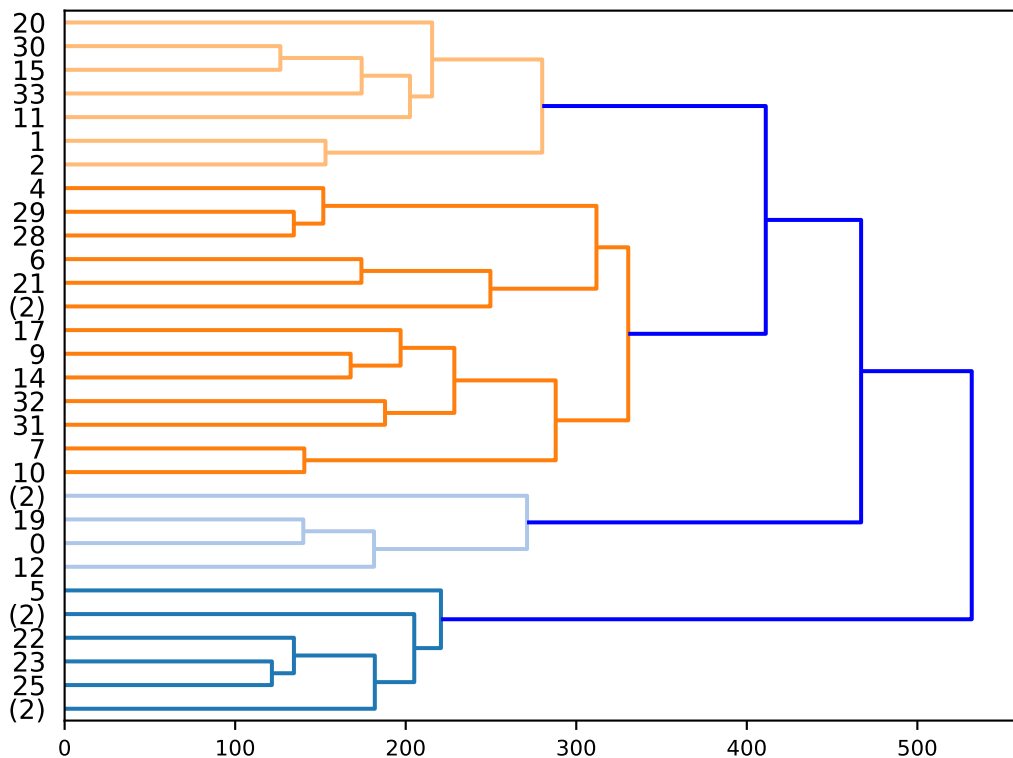

Supplement: Supplementary file 1 [file life-12-00541-s001.zip › life-1592845-supplementary/Heermann-dendrogram-chr3.pdf]

# Analysis for Chromosome: chr4

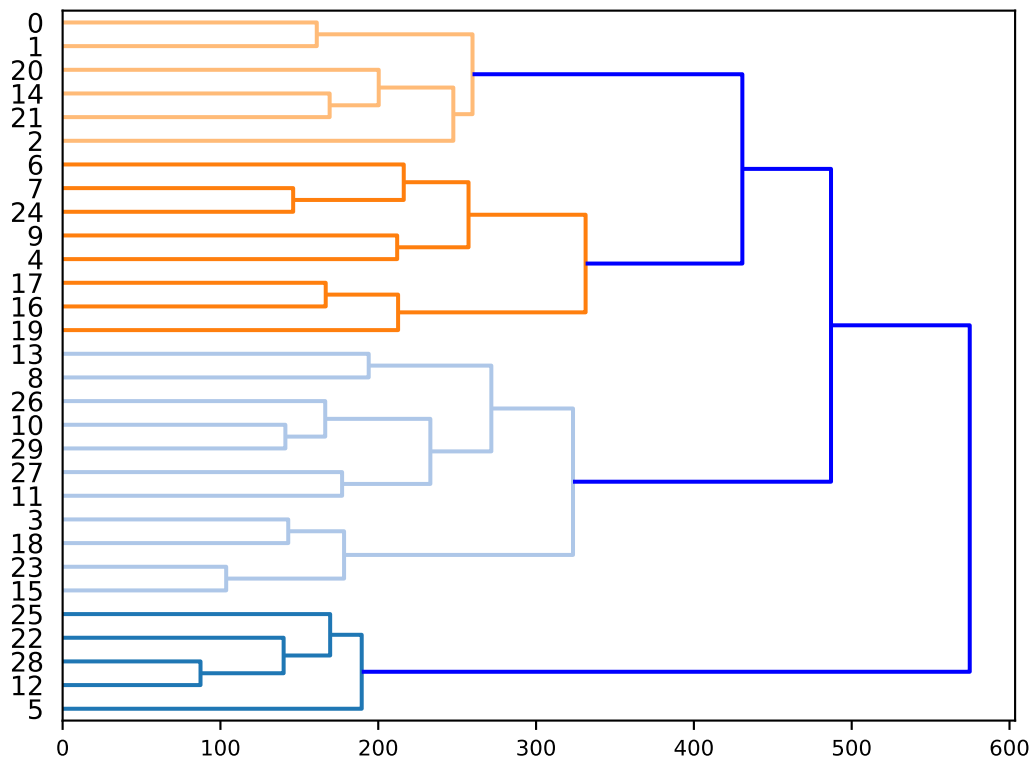

Supplement: Supplementary file 1 [file life-12-00541-s001.zip › life-1592845-supplementary/Heermann-dendrogram-chr4.pdf]

# Analysis for Chromosome: chr5

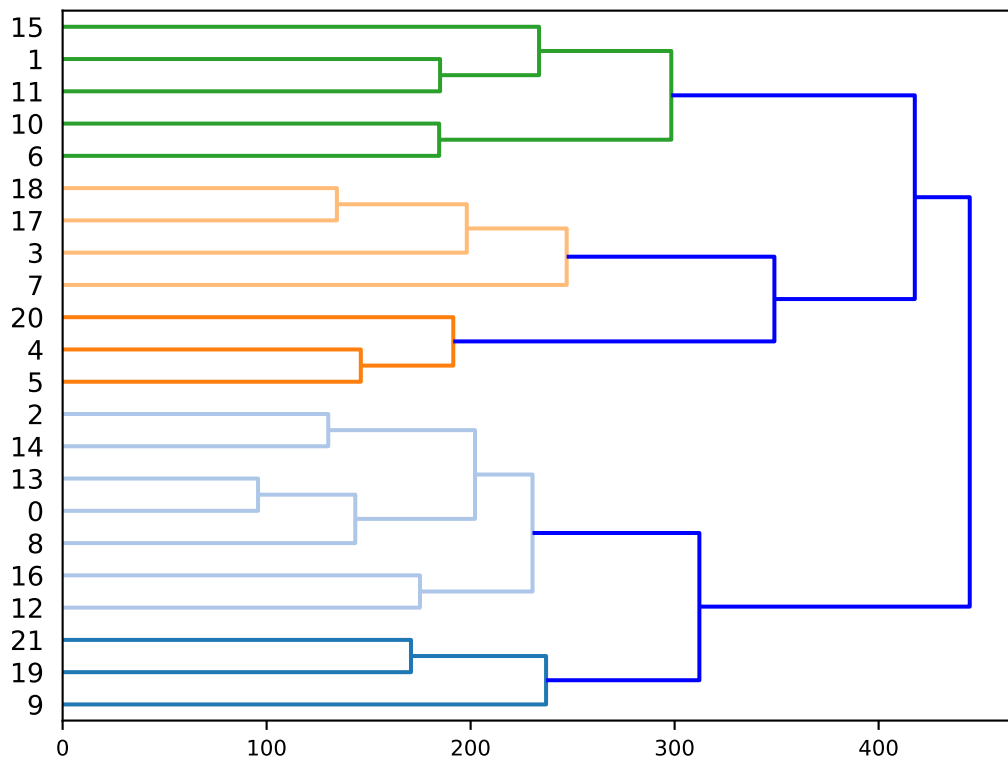

Supplement: Supplementary file 1 [file life-12-00541-s001.zip › life-1592845-supplementary/Heermann-dendrogram-chr5.pdf]

# Analysis for Chromosome: chr6

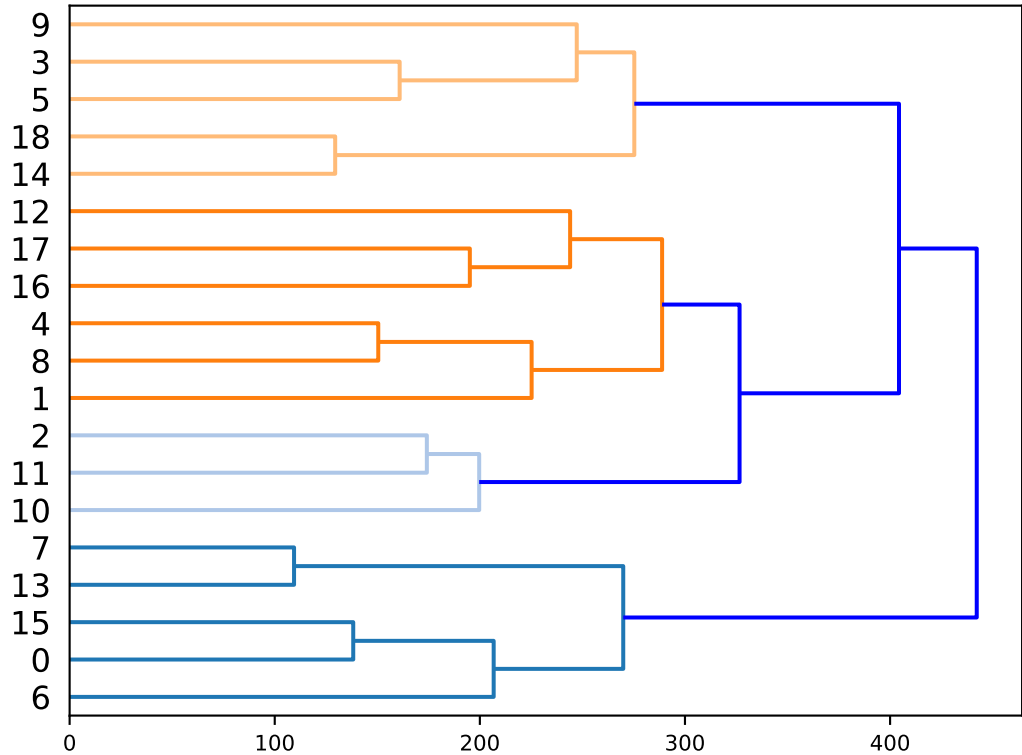

Supplement: Supplementary file 1 [file life-12-00541-s001.zip › life-1592845-supplementary/Heermann-dendrogram-chr6.pdf]

# Analysis for Chromosome: chr7

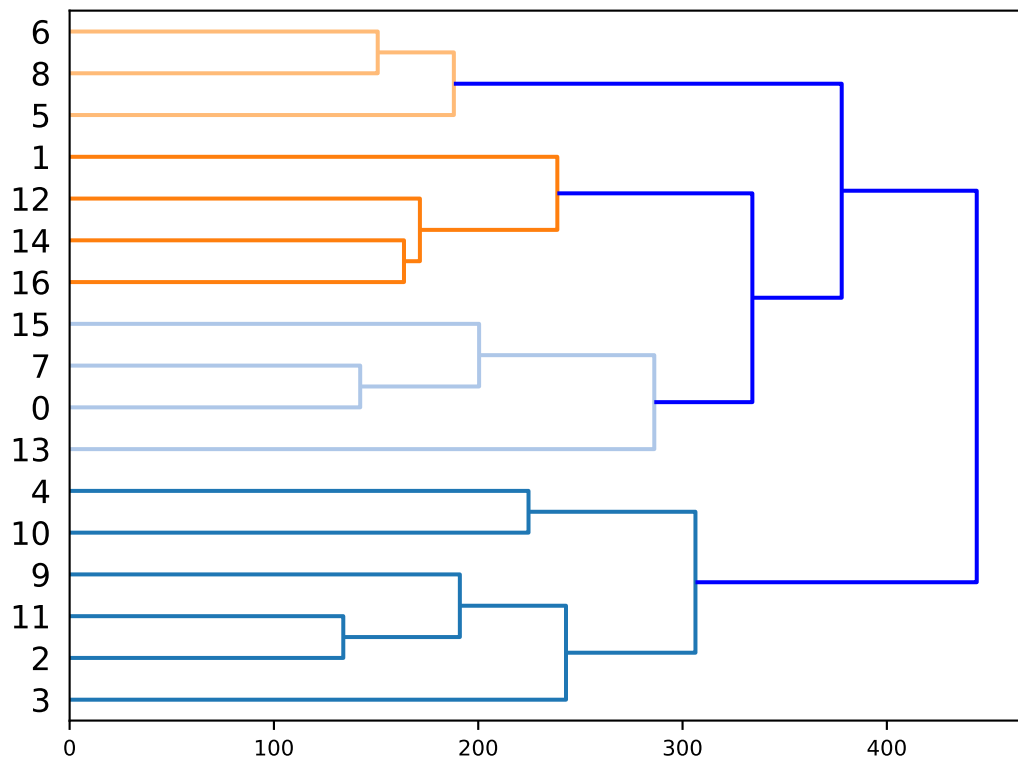

Supplement: Supplementary file 1 [file life-12-00541-s001.zip › life-1592845-supplementary/Heermann-dendrogram-chr7.pdf]

# Analysis for Chromosome: chrR

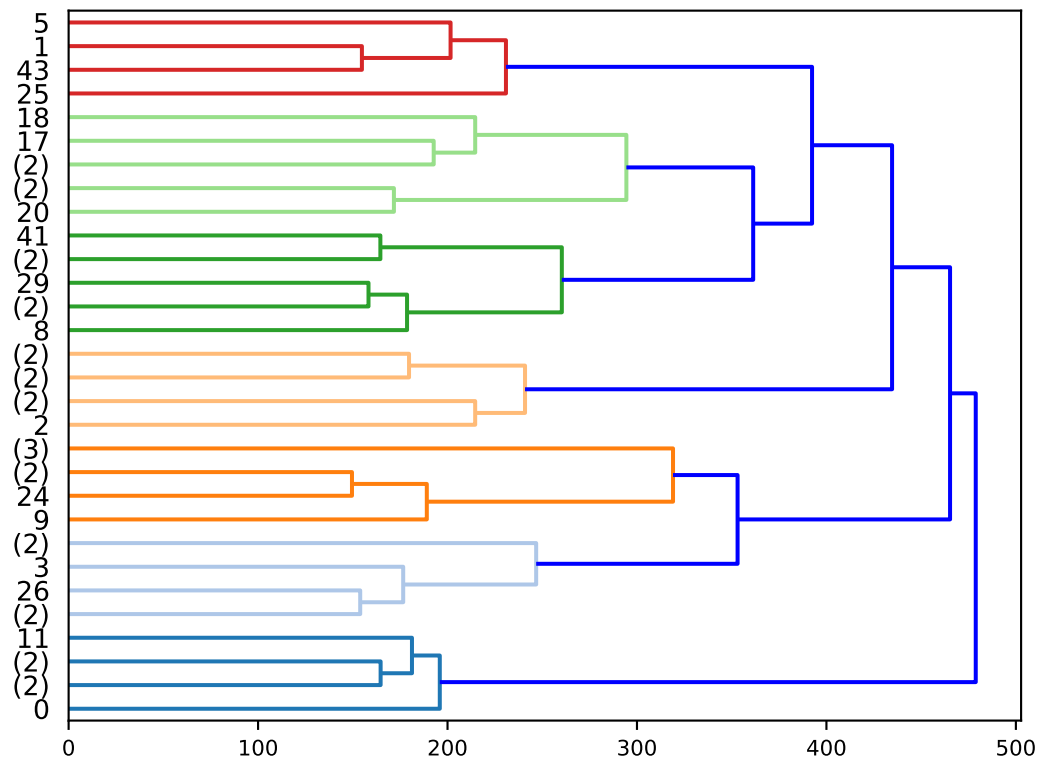

Supplement: Supplementary file 1 [file life-12-00541-s001.zip › life-1592845-supplementary/Heermann-dendrogram-chrR.pdf]

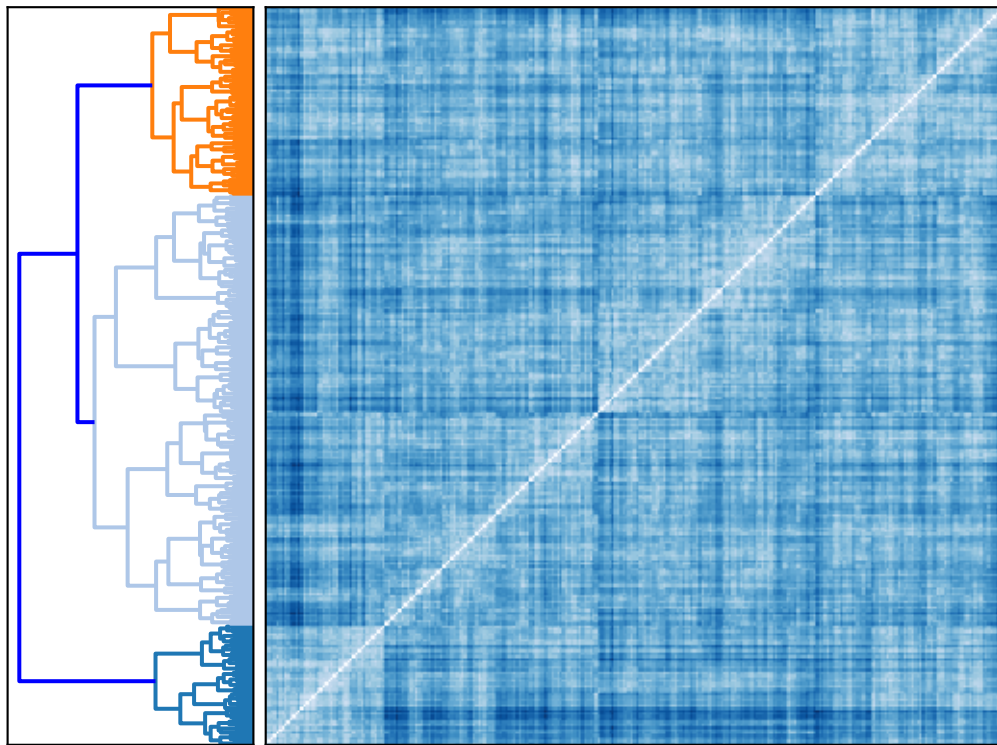

chr1-chr2-chr3-chr4-chr5-chr6-chr7-chrR

Supplement: Supplementary file 1 [file life-12-00541-s001.zip › life-1592845-supplementary/Heermann-dendrogram-distance-matrix-chr1-chr2-chr3-chr4-chr5-chr6-chr7-chrR.pdf]

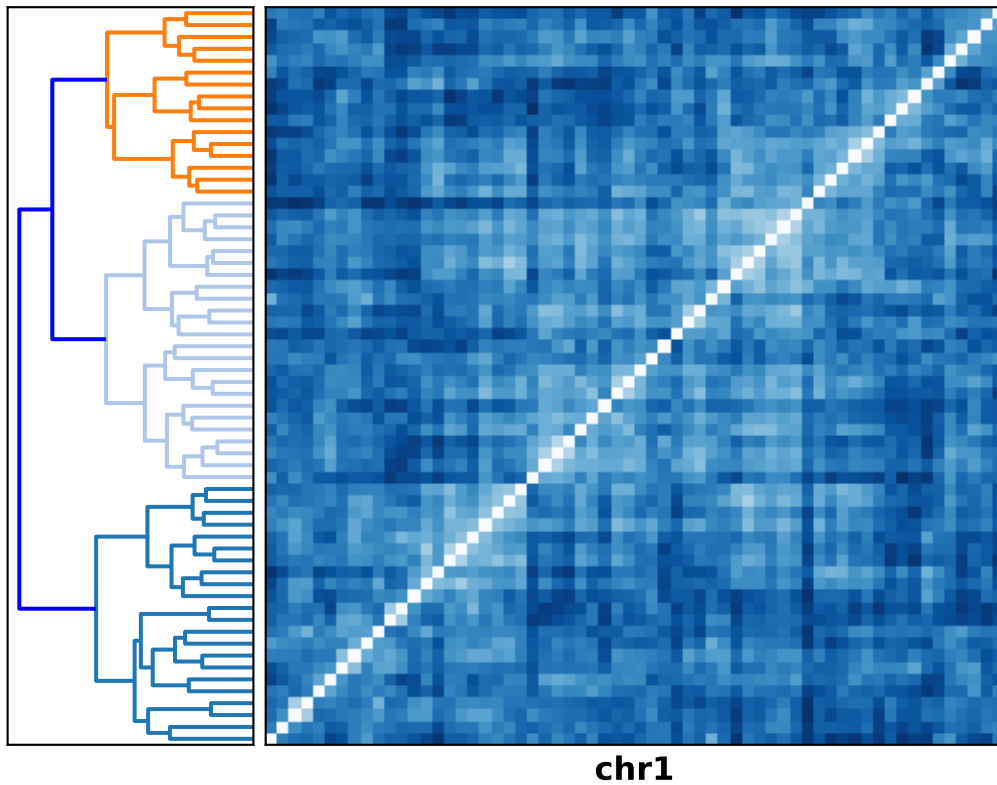

Supplement: Supplementary file 1 [file life-12-00541-s001.zip › life-1592845-supplementary/Heermann-dendrogram-distance-matrix-chr1.pdf]

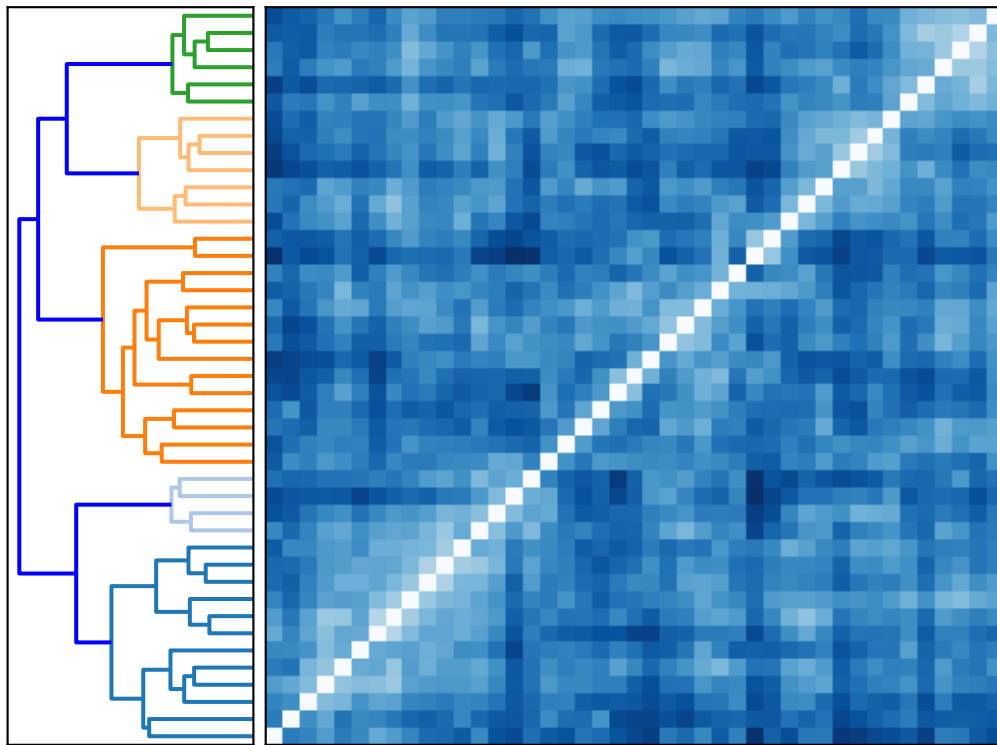

**chr2**

Supplement: Supplementary file 1 [file life-12-00541-s001.zip › life-1592845-supplementary/Heermann-dendrogram-distance-matrix-chr2.pdf]

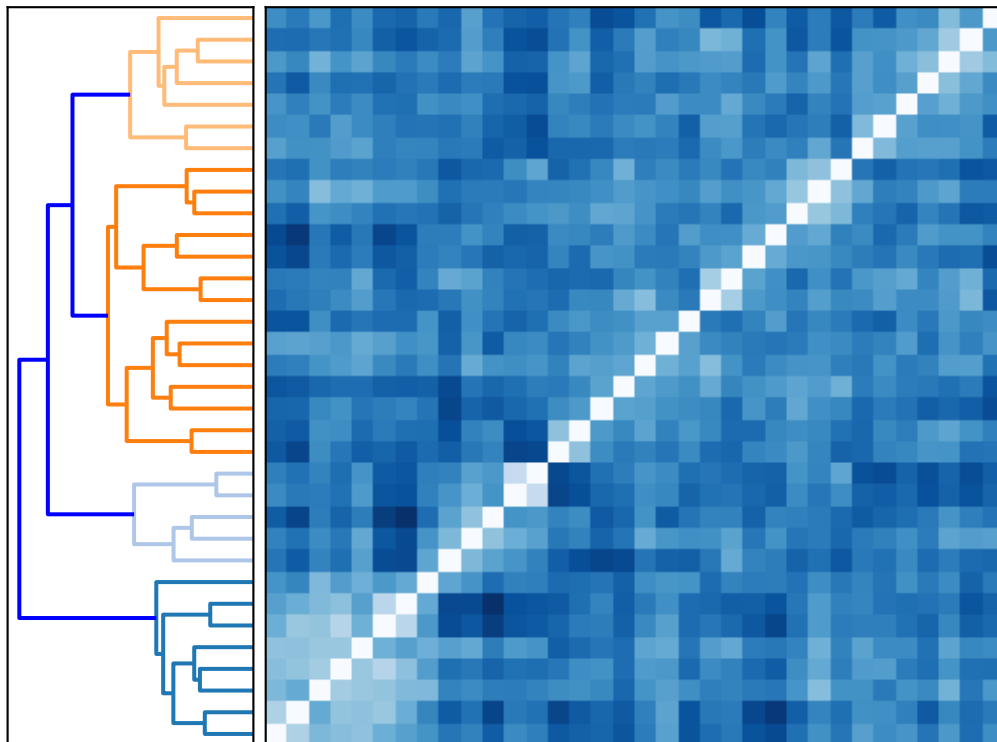

**chr3**

Supplement: Supplementary file 1 [file life-12-00541-s001.zip › life-1592845-supplementary/Heermann-dendrogram-distance-matrix-chr3.pdf]

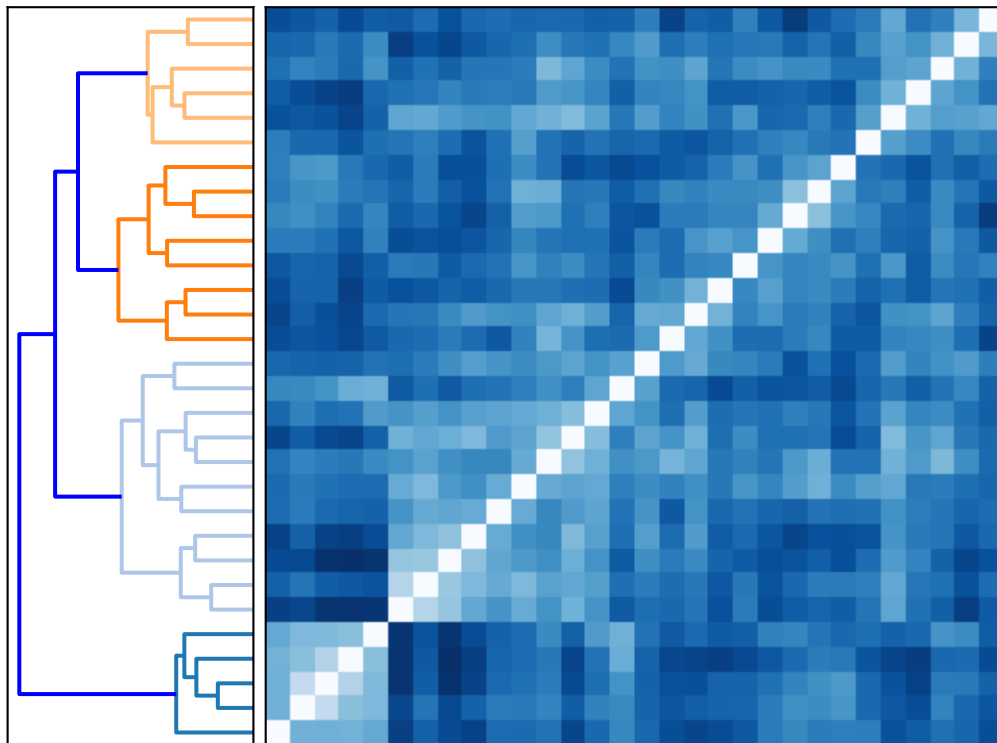

**chr4**

Supplement: Supplementary file 1 [file life-12-00541-s001.zip › life-1592845-supplementary/Heermann-dendrogram-distance-matrix-chr4.pdf]

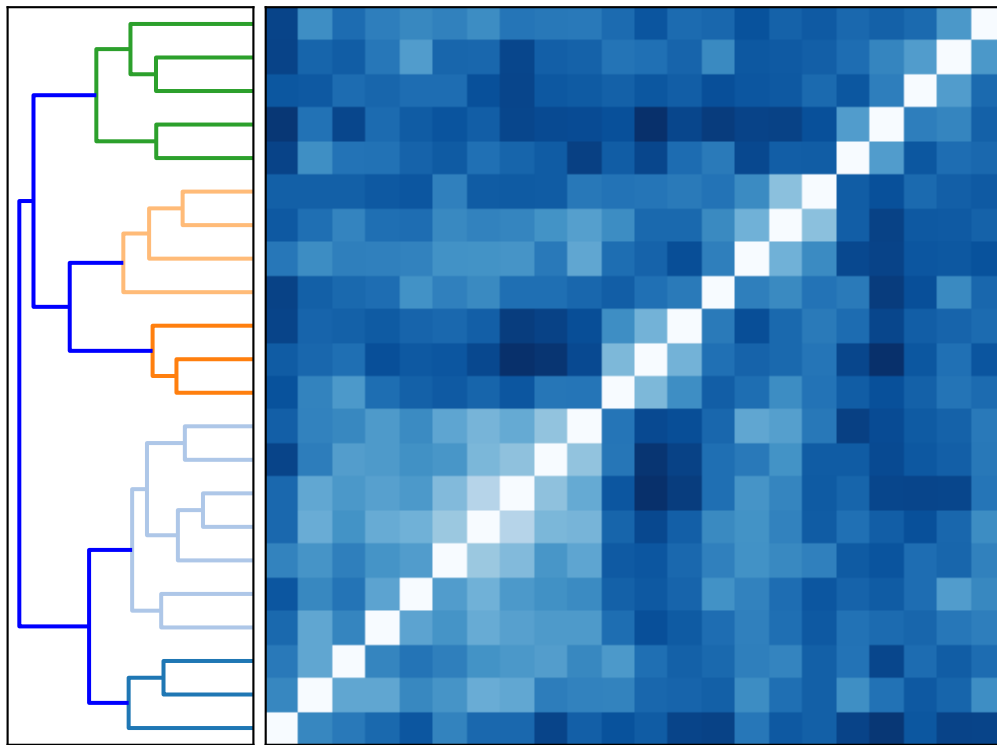

**chr5**

Supplement: Supplementary file 1 [file life-12-00541-s001.zip › life-1592845-supplementary/Heermann-dendrogram-distance-matrix-chr5.pdf]

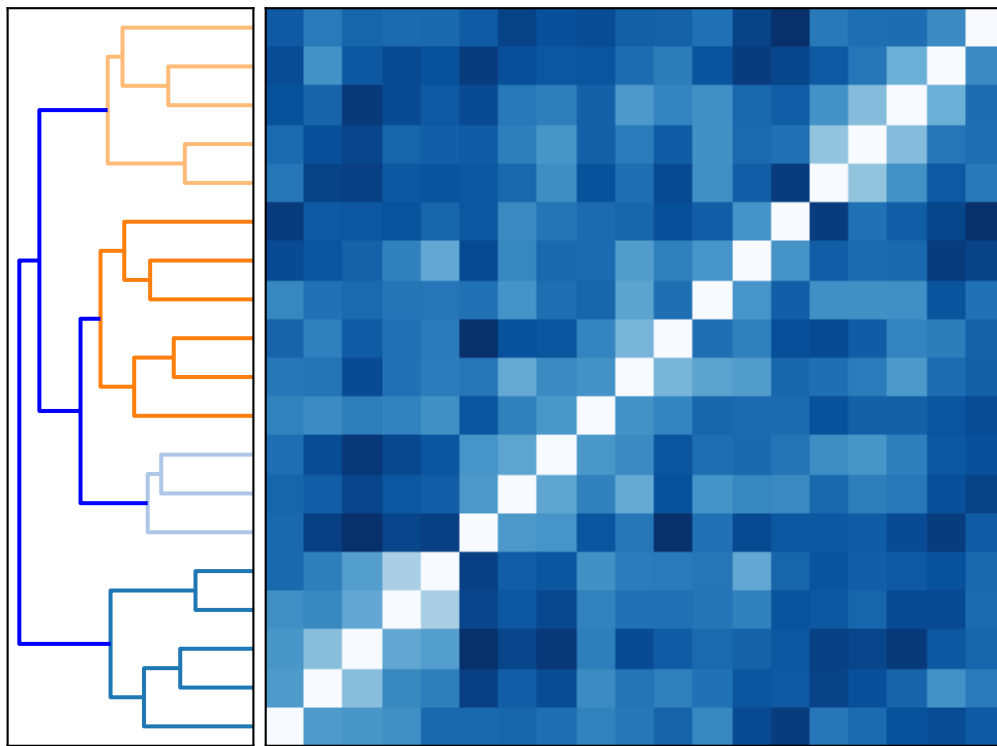

**chr6**

Supplement: Supplementary file 1 [file life-12-00541-s001.zip › life-1592845-supplementary/Heermann-dendrogram-distance-matrix-chr6.pdf]

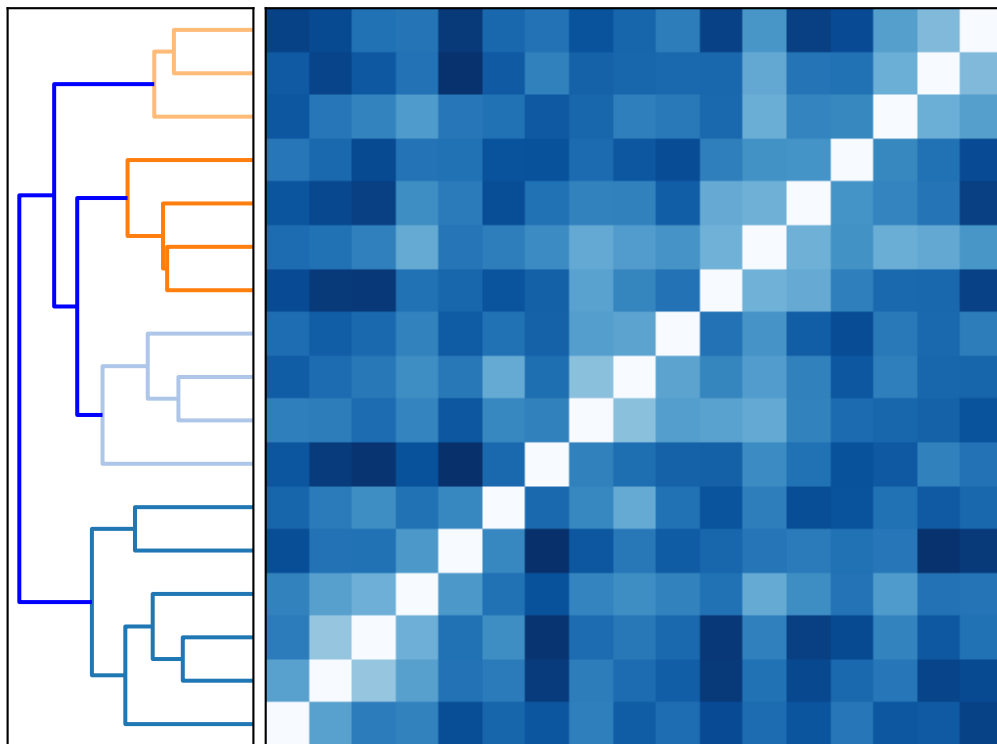

**chr7**

Supplement: Supplementary file 1 [file life-12-00541-s001.zip › life-1592845-supplementary/Heermann-dendrogram-distance-matrix-chr7.pdf]

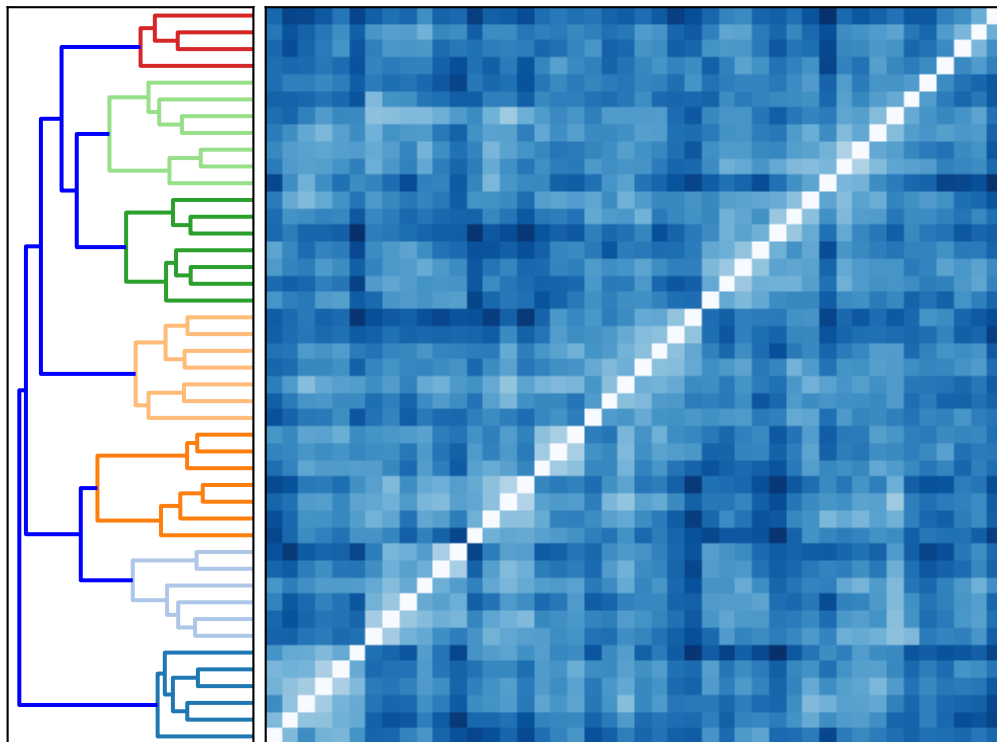

**chrR**

Supplement: Supplementary file 1 [file life-12-00541-s001.zip › life-1592845-supplementary/Heermann-dendrogram-distance-matrix-chrR.pdf]

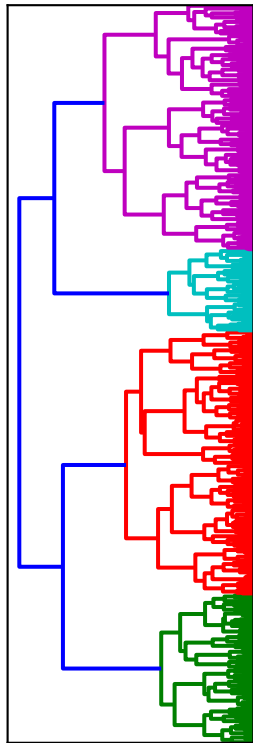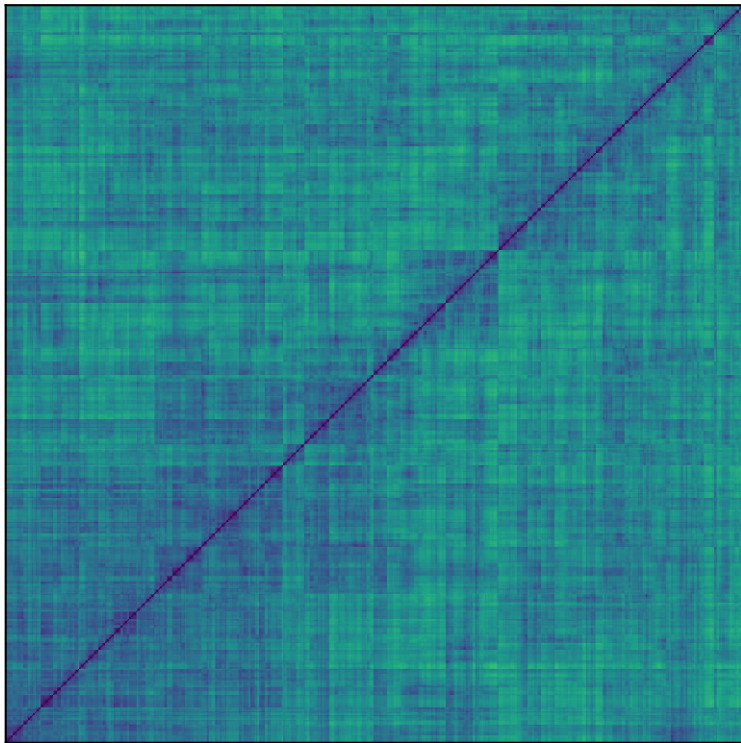

Supplement: Supplementary file 1 [file life-12-00541-s001.zip › life-1592845-supplementary/Heermann-dendrogram-distance-matrix.pdf]

chr1-chr2-chr3-chr4-chr5-chr6-chr7-chrR

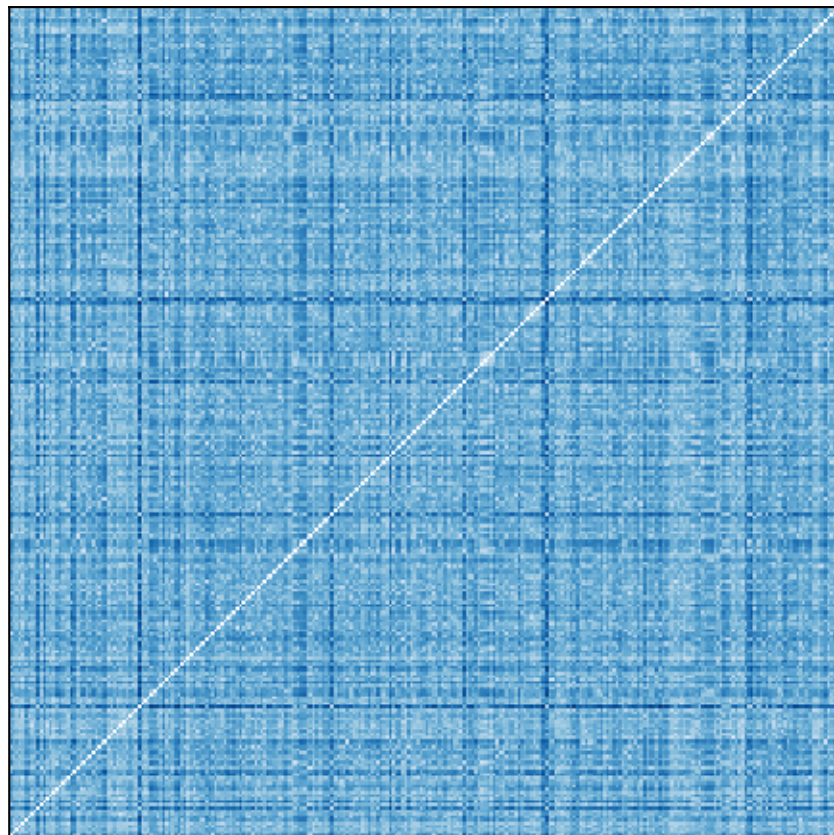

chr1-chr2-chr3-chr4-chr5-chr6-chr7-chrR

Supplement: Supplementary file 1 [file life-12-00541-s001.zip › life-1592845-supplementary/Heermann-distance-matrix-chr1-chr2-chr3-chr4-chr5-chr6-chr7-chrR.pdf]

**chr1**

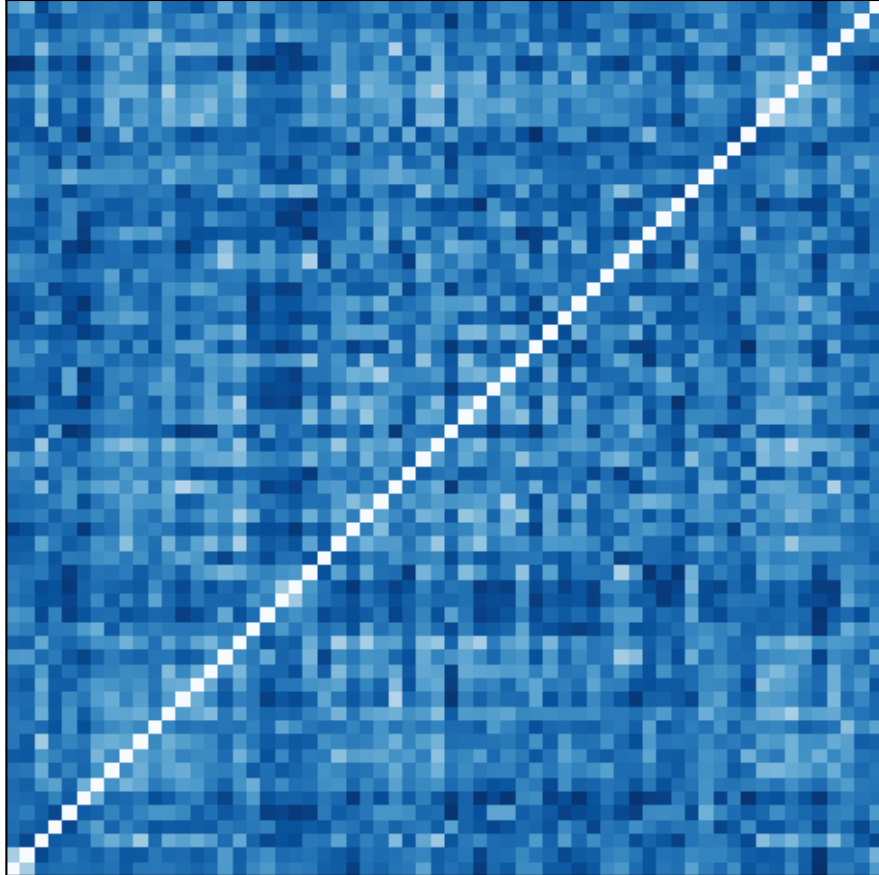

**chr1**

Supplement: Supplementary file 1 [file life-12-00541-s001.zip › life-1592845-supplementary/Heermann-distance-matrix-chr1.pdf]

chr2

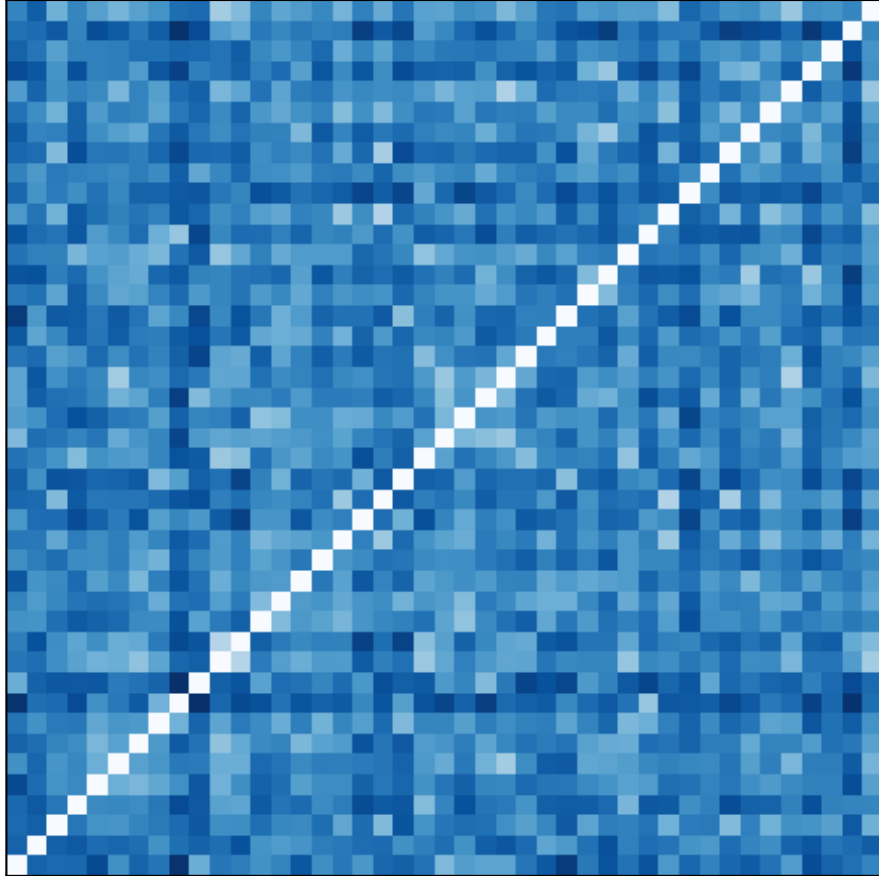

chr2

Supplement: Supplementary file 1 [file life-12-00541-s001.zip › life-1592845-supplementary/Heermann-distance-matrix-chr2.pdf]

chr3

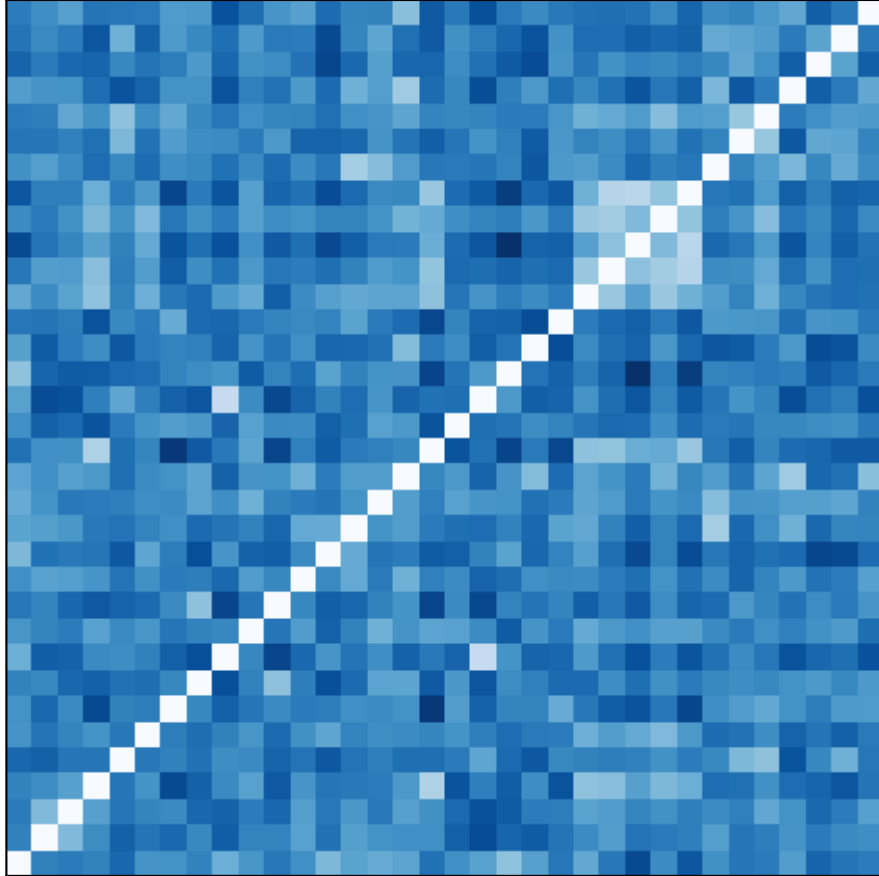

chr3

Supplement: Supplementary file 1 [file life-12-00541-s001.zip › life-1592845-supplementary/Heermann-distance-matrix-chr3.pdf]

**chr4**

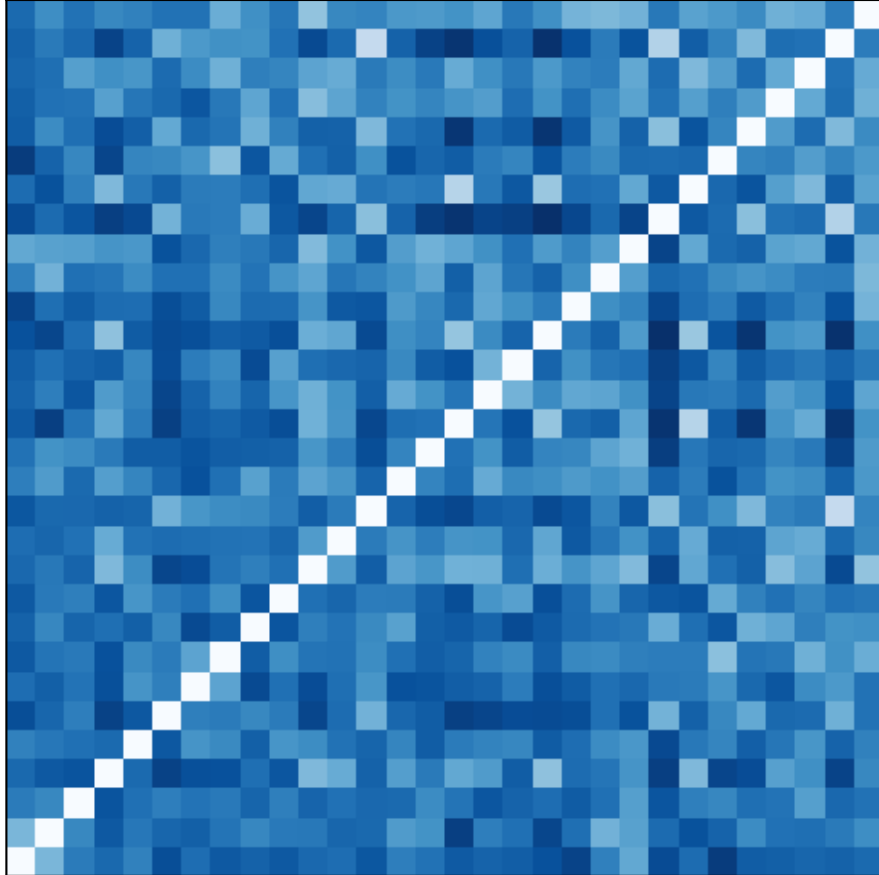

**chr4**

Supplement: Supplementary file 1 [file life-12-00541-s001.zip › life-1592845-supplementary/Heermann-distance-matrix-chr4.pdf]

**chr5**

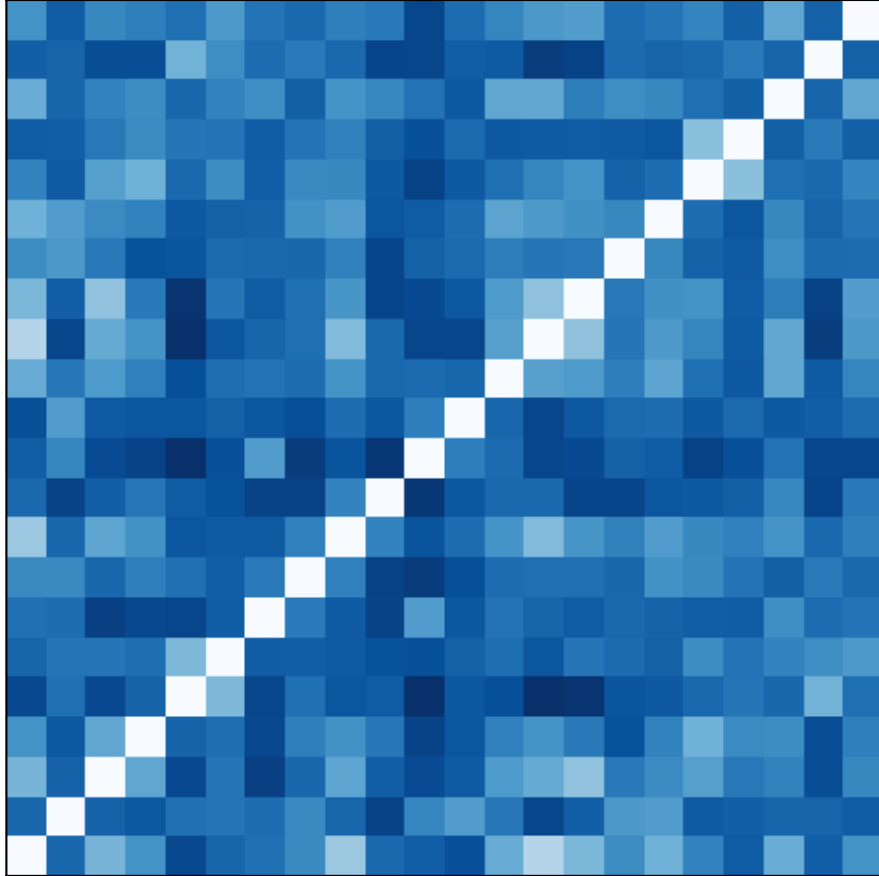

**chr5**

Supplement: Supplementary file 1 [file life-12-00541-s001.zip › life-1592845-supplementary/Heermann-distance-matrix-chr5.pdf]

chr6

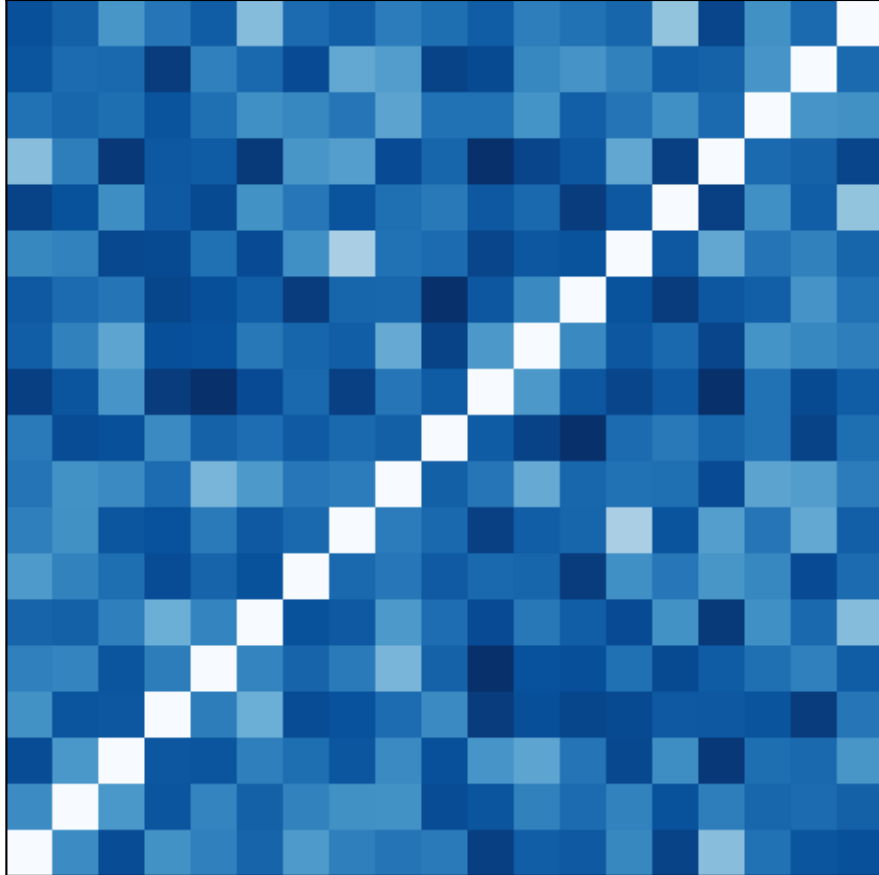

chr6

Supplement: Supplementary file 1 [file life-12-00541-s001.zip › life-1592845-supplementary/Heermann-distance-matrix-chr6.pdf]

chr7

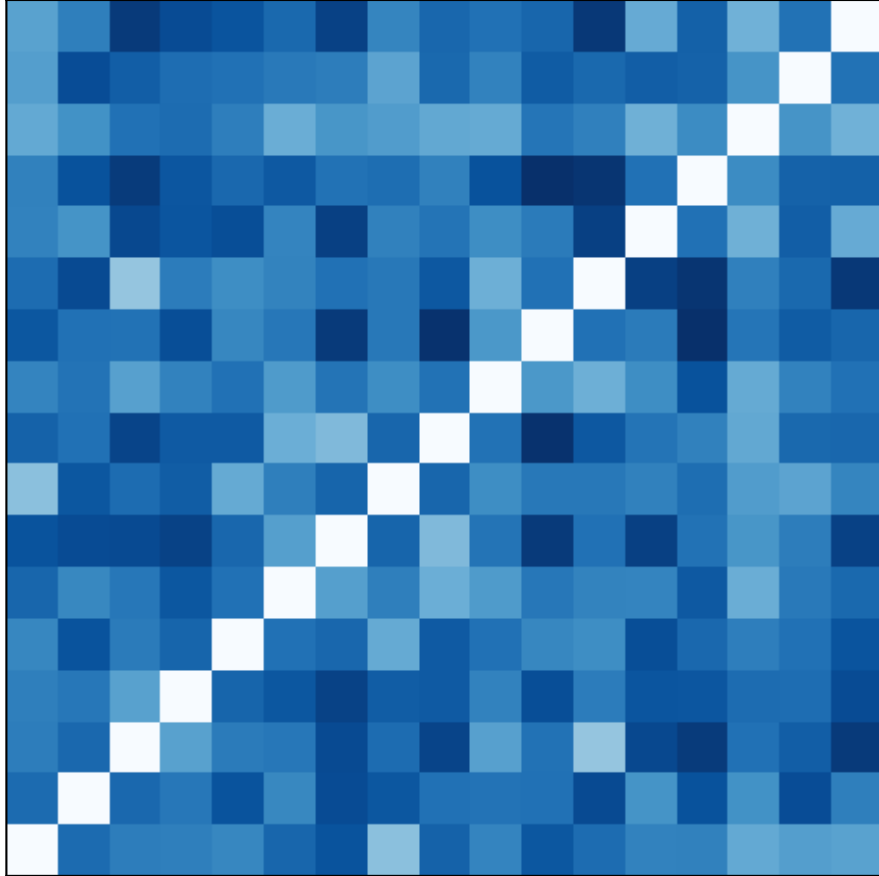

chr7

Supplement: Supplementary file 1 [file life-12-00541-s001.zip › life-1592845-supplementary/Heermann-distance-matrix-chr7.pdf]
